# Diversity and phylogeny of parasitic copepods of freshwater fishes from the Mediterranean and the Middle East

**DOI:** 10.1017/S0031182025100814

**Published:** 2025-10

**Authors:** Robert Míč, Iveta Hodová, Andrea Šímková, Michal Benovics, Radek Šanda, Jasna Vukić, Mária Seifertová

**Affiliations:** 1 Department of Botany and Zoology, Faculty of Science, Masaryk University, Brno, Czech Republic; 2 Department of Zoology, Faculty of Natural Sciences, Comenius University in Bratislava, Bratislava, Slovakia; 3 Department of Ecology, Faculty of Science, Charles University, Prague, Czech Republic

**Keywords:** Cyclopoida, Cyprinids, Ergasilidae, Lernaeidae, Mediterranean, Middle East, Molecular phylogeny, Species diversity

## Abstract

The Mediterranean and the Middle East represent unique biogeographical regions that significantly shaped the evolutionary history and particular diversity of their associated organisms. However, knowledge on the copepods parasitizing freshwater fishes in these regions is limited. This study aims to investigate the diversity and phylogeny of parasitic copepods in freshwater fishes across the Mediterranean and the Middle Eastern regions. A total of 169 freshwater fish species from the Mediterranean and Middle East were examined for metazoan parasites, yielding over 1000 parasitic copepods. A thorough morphological evaluation combined with molecular analyses of partial fragments of rDNA (18S and 28S) and mitochondrial cytochrome c oxidase subunit I (COI) led to the identification of 7 species of Ergasilidae and 3 species of Lernaeidae. These findings include the descriptions of 2 new species: *Ergasilus italicus* n. sp. parasitizing South European nase, *Protochondrostoma genei* (Bonaparte, 1839), in Italy and *Pseudolamproglena zahrziensis* n. sp. found in yellow barbel, *Carasobarbus luteus* (Heckel, 1843), in Iraq. New host and geographical records, along with molecular data are provided for 8 previously described species – *Ergasilus barbi* Rahemo, 1982, *Ergasilus briani* Markevich, 1933*, Ergasilus lizae* Krøyer, 1863*, Ergasilus rostralis* Ho, Jayarajan & Radhakrishnan 1992*, Neoergasilus japonicus* (Harada, 1930)*, Paraergasilus longidigitus* Yin, 1954, *Lamproglena pulchella* von Nordmann, 1832 and *Lernaea cyprinacea* Linnaeus, 1758.

## Graphical abstract

**Figure:**
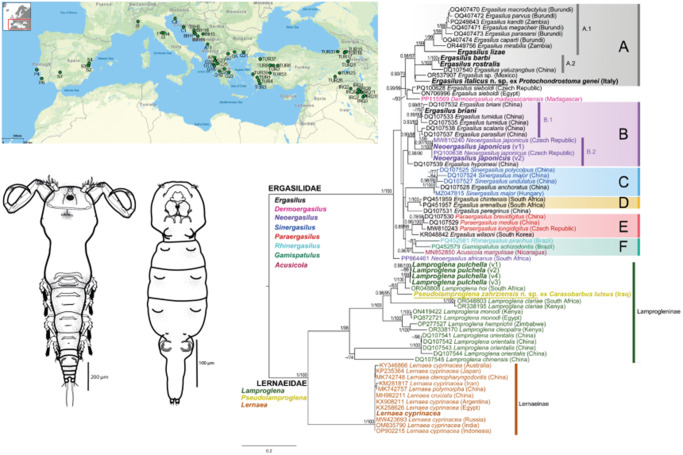

## Introduction

Freshwater parasitic copepods, particularly members of the Ergasilidae and Lernaeidae, are significant pathogens and vectors of fish diseases, impacting fish population dynamics and health (Boxshall and Defaye, 2008; Boxshall and Hayes, 2019). Despite their ecological importance, these parasites remain relatively understudied, and their role in aquatic ecosystems is still not completely clarified. Current knowledge of their diversity varies regionally due to inconsistent research efforts.

Within the Palearctic realm, the Mediterranean and the Middle East are biogeographical regions, each defined by its unique combination of climatic, geological and hydrological conditions that have shaped the evolution and diversity of organisms living in these areas. Both regions are recognized for their high level of biodiversity and endemism, which has long attracted scientific interest (Cuttelod et al., 2009; Freyhof et al., 2014, 2021). However, research on parasitic copepods of the Ergasilidae and Lernaeidae in these regions remains limited and rather uneven. More extensive studies, including the descriptions of new species (*Dermoergasilus cichlidus* Ali and Adday, 2019; *Ergasilus luteusi;* Al-Sahlany et al., 2024; *Pseudolamproglena boxshalli*; Al-Nasiri et al., 2012) in recent years (Al-Nasiri et al., 2012; Ali and Adday, 2019; Al-Sahlany et al., 2024), have been conducted only in a few countries, mainly in Turkey (e.g. Soylu and Soylu, 2012; Koyun et al., 2015; Öktener, 2021) and Iraq (e.g. Mhaisen and Abdul-Ameer, 2021; Mhaisen and Al-Daraji, 2023). In the Mediterranean, particularly in its European part, research on parasitic copepods is also limited to specific regions, several studies were conducted in Bosnia and Herzegovina (Nedić et al., 2014; Skenderović et al., 2015, 2021), Croatia (Tomašec, 1953; Fijan, 1974, 1982), Greece (Zarfdjian and Economidis, 1989; Ragias et al., 2005), Spain (e.g. Simon Vicente et al., 1973; Almeida et al., 2008), Portugal (e.g. Hermida et al., 2008; Bao et al., 2016) and Italy (Grandori, 1925; Fratello and Sabatini, 1972; Macchioni et al., 2015). However, most of these studies do not represent comprehensive research integrating both morphological and molecular approaches, and parasitic copepods have often also remained unidentified (Saraiva and Valente, 1988; Vagianou et al., 2006; Nedić et al., 2014; Stamou et al., 2022).

Until now, 30 species belonging to 6 genera of Ergasilidae [*Dermoergasilus* Ho & Do, 1982 (3), *Ergasilus* von Nordmann, 1832 (19), *Mugilicola* Tripathi, 1960 (2), *Nipergasilus* Yamaguti, 1939 (1), *Neoergasilus* (Yin, 1956) (2) and *Paraergasilus* Markevich, 1937 (3)) and 10 species belonging to 3 genera of Lernaeidae (*Lamproglena* von Nordmann, 1832 (5), *Lernaea* Linnaeus, 1758 (3) and *Pseudolamproglena* (Boxshall, 1976) (2)] have been recorded in the Mediterranean and Middle East regions. A detailed checklist of all records of species of the Ergasilidae and Lernaeidae in the Mediterranean and the Middle East is provided in Supplementary Table S1.

Within the Ergasilidae, *Ergasilus* is currently the most abundant and diverse genus, with up to 19 species reported in these areas. The most widespread species is *Ergasilus sieboldi* von Nordmann, 1832, which has been reported from various freshwater hosts from the Iberian Peninsula to Iraq. In contrast, other *Ergasilus* species have shown more restricted distributions (e.g. *Ergasilus boleophthalmi* Adday & Ali, 2011; *Ergasilus iraquensis* Amado in Amado, da Rocha, Piasecki, Al-Daraji & Mhaisen, 2001; *Ergasilus luteusi* Al-Sahlany et al., 2024; *Ergasilus pararostralis* Amado in Amado, da Rocha, Piasecki, Al-Daraji & Mhaisen, 2001 and *Ergasilus synanciensis* Amado in Amado, da Rocha, Piasecki, Al-Daraji & Mhaisen, 2001 are all restricted to Iraq) or greater host specialization, such as *Ergasilus gibbus* von Nordmann, 1832, which predominantly parasitizes fishes from the family Anguillidae. From the genus *Neoergasilus* have been recorded 2 species, *Neoergasilus longispinosus* (Yin, 1956) on cyprinids in Algeria (Boucenna et al., 2018; Berrouk et al., 2020, 2022) and *Neoergasilus japonicus*, a globally invasive species (Ondračková et al., 2024) of various fish families, has been recorded in several countries in both regions (e.g. Soylu and Soylu, 2012; Mirzaei et al., 2016; Berrouk et al., 2022). Three species of *Paraergasilus* (*Paraergasilus brevidigitus* Yin, 1954; *Paraergasilus inflatus* Ho et al., 1996 and *Paraergasilus longidigitus* Yin, 1954) were recorded in Iraq, Algeria and Turkey (Ho et al., 1996; Koyun et al., 2007; Berrouk et al., 2022). In addition, an introduced non-native species, *Nipergasilus bora* (Yamaguti, 1939), has been recorded on fish hosts of the family Mugilidae in several Mediterranean countries (Paperna, 1975; Ben Hassine, 1983; Koyun et al., 2007). Two species of *Mugilicola* (*Mugilicola bulbosa* Tripathi, 1960 and *Mugilicola kabatai* Piasecki et al., 1991) parasitizing on fishes of the family Mugilidae and 3 species of *Dermoergasilus* (*Dermoergasilus amplectens* (Dogiel & Akhmerov, 1952), *D. cichlidus* and *Dermoergasilus varicoleus* Ho et al., 1992) found on a variety of fish species were reported only in Iraq (Piasecki et al., 1991; Ho et al., 1996; Amado Pinto da Motta et al., 2001; Ali and Adday, 2019; Al-Mosawi and Adday, 2024).

Within the Lernaeidae, 5 species of the genus *Lamproglena* have been recorded, but only *L. pulchella* was found in Europe, with its distribution extending as far as Iraq (Mhaisen et al., 2024). Other *Lamproglena* species, *Lamproglena chinensis* Yü, 1937; *Lamproglena compacta* Markevich, 1936 and *Lamproglena jordani* (Paperna, 19640 have been recorded on the Cyprinidae and Leuciscidae in Iraq, Iran and Israel, respectively, while *Lamproglena monodi* Capart, 1944 was recorded on Cichlidae in Egypt (e.g. 1964; Pazooki and Masoumian, 2012; Hassan et al., 2013). The genus *Lernaea* is predominantly represented by the invasive cosmopolitan species *L. cyprinacea*, which is distributed in almost all Mediterranean and Middle Eastern countries (Ondračková et al., 2024). Additionally, two other species, *Lernaea ctenopharyngodontis* Yin, 1960 and *Lernaea oryzophila* Monod, 1932, have been recorded from cyprinid hosts in Iran and Iraq, respectively (Al-Nasiri et al., 2001; Pazooki and Masoumian, 2012). The genus *Pseudolamproglena* is absent in Europe, but 2 species (*Pseudolamproglena annulata* Boxshall, 1976 and *Pseudolamproglena boxshalli;* Al-Nasiri et al., 2012) were recorded on Cyprinidae, Leucisdidae and Mugilidae in Iraq (Boxshall, 1976; Al-Nasiri et al., 2012).

In this study, we provide an updated overview of the parasitic copepod fauna in freshwater fish species from the Mediterranean and the Middle Eastern regions. The research is based on extensive sampling conducted from 2014 to 2023. This comprehensive dataset offers new insights into the diversity, distribution and host associations of parasitic copepods in these areas, filling important gaps in the current knowledge.

## Materials and methods

### Fish collection

**Figure 1.**
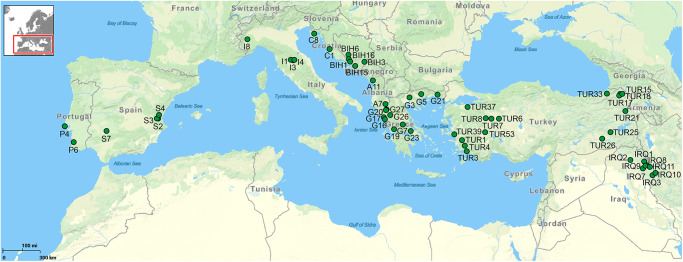
Map of sampling localities with records of parasitic copepods. (P – Portugal, S – Spain, I – Italy, C – Croatia, BIH – Bosnia and Herzegovina, A – Albania, G – Greece, TUR – Turkey, IRQ – Iraq).

During several parasitological surveys between 2014 and 2023, 169 fish species (1484 specimens) were examined for the presence of metazoan parasites. Examined fish included mainly representatives of the Cyprinidae and Leuciscidae (total of 162 species), several fishes of the other families living in sympatry with cyprinoids were also examined (3 species of Gobionidae, 2 species of Nemacheilidae, 1 species of Cobitidae and 1 species of Mugilidae). Fishes were sampled in 155 localities including Spain (13 localities), Portugal (7 localities), Italy (8 localities), Croatia (15 localities), Bosnia and Herzegovina (11 localities), Albania (11 localities), Greece (27 localities), Turkey (52 localities) and Iraq (11 localities) (see Table 1 and Figure 1; for detailed information see Supplementary material Table S2 and Figure S1).

**Table 1.** List of sampled localities with coordinates (only positive records of parasitic copepods listed, all localities are listed in Supplementary Table S2)

Country	ID	Locality	Coordinates
Portugal	P4	Colares	38°47′53.37″N 09°26′14.16″W
	P6	Torgal river, Mira basin	37°38′16.76″N 08°37′10.58″W
Spain	S2	Magro river (1)	39°21′25.76″N 00°39′51.76″W
	S3	Magro river (2)	39°21′18.85″N 00°40′38.85″W
	S4	Turia river	39°34′46.46″N 00°37′09.63″W
	S7	Peraleda de Zaucejo, Zujar river	38°27′12.02″N 05°31′59.67″W
Italy	I3	Torrente Cerfone, Intoppo	43°26′12.03″N 11°58′33.00″E
	I4	Torrente Cerfone, Le Ville	43°28′42.00″N 12°04′25.03″E
	I8	Carmagnola, Cave Germaire	44°51′42.96″N 07°40′26.33″E
Croatia	C1	Baštica river, below the Baštica reservoir/Grabovač reservoir	44°11′42.37″N 15°24′32.13″E
	C8	Pazin, Pazinčica river	45°14′47.92″N 13°58′10.66″E
Bosnia and Herzegovina	BIH1	Krenica lake, Drinovci	43°22′25.00″N 17°19′59.04″E
	BIH3	Mušnica, Avtovac	43°08′42.05″N 18°35′45.00″E
	BIH6	Šujica, Šujičko Polje	43°49′41.43″N 17°10′48.20″E
	BIH15	Rečina river, near Jelim lake, Hutovo Blato	43°03′39.72″N 17°48′29.30″E
	BIH16	Zagorje, Jabuke	43°32′18.53″N 17°12′34.28″E
Albania	A7	Osum, Vodice	40°24′13.07″N 20°39′04.04″E
	A11	Skadar lake, Shiroke	42°03′24.94″N 19°28′07.05″E
Greece	G3	Gallikos, Mandres, Gallikos basin	40°52′07.33″N 22°53′59.12″E
	G5	Angistis, between Alistrati & Drama	41°05′42.08″N 24°00′18.29″E
	G7	Sperchios, Ypati	38°54′14.33″N 22°17′30.22″E
	G16	Acheron, Gliki	39°19′00.05″N 20°36′04.03″E
	G17	Kokitos, Pagrati	39°26′53.02″N 20°30′03.06″E
	G19	Trichonis lake, Panetolio	38°35′20.19″N 21°28′02.68″E
	G20	Aoos, Kalithea	40°01′16.67″N 20°41′40.19″E
	G21	Macropotamos river, Filiouri basin	41°04′13.00″N 25°32′52.00″E
	G23	Yliky Lake	38°25′47.82″N 23°14′37.09″E
	G26	Zagoritikos River, Baldouma	39°41′46.28″N 20°59′46.00″E
	G27	Aoos	40°01′31.39″N 20°41′48.75″E
Iraq	IRQ1	Dukan Lake	36°10′12″N 44°57′24″E
	IRQ2	Great Zab River (1)	36°16′25″N 43°38′40″E
	IRQ3	Darbandikhan Lake	35°07′17″N 45°43′50″E
	IRQ7	Wadi Kalat Shirah, tributary of Tabin River	35°47′3″N 44°58′43″E
	IRQ8	Kani Shok, tributary of Tabin River	35°50′01″N 45°06′16″E
	IRQ9	Zahrzi, Tabin River	35°48′32″N 45°01′20″E
	IRQ10	Grdi Go, Zalm Stream	35°18′26″N 45°58′18″E
	IRQ11	Du Choman, Aw-e Shiler River	35°45′49″N 45°27′12″E
Turkey	TUR1	Çine River, near Çiftlikköy	37°45′48″N 27°50′03″E
	TUR3	Kocaalam Deresi	36°57′12″N 28°17′13″E
	TUR4	Çine River, near Sitmalik	37°24′36″N 28°06′49″E
	TUR6	Çifteler	39°20′40.2″N 31°18′44.8″E
	TUR7	Seyitgazi	39°21′27.5″N 30°35′37.0″E
	TUR8	Kütahya	39°22′48.8″N 30°03′58.9″E
	TUR15	Ardahan, Kura	41°06′56.9″N 42°42′02.5″E
	TUR17	Yiğitkonağı closest village, Çakir, Kura Basin	40°58′00.6″N 42°35′15.9″E
	TUR18	Ölçek, Ölçeksuyu, Kura Basin	41°08′01.4″N 42°51′21.7″E
	TUR21	Kayacik, Cuma stream, Euphrates Basin	39°53′45″N 43°10′34″E
	TUR25	Near Meydan village, inflow of Garzan river, Tigris Basin	38°21′19″N 41°46′48″E
	TUR26	Sinanköy, Akçayır stream (inflow of Batman river), Tigris Basin	37°51′56″N 40°59′21″E
	TUR33	ca 5 km north of Borçka, inflow of Çoruh, Çoruh Basin	41°24′13″N 41°41′47″E
	TUR37	Simav river, Karacabey	40°11′50.526″N 28°21′12.321″E
	TUR39	Sasal stream, Kuner	38°11′58.017″N 27°8′9.305″E
	TUR53	Büyük Menderes, around 1 km east of Işıklı lake	38°12′49.74″N 29°49′16.83″E

The fish sampling was carried out following local regulations. All applicable institutional, national and international guidelines for the care and use of animals were followed. All fish specimens were transported alive to the field laboratory, sacrificed by severing the spinal cord, and dissected within 48 hours following the classical parasitological dissection procedure (Scholz et al., 2018). All fish species used in this study were originally collected and previously used for the studies of monogenean parasites including molecular identification of fish (cytochrome *b*) (see Šimková et al., 2017; Benovics et al., 2018, 2020, 2021a, 2021b, 2021c, 2023, 2024; Nejat et al., 2023, 2025; Rahmouni et al., 2023). All fish sampling and morphological identification in the field was performed by the members of Czech team (Radek Šanda and Jasna Vukić) with contribution of local coworkers in all countries, their names are included in acknowledgements. The present study was part of a larger project concerning host-parasite relationships between monogeneans and their cyprinoid hosts.

### Parasite collection and identification

Live copepods were collected from the gills using fine needles and processed for morphological and molecular purposes, as described in Míč et al. (2023). The mounted specimens in GAP (mixture of glycerine and ammonium picrate) or pure glycerine were studied using an Olympus BX61 microscope equipped with phase contrast optics. Drawings of the copepods were made using an Olympus drawing attachment and edited with a graphic tablet (Wacom Intuos5 Touch) compatible with Adobe Illustrator and Adobe Photoshop (Adobe Systems Inc., San Jose, CA, USA). All measurements (in micrometres) were taken using digital image analysis software (Olympus Stream Motion v. 1.9.3) and are presented as the mean followed by the range and the number (*n*) of specimens measured in parentheses.

For scanning electron microscope analysis, two specimens fixed in 70% ethanol were dehydrated in an increasing ethanol grades, dried in a CPD 030 critical point drying apparatus (Bal-tec, Balzers, Liechtenstein) using liquid CO_2_, mounted on aluminium stubs with double sided adhesive discs, coated with gold in a SCD 040 sputter coating unit (OC Oerlikon Balzers Coating, Balzers, Liechtenstein) and examined in a VEGA scanning electron microscope (TESCAN) operating at 5 kV.

The type specimens of the copepods collected in the present study were deposited in the Institute of Parasitology, Czech Academy of Sciences, České Budějovice, Czech Republic. Prevalence (percentage of infected fish) and mean intensity of infection (mean number of parasites per infected host) were calculated following Bush et al. (1997). Morphological terminology follows Huys and Boxshall (1991), and host nomenclature was checked against the World Register of Marine Species (WoRMS, www.marinespecies.org).

### Molecular and phylogenetic analyses

Genomic DNA was extracted from each individual parasite specimen (or egg sacs only, when applicable) using DNeasy^®^Blood & Tissue Kit (Qiagen, Hilden, Germany) following the manufacturer’s protocol. Three genetic markers were used for molecular identification of copepod species: two partial fragments of nuclear ribosomal DNA (rDNA) regions (28S and 18S rDNA) and one fragment of the mitochondrial cytochrome c oxidase subunit I (COI) gene. The primers used for amplification are listed in Table 2. PCR amplification and sequencing were conducted according to the protocols and conditions outlined in Míč et al. (2023), (2024)). Obtained sequences were edited using Sequencher® v. 4.10.1 (Gene Codes Corporation, Ann Arbor, MI, USA), and the newly generated sequences for parasite species were checked by the nBLAST Search Tool (https://blast.ncbi.nlm.nih.gov/Blast.cgi) to assess any similarity to available congeners and deposited in GenBank (for accession numbers, see Table 3).

**Table 2.** List of primers and PCR conditions used for DNA amplification of partial fragments of ribosomal genes (18S and 28S rDNA) and partial mitochondrial cytochrome oxidase gene (COI) of parasitic copepods

DNA fragment	Primer name	Direction	Sequence (5´−3´)	PCR thermal profile	Product size (bp)	Reference
18S rDNA	18SF	Forward	AAG GTG TGM CCT ATC AAC T	94 °C, 5 min; 40× (94°C, 30 s; 54°C, 30 s; 72°C, 1 min); 72°C, 5 min	∼1300	Song et al. (2008)
	18SR	Reverse	TTA CTT CCT CTA AAC GCT C			
28S rDNA	28SF	Forward	ACA ACT GTG ATG CCC TTA G	94°C, 5 min; 40 × (94°C, 30 s; 54°C, 30 s; 72°C, 1 min); 72°C, 5 min	∼650	Song et al. (2008)
	28SR	Reverse	TGG TCC GTG TTT CAA GAC G			
CO1 mtDNA	ergLCO-modif	Forward	AAYCAYAARGATATTG GNAC	95 °C, 5 min; 40 × (94 °C, 60 s; 45 °C, 60s; 72 °C, 60s); 72 °C, 7 min	∼650	Present study
	ergHCO-modif	Reverse	GGRTGACCRAAAAAYC ARAA

**Table 3.** List of Ergasilidae and Lernaeidae species molecularly analysed in this study, including their host species, locality, total number of isolates, GenBank accession numbers for 18S 28S, COI sequences and values of intraspecific genetic distances. For locality ID abbreviations see Table S2

					GB Acc. No.			Intraspecific p-distance		
Parasite species	Host species	Host family	Loc. ID	N. of isolates	18S	28S	COI	18S	28S	COI
**Ergasilidae**								Mean (in %)/number of genetic variants		
*Ergasilus barbi*	*Barbus escherichii*	Cichlidae	TUR8	3	PX000625	PX000652	–	0/1	0/1	NA
*Ergasilus briani*	*Alburnoides fasciatus*	Leuciscidae	TUR33	2	PX000629	PX000656	PV988327 (hap1)	0/1	0/1	0.81/3
	*Alburnus neretvae*	Leuciscidae	BIH16	1	PX000626	PX000653	PV988324 (hap2)			
	*Gobio artvinicus*	Gobionidae	TUR33	1	PX000630	PX000657	PV988328 (hap1)			
	*Leucos aula*	Leuciscidae	C1	1	PX000627	PX000654	PV988325 (hap2)			
	*Leucos ylikiensis*	Leuciscidae	G23	1	PX000628	PX000655	PV988326 (hap3)			
*Ergasilus italicus* n. sp.	*Protochondrostoma genei*	Leuciscidae	I4	2	PX000631	PX000658	–	0/1	0/1	NA
*Ergasilus lizae*	*Barbus sperchiensis*	Cyprinidae	G7	3	PX000632	PX000659	PV988329 (hap1), PV988330 (hap2)	0/1	0/1	0.47/3
	*Luciobarbus graecus*	Cyprinidae	G7	1	PX000633	PX000660	PV988331 (hap3)			
*Ergasilus rostralis*	*Barbus lacerta*	Cyprinidae	IRQ8	3	PX000634	PX000661	PV988332 (hap1), PV988333 (hap2), PV988334 (hap3)	0/1	0/1	0.21/3
*Neoergasilus japonicus*	*Chondrostoma regium*	Leuciscidae	IRQ11	1	PX000635 (v1)	PX000662 (v1)	PV988335 (hap1)	0.15/2	0.15/2	11.49/3
	*Chondrostoma soetta*	Leuciscidae	I8	1	PX000636 (v2)	PX000663 (v2)	PV988336 (hap2)			
	*Leucos aula*	Leuciscidae	C1	1	PX000637 (v1)	PX000664 (v1)	–			
	*Squalius squalus*	Leuciscidae	C8	1	PX000638 (v2)	PX000665 (v2)	PV988337 (hap3)			
**Lernaeidae**										
*Lamproglena pulchella*	*Barbus cyri*	Cyprinidae	TUR17	2	PX000643	PX000672 (v3)	PV988339 (hap2), PV988340 (hap3)	0/1	0.37/4	13.82/4
	*Garra rezai*	Cyprinidae	TUR25	1	PX000644	PX000673 (v4)	PV988341 (hap4)			
	*Gobio sakaryaensis*	Gobionidae	TUR7	2	PX000645	PX000674 (v1)	–			
	*Protochondrostoma genei*	Leuciscidae	I4	1	–	PX000667 (v1)	–			
	*Scardinius plotizza*	Leuciscidae	BIH15	1	PX000639	PX000666 (v1)	PV988338 (hap1)			
	*Squalius fellowesii*	Leuciscidae	TUR3	3	PX000642	PX000671 (v1)	–			
	*Squalius orpheus*	Leuciscidae	G5	1	PX000641	PX000670 (v2)	–			
	*Squalius squalus*	Leuciscidae	I3	1	PX000640	PX000668 (v1)	–			
	*Squalius vardarensis*	Leuciscidae	G3	1	–	PX000669 (v2)	–			
*Lernaea cyprinacea*	*Chondrostoma meandrense*	Leuciscidae	TUR53	1	PX000649	PX000678	–	0/1	0/1	5.69/2
	*Cyprinion kais*	Cyprinidae	TUR26	1	PX000650	PX000679	PV988345 (hap2)			
	*Parachondrostoma arrigonis*	Leuciscidae	S3	1	PX000648	PX000677	PV988344 (hap2)			
	*Squalius tenellus*	Leuciscidae	BIH6	1	PX000646	PX000675	PV988342 (hap1)			
	*Squalius valentinus*	Leuciscidae	S3	2	PX000647	PX000676	PV988343 (hap2)			
*Pseudolamproglena zahrziensis* n. sp.	*Carasobarbus luteus*	Cyprinidae	IRQ9, IRQ10, IRQ11	4	PX000651	PX000680	PV988346 (hap1), PV988347 (hap2), PV988348 (hap3), PV988349 (hap4)	0/1	0/1	0.27/3

Molecular vouchers (hologenophores, paragenophores; Pleijel et al., 2008) were deposited in the Institute of Parasitology, Czech Academy of Sciences, České Budějovice, Czech Republic.

To investigate the phylogenetic position of collected parasitic copepods to the representatives of parasitic Cyclopoida, 59 sequences of 28S rDNA of the species belonging to 8 genera of Ergasilidae and 2 genera of Lernaeidae were retrieved from GenBank (for details, see Supplementary Table S3). The sequences were aligned with the G‐INS‐i method in MAFFT online service version 7 (Katoh et al., 2019) and ambiguous positions in the alignment were manually edited in BioEdit (Hall, 1999). ModelFinder (Kalyaanamoorthy et al., 2017) was employed to select the most appropriate model of DNA evolution. According to the Bayesian Information Criterion (BIC), GTR + F + I + G4 was selected as the best‐fit model. Both maximum likelihood (ML) analysis and Bayesian inference (BI) were used to reconstruct the phylogenetic tree. The ML tree was constructed using an ultrafast bootstrap method (Hoang et al., 2018) with 1000 replicates in the IQ‐TREE web server (Trifinopoulos et al., 2016). BI analysis was carried out in MrBayes 3.2.6 (Huelsenbeck and Ronquist, 2001), the analysis included 2 simultaneous runs of Markov chain Monte Carlo for 10^6^ generations, sampling every 100 generations, with a ‘burn-in’ of 25%. The trees were visualized and edited in FigTree v. 1.4.3 (Rambaut, 2016) and Adobe Photoshop (Adobe Systems Inc., San Jose, CA, USA). Genetic distances (uncorrected *p*-distance) were calculated in MEGA v. 11 (Tamura et al., 2021).

## Results

**Figure 2.**
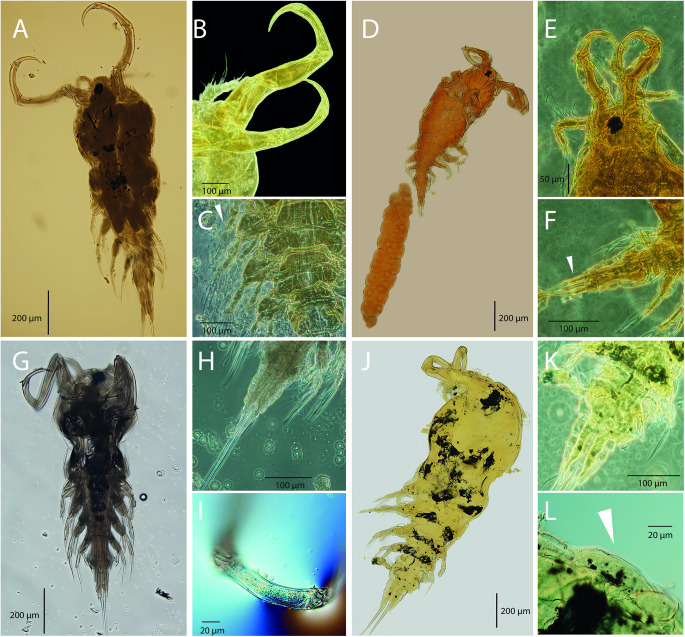
Photomicrographs of representative species from the Mediterranean and the Middle East: (A) *E. barbi*; (B) antennae of *E. barbi*; (C) legs of *E. barbi*, spine on Exp-2 of L1 (white arrow); (D) *E. briani*; (E) antennae of *E. briani*; (F) urosome of *E. briani*, long caudal rami (white arrow); (G) *E. lizae*; (H) urosome of *E. lizae*; (I) antenna of *E. lizae*; (J) *E. rostralis*; (K) urosome of *E. rostralis*; (L) rostrum of *E. rostralis* (white arrow).

**Figure 3.**
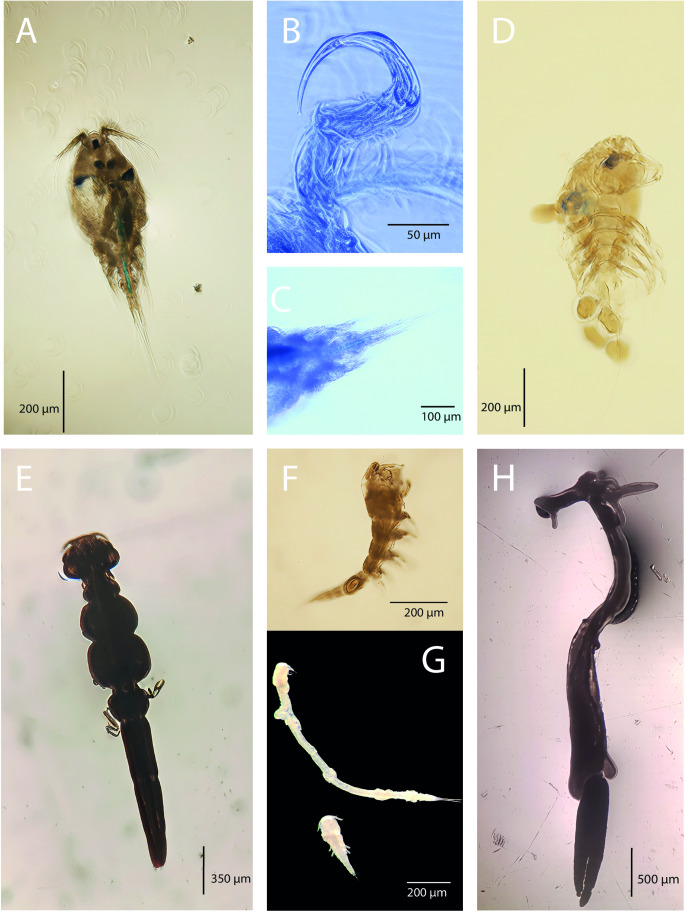
Photomicrographs of representative species from the Mediterranean and the Middle East: (A) *N. japonicus*; (B) antenna of *N. japonicus*; (C) urosome of *N. japonicus*; (D) *P. longidigitus*; (E) *L. pulchella*; (F) copepodid stage of *L. pulchella*; (G) copepodid stages of *L. cyprinacea*; (H) *L. cyprinacea*.

A total of 59 (39 Leuciscidae, 18 Cyprinidae and 2 Gobionidae; 35%) of the 169 fish species sampled in the Mediterranean and the Middle East watersheds were found to be positive for parasitic copepods of the Ergasilidae and Lernaeidae (1004 parasitic copepod adult females). The collected parasites were identified as 6 previously described species of Ergasilidae (*E. barbi, E. briani, E. lizae, E. rostralis, N. japonicus* and *P. longidigitus*) and 2 previously described species of Lernaeidae (*L. pulchella* and *L. cyprinacea*) based on their morphological and molecular characteristics (Figures 2 and 3). Additionally, two new species, *Ergasilus italicus* n. sp. found on *Protochondrostoma genei* (Bonaparte, 1839) in Italy and *Pseudolamproglena zahrziensis* n. sp. parasitizing *Carasobarbus luteus* (Heckel, 1843) in Iraq were described. Their morphological characterization and detailed description are provided below. All species are listed in Table 4, including their host(s), locality of collection, localization on fish and values of abundance, prevalence and intensity of infection. The full list of all fish examined (including non-infected fish) is given in Supplementary Tables S4 and S5.

**Table 4.** List of collected parasitic copepods from respective hosts, including localities of their collection and their epidemiological statistics in the Mediterranean and the Middle East

Parasitic copepod	Host species	Host family	Loc. ID	N	NP	S	L	A	IN	P
**Ergasilidae Burmeister, 1835**										
*Ergasilus barbi* Rahemo, 1982	Barbus escherichii*	Cyprinidae	TUR8	5	5	A	Gills	165	3−72	100%
	*Barbus* sp.*	Cyprinidae	TUR39	5	1	A	Gills	5	5	20%
	Capoeta aydinensis*	Cyprinidae	TUR1	10	4	A	Gills	8	1−3	40%
	Chondrostoma colchicum*	Leuciscidae	TUR6	5	1	A	Gills	1	1	20%
			TUR7	5	1	A	Gills	3	3	20%
*Ergasilus briani* Markevich, 1932	Alburnoides fasciatus*	Leuciscidae	TUR33	5	3	A	Gills	14	2−9	60%
	Alburnus neretvae*	Leuciscidae	BIH3	7	1	A	Gills	2	2	14%
			BIH16	10	9	A	Gills	52	1−14	90%
	Alburnus scoranza*	Leuciscidae	A11	5	3	A	Gills	4	1	60%
	*Alburnus* sp.*	Leuciscidae	TUR37	10	1	A	Gills	1	1	10%
	Gobio artvinicus*	Gobionidae	TUR33	5	1	A	Gills	41	41	20%
	Leucos aula*	Leuciscidae	C1	10	5	A	Gills	16	1−5	50%
	Leucos basak*	Leuciscidae	BIH1	13	12	A	Gills	205	5−27	92%
	Leucos ylikiensis*	Leuciscidae	G23	9	8	A	Gills	84	1−25	89%
	Squalius tenellus*	Leuciscidae	BIH5	3	1	A	Gills	1	1	33%
***Ergasilus italicus* n. sp.**	Protochondrostoma genei*	Leuciscidae	I4	9	4	A	Gills	4	1	44%
*Ergasilus lizae* Krøyer, 1863	Barbus cyclolepis*	Cyprinidae	G21	3	1	A	Gills	20	20	33%
	Barbus sperchiensis*	Cyprinidae	G7	8	5	A	Gills	87	1−64	63%
	Luciobarbus graecus*	Cyprinidae	G7	3	3	A	Gills	5	1−2	100%
*Ergasilus rostralis* Ho et al., 1992	Barbus lacerta*	Cyprinidae	IRQ8	10	3	A	Gills	7	1−4	30%
	Capoeta umbla*	Cyprinidae	IRQ7	10	1	A	Gills	1	1	10%
*Neoergasilus japonicus* (Harada, 1930)	Acanthobrama marmid*	Leuciscidae	IRQ11	10	1	A	Fins	1	1	10%
	Alburnus arborella*	Leuciscidae	I1	10	1	A	Fins	1	1	10%
	Alburnus sellal*	Leuciscidae	IRQ1	2	1	A	Fins	2	1	50%
			IRQ2	1	1	A	Fins	1	1	100%
			IRQ9	7	1	A	Fins	1	1	14%
	Carasobarbus luteus*	Cyprinidae	IRQ3	3	1	A	Fins	1	1	33%
	Chondrostoma soetta*	Leuciscidae	I8	5	2	A	Fins	2	1	40%
	Chondrostoma regium*	Leuciscidae	IRQ11	6	6	A	Fins	18	1 − 5	100%
	Chondrostoma turnai*	Leuciscidae	TUR4	5	3	A	Fins	22	1 − 18	60%
	Cyprinion macrostomus*	Cyprinidae	IRQ1	5	2	A	Fins	2	1	40%
			IRQ3	9	3	A	Fins	3	3	33%
	Leucos aula*	Leuciscidae	C1	10	2	A	Fins	3	1 − 2	20%
	Luciobarbus schejch*	Cyprinidae	IRQ11	7	2	A	Fins	2	1	29%
	Scardinius dergle*	Leuciscidae	C1	10	1	A	Fins	1	1	10%
	Squalius lepidus*	Leuciscidae	IRQ11	9	4	A	Fins	7	1 − 3	44%
	Squalius squalus*	Leuciscidae	C8	10	3	A	Fins	4	2	30%
	Vimba mirabilis*	Leuciscidae	TUR4	10	3	A	Fins	5	1 − 3	30%
*Paraergasilus longidigitus* Yin, 1954	Alburnoides fangfangae*	Leuciscidae	A7	7	1	A	Nose	1	1	14%
	Alburnus scoranza*	Leuciscidae	A11	5	2	A	Nose	2	1	40%
**Lernaeidae Cobbold, 1879**										
*Lamproglena pulchella* von Nordmann, 1832	Acanthobrama microlepis*	Leuciscidae	TUR18	5	1	A	Gills	1	1	20%
	Alburnoides ohridanus*	Leuciscidae	G27	10	1	A	Gills	1	1	10%
	Barbus cyri*	Cyprinidae	TUR17	4	2	A	Gills	5	2 − 3	50%
	Capoeta capoeta*	Cyprinidae	TUR15	7	1	A	Gills	1	1	14%
	Capoeta sieboldii*	Cyprinidae	TUR6	7	1	A	Gills	1	1	14%
	Capoeta tinca*	Cyprinidae	TUR6	5	1	A	Gills	4	4	20%
	Garra rezai*	Cyprinidae	TUR25	5	3	A	Gills	3	1	60%
	Gobio sakaryaensis*	Gobionidae	TUR7	10	4	A	Gills	22	1 − 12	40%
	Protochondrostoma genei*	Leuciscidae	I4	9	2	A	Gills	4	1 − 3	22%
	Sarmarutilus rubilio*	Leuciscidae	I3	10	1	A	Gills	1	1	10%
	Scardinius acarnanicus*	Leuciscidae	G19	4	2	A	Gills	5	2 − 3	50%
	Scardinius plotizza*	Leuciscidae	BIH15	7	6	A	Gills	20	1 − 6	86%
	Squalius fellowesii*	Leuciscidae	TUR3	10	10	A	Gills	58	1 − 13	100%
	Squalius orpheus*	Leuciscidae	G5	1	1	A	Gills	4	4	100%
	Squalius pamvoticus*	Leuciscidae	G16	6	1	A	Gills	1	1	17%
	Squalius prespensis*	Leuciscidae	G20	6	1	A	Gills	1	1	17%
			G27	1	1	A	Gills	3	3	100%
	Squalius squalus*	Leuciscidae	I3	11	1	A	Gills	4	4	9%
	Squalius vardarensis*	Leuciscidae	G3	7	1	A	Gills	1	1	14%
	Telestes pleurobipunctatus*	Leuciscidae	G17	6	3	A	Gills	3	1 − 2	50%
	*Telestes* sp.*	Leuciscidae	G26	11	2	A	Gills	2	1	18%
*Lernaea cyprinacea* Linnaeus, 1758	*Capoeta damascina*	Cyprinidae	TUR21	4	1	A	Skin	1	1	25%
	Chondrostoma meandrense*	Leuciscidae	TUR53	9	1	A	Skin	1	1	11%
	Cyprinion kais*	Cyprinidae	TUR26	8	1	A	Skin	2	2	13%
	*Leucos aula*	Leuciscidae	C1	10	1	A	Skin	1	1	10%
	Luciobarbus guiraonis*	Cyprinidae	S2	1	1	A	Skin	13	13	100%
			S3	10	3	A	Skin	3	1	30%
			S4	1	1	A	Skin	2	2	100%
	Parachondrostoma arrigonis*	Leuciscidae	S3	3	1	A	Skin	1	1	33%
	*Pseudochondrostoma polylepis*	Leuciscidae	P4	15	1	A	Skin	1	1	7%
	Squalius pyrenaicus*	Leuciscidae	S7	5	1	A	Skin	1	1	20%
	Squalius tenellus*	Leuciscidae	BIH6	11	1	A	Skin	2	2	9%
	Squalius torgalensis*	Leuciscidae	P6	10	3	A	Skin	3	1	30%
	Squalius valentinus*	Leuciscidae	S3	5	5	A	Skin	7	1−3	100%
***Pseudolamproglena zahrziensis* n. sp.**	Carasobarbus luteus*	Cyprinidae	IRQ9	8	5	A	Gills	6	1−4	63%
			IRQ10	2	2	A	Gills	7	7	50%
			IRQ11	3	2	A	Gills	3	1−2	67%

*Notes:* N – number of fish hosts; NP – number of fish hosts positive for parasitic copepods; S – stage; L – localization on the host; A – abundance; IN – intensity of infection (min – max); P – prevalence;

* – new host records for the species

*Ergasilus briani* was documented as the most abundant species, its occurrence was confirmed in Turkey, where it was previously recorded (Supplementary Table S1) and now we documented the presence of this species for the first time in Bosnia and Herzegovina, Croatia, Albania and Greece. *Ergasilus lizae* was found on two localities in Greece, confirming its previous presence. The occurrence of *E. barbi* in Turkey, *P. longidigitus* in Albania and *N. japonicus* in Croatia and Iraq were revealed for the first time. *Neoergasilus japonicus* was also found in Italy and Turkey, where its presence had been previously documented (Supplementary Table S1). Within Ergasilidae, the highest host range was observed for *N. japonicus* (14) and *E. briani* (10).

The occurrence of *L. pulchella* was confirmed in Italy and Turkey, and for the first time in Bosnia and Herzegovina and Greece. Moreover, this species exhibited the highest host range encompassing 19 host species (Table 4). *Lernea cyprinacea* was recorded on nine host species in Portugal, Spain, Croatia, Bosnia and Herzegovina and Turkey. It was the only copepod parasite recorded on the Iberian Peninsula in this study. All species of Lernaeidae were found in both copepodid and adult stages.

All *Ergasilus, Lamproglena* and *Pseudolamproglena* specimens were found on the gills, adult *Lernaea* specimens usually burrowed in the skin, *N. japonicus* was attached to the fins and *P. longidigitus* was found in the nasal cavity. Juvenile stages of *Lamproglena, Pseudolamproglena* and *Lernaea* were found on the gills. Each fish specimen typically harboured only a single species of parasitic copepod. Several cases of mixed infections, such as the co-infestation of gills by adult females of Ergasilidae and copepodid stages of Lernaeidae, were reported. The co-occurrence of *N. japonicus* on the fins and an ergasilid species on the gills of a single fish was also found.

**Family Ergasilidae Burmeister, 1835**

**Genus *Ergasilus* Nordmann, 1832**

*Ergasilus italicus* (Míč et al., 2024) n. sp.

***Type-host***: *Protochondrostoma genei* (Bonaparte, 1839) (Leuciscidae)

***Type-locality***: Torrente Cerfone, Tiber River drainage, Le Ville, Italy; 43°28′42″N 12°04′25″E

***Type and voucher material***: Holotype (adult female): IPCAS Cr-40 (1 specimen). Paratype (adult female): IPCAS Cr-40 (1 specimen). Hologenophores (adult females): IPCAS Cr-40 (2 specimens).

***Site on host***: Gill filaments.

***Prevalence and intensity of infection***: 44% (4 fish infected/9 fish examined); 1 specimen per infected host.

***ZooBank registration***: urn:lsid:zoobank.org:act:48903023-9FEB-4CD6-8F8B-2C0E10BF1EF7

***Representative DNA sequences***: A 1355 bp long 18S rDNA sequence and 640 bp long 28S rDNA sequence obtained from 2 specimens are deposited in the NCBI GenBank database under the accession numbers PX000631 and PX000658, respectively.

***Etymology***: The species was named after the country Italy where it was first discovered.

## Description

**Figure 4.**
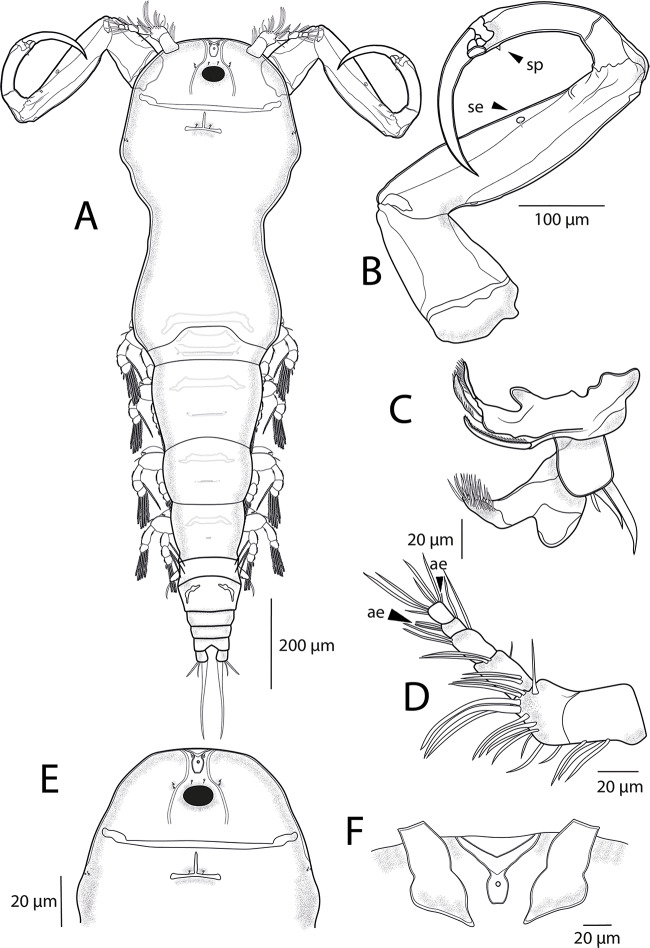
*Ergasilus italicus* Míč & Seifertová, 2025 n. sp., paratype female. (A) habitus, dorsal; (B) antenna with spine (sp) and sensillum (se), ventral; (C) mouthparts, ventral; (D) antennule, distal segment 2 aesthetasc (ae), ventral; (E) cephalosome, dorsal; (F) rostrum, ventral.

**Figure 5.**
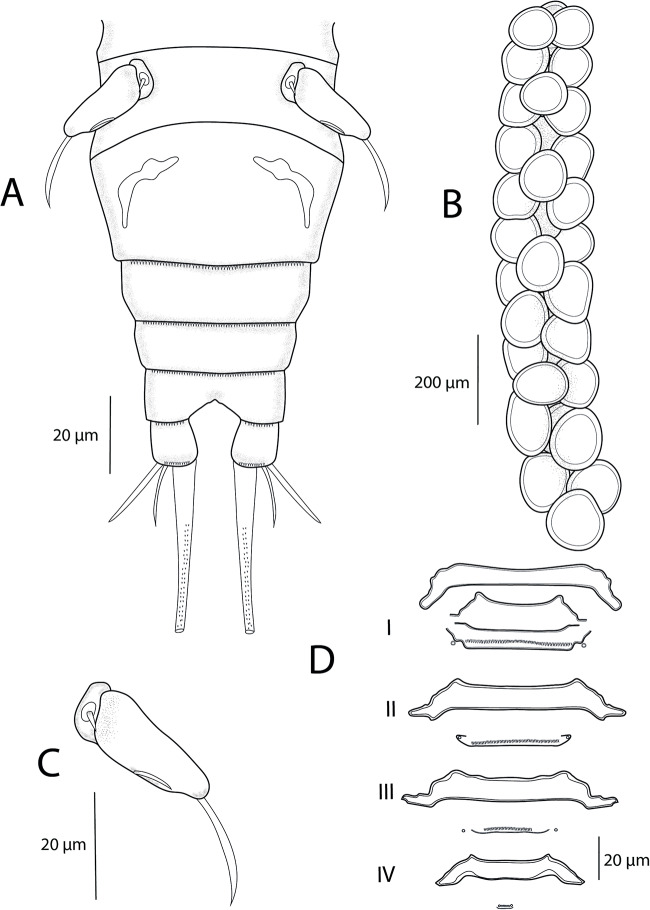
*Ergasilus italicus* Míč & Seifertová, 2025 n. sp., paratype female. (A) abdomen and caudal rami; (B) egg sac, dorsal; (C) leg 5, ventral; (D) interpodal plates, ventral.

**Figure 6.**
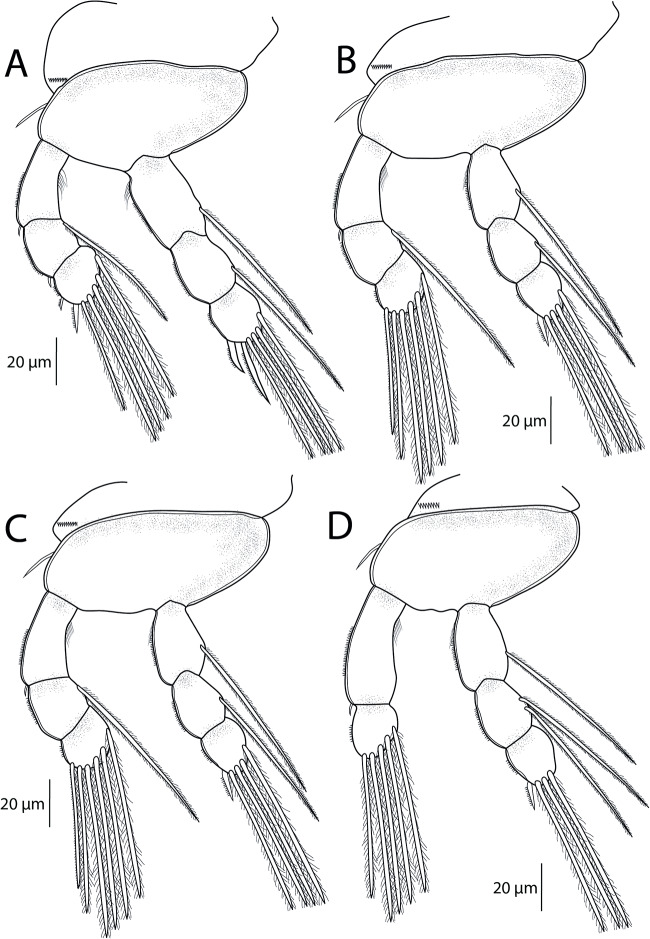
*Ergasilus italicus* Míč & Seifertová, 2025 n. sp., paratype female. (A) leg 1, ventral; (B) leg 2, ventral; (C) leg 3, ventral; (D) leg 4, ventral.

**Figure 7.**
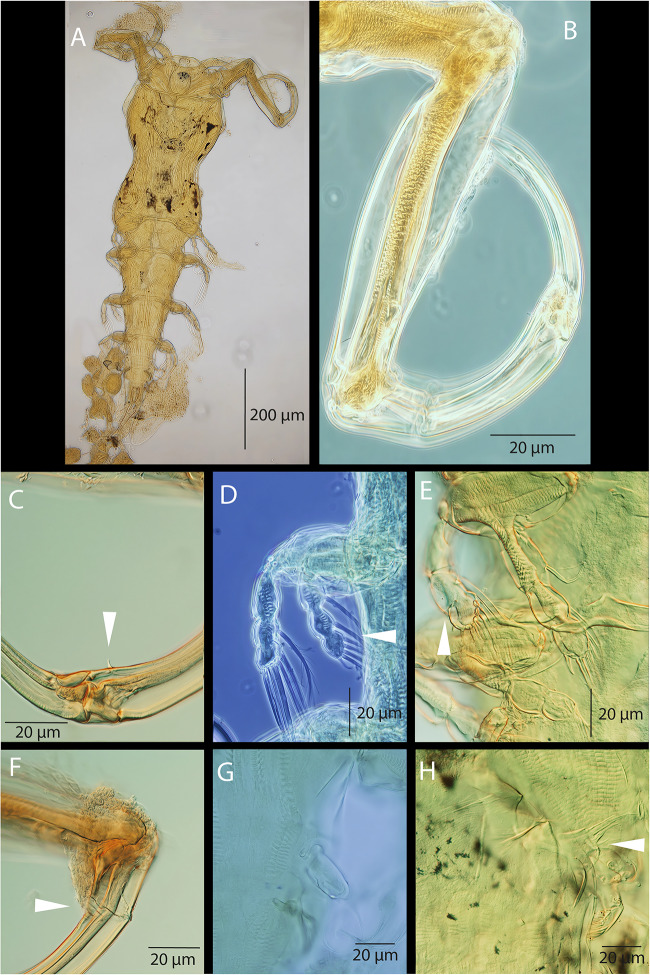
Light microscope photographs of *Ergasilus italicus* Míč & Seifertová, 2025 n. sp., paratype female. (A) habitus, dorsal; (B) antenna, ventral; (C) spine on the antenna (arrow); (D) leg 2 with only 1 seta on the second segment of endopod (arrow); (E) leg 1 with spine on the second segment of exopod (arrow), dorsal; (F) groove on the antenna (arrow); (G) leg 5; (H) maxillule with 3 setae (arrow)

*Adult female.* [Based on 4 specimens; Figures 4–7; measurements in Table 5]. Body length (measured from anterior margin of prosome to posterior margin of caudal rami) 1363 (1360-1365; *n* = 4). Body elongated and comprises prosome, urosome and caudal rami (Fig. 4A; 7A). Prosome 5-segmented, composed of cephalothorax and 3 free pedigerous somites; cephalosome and first pedigerous somite (PS-1) fused together, without distinct separation. Cephalothorax guitar-shaped, much longer than wide, bearing deep indentation between anterior cephalosome and posterior first pedigerous somite. Cephalic ornamentation comprising anterior circular eyespot and inverted T-shaped marking of thickened chitin situated medially on dorsal side. Paired sensory pores and papillae observed on the rostrum, anterior to eyespot and T-shaped marking, as well as on lateral margins of cephalosome (Figure 4E). Rostrum (Figure 4F) well-developed, with triangular posterior margin. Second to fourth pedigerous somites (PS-2 to PS-4) all wider than long and each markedly narrowing posteriorly.

**Table 5.** Measurements (in micrometres) of specimens (*n* = 4) of *Ergasilus italicus* n. sp. parasitizing *Protochondrostoma genei* in Italy

Character	Range	Mean
Total length	1360−1365	1363
Body width	400−403	401
Cephalosome length	Fused	NA
Cephalosome width	399−402	400
Antennule length	128−132	129
Antenna length	674−699	680
Antennal segment 1 length	154−156	155
Antennal segment 2 length	232−250	240
Antennal segment 3 length	165−170	168
Antennal segment 4 (claw) length	123−125	124
Cephalothorax length	616−625	622
Cephalothorax width	342−345	344
Pedigerous somite 2 length	161−169	164
Pedigerous somite 2 width	238−247	242
Pedigerous somite 3 length	154−158	155
Pedigerous somite 3 width	183−194	187
Pedigerous somite 4 length	109−119	115
Pedigerous somite 4 width	134−141	136
Pedigerous somite 5 length	39−40	39
Pedigerous somite 5 width	109−114	112
Genital double somite length	81−83	82
Genital double somite width	110−114	112
Abdominal somite 1 length	28−29	29
Abdominal somite 1 width	96−98	97
Abdominal somite 2 length	25−26	25
Abdominal somite 2 width	77−79	78
Abdominal somite 3 length	21−23	22
Abdominal somite 3 width	66−67	66
Caudal ramus length	23−25	24
Caudal ramus width	20−23	21
Egg-sac length	562−575	565
Egg-sac width	134−137	135

Urosome (Fig. 5A; 7E) comprising fifth pedigerous somite (PS-5), genital double somite and 3 free abdominal somites (AS-1 to AS-3). PS-5 reduced but clearly visible, carrying rudimentary leg 5. Genital double-somite relatively small, wider than long, with transverse row of spinules and pair of hook-shaped ornamentation on ventral side, bearing pair of multiseriate egg sacs dorsally. Free abdominal somites decreasing in width posteriorly. AS-1 wider than long (2.8-3.1 times), slightly larger than AS-2 (1.2 times), bearing transverse row of spinules ventrally at widest part. AS-2 only slightly larger than AS-3 (1.07 times) with transverse row of spinules at midlength. AS-3 (= anal somite) deeply incised posteromedially, with spinules on posterior margin. Caudal rami slightly longer than AS-3 (1.07-1.1 times), slightly longer than wide (1.14-1.16 times) and ornamented with row of spinules towards distal margin. Each caudal rami ornamented ventrally with row of spinules on posterior margin and each bearing 3 terminal setae – innermost longest and thickest, ornamented with transversal rings of inconspicuous scales at posterior 3/4. Egg sacs (Figure 5B) long and multiseriate, much longer than wide (4.2 times), each composed of 2–3 rows of eggs.

Antennule (Figure 4D) 6-segmented, tapering, armed with long and short setae. The margin between the first and second segments inconspicuous (fused dorsally). Setal formula from proximal to distal segments: 3–13–5–3–2 + ae–6 + ae. Antenna (Fig. 4B; 7B) 4-segmented, comprising coxobasis, 3-segmented endopod (Enp-1 to Enp-3) and curved terminal claw. Enp-1 (proximal) longest, nearly 1.55 times longer than coxobasis, tapering distally, bearing one sensillum distally on the concave margin; Enp-2 (medial) elongated, slightly curved, about 0.7 length of Enp-1, with prominent spine distally on anterior margin and with conspicuous groove in cuticle on inner side (Figure 7C, F). Enp-3 inconspicuous, unornamented. Terminal claw long and curved, about 0.7 size of Enp-2, unornamented.

Mouthparts (Fig. 4C; 7H) comprising mandible, maxillule and maxilla; maxilliped absent. Mandible consisting of 3 blades (anterior, middle and posterior); anterior blade with sharp teeth on anterior margin; middle blade with sharp teeth on both margins; and posterior blade with sharp teeth on anterior margin. Maxillule bearing 2 unequally long smooth outer setae and 1 minute inner seta. Maxilla 2-segmented, comprising unarmed syncoxa and basis, distally with numerous sharp teeth on anterior margin.

Swimming legs (L1 to L4) biramous; each comprising coxa, basis, endopod (inner ramus) and exopod (outer ramus) (Figure 6). Intercoxal sclerites (Figure 5D) slender; each with tapering ends directed posterolaterally, unornamented. Interpodal plates slender (Figure 5D), each different in shape and decreasing in size, each with 2 inconspicuous bilateral pores and row of spinules. Armature formula of L1–L4 (spines – Roman numerals; setae – Arabic numerals) shown in Table 6.

**Table 6.** Spine (Roman numerals) and setal (Arabic numerals) formula of swimming legs of Ergasilus italicus n. sp

	Coxa	Basis	Exopod	Endopod
Leg 1	0−0	1−0	I−0; I−1; II−5	0−1; 0−1; II−4
Leg 2	0−0	1−0	I−0; 0−1; 0−6	0−1; 0−1; I−4
Leg 3	0−0	1−0	I−0; 0−1; 0−6	0−1; 0−1; I−4
Leg 4	0−0	1−0	I−0; 0−5	0−1; 0−2; I−3

Coxa of all legs unarmed; coxa of L1-L4 with a row of spinules extending along its outer posterior margin. Basis of all legs armed with proximal outer spine, unornamented. L1–L4 with outer margin of both rami ornamented with rows of spinules; outer and inner margin of first endopodal and exopodal segment, respectively, of all legs partly or completely covered with bristles.

Leg 1 (Fig. 6A; 7E): exopod 3-segmented; first segment with small naked spine arising from outer posterior margin; second segment with small naked spine arising from outer posterior margin and 1 inner plumose seta; third segment with 2 blade-like serrated spines (shorter more proximal) and 5 plumose setae. Endopod 3-segmented; first and second segments each with 1 plumose seta; third segment with 4 plumose setae and 2 blade-like serrated spines.

Leg 2 (Fig. 6B; 7D): exopod 3-segmented; first segment with small naked spine arising from outer posterior margin; second segment with 1 plumose seta; third segment with 6 plumose setae. Endopod 3-segmented; first and second segments each with 1 plumose seta; third segment with 1 blade-like serrated spine and 4 plumose setae.

Leg 3 (Figure 6C) with same ornamentation and armament described for L2.

Leg 4 (Figure 6D): exopod 2-segmented; first segment elongated, with small naked spine arising from outer posterior margin; second segment with 5 plumose setae. Endopod 3-segmented; first segment with 1 plumose seta; second segment with 2 plumose setae; third segment with 1 slender blade-like serrated spine and 3 plumose setae.

Leg 5 (Fig. 5C; 7G): reduced, but clearly visible, 2-segmented. Basal segment very small, bearing outer seta. Distal segment rectangular with 1 small seta on lateral margin and 1 long apical seta.

Specimens preserved in ethanol faint brown in colour, with dark brown spots in the cephalothorax.

Male: unknown

## Remarks

*Ergasilus italicus* n. sp. has a combination of unique morphological characteristics distinguishing it from other species of *Ergasilus*. The most prominent distinguishing features are the morphology of the cephalothorax, and the leg armature formula. With an overall mean body length exceeding 1350 μm, *E.italicus* n. sp. also belongs to one of the largest species currently known.

The leg armature formula of *E. italicus* n. sp. closely resembles that of *E. sieboldi*, particularly in the presence of a spine on the outer margin of the second exopod segment of leg 1. However, a key difference lies in the armature of the second endopod segment of legs 2 and 3. While most species of *Ergasilus* carry 2 inner setae on this segment, *E. italicus* n. sp. bears only 1 seta. This feature is shared among European species only with *E. gibbus* and *Ergasilus tumidus* Markevich, 1940, but *E. italicus* n. sp. differs from *E. gibbus* by having: (i) two-segmented leg 5 (*vs* small one-segmented papilla); (ii) 2 setae on the second endopod segment of leg 4 (*vs* 1 seta); (iii) 2 unequal setae and 1 minute seta on maxillule (*vs* only 2 setae on maxillule); and from *E. tumidus* by having: (i) spine on Enp-2 of the antenna (*vs* absence); (ii) two-segmented leg 5 (*vs* small one-segmented papilla); (iii) outer spine on Enp-3 of legs 2 and 3 (*vs* absence). All other species of *Ergasilus* recorded in Europe are characterized by the presence of 2 setae on the second endopod segment of legs 2 and 3. The descriptions of *Ergasilus boettgeri* Reichenbach-Klinke, 1958, *Ergasilus osmeri* Beneden, 1870 and *Ergasilus suboculatus* (Hesse, 1871) do not include the leg armature formula. However, based on the available drawings, the shape of the cephalothorax and antenna do not match *E. italicus* n. sp.

The guitar-shaped cephalothorax has been noted in 12 currently known species worldwide: *Ergasilus arthrosis* (Roberts, 1970) from the USA; *Ergasilus atafonensis* Amado & Rocha, 1996 from Brazil; *Ergasilus bahiensis* Amado & Rocha, 1996 from Brazil; *E. barbi* from Iraq; *E. briani* from many countries in Europe and China; *Ergasilus curticrus* Muriel-Hoyos, Santana-Pineros, Cruz-Quintana & Suarez-Morales, 2015 from Colombia; *Ergasilus cyanopictus* Carvalho, 1962 from Brazil; *E. iraquensis* from Iraq; *Ergasilus mirabilis* Oldewadge & Van As, 1987 from South Africa; *E. mosulensis* from Iraq and *Ergasilus parabahiensis* El-Rashidy & Boxshall, 1999 from Guyana.

Only 3 species (*E. barbi, E. luteusi* and *E. mosulensis*) share the combination of the guitar-shaped cephalothorax and in the same time have only 1 seta on the armature of the second endopod segment of legs 2 and 3. The new species differs from *E. barbi* by having: (i) only 1 spine on the antenna (*vs* 3 spines on the antenna); (ii) only 2 setae on leg 5 (*vs* 3 setae on leg 5); (iii) mean body length over 1350 µm (*vs* mean body length 813–1138 µm). It is clearly distinguished from *E. luteusi* by having: (i) cephalosome completely fused with the first pedigerous somite (*vs* well-developed depression between the cephalosome and the first pedigerous somite; (ii) only 1 spine on the antenna (*vs* 3 spines on the antenna); (iii) 2 unequal setae and 1 minute seta on maxillule (*vs* only 2 unequal setae on maxillule). It also differs from *E. mosulensis* by having: (i) an outer spine on the second segment of the exopod of leg 1 (*vs* absence); (ii) only 1 spine on the antenna (*vs* 3 spines on the antenna); (iii) only 2 setae on leg 5 (*vs* 3 setae on leg 5).

*E. italicus* n. sp. represents the second ergasilid copepod described solely from Italy (after *Ergasilus lagunaris* Grandori, 1925).

**Family Lernaeidae Cobbold, 1879**

**Genus *Pseudolamproglena*** (Boxshall, 1976)

*Pseudolamproglena zahrziensis* (Míč et al., 2024) n. sp.

***Type-host***: *Carasobarbus luteus* (Heckel, 1843) (Cyprinidae)

***Type-locality***: Zahrzi, Tabin River, Tigris River drainage, Iraq; 35°48′32″N 45°01′20″E

***Additional localities***: Grdi Go, Zalm Stream, Tigris River drainage, Iraq; 35°18′26″N 45°58′18″E and Du Choman, Aw-e Shiler River, Tigris River drainage, Iraq; 35°45′49″N 45°27′12″E

***Type and voucher material***: Holotype (adult female): IPCAS Cr-41 (1 specimen). Paratypes (adult females): IPCAS Cr-41 (1 specimen). Hologenophores (adult females): IPCAS Cr-41 (3 specimens).

***Site on host***: Gill filaments.

***Prevalence and intensity of infection***: 69% (9 fish infected/13 fish examined); 1–4 specimens per infected host.

***ZooBank registration***: urn:lsid:zoobank.org:act:00366F43-B1C2-4F48-9040-5B8B3FE43BA7

***Representative DNA sequences***: A 1395 bp long 18S rDNA sequence, 733 bp long 28S rDNA sequence and 3 haplotypes of 620 bp long COI sequences obtained from 4 specimens are deposited in the NCBI GenBank database under the accession numbers PX000651, PX000680 and PV988346–PV988349, respectively.

***Etymology***: The species was named after the city of Zahrzi in Iraq, near which it was first discovered.

## Description

**Figure 8.**
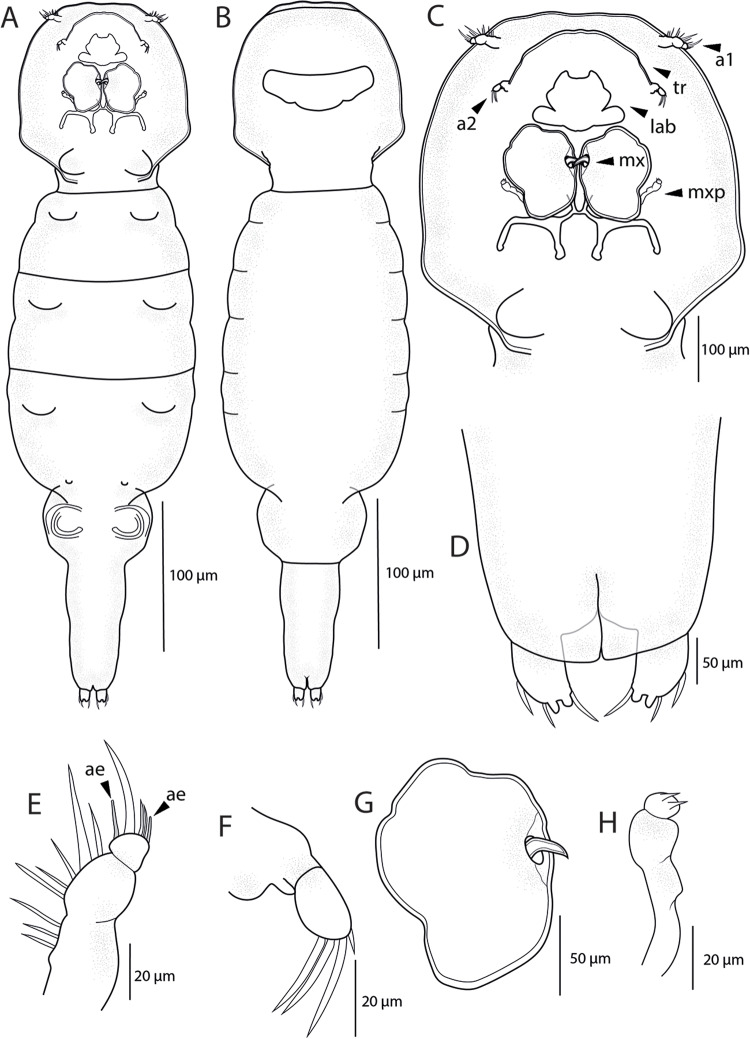
*Pseudolamproglena zahrziensis* Míč & Seifertová, 2025 n. sp., paratype female. (A) habitus, ventral; (B) habitus, dorsal; (C) cephalothorax with antennule (a1), antenna (a2), transversal ridge (tr), labrum (lab), maxilla (mx), maxilliped (mxp), ventral; (D) caudal rami; (E) antennule, distal segment 2 aesthetasc (ae); (F) antenna; (G) maxilla; (H) maxilliped.

**Figure 9.**
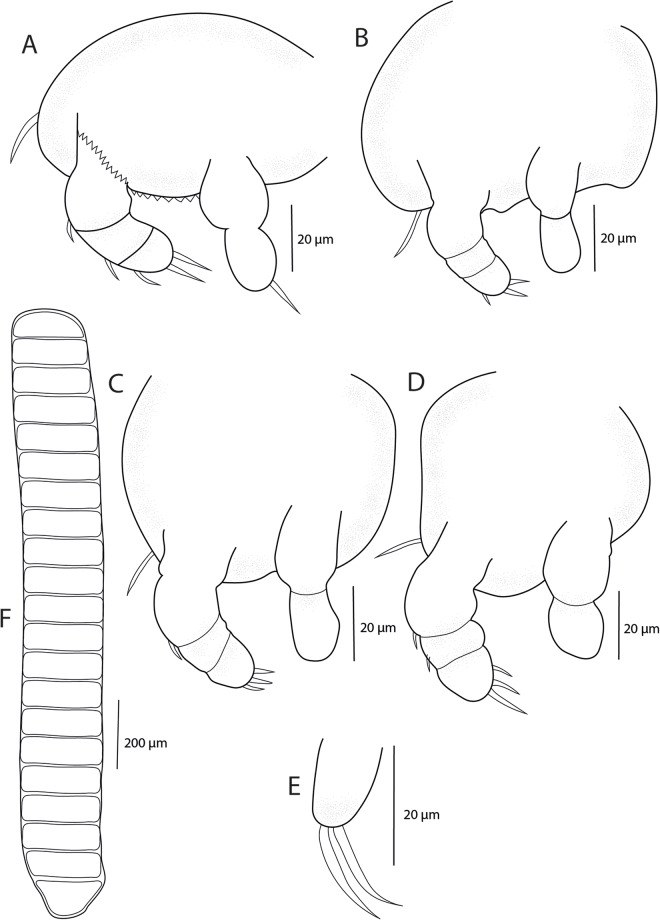
*Pseudolamproglena zahrziensis* Míč & Seifertová, 2025 n. sp., paratype female. (A) leg 1; (B) leg 2; (C) leg 3; (D) leg 4; (E) leg 5; (F) egg sac.

**Figure 10.**
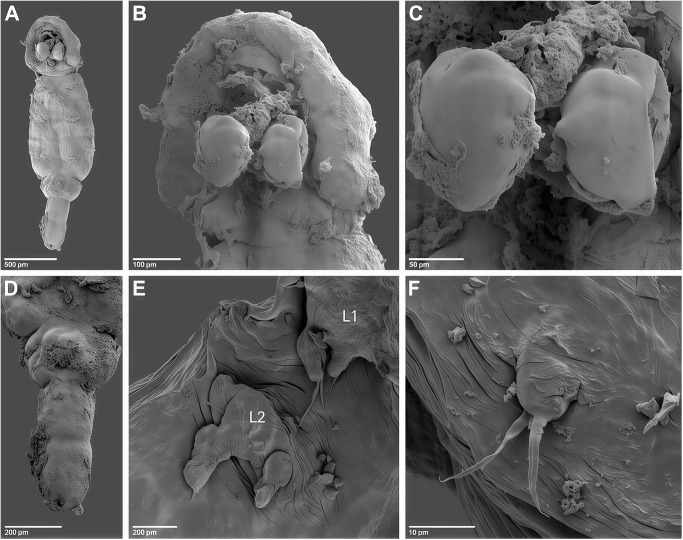
Scanning electron micrographs of *Pseudolamproglena zahrziensis* Míč & Seifertová, 2025 n. sp., paratype female. (A) habitus, ventral; (B) cephalothorax, ventral; (C) maxilla; (D) abdomen and caudal rami, ventral; (E) leg 1 and leg 2; (F) leg 5.

*Adult female.* [Based on 10 specimens; Figures 8–10].

Body length (measured from anterior margin of head to posterior margin of caudal rami) 2172 µm (1816–2445 µm; *n* = 10). Body cylindrical and indistinctly segmented (Fig. 8A, B; 10A). Cephalothorax (Fig. 8C; 10B) broad, dorsal surface concave, comprising 16-20% of total body length. First pedigerous somite incorporated into cephalothorax, narrowing posteriorly to form ‘neck’ between cephalothorax and trunk. Second to fourth pedigerous somites separated by intersegmental sutures, subdivided into anterior and posterior portions by a transverse groove, equal in width. Thoracic legs located anterior to groove. Pedigerous somites increasing in size posteriorly.

Genital complex small, narrower than fourth pedigerous somite and with conspicuous dorsal swellings marking the genital apertures situated dorso-laterally. Abdomen elongate (Figure 10D), consisting of two indistinctly divided somites, narrower than fourth pedigerous somite and genital complex. Posterior margin of anal somite bilobate, bearing medially directed caudal rami. Egg string (Figure 9F) uniseriate, containing up to 21 eggs.

Antennule (Figure 8E) situated on ventral surface near anterior margin of cephalothorax, directed posterolaterally, only apical segment clearly delimited. Armature comprising 8 setae on anterior margin of proximal segment, 3 setae on distal segment and 2 aesthetasc-like structures.

Antenna (Figure 8F) situated lateral to transverse ridge on ventral surface of cephalothorax, curved posteriorly, indistinctly two-segmented with 3 longer and 1 shorter setae.

Oral region (Fig. 8C; 10B) occupied by large trilobed labrum. Transverse ridge present on ventral surface of cephalothorax anterior to trilobed labrum. Maxillule absent. Maxilla (Fig. 8G; 10C) large, indistinctly two-segmented; proximal segment broad, distal segment marked by transverse constriction and armed with robust dorsally curved claw on medial surface. Maxilliped (Figure 8H) indistinctly three-segmented, proximal segment connected by transverse ridge of tissue on ventral surface, middle segment elongated and widening distally, terminal segment round and armed with two setiform spines on medial surface.

Thoracic legs 1-4 (Figure 9) similar, biramous. Sympod projecting from body surface, bearing single seta lateral to exopod base. Endopod indisctinctly two-segmented, exopod three-segmented. Leg 1 (Fig. 9A; 10E) sympod with serrated distal margin; endopod with one terminal seta; exopod with 3 terminal setae and 1 lateral spine on both middle and proximal segments. Leg 2 (Fig. 9B; 10E) endopod unarmed; exopod with 3 short terminal setae on distal segment. Leg 3 (Figure 9C) endopod unarmed; exopod with 3 short terminal setae on distal segment and 1 lateral spine on proximal segment. Leg 4 (Figure 9D) endopod unarmed; exopod with 3 terminal setae on distal segment and 1 lateral spine on both middle and proximal segments. Leg 5 (Fig. 9E; 10F) simple process with 2 apical setae, positioned anteriorly on ventral surface of genital complex.

Caudal ramus (Figure 8D) armed with 3 setae; 1 on lateral margin and 2 at each posterolateral corner. Two small papillae present on distal margin.

## Remarks

Currently, there are 4 species of *Pseudolamproglena* described, with similar general body shape, but differences in other morphological traits. *Pseudolamproglena sinilabis* (Kuang, 1980) is the longest of all the species of the genus, with the total length of 2930–3670 µm (Kuang, 1980). In comparison, *P. boxshalli* is less than 2600 µm, *Pseudolamproglena simplex* (Boxshall, 1976) less than 2450 µm (Boxshall, 1976) and *P. annulata* less than 2200 µm (Boxshall, 1976), while the newly described species less than 2450 µm. *P. zahrziensis* n. sp. is also distinguishable from *P. sinilabis* in its armature on the antenna with 4 setae (*vs* 8), bearing three-segmented exopods on all four pairs of legs (*vs* two-segmented exopods), caudal rami with 3 setae (*vs* 6) and posession of maxillipeds (*vs* maxillipeds absent).

*P. zahrziensis* n. sp. shares the same armature of the maxillipeds (2 setae on the distal segment) with *P. simplex*, but differs from it by having more distinctly segmented body, the absence of maxillules (*vs* presence), a presence of trilobed labrum in the oral region (*vs* hemispheric labrum), caudal rami with 3 setae (*vs* 6) and differences in the armature of the legs 1-4.

*P. zahrziensis* n. sp. is similar to *P. annulata* and *P. boxshalli* in its distinctly segmented body, the absence of the maxillule and the presence of the large trilobate labrum in the oral region. It also shares the same caudal rami armature (3 setae) and serrated distal margin of sympod with *P. boxshalli*, and similar antennule armature (8 setae on the proximal segment) and presence of single seta lateral to exopod of legs 1-4 with *P. annulata*. However, it differs from *P. annulata* in the armature of the maxillipeds with 2 setae on the distal segment (*vs* 1), caudal rami with 3 setae (*vs* 4), serrated distal margin of sympod (*vs* smooth) and differences in the armature of the legs 1-4. From *P. boxshalli* it differs in the armature of the maxillipeds with 2 setae on the distal segment (*vs* 9), the armature of the antennules with 8 setae on the proximal segment (*vs* 14), presence of single seta lateral to exopod of legs 1-4 (*vs* absence) and in differences in the armature of the legs 1-4.

### Molecular characterisation and phylogenetic relationships of Mediterranean and Middle East Ergasilidae and Lernaeidae

In this study, the first molecular data were obtained for parasite copepod species *E. barbi, E. lizae, E. rostralis* and *L. pulchella*. Despite many attempts, no molecular data were obtained for specimens of *P. longidigitus,* probably due to inappropriate fixation or drying up of the specimens during long term storage. No intraspecific genetic variability was observed for rDNA sequences of *E. barbi, E. briani, E. lizae, E. italicus* n. sp., *P. zahrziensis* n. sp. and *L. cyprinacea*. Two genetic variants of 18S and 28S rDNA were observed for *N. japonicus* (variant 1: PX000635, PX000637 (18S), PX000662, PX000664 (28S); variant 2: PX000636, PX000638 (18S), PX000663, PX000665 (28S)), which were identical with 2 type of genetic variants previously observed in the Czech Republic (Ondračková et al., 2019). For *L. pulchella*, 4 closely related genetic variants of 28S rDNA (variant 1: PX000666, PX000667, PX000668, PX000671, PX000674; variant 2: PX000669, PX000670; variant 3: PX000672; variant 4: PX000673), but only one variant of 18S rDNA were found. Intraspecific mean *p*-distance values are listed in Table 3. From 2 to 4 unique COI haplotypes were obtained for species studied, except *E. italicus* n. sp. and *E. barbi* (even if several PCR modifications and primers combinations were tested, PCR failed). The intraspecific COI variation ranged from 0.21 % in *E. rostralis* to 13.82 % in *L. pulchella* (Table 3).

**Figure 11.**
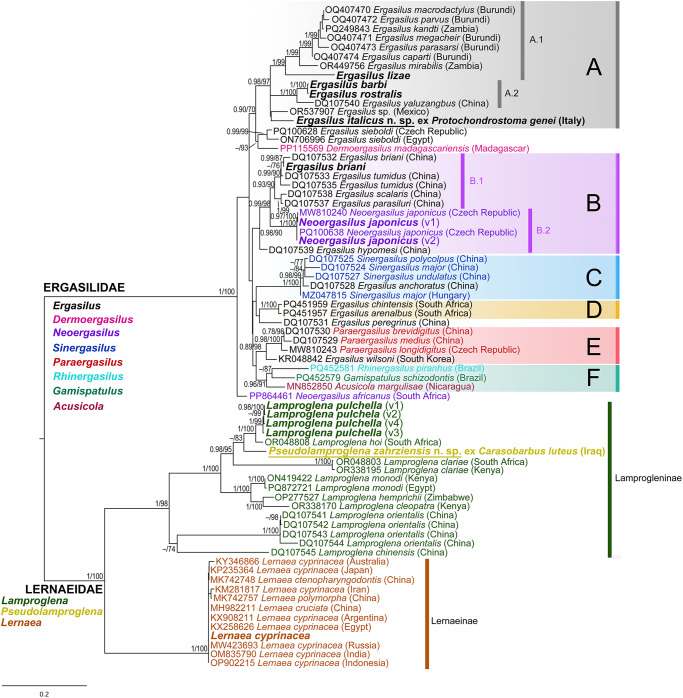
Phylogenetic tree of Ergasilidae and Lernaeidae reconstructed by Maximum Likelihood. The tree is based on the partial 28S rDNA sequences. Values along the branches indicate posterior probabilities from Bayesian Inference and bootstrap values from Maximum Likelihood (dashes indicate values below 0.7 and 50, respectively). New sequences are in bold and newly described species are underlined. Detailed information about localities and accession numbers are given in Table 3.

The tree topologies obtained by ML and BI methods were almost identical, and the resulting phylogram based on the ML analysis of 28S rDNA sequences with posterior probabilities (BI) and bootstrap values (ML) along nodes is presented in Figure 11. The phylogenetic analyses confirmed the presence of several well-supported clades congruent with previous studies (e.g. Míč et al., 2023, 2024; Jansen et al., 2024; Narciso et al., 2024). The species of the Ergasilidae formed 6 well-supported clades (A-F, Figure 11). Species of *E. barbi, E. lizae, E. rostralis* and *E. italicus* n. sp. were placed in clade A (BI = 0.98; ML = 97), which includes species parasitizing African cichlids [*Ergasilus macrodactylus* (Sars, 1909)*, Ergasilus parvus* (Míč et al., 2023)*, Ergasilus kandti* van Douwe, 1912*, Ergasilus megacheir* (Sars, 1909)*, Ergasilus parasarsi* (Míč et al., 2023) and *Ergasilus caparti* (Míč et al., 2023)] and catfishes (*E. mirabilis*), the Chinese species *Ergasilus yaluzangbus* Kuang & Qian, 1985, and unidentified species of *Ergasilus* from the endemic *Floridichthys polyommus* Hubbs, 1936 in the Yucatán Peninsula. *Ergasilus lizae* was placed in a basal position within the African subclade A.1., *E, barbi* and *E. rostralis* formed a well-supported subclade A.2 (BI = 1; ML = 100) with the Chinese species *E. yaluzangbus*, positions of the new described species *E. italicus* n. sp. and an unidentified Mexican species were unresolved within clade A. The species of *Ergasilus briani* and *N. japonicus* were included in clade B, *E. briani* together with 3 other Chinese species (*E. tumidus, Ergasilus scalaris* Markevich, 1940 and *Ergasilus parasiluri* (Yamaguti, 1936)) formed subclade B.1 (BI = 0.99; ML = 99), and two genetic variants of *N. japonicus* from Mediterranean and Middle East were included in subclade B.2 (BI = 1; ML = 100).

Within Lernaeidae, species were formed by 2 big clades, corresponding to subfamilies Lamprogleninae and Lernaeinae (BI = 1; ML = 100). All observed genetic variants of *L. pulchella* and the new described species *P. zahrziensis* n. sp. clustered together with African species *Lamproglena hoi* Dippenaar, Luus-Powell & Roux, 2001 and *Lamproglena clariae* Fryer, 1956 (BI = 0.98; ML = 95). The newly obtained sequence of *L. cyprinacea* was placed with others *L. cyprinacea* sequences isolated from diverse fish hosts from different parts of world and Chinese species *Lernaea cruciata* (Lesueur, 1824), *L. ctenopharyngodontis* and *Lernaea polymorpha* (Yü, 1938) (BI = 1; ML = 100).

## Discussion

In the present study, parasitic copepods were recorded on 60 fish host species across 9 countries. These findings, which include the identification of 10 species from six genera, contribute to the growing knowledge of parasitic copepod diversity in the Mediterranean and Middle East regions. Comparisons with previous records of 30 species belonging to 6 genera of Ergasilidae and 10 species of 3 genera of Lernaeidae in the Mediterranean and Middle Eastern regions reveal both consistencies and novel findings.

Within the Ergasilidae, 7 species representing 3 genera were found, with the highest species richness observed for the genus *Ergasilus*. No species were recorded from the genera *Dermoergasilus, Mugillicola* and *Nipergasilus* that had previously been reported in Mediterranean areas and Iraq. From the 19 previously described *Ergasilus* species in the Mediterranean and Middle Eastern regions, only *E. barbi, E. briani, E. lizae* and *E. rostralis* were found in this study.

*Ergasilus briani* was found to be the most prevalent and abundant species, exhibiting the widest distribution range. In this study, its presence has been recorded for the first time in Bosnia and Herzegovina, Croatia, Albania and Greece. However, the Mediterranean regions have historically been under-sampled for fish parasites. Recent records of this species in North Macedonia, Algeria or Turkey (Alas et al., 2015; Berrouk et al., 2020, 2022; Blazhekovikj-Dimovska and Stojanovski, 2022), combined with the present findings, suggest a broader distribution range than previously assumed. No specimens have yet been detected in the Iberian or Apennine peninsulas. *Ergasilus briani* was originally described by Markevich (1933) from Russia (then the USSR) and has since been documented across much of the Palearctic region. Parasitic females of this species predominantly infest various cyprinid fishes, typically residing on the inner side of the gill filaments (Alston et al., 1993). Our morphological identification of specimens of *E. briani* matched the most recent redescription by Alston et al. (1996). Additionally, molecular comparison with Chinese specimens deposited in the GenBank database [DQ107572 (18S), DQ107532 (28S); Song et al., 2008] showed high similarity (99.71% for 18S, 98.51% for 28S).

*Ergasilus barbi,* previously confirmed only in Iraq, was now recorded at 5 localities in Turkey, with the highest abundance found on *Barbus escherichii* (Steindachner, 1897) in Kütahya (a total of 165 specimens recorded on 5 host specimens). It was originally described by Rahemo (1982) from *Arabibarbus grypus* (Heckel, 1843) in the Tigris River near Mosul, Iraq. In the same study, morphologically very similar *E. mosulensis* was also described. Ho et al. (1996) later provided a redescription due to discrepancies between their collected specimens and the original paratypes. The only difference between the two species is a presence of an outer spine on the second exopod segment of leg 1 in *E. barbi* (*vs* absence in *E. mosulensis*). Until now, *E. barbi* had not been recorded outside of Iraq. However, given that *E. mosulensis* has previously been reported in Atatürk Dam Lake, Turkey (Jawad and Öktener, 2007; Öktener et al., 2007, 2008; Öktener and Alaş, 2009; Öktener, 2021), it is plausible that both species may have expanded from Iraq. Nevertheless, comprehensive morphological and molecular analyses are recommended to reliably differentiate *E. barbi* from *E. mosulensis* and to avoid possible misidentification, especially given that no molecular data are currently available for *E. mosulensis*.

The findings of *Ergasilus rostralis* in low abundances at 2 localities in Iraq are in accordance with its previous geographical records. It was first discovered on coastal water fishes from Kerala, India on three species of grey mullet in Veli Lake (estuarine), Trivandrum and in Neendakara (estuarine), Quilon (Ho et al., 1992). Later, it was recorded from the Shatt Al-Arab River, Basrah Province, Iraq, where it is currently known to infect 20 species of both fresh and marine fish (Ho et al., 1996; Al-Daraji and Mhaisen, 2023; Mhaisen and Al-Daraji, 2023). Phylogenetic analysis revealed a close relationship with *E. barbi*, suggesting that both species may have originated in Iraq or close area. The occurrence of *E. rostralis* in India could be a secondary introduction or expansion, but molecular data from India are still lacking.

In this study, *E. lizae* was recorded only in Greece, which represents the second record of this species in the country (Ragias et al., 2005). In the Mediterranean and Middle East areas, it was previously reported from several countries. Generally, *E. lizae* is considered an almost cosmopolitan species, primarily restricted to fish hosts from the family Mugilidae, but it is not highly host-specific and may also infect other fish species such as cichlids, eels or cyprinids, especially when they occur in the same area as mullets (Paperna, 1975). *Ergasilus lizae* was first reported by Krøyer (1863) from the gills of the mullet in USA (New Orleans), although no drawings were provided. Later, Ben Hassine and Raibaut (1980) synonymised *Ergasilus nanus* Beneden, 1870 with *E. lizae* even though Kabata (1979) concluded, based on a comparison of his specimens with Roberts (1970) description, that they are distinct species. *Ergasilus lizae* was redescribed by Kabata (1992) from specimens from Australia and most recently from Mexico (Morales-Serna and Camacho-Zepeda, 2024). In our sampling, the morphology of specimens of *E. lizae* was more consistent with the former descriptions of Kabata (1992) instead of the redescription from Mexico. Moreover, some authors claim *E. lizae* to be a marine species (Morales-Serna and Camacho-Zepeda, 2024), while others report it from brackish waters (Paperna, 1975; Yalım et al., 2023) or even freshwater (Kabata, 1992). We identified *E. lizae* in the Sperchios River (near the village of Ypati), which has over 60 tributaries and forms a large delta before emptying into the Maliakos Gulf, ultimately reaching the Aegean Sea (Piria et al., 2018). In this river, which is influenced by seawater, salinity levels in the estuarine area can fluctuate, creating brackish conditions that may be suitable for *E. lizae*. In addition, El-Rashidy (1999) suggested that *E. lizae* might also represent a complex of cryptic species with similar morphology. The discrepancies among authors regarding whether it is marine or freshwater species, as well as synonymization of *E. nanus,* might actually support this hypothesis of cryptic species complex. The phylogenetic analyses revealed a close relationship between *E. lizae* and African *Ergasilus* species, including *E. kandti*, a species recorded from the upper parts of Egypt, which belongs to the Mediterranean and Middle East region. This finding could suggest a possible evolutionary or biogeographical link between African and Mediterranean species.

The new described species, *E. italicus* n. sp., is the first description of a new species of Ergasilidae in Europe in this century. Only 4 specimens were found on the gills of the endemic fish *Protochondrostoma genei*, distributed in Italy and Slovenia. Previously, only 3 known species of the genus *Ergasilus* (*E. lagunaris, E. lizae* and *E. sieboldi*) were recorded in Italy (Grandori, 1925; Aisa et al., 1983; Lui et al., 2013). Additionally, an unidentified *Ergasilus* sp. was used in the study examining mast cell responses (Dezfuli et al., 2011). However, neither morphological nor molecular data were provided to support its identification. Based on the single photo it is difficult to presume which species it might be, but it does not appear to match *E. italicus* n. sp. *Ergasilus lagunaris* was described from the Venetian Bay in 1925 (Grandori, 1925), but it was documented on a single occasion and has not been referenced since. The description of *E. lagunaris* is outdated and only based on a male specimen, which should not be a standard for describing new species of parasitic copepods. Both *E. lizae* and *E. sieboldi* from Scardovari Lagoon and Lake Trasimeno have also been recorded on a single occasion only. There is a considerable lack of scientific papers on ergasilids or lernaeids from Italy, and our discovery may indicate the potential for further new records from this area. Phylogenetic analyses confirmed the inclusion of *E. italicus* n. sp. in clade A, which includes the African *Ergasilus* species, *E. barbi, E. lizae, E. rostralis, E. yaluzangbus* and unidentified *Ergasilus* sp. from Mexico, but its closer relationship with these species was not supported.

From 2 *Neoergasilus* species previously recorded in the Mediterranean and Middle East, *N. japonicus* was recorded in this study from 14 host species of the Leuciscidae and Cyprinidae in Italy, Croatia, Turkey and Iraq. *Neoergasilus japonicus* was originally described from Taiwan in Lake Jitsugetsutan (Harada, 1930) as *Ergasilus japonicus* (Harada, 1930) from cyprinid fishes, but later transferred by Yin (1956) to the genus *Neoergasilus*. This species is native to eastern Asia, including Taiwan, China, Japan, Korea and the Russian Far East (Nagasawa and Uyeno, 2012). Since its discovery it has spread throughout the world, often introduced along with live fishes and exhibits extremely low host specificity (Suárez-Morales et al., 2010) and is currently classified as an invasive parasite (Ondračková et al., 2024, 2025). Our new geographical records in Croatia and Iraq further support the hypothesis that *N. japonicus* is readily dispersed, likely through natural water flows, fish translocations, or human-mediated activities. Present results suggest that its current distribution is likely underestimated, with a potential for even wider dissemination than currently documented. Phylogenetic reconstruction confirmed its placement in clade B with the closest relationships with ergasilids from China (Song et al., 2008). For both rDNA sequences, 2 types of genetic variants were found, which is consistent with the findings of Ondračková et al. (2019). However, no morphological differences were observed, and it might only be a case of intraspecific variability due to introduction from various places on multiple occasions. Genetic data are currently available for only two species of this genus, namely *N. japonicus*, which forms a monophyletic well-supported group within clade B, and the newly described *Neoergasilus africanus* (Fikiye et al., 2024) parasitizing *Clarias gariepinus* (Burchell, 1822) in South Africa (Fikiye et al., 2024), which has been placed at the base of an entire clade of Ergasilidae, raising doubts about the monophyly of the genus.

Only 1 species of the genus *Paraergasilus*, namely *P. longidigitus* has been found for the first time in Albania with very low abundances (3 specimens overall). In the studied areas, it was previously documented only from *Alburnus alburnus* (Linnaeus, 1758) in Turkey (Koyun et al., 2007). In the Palearctic region, it is a widely distributed species, typically inhabiting the nasal cavity of fish. This specific localization may lead to its underreporting if dissections are not conducted thoroughly. In general, the members of the genus *Paraergasilus* are known for their low host specificity, with over 20 cyprinid species identified as hosts, while some species are also capable of parasitising bivalve molluscs (Chernysheva and Purasjoki, 1991). Unfortunately, we were unable to obtain any molecular data for *P. longidigitus* due to drying off of fixed specimens during long-term storage, but the morphological examination and comparison matched the most recent study of Kvach et al. (2021).

Within Lernaeidae, only 3 species belonging to 3 genera were recorded in this study. *Lernaea cyprinacea* was found in both adult and copepodid forms in various stages of the life cycle in Portugal, Spain, Italy, Croatia, Bosnia and Herzegovina and Turkey, thereby confirming its extensive distribution in these regions and very low host specificity. It is one of the most recognized parasitic copepods and is currently regarded as a cosmopolitan species with a broad geographic distribution, spanning North and South America, Europe, Asia, Southern Africa and Australia (Avenant-Oldewage, 2012). No intraspecific variability was observed in our dataset, and all available genetic sequences of *Lernaea* species are strikingly similar. The phylogenetic analysis suggests that *L. cruciata, L. ctenopharyngodontis* and *L. polymorpha* are not genetically distinct from *L. cyprinacea*. This observation raises doubts about the distinctiveness of these *Lernaea* species, suggesting that they may not be separate species, but rather variations of a single, widely distributed species, *L. cyprinacea*, with greater intraspecific genetic variability. Another possible explanation is that the rDNA genetic markers, commonly used for phylogenetic analysis and delimitation of species of Lernaeidae, may not provide enough information to accurately distinguish species within *Lernaea*. Moreover, several *Lernaea* species have previously been synonymized with *L. cyprinacea* (e.g., *Lernaea carassii* Tidd, 1933 and *Lernaea elegans* Leigh-Sharpe, 1925) (Harding, 1950) and multiple subspecies have also been described in the past (Yü, 1938; Hu, 1948; Gnanamuthu, 1951).

In the Mediterranean and Middle Eastern regions, *L. pulchella* was previously recorded only from Italy, Iraq and Turkey and now it was found as adults on 20 different host species across Italy, Bosnia and Herzegovina, Greece and Turkey. It is presumably the only *Lamproglena* species occurring in Europe, with a distribution extending across most of the Palearctic region, reaching as far as Iraq (Rahemo and Ami, 2013), and with its most recent discovery in North Macedonia (Blazhekovikj-Dimovska and Stojanovski, 2024). Even though this species appears to be quite uniform in its morphological traits, several discrepancies between the original description and later records have been observed. The molecular analysis of *L. pulchella* revealed the presence of 4 distinct but closely related 28S rDNA genetic variants, suggesting that this taxon may represent a complex of cryptic species rather than a single species. Despite genetic variation, individuals exhibited highly similar morphological traits, with only minor differences observed in their overall size. Specimens were collected from multiple locations and fish hosts, with main differences between sequences from Turkey and European countries. This may highlight the necessity for further studies to clarify the taxonomic status of *L. pulchella* and potentially new species might have to be described in the future. Since no distinct morphological traits were observed for the description of new species, we identify all of them as *L. pulchella*.

The newly described species, *P. zahrziensis* n. sp., is the third species of genus *Pseudolamproglena* recorded from Iraq, following *P. annulata* and *P. boxshalli*, and the fifth within the genus, distinguished by several unique morphological features. The genus *Pseudolamproglena* appears to be geographically restricted to the Middle and Far East, but it has rarely been reported outside of Iraq since its initial description and does not seem to exhibit high prevalence or dispersal tendencies. The genus *Pseudolamproglena* is distinguished from *Lamproglena* mainly by distinctive somatic segmentation and the structure and armature of the maxillae and maxillipeds (Boxshall, 1976). The molecular data obtained in this study represent the first genetic records for this genus. Molecular analysis placed *P. zahrziensis* n. sp. firmly within the family Lernaeidae, clustering closely with sequences of *L. pulchella* from the Middle East and Mediterranean, as well as *L. hoi* from South Africa. The close phylogenetic relationship between *P. zahrziensis* n. sp. and species of *Lamproglena* suggests that the members of *Pseudolamproglena* may not represent a distinct genus, but instead could be highly modified copepods within the *Lamproglena* clade. However, further molecular data, particularly from additional *Pseudolamproglena* species and other members of the *Lamproglena* clade, are necessary to resolve this issue definitively. Until more comprehensive genetic analyses are available, the status of *Pseudolamproglena* as a separate genus remains uncertain. It is possible that future studies will reveal that *Pseudolamproglena* should be synonymized with *Lamproglena*.

## Conclusion

This study provides new insights into the distribution and taxonomy of parasitic copepods in the Mediterranean and the Middle East, expanding known host ranges and identifying new localities for several species. The findings underscore the importance of continued research on these parasites, as their diversity and biogeographical patterns remain underexplored. Molecular analyses revealed significant phylogenetic challenges within the families Ergasilidae and Lernaeidae, particularly regarding the polyphyletic nature of *Ergasilus* and the uncertain species boundaries in the genera *Lernaea* and *Lamproglena*. The study suggests that taxonomic revisions are necessary, potentially requiring the reclassification of some genera. Comprehensive morphological and molecular studies are needed to resolve taxonomic uncertainties and assess the true diversity and distribution of these parasitic copepods.

## Supporting information

Míč et al. supplementary material Míč et al. supplementary material

## Data Availability

Type and voucher specimens were deposited in the Institute of Parasitology, Czech Academy of Sciences, České Budějovice, Czech Republic (accession codes IPCAS Cr-40 and IPCAS Cr-41). The sequences produced in this study were deposited in GenBank of NCBI at https://www.ncbi.nlm.nih.gov/ (accession codes PX000625-PX000680; PV988324-PV988349).

## Supplementary material

The supplementary material for this article can be found at https://doi.org/10.1017/S0031182025100814.

## References

[ref1] Aisa E, Desideri L, Guerrieri P and Bonelli P (1983) Further information on fish parasites from Lake Trasimeno. II. Observations on the population of *Scardinius erythrophthalmus* L. *Bollettino Della Societa Italiana di Biologia Sperimentale* 59(9), 1383–1389. 6414493

[ref2] Alas A, Öktener A and Türker DÇ (2015) Review of parasitic copepods recorded in fish from Turkey. *Transylvanian Review of Systematical and Ecological Research* 17(1), 39–62.

[ref3] Al-Daraji S and Mhaisen F (2023) Effect of the infection of the mugilid fish *Planiliza subviridis* with the copepod *Ergasilus rostralis*: A haematological study. *Journal of Biological Studies* 6(3), 264–272.

[ref4] Ali AH and Adday TK (2019) Description of a new species of *Dermoergasilus* Ho & Do, 1982 (Copepoda: Ergasilidae) from the redbelly tilapia *Coptodon zillii* (Gervais)(Perciformes: Cichlidae) in Basrah, southern Iraq. *Systematic Parasitology* 96, 715–722. 31515681 10.1007/s11230-019-09882-8

[ref5] Almeida D, Almodóvar A, Nicola GG and Elvira B (2008) Fluctuating asymmetry, abnormalities and parasitism as indicators of environmental stress in cultured stocks of goldfish and carp. *Aquaculture* 279, 120–125.

[ref6] Al-Mosawi MH and Adday TK (2024) First Record of *Mugilicola bulbosa* Tripathi, 1960 (Ergasilidae: Cyclopoida) on the Gills of Greenback Mullet *Planiliza subviridis* (Valenciennes) from Shatt Al-Arab River, Southern Iraq. *Basrah Journal of Agricultural Sciences* 37(2), 67–77.

[ref7] Al-Nasiri FS, Ho JS and Mhaisen FT (2012) *Pseudolamproglena boxshalli* sp. n. (Lernaeidae: Lamprogleninae) parasitic on gills of *Cyprinion macrostomum* (Teleostei: Cyprinidae) from the Tigris River, Iraq. *Folia Parasitologica* 59(4), 308. 23327013 10.14411/fp.2012.043

[ref8] Al-Nasiri FS, Mhaisen FT and Al-Nasiri SK (2001) First record of the ectoparasitic crustacean *Lernaea oryzophila* Monod, 1932 (Copepoda: Lernaeidae) in Iraq on the common carp Cyprinus carpio. *Al-Mustansiriyah Journal of Science* 12(7), 643–648.

[ref9] Al-Sahlany BA, Adday TK and Ali AH (2024) A New *Ergasilus* Nordmann, 1832 Species (Copepoda: Cyclopoida, Ergasilidae) from Gills of Two Freshwater Fishes at Al-Gharraf River, Southern Iraq. *Egyptian Journal of Aquatic Biology and Fisheries* 28(6), 633–643.

[ref10] Alston S, Boxshall GA and Lewis JW (1993) A redescription of adult females of *Ergasilus briani* Markewitsch, 1933 (Copepoda: Poecilostomatoida). *Systematic parasitology* 24, 217–227.

[ref11] Alston S, Boxshall GA and Lewis JW (1996) The life-cycle of *Ergasilus briani* Markewitsch, 1993 (Copepoda: Poecilostomatoida). *Systematic Parasitology* 35, 79–110.

[ref12] Amado Pinto da Motta MA, Falavigna da rocha CE, Piasecki W, Al-Daraji SA and Mhaisen FT (2001) Copepods of the family Ergasilidae (Poecilostomatoida) parasitic on fishes from Khor Al-Zubair lagoon, Iraq. *Hydrobiologia* 459, 213–221.

[ref13] Avenant-Oldewage A (2012) *Lernaea cyprinacea* and related species. In Woo PTK and Buchmann K (eds.), *Fish parasites pathobiology and protection*. UK: CAB International Press, pp. 337–349.

[ref14] Bao M, Costal D, Garci ME, Pascual S and Hastie LC (2016) Sea lice (*Lepeophtheirus salmonis*) and anchor worms (*Lernaea cyprinacea*) found on sea trout (*Salmo trutta*) in the River Minho catchment, an important area for conservation in NW Spain. *Aquatic Conservation: Marine and Freshwater Ecosystems* 26(2), 386–391.

[ref15] Ben Hassine K (1983) Les Copepodes parasites de Poissons Mugilidae en Mediterranee occidentale. pp. 452, (Doctoral dissertation, Université Montpellier II, France).

[ref16] Ben Hassine K and Raibaut A (1980) Sur la synonymie de *Ergasilus lizae* Krøyer, 1863 et de *Ergasilus nanus* Van Beneden, 1870 (Copepoda, Ergasilidae). *Bulletin Officiel National de la Pêche de Tunis* 4(2), 209–213. [in French]

[ref17] Benovics M, Desdevises Y, Vukić J, Šanda R and Šimková A (2018) The phylogenetic relationships and species richness of host-specific *Dactylogyrus* parasites shaped by the biogeography of Balkan cyprinids. *Scientific Reports* 8(1), 13006. 30158640 10.1038/s41598-018-31382-w PMC6115452

[ref18] Benovics M, Francová K, Volta P, Dlapka V and Šimková A (2021c) Helminth communities of endemic cyprinoids of the Apennine Peninsula, with remarks on ectoparasitic monogeneans, and a description of four new *Dactylogyrus* Diesing, 1850 species. *Parasitology* 148(8), 1003–1018. 33843503 10.1017/S0031182021000615 PMC10090784

[ref19] Benovics M, Koubková B, Civáňová K, Rahmouni I, Čermáková K and Šimková A (2021b) Diversity and phylogeny of *Paradiplozoon* species (Monogenea: Diplozoidae) parasitising endemic cyprinoids in the peri-Mediterranean area, with a description of three new *Paradiplozoon* species. *Parasitology Research* 120, 481–496. 33409627 10.1007/s00436-020-06982-z

[ref20] Benovics M, Nejat F, Abdoli A and Šimková A (2021a) Molecular and morphological phylogeny of host-specific *Dactylogyrus* parasites (Monogenea) sheds new light on the puzzling Middle Eastern origin of European and African lineages. *Parasites and Vectors* 14, 1–15. 34289869 10.1186/s13071-021-04863-7 PMC8293574

[ref21] Benovics M, Rahmouni C, Řehulková E, Nejat F and Šimková A (2024) Uncovering the monogenean species diversity of cyprinoid fish in Iraq using an integrative approach. *Parasitology* 151(2), 220–246. 38116665 10.1017/S0031182023001348 PMC10941050

[ref22] Benovics M, Vukić J, Šanda R, Nejat F, Charmpila EA, Buj I, Shumka S, Porcelloti S, Tarkan SA, Aksu S, Emiroğlu O and Šimková A (2023) Monogeneans and chubs: Ancient host-parasite system under the looking glass. *Molecular Phylogenetics & Evolution* 179, 107667. 36400419 10.1016/j.ympev.2022.107667

[ref23] Benovics M, Vukić J, Šanda R, Rahmouni I and Šimková A (2020) Disentangling the evolutionary history of peri-Mediterranean cyprinids using host-specific gill monogeneans. *International Journal for Parasitology* 50(12), 969–984. 32619430 10.1016/j.ijpara.2020.05.007

[ref24] Berrouk H, Sid A, Lahoual A, Sahtout F, Kaouachi N and Boualleg C (2022) Effect of parasitic copepods on the length-weight relationship and the condition factor of crucian carp (*Carassius carassius*) in the Beni-Haroun Dam, Mila City, Northeast Algeria. *Animal Research International* 19(3), 4625–4633.

[ref25] Berrouk H, Tolba M, Touarfia M and Boualleg C (2020) A study of parasitic copepod infesting two freshwater fish populations (*Cyprinus carpio* and *Abramis brama*) from Beni-Haroun Dam (Mila) North-East of Algeria. *Annual Research & Review in Biology* 34(3), 1–11.

[ref26] Blazhekovikj-Dimovska D and Stojanovski S (2022) Parasitic Copepods on Common Carp (*Cyprinus carpio*, L. 1758) from Gradche Reservoir (Macedonia). *Journal of Fisheries Science* 4(1), 61–67.

[ref27] Blazhekovikj-Dimovska D and Stojanovski S (2024) Occurrence of *Lamproglena pulchella* (Nordmann, 1832) (Copepoda: Lernaeidae) in some cyprinid fish from Ohrid Lake (Macedonia). *JASRD Journal of Agriculture and Sustainable Rural Development* 2(3-4), 61–64.

[ref28] Boucenna I, Khelifi N, Boualleg C, Allalgua A and Bensouilah M (2018) L’infestation de *Luciobarbus callensis* (Cyprinidés) par les copépodes parasites dans le barrage Foum El Khanga (Souk-Ahras, Algérie). *Bulletin de la Société Zoologique de France* 143(4), 201–214.

[ref29] Boxshall GA (1976) A new genus and two new species of copepod parasitic on freshwater fishes. *Bulletin of the British Museum Natural History, (Zoology)* 30(6), 209–216.

[ref30] Boxshall GA and Defaye D (2008) Global diversity of copepods (Crustacea: Copepoda) in freshwater. *Hydrobiologia* 595, 195–207.

[ref31] Boxshall GA and Hayes P (2019) Biodiversity and taxonomy of the parasitic Crustacea. In Smit N, Bruce N and Hadfield K (eds.), *Parasitic Crustacea*. Cham: Springer Nature, Vol. 3, 73–134.

[ref32] Bush AO, Lafferty KD, Lotz JM and Shostak AW (1997) Parasitology meets ecology on its own terms: Margolis *et al.* revisited. *The Journal of Parasitology* 83(4), 575–583. 9267395

[ref33] Chernysheva NB and Purasjoki KJ (1991) A redescription of *Paraergasilus rylovi* Markevich, 1937 (Copepoda, Ergasilidae). *Systematic Parasitology* 20(3), 165–171.

[ref34] Cuttelod A, García N, Malak DA, Temple HJ and Katariya V (2009) The Mediterranean: A biodiversity hotspot under threat. *Wildlife in a Changing World–an Analysis of the 2008 IUCN Red List of Threatened Species* 89(9), 1–4.

[ref35] Dezfuli BS, Giari L, Lui A, Lorenzoni M and Noga EJ (2011) Mast cell responses to *Ergasilus* (Copepoda), a gill ectoparasite of sea bream. *Fish & Shellfish Immunology* 30(4-5), 1087–1094. 21316458 10.1016/j.fsi.2011.02.005

[ref36] El-Rashidy HH (1999). *Ergasilid copepods and grey mullet* (Doctoral dissertation, Queen Mary University of London, Great Britain).

[ref37] Fijan N (1974) *Diseases of Fish and Crustaceans*. Zagreb: Sveučilište u Zagrebu, Veterinarski fakultet. Sveučilišna naklada Liber, 105. [in Croatian]

[ref38] Fijan N (1982). *Diseases and enemies of fishes*, pp. 439-513. In: Habeković, D., Bojčić, C. (eds), Slatkovodno ribarstvo. Jugoslavenska medicinska naklada. Poslovna zajednica slatkovodnog ribarstva Jugoslavije, Ribozajednica Zagreb. 605pp. [in Croatian]

[ref39] Fikiye PP, Van As LL, Truter M, Smit NJ and Hadfield KA (2024) A new species of *Neoergasilus* Yin 1956 (Copepoda: Cyclopoida: Ergasilidae) parasitic on the catfish *Clarias gariepinus* (Burchell, 1822) (Siluriformes: Clariidae) from South Africa. *Systematic Parasitology* 101(5), 64. 39316200 10.1007/s11230-024-10189-6 PMC11422265

[ref40] Fratello B and Sabatini MA (1972) Cariologia e sistematica di *Lernaea cyprinacea* L.(Crustacea, Copepoda). *Atti della Accademia Nazionale Dei Lincei, Classe Di Scienze Fisiche, Matematiche E Naturali, Rendiconti* 53(1-2), 209–213. In Italian

[ref41] Freyhof J, Ekmekçi FG, Ali A, Khamees NR, Özuluğ M, Hamidan N, Küçük F and Smith KG (2014) Freshwater fishes. In Smith KG, Barrios, V Darwall, WRT and Numa C (eds.),*The Status and Distribution of Freshwater Biodiversity in the Eastern Mediterranean*, pp. 19–42. Malaga, Spain: IUCN.

[ref42] Freyhof J, Kaya C and Ali A (2021) A critical checklist of the inland fishes native to the Euphrates and Tigris drainages. In Jawad LA (ed.), *Tigris and Euphrates Rivers: their Environment from Headwaters to Mouth, Vol. 11. Aquatic Ecology Series*. Cham: Springer, 815–854.

[ref43] Gnanamuthu CP (1951) *Lernaea chackoensis* n. sp., a copepod parasitic on two Madras fishes. *Parasitology* 41(1), 143–147. 14911208 10.1017/s0031182000083979

[ref44] Grandori R (1925) Nuove specie di Copepodi della Laguna Veneta. *Bollettino Di Istituto Di Zoologia, Reale Universita Di Roma* 3, 38–70. [in Italian].

[ref45] Hall TA (1999) BioEdit: A user-friendly biological sequence alignment editor and analysis program for Windows 95/98/NT. *Nucleic Acids Symposium Series* 41(41), 95–98.

[ref46] Harada I (1930) Studies on freshwater fauna of Formosa (I): A new copepod species parasitic on Formosan freshwater fishes. *Journal of the Society of Tropical Agriculture* 2(1), 71–76.

[ref47] Harding JP (1950) On some species of *Lernaea* (Crustacea, Copepoda: Parasites of fresh-water fish). *Bulletin of the British Museum (Natural History), Zoology Series* 1(1), 1–27.

[ref48] Hassan ES, Mahmoud MM, Metwally AM and Mokhtar DM (2013) *Lamproglena monodi* (Copepoda: Lernaeidae), infesting gills of *Oreochromis niloticus* and *Tilapia zillii*. *Global Journal of Fisheries and Aquaculture Research* 6, 1–16.

[ref49] Hermida M, Saraiva A and Cruz C (2008) Metazoan parasite community of a European eel (*Anguilla anguilla*) population from an estuary in Portugal. *Bulletin-european Association of Fish Pathologists* 28(1), 35.

[ref50] Ho JS, Jayarajan P and Radhakrishnan S (1992) Copepods of the family Ergasilidae (Poecilostomatoida) parasitic on coastal fishes of Kerala, India. *Journal of Natural History* 26(6), 1227–1241.

[ref51] Ho JS, Khamees NR and Mhaisen FT (1996) Ergasilid copepods (Poecilostomatoida) parasitic on the mullet *Liza abu* in Iraq, with the description of a new species of *Paraergasilus* Markevich, 1937. *Systematic Parasitology* 33, 79–87.

[ref52] Hoang DT, Chernomor O, Von Haeseler A, Minh BQ and Vinh LS (2018) UFBoot2: Improving the ultrafast bootstrap approximation. *Molecular Biology and Evolution* 35(2), 518–522. 29077904 10.1093/molbev/msx281 PMC5850222

[ref53] Hu YT (1948) Studies on the parasitic copepods of China, part 3: The Far Eastern allies of *Lernaea cyprinacea* L., with a description of two new subspecies and *L. rhodei* sp. nov. *Sinensia* 19, 86–98.

[ref54] Huelsenbeck JP and Ronquist F (2001) MRBAYES: Bayesian inference of phylogenetic trees. *Bioinformatics* 17(8), 754–755. 11524383 10.1093/bioinformatics/17.8.754

[ref55] Huys R and Boxshall GA (1991) Copepod evolution. *Ray Society (Publications)* 159, 1–468.

[ref56] Jansen D, Vanhove MP, Makasa L, Vorel J, Kmentová N and Cruz-Laufer AJ (2024) Mitogenomics, phylogenetic position, and updated distribution of *Ergasilus kandti*, an ergasilid copepod parasitizing African cichlid fishes. *Hydrobiologia* 852, 1–26.

[ref57] Jawad LA and Öktener A (2007) Incidence of lordosis in the freshwater mullet, *Liza abu* (Heckel, 1843) collected from Atatürk Dam Lake, Turkey. *Annales de Biologie* 29, 105–113.

[ref58] Kabata Z (1979) Parasitic Copepoda of British Fishes. *The Ray Society* 152, 1-468.

[ref59] Kabata Z (1992) Copepoda parasitic on Australian fishes, XV. family Ergasilidae (Poecilostomatoida). *Journal of Natural History* 26(1), 47–66.

[ref60] Kalyaanamoorthy S, Minh BQ, Wong TK, Von Haeseler A and Jermiin LS (2017) ModelFinder: Fast model selection for accurate phylogenetic estimates. *Nature Methods* 14(6), 587–589. 28481363 10.1038/nmeth.4285 PMC5453245

[ref61] Katoh K, Rozewicki J and Yamada KD (2019) MAFFT online service: Multiple sequence alignment, interactive sequence choice and visualization. *Briefings in Bioinformatics* 20(4), 1160–1166. 28968734 10.1093/bib/bbx108 PMC6781576

[ref62] Koyun M, Altunel FN and Öktener A (2007) *Paraergasilus longidigitus* Yin, 1954 (Copepoda: Poecilostomatoida) infestations in the bleak, *Alburnus alburnus* Lin., 1758 from Enne dam lake. *Turkiye Parazitoloji Dergisi* 31(2), 158–161. 17594662

[ref63] Koyun M, Ulupınar M and Gül A (2015) Seasonal distribution of Metazoan Parasites on Kura Barbell (*Barbus lacerta*) in Eastern Anatolia, Turkey. *Pakistan Journal of Zoology* 47(5), 1253–1261.

[ref64] Krøyer HN (1863) Bidrag til kundskab om snyltekrebsene. Thieles Bogtrykkeri. *Naturhistorisk Tidsskrift Ser. III* 2(1/2), 75–320.

[ref65] Kuang PR (1980) A new genus of Lernaeidae (parasitic copepods) and its relation to affined genera. *Acta Zootaxonomica Sinica* 5, 124–128. In Chinese with English summary

[ref66] Kvach Y, Tkachenko MY, Seifertová M and Ondračková M (2021) Insights into the diversity, distribution and phylogeny of three ergasilid copepods (Hexanauplia: Ergasilidae) in lentic water bodies of the Morava river basin, Czech Republic. *Limnologica* 91, 125922.

[ref67] Lui A, Manera M, Giari L, Mulero V and Dezfuli BS (2013) Acidophilic granulocytes in the gills of gilthead seabream *Sparus aurata*: Evidence for their responses to a natural infection by a copepod ectoparasite. *Cell and Tissue Research* 353, 465–472. 23644766 10.1007/s00441-013-1627-5

[ref68] Macchioni F, Chelucci L, Torracca B, Prati MC and Magi M (2015) Fishes and their parasites in the water district of Massaciuccoli (Tuscany, Central Italy). *Veterinaria Italiana* 51(3), 199–203. 26455372 10.12834/VetIt.230.733.1

[ref69] Markevich AP (1933) Descrizione di due species nuove di *Ergasilus* provenienti dalla Russia (URSS). *Memorie Della Società Entomologica Italiana* 12, 129–141.

[ref70] Mhaisen F, Ali A and Adday T (2024) Checklists of Fish Species Infected with Parasites of the Genera *Lamproglena* and *Pseudolamproglena* (Copepoda: Cyclopoida: Lernaeidae) in Iraq. *Iraqi Journal of Aquaculture* 21(2), 1–16.

[ref71] Mhaisen FT and Abdul-Ameer KN (2021) Checklist of fish hosts of species of *Lernaea* Linnaeus, 1758 (Hexanauplia: Cyclopoida: Lernaeidae) in Iraq. *Biological and Applied Environmental Research* 5(1), 53–73.

[ref72] Mhaisen FT and Al-Daraji SA (2023) Checklists of Species of *Ergasilus* von Nordmann, 1832 (Copepoda: Ergasilidae) Parasitic on Fishes of Iraq. *Iraqi Journal of Aquaculture* 20(2), 211–244.

[ref73] Míč R, Řehulková E and Seifertová M (2023) Species of *Ergasilus* von Nordmann, 1832 (Copepoda: Ergasilidae) from cichlid fishes in Lake Tanganyika. *Parasitology* 150(7), 579–598. 36938816 10.1017/S0031182023000239 PMC10260305

[ref74] Míč R, Řehulková E, Šimková A, Razanabolana JR and Seifertová M (2024) New species of *Dermoergasilus* Ho & Do, 1982 (Copepoda: Cyclopoida: Ergasilidae) parasitizing endemic cichlid *Paretroplus polyactis* (Bleeker) in Madagascar. *Parasitology* 151(3), 319–336. 38239098 10.1017/S0031182024000088 PMC11007281

[ref75] Mirzaei M, Khovand H and Kheirandish R (2016) The prevalence of non-indigenous parasitic copepod (*Neoergasilus japonicus*) spreads with fishes of pet trade in Kerman, Iran. *Journal of Parasitic Diseases* 40, 1283–1288. 27876931 10.1007/s12639-015-0669-x PMC5118295

[ref76] Morales-Serna FN and Camacho-Zepeda S (2024) Morphology, DNA barcoding and seasonal occurrence of *Ergasilus lizae* Krøyer, 1863 (Copepoda: Ergasilidae) parasitizing mullets from northwestern Mexico. *Systematic Parasitology* 101(5), 54. 39120762 10.1007/s11230-024-10179-8 PMC11315719

[ref77] Nagasawa K and Uyeno D (2012) Utilization of alien freshwater fishes by the parasitic copepod *Neoergasilus japonicus* (Ergasilidae) on Okinawa-jima Island, Japan, with a list of its known hosts. *Zoosymposia* 8, 81–96.

[ref78] Narciso RB, Smit NJ, Perbiche-Neves G and da Silva RJ (2024) Integrative taxonomy approach to the study of parasitic ergasilids (Cyclopoida: Ergasilidae) of fishes from the Pardo River, Brazil with a redescription of *Rhinergasilus piranhus* Boeger and Thatcher, 1988 and a molecular phylogeny for Ergasilidae. *Parasitology* 152(1), 1–24. 10.1017/S003118202400129X PMC12088924 39587408

[ref79] Nedić Z, Skenderović I and Riđanović S (2014) Skin ectoparasites of fish from the lower flow of the Sava River. *Veterinaria* 63(1-4), 45–53.

[ref80] Nejat F, Benovics M, Řehulková E, Vukić J, Šanda R, Kaya C, Tarkan AS, Abdoli A, Aksu S and Šimková A (2023) Diversity, phylogeny and intraspecific variability of *Paradiplozoon* species (Monogenea: Diplozoidae) parasitizing endemic cyprinoids in the Middle East. *Parasitology* 150(8), 705–722. 37157105 10.1017/S0031182023000446 PMC10410381

[ref81] Nejat F, Benovics M, Šanda R, Vukić J, Kaya C, Tarkan AS and Šimková A (2025) The role of the Middle East in the biogeographical dispersal of host-specific parasites: Monogeneans and their cyprinoid fish hosts. *Journal of Biogeography* 0: e70034, 1–28.

[ref82] Öktener A (2021) The parasites of fishes of the Euphrates and Tigris Rivers: Iraq and Turkey. *Tigris and Euphrates Rivers: Their Environment from Headwaters to Mouth* 11, 1419–1444.

[ref83] Öktener A and Alaş A (2009) A parasitological study of fish from the Atatürk Dam Lake, Turkey. *Bulletin of the European Association of Fish Pathologists* 29(6), 193.

[ref84] Öktener A, Ali AH and Alas A (2008) New host record, *Chalcalburnus mossulensis* (Heckel, 1843) (Teleostei; Cyprinidae) for *Ergasilus mosulensis* Rahemo, 1982 (Copepoda; Ergasilidae). *Bulletin-European Association of Fish Pathologists* 28(5), 194–197.

[ref85] Öktener A, Trilles JP and Leonardos I (2007) Five ectoparasites from Turkish fish. *Türkiye Parazitoloji Dergisi* 31(2), 154–157. 17594661

[ref86] Ondračková M, Fojtů J, Seifertová M, Kvach Y and Jurajda P (2019) Non-native parasitic copepod *Neoergasilus japonicus* (Harada, 1930) utilizes non-native fish host *Lepomis gibbosus* (L.) in the floodplain of the River Dyje (Danube basin). *Parasitology Research* 118, 57–62. 30353234 10.1007/s00436-018-6114-1

[ref87] Ondračková M, Kvach Y, Tkachenko MY, Pravdová M, Seifertová M, Bartáková V and Jurajda P (2025) The role of North American bullhead catfish as parasite reservoirs in central European fishing grounds. *Aquaculture* 599, 742100.

[ref88] Ondračková M, Tkachenko MY, Vetešník L, Hronek J and Janáč M (2024) Distribution and host range of a highly invasive parasitic copepod. *Hydrobiologia* 852, 2221–2239.

[ref89] Paperna I (1964) Parasitic Crustacea (Copepoda and Branchiura) from inland water fishes of Israel. *Israel Journal of Ecology and Evolution* 13(2), 58–68.

[ref90] Paperna I (1975) Parasites and diseases of the grey mullet (Mugilidae) with special reference to the seas of the Near East. *Aquaculture* 5(1), 65–80.

[ref91] Pazooki J and Masoumian M (2012) Synopsis of the parasites in Iranian freshwater fishes. *Iranian Journal of Fisheries Sciences* 11(3), 570–589.

[ref92] Piasecki W, Khamees NR and Mhaisen FT (1991) A new species of *Mugilicola* Tripathi, 1960 (Crustacea, Copepoda, Therodamasidae) parasitic on Iraqi fish. *Acta Ichthyologica Et Piscatoria* 21(2), 143–151.

[ref93] Piria M, Simonović P, Kalogianni E, Vardakas L, Koutsikos N, Zanella D, Ristovska M, Apostolou A, Adrović A, Mrdak D, Tarkan AS, Miloševič D, Zanella LN, Bakiu R, Ekmekçi FG, Povž M, Korro K, Nikolić V, Škrijelj R, Kostov V, Gregori A and Joy MK (2018) Alien freshwater fish species in the Balkans—Vectors and pathways of introduction. *Fish and Fisheries* 19(1), 138–169.

[ref94] Pleijel F, Jondelius U, Norlinder E, Nygren A, Oxelman B, Schander C, Sundberg P and Thollesson M (2008) Phylogenies without roots? A plea for the use of vouchers in molecular phylogenetic studies. *Molecular Phylogenetics & Evolution* 48(1), 369–371. 18424089 10.1016/j.ympev.2008.03.024

[ref95] Ragias V, Athanassopoulou F and Sinis A (2005) Parasites of Mugilidae spp. reared under semi-intensive and intensive conditions in Greece. *Bulletin of the European Association of Fish Pathologists* 25(3), 107–113.

[ref96] Rahemo ZI (1982) Two new species of *Ergasilus* (Copepoda: Cyclopoida) from the gills of two Iraqi freshwater fishes. *Bulletin of Basrah Natural History Museum* 5, 39–59.

[ref97] Rahemo ZI and Ami SN (2013) Studies on the Fresh Water Fish (Bizz), Barbus Esocinus Caught from Mosul Dam Lake, Iraq. *Science Journal of University of Zakho* 1(2), 692–698.

[ref98] Rahmouni C, Seifertová M, Benovics M and Šimková A (2023) Diversity and phylogeny of *Gyrodactylus* spp.(Monogenea: Gyrodactylidae) across the Strait of Gibraltar: Parasite speciation and historical biogeography of West Mediterranean cyprinid hosts. *Diversity* 15(11), 1152.

[ref99] Rambaut A (2016) FigTree v1.4.3. http://tree.bio.ed.ac.uk/software/figtree/ (accessed 14 February 2025).

[ref100] Roberts LS (1970) Ergasilus (Copepoda: Cyclopoida): Revision and key to species in North America. *Transactions of the American Microscopical Society* 89(1), 134–161.

[ref101] Saraiva A and Valente ACN (1988) Black spot disease and *Lernaea* sp. infestation on *Leuciscus cephalus* L.(Pisces: Cyprinidae) in Portugal. *Bulletin of the European Association of Fish Pathologists* 8(1), 7–8.

[ref102] Scholz T, Vanhove MP, Smit N, Jayasundera Z and Gelnar M (Eds.) (2018) *A Guide to the Parasites of African Freshwater Fishes*. (Vol. 18, 9–13) Brussels: Royal Belgian Institute of Natural Sciences.

[ref103] Šimková A, Benovics M, Rahmouni I and Vukić J (2017) Host-specific *Dactylogyrus* parasites revealing new insights on the historical biogeography of NorthWest African and Iberian cyprinid fish. *Parasites and Vectors* 10, 1–16. 29183392 10.1186/s13071-017-2521-x PMC5706372

[ref104] Simon Vicente F, Ramajo V and Encinas A (1973) Fauna parasitaria de peces españoles de agua dulce: *Allocreadium isoporum* (Trematoda: Allocreadidae). *Lernaea Esocina; L. Cyprinacea, Revista Ibe´rica de Parasitologi´a* 33, 633–647.

[ref105] Skenderović I, Adrović A, Hajdarević E, Hadžiahmetović Jurida E, Čekmić M and Bajrić A (2015) Parazitski rakovi (Crustacea) ciprinidnih riba iz hidroakumilacije Modrac. Zbornik radova, Naučna konferencija, Lukavac. [Parasitic crustaceans (Crustacea) of cyprinid fish from the Modrac hydroaccumulation. Proceedings, Scientific Conference, Lukavac], 669–676. [In Bosnian]

[ref106] Skenderović I, Adrović A, Jazić A, Zuko A and Hadzimustafic E (2021) Review of freshwater fish parasitofauna of Bosnia and Herzegovina. *Biologia* 76, 475–515.

[ref107] Song Y, Wang GT, Yao WJ, Gao Q and Nie P (2008) Phylogeny of freshwater parasitic copepods in the Ergasilidae (Copepoda: Poecilostomatoida) based on 18S and 28S rDNA sequences. *Parasitology Research* 102, 299–306. 17940799 10.1007/s00436-007-0764-8

[ref108] Soylu E and Soylu MP (2012) First record of the nonindigenous parasitic copepod *Neoergasilus japonicus* (Harada, 1930) in Turkey. *Turkish Journal of Zoology* 36(5), 662–667.

[ref109] Stamou G, Kourkoutmani P and Michaloudi E (2022) The Inland Cladocera and Copepoda Fauna in Greece. *Diversity* 14(11), 997.

[ref110] Suárez-Morales E, Paredes-Trujillo A and González-Solís D (2010) The introduced Asian parasitic copepod *Neoergasilus japonicus* (Harada) (Cyclopoida: Ergasilidae) from endangered cichlid teleosts in Mexico. *Zoological Science* 27(11), 851–855. 21039123 10.2108/zsj.27.851

[ref111] Tamura K, Stecher G and Kumar S (2021) MEGA11: Molecular evolutionary genetics analysis version 11. *Molecular Biology and Evolution* 38(7), 3022–3027. 33892491 10.1093/molbev/msab120 PMC8233496

[ref112] Tomašec I (1953). *Diseases of freshwater fishes and crustaceans.* Zagreb: Jugoslavenska Akademija Znanosti i Umjetnosti, pp. 76.

[ref113] Trifinopoulos J, Nguyen LT, von Haeseler A and Minh BQ (2016) W-IQ-TREE: A fast online phylogenetic tool for maximum likelihood analysis. *Nucleic Acids Research* 44(W1), W232–W235. 27084950 10.1093/nar/gkw256 PMC4987875

[ref114] Vagianou S, Athanassopoulou F, Ragias V, Di Cave D, Leontides L and Golomazou E (2006) Prevalence and pathology of ectoparasites of Mediterranean Sea bream and sea bass reared under different environmental and aquaculture conditions. *The Israeli Journal of Aquaculture – Bamidgeh* 58, 78–88.

[ref115] Yalım FB, Emre N, Emre Y and Kaymak N (2023) Influence of the host sex, size, and season on *Ergasilus lizae* infestation of Thicklip Grey Mullet (*Chelon labrosus*, L., 1758) in Beymelek Lagoon Lake (Antalya, Türkiye). *Journal of Limnology and Freshwater Fisheries Research* 9(3), 147–153.

[ref116] Yin WY (1956) Studies on the Ergasilidae (parasitic Copepoda) from the freshwater fishes of China. *Acta Hydrobiologica Sinica/Shuisheng Shengwu Xuebao* 2, 209–270, pls.1–18. in Chinese with English summary

[ref117] Yü SC (1938) Some parasitic Copepoda from fresh-water fishes of China. *Bulletin of the Fan Memorial Institute of Biology, Zoology* 8(2), 105–114.

[ref118] Zarfdjian MH and Economidis PS (1989) Listes provisoires des rotifères, cladocéres et copépodes des eaux continentales grecques (Provisional lists of rotifers, Cladocera and copepods from continental waters of Greece). *Biologia Gallo-hellenica* 15, 129–146. [in French]

