# Supplementary material for: Diversity and phylogeny of parasitic copepods of freshwater fishes from the Mediterranean and the Middle East
Source: Parasitology. 2025 Sep 10;152(12):1193–220. doi: 10.1017/S0031182025100814 (PMC12921258; doi:10.1017/S0031182025100814)
Supplement: Míč et al. supplementary material [file S0031182025100814sup001.docx]

SUPPLEMENTARY MATERIAL

**Supplementary Figure S1: Map of all collection localities in the Mediterranean and the Middle East (green = parasitic copepods present, red = no parasitic copepods found)**

**
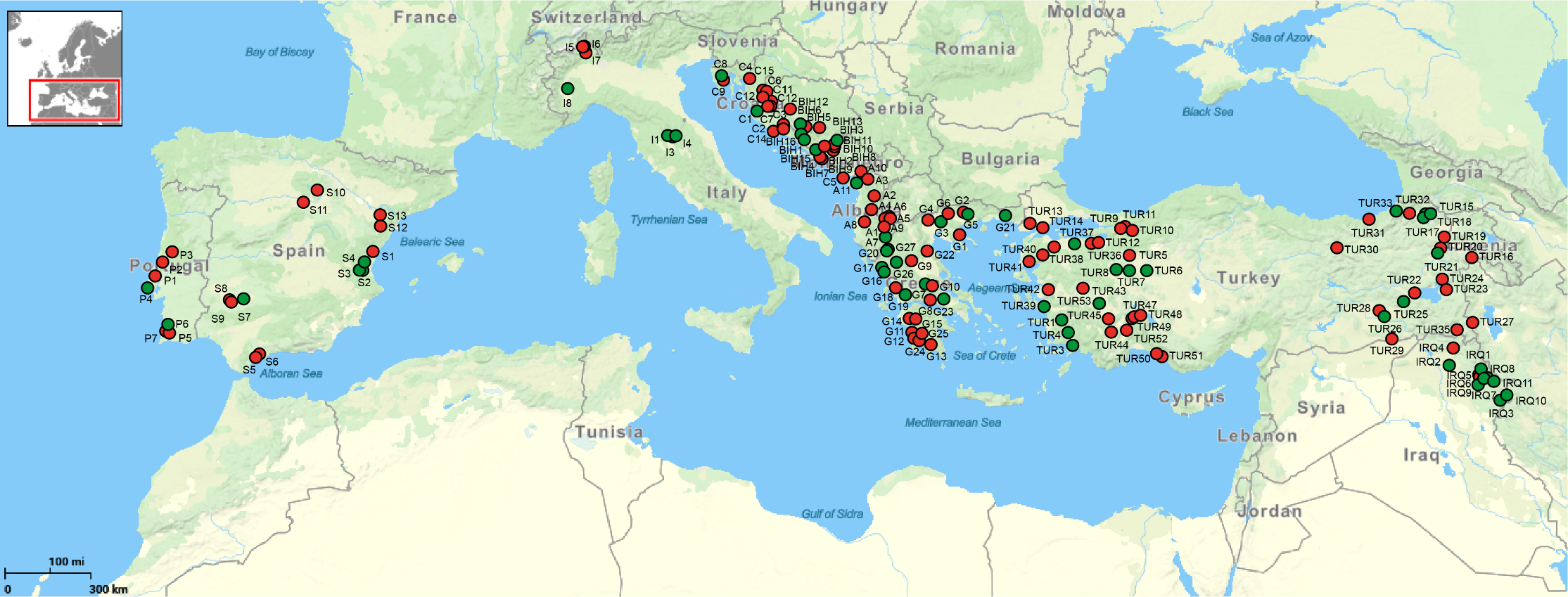
**

**Supplementary Table S1:** Checklist of parasitic copepods from families Ergasilidae and Lernaeidae including host species, host family and locality of collection in the Mediterranean and the Middle East. New records are highlighted in bold.

| **Parasite species** | **Host species** | **Host family** | **Locality** | **Country** | **Reference** |
| --- | --- | --- | --- | --- | --- |
| **Ergasilidae Burmeister, 1835** |  |  |  |  |  |
| ***Dermoergasilus* Ho & Do, 1982** |  |  |  |  |  |
| *Dermoergasilus amplectens* (Dogiel & Akhmerov, 1952) | *Planiliza abu* | Mugilidae | Al-Khazir River, Ninevah Province | Iraq | Mhaisen *et al*. (2017) |
| *Dermoergasilus cichlidus* Ali & Adday, 2019 | *Coptodon zillii* | Cichlidae | Shatt Al-Arab River, Al-Hartha District | Iraq | Ali & Adday (2019) |
|  |  |  | Pond of Marine Sciences Centre, Basrah | Iraq | Ali & Adday (2019) |
| *Dermoergasilus varicoleus* Ho *et al.* 1996 | *Alburnus sellal* | Leuciscidae | Some drainage networks in Babylon Province | Iraq | Mhaisen *et al*. (2017) |
|  |  |  | Garmat Ali River | Iraq | Mhaisen *et al*. (2017) |
|  | *Carasobarbus luteus* | Cyprinidae | Al-Husainia Creek in Karbala Province | Iraq | Al-Saadi *et al*. (2010) |
|  | *Cyprinus carpio* | Cyprinidae | Garmat Ali River | Iraq | Mhaisen *et al*. (2017) |
|  | *Leuciscus vorax* | Leuciscidae | Garmat Ali River | Iraq | Mhaisen *et al*. (2017) |
|  | *Mesopotamichthys sharpeyi* | Cyprinidae | Garmat Ali River | Iraq | Mhaisen *et al*. (2017) |
|  | *Planiliza abu* | Mugilidae | Shatt Al-Arab River | Iraq | Khamees & Mhaisen (1995), Ho *et al*. (1996) |
|  |  |  | marine waters, Khor Al-Zubair | Iraq | Amado *et al*. (2001) |
|  |  |  | Some drainage networks in Babylon Province | Iraq | Mhaisen *et al*. (2017) |
|  |  |  | Garmat Ali River | Iraq | Mhaisen *et al*. (2017) |
|  |  |  | Euphrates River, Al-Anbar Province | Iraq | Mhaisen *et al*. (2017) |
|  | *Planiliza subviridis* | Mugilidae | Garmat Ali River | Iraq | Mhaisen *et al*. (2017) |
|  | *Silurus triostegus* | Siluridae | Al-Hammar Marsh in Basrah Province | Iraq | Jori (2006) |
| *Dermoergasilus* sp. | *Cyprinus carpio* | Cyprinidae | Pond of Marine Sciences Centre, Basrah | Iraq | Ahmed & Ali (2013) |
| ***Ergasilus* von Nordmann, 1832** |  |  |  |  |  |
| *Ergasilus barbi* Rahemo, 1982 | *Arabibarbus grypus* | Cyprinidae | Tigris River, Nineveh Province | Iraq | Rahemo (1982), Mhaisen & Al-Daraji (2023) |
|  |  |  | Dokan Lake | Iraq | Abdullah & Rasheed (2004), Mhaisen & Al-Daraji (2023) |
|  | ***Barbus escherichii*** | **Cyprinidae** | **Kütahya** | **Turkey** | **present study** |
|  | ***Barbus* sp. Tahtali** | **Cyprinidae** | **Sasal stream, Kuner** | **Turkey** | **present study** |
|  | ***Capoeta aydinensis*** | **Cyprinidae** | **Çine River, near Çiftlikköy** | **Turkey** | **present study** |
|  | *Carasobarbus luteus* | Cyprinidae | Greater Zab River | Iraq | Abdullah (2002), Abdullah & Mhaisen (2003), Mhaisen & Al-Daraji (2023) |
|  |  |  | Lesser Zab River | Iraq | Abdullah & Mhaisen (2006) |
|  |  |  | fish ponds, Nineveh Province | Iraq | Mhaisen & Al-Daraji (2023) |
|  |  |  | Al-Qadisiya Dam Lake | Iraq | Balasem *et al.* (2003) |
|  |  |  | Euphrates River, Karbala Province | Iraq | Al-Saadi (2007), Al-Saadi *et al.*(2010) |
|  |  |  | Euphrates River, Al-Anbar Province | Iraq | Al-Salmany (2022) |
|  | *Carassius auratus* | Cyprinidae | Diyala River | Iraq | Mhaisen & Al-Daraji (2023) |
|  | *Carassius carassius* | Cyprinidae | Euphrates River, Al-Anbar Province | Iraq | Al-Salmany (2022) |
|  | ***Chondrostoma angorense*** | **Leuciscidae** | **Çifteler** | **Turkey** | **present study** |
|  |  |  | **Seyitgazi** | **Turkey** | **present study** |
|  | *Cyprinion macrostomus* | Cyprinidae | Greater Zab River | Iraq | Mhaisen & Al-Daraji (2023) |
|  |  |  | Dokan Lake | Iraq | Abdullah & Rasheed (2004), Mhaisen & Al-Daraji (2023) |
|  |  |  | Bahdinan River, Erbil Province | Iraq | Bilal & Abdullah (2008), Mhaisen & Al-Daraji (2023) |
|  |  |  | Euphrates River, Al-Anbar Province | Iraq | Mhaisen & Al-Daraji (2023) |
|  | *Cyprinus carpio* | Cyprinidae | fish ponds, Erbil Province | Iraq | Abdullah (2004) |
|  |  |  | Euphrates River, Karbala Province | Iraq | Al-Saadi (2007), Al-Saadi *et al.*(2010) |
|  |  |  | Diyala River | Iraq | Mhaisen & Al-Daraji (2023) |
|  | *Glyptothorax* sp. | Sisoridae | Greater Zab River | Iraq | Mhaisen & Al-Daraji (2023) |
|  | *Leuciscus vorax* | Leuciscidae | Euphrates River, Karbala Province | Iraq | Al-Saadi (2007), Al-Saadi *et al.*(2010) |
|  | *Luciobarbus esocinus* | Cyprinidae | Dokan Lake | Iraq | Abdullah & Rasheed (2004), Mhaisen & Al-Daraji (2023) |
|  | *Luciobarbus schejch* | Cyprinidae | Bahdinan River, Erbil Province | Iraq | Bilal & Abdullah (2008), Mhaisen & Al-Daraji (2023) |
|  |  |  | Greater Zab River | Iraq | Mhaisen & Al-Daraji (2023) |
|  |  |  | Bahdinan River, Erbil Province | Iraq | Bilal & Abdullah (2008), Mhaisen & Al-Daraji (2023) |
|  | *Planiliza abu* | Mugilidae | fish ponds, Nineveh Province | Iraq | Mhaisen & Al-Daraji (2023) |
|  |  |  | Greater Zab River | Iraq | Abdullah (2002) |
|  |  |  | Some drainage networks in Baghdad Province | Iraq | Balasem *et al*. (2002b) |
|  |  |  | Some drainage networks in Babylon Province | Iraq | Mhaisen & Al-Daraji (2023) |
|  |  |  | Lesser Zab River | Iraq | Abdullah & Mhaisen (2006) |
|  |  |  | Greater Zab River | Iraq | Abdullah & Mhaisen (2011) |
|  |  |  | Euphrates River, Karbala Province | Iraq | Al-Saadi (2007), Al-Saadi *et al.*(2010, 2011) |
|  |  |  | fish ponds and farms, Babylon Province | Iraq | Al-Dulaimi *et al*. (2006) |
|  |  |  | fish ponds, Baghdad Province | Iraq | Al-Nasiri *et al.* (2003) |
|  |  |  | Diyala River | Iraq | Mhaisen & Al-Daraji (2023) |
|  |  |  | Euphrates River, Al-Anbar Province | Iraq | Al-Salmany (2022) |
|  | *Silurus triostegus* | Siluridae | Greater Zab River | Iraq | Abdullah (2002) |
|  |  |  | Lesser Zab River | Iraq | Abdullah & Mhaisen (2006) |
|  | *Squalius lepidus* | Leuciscidae | Greater Zab River | Iraq | Mhaisen & Al-Daraji (2023) |
|  | *Squalius spurius* | Leuciscidae | Greater Zab River | Iraq | Mhaisen & Al-Daraji (2023) |
| *Ergasilus boleophthalmi* Adday & Ali, 2011 | *Bathygobius fuscus* | Gobionidae | Shatt Al-Basrah Canal | Iraq | Adday & Ali (2011) |
|  | *Boleophthalmus dussumieri* | Oxudercidae | Shatt Al-Basrah Canal | Iraq | Adday & Ali (2011) |
| *Ergasilus briani* Markevich, 1932 | *Abramis brama* | Leuciscidae | Beni-Haroun dam, Mila | Algeria | Berrouk *et al.* (2019) |
|  | ***Alburnoides fasciatus*** | **Leuciscidae** | **inflow of Iyidere, Iyidere basin** | **Turkey** | **present study** |
|  | ***Alburnus neretvae*** | **Leuciscidae** | **Mušnica, Artovac** | **Bosnia and Herzegovina** | **present study** |
|  |  |  | **Zagorje, Jabuke** | **Bosnia and Herzegovina** | **present study** |
|  | ***Alburnus scoranza*** | **Leuciscidae** | **Skadar lake, Shiroke** | **Albania** | **present study** |
|  | *Alburnus sellal* | Leuciscidae | Keban Dam Lake | Turkey | Alas *et al*. (2015) |
|  | ***Alburnus sp.* (from *Alburnus alburnus* complex)** | **Leuciscidae** | **Simav river, Karacabey** | **Turkey** | **present study** |
|  | *Carassius carassius* | Cyprinidae | Beni-Haroun dam, Mila | Algeria | Berrouk *et al.* (2018) |
|  | *Cyprinus carpio* | Cyprinidae | Gradche Reservoir | North Macedonia | Blazhekovikj-Dimovska & Stojanovski (2022) |
|  |  |  | Beni-Haroun dam, Mila | Algeria | Berrouk *et al.* (2019) |
|  | ***Gobio* *artvinicus*** | **Gobionidae** | **west of Ardanuç, Cehennem/Şuat/Köprüler stream, Çoruh basin** | **Turkey** | **present study** |
|  | ***Leucos aula*** | **Leuciscidae** | **Grabovač reservoir, Croatia** | **Croatia** | **present study** |
|  | ***Leucos basak*** | **Leuciscidae** | **Krenica lake, Drinovci** | **Bosnia and Herzegovina** | **present study** |
|  | ***Leucos ylikiensis*** | **Leuciscidae** | **Yliky Lake** | **Greece** | **present study** |
|  | *Luciobarbus callensis* | Cyprinidae | Beni-Haroun dam, Mila | Algeria | Berrouk *et al.* (2018) |
|  |  |  | Foum El Kangha | Algeria | Boucenna *et al*. (2018) |
|  | ***Squalius tenellus*** | **Leuciscidae** | **Šujica, Duvansko Polje** | **Bosnia and Herzegovina** | **present study** |
| *Ergasilus egyptiacus* Abdel-Hady, Bayoumy & Osman, 2008 | *Coptodon zillii* | Cichlidae | Lake Temsah | Egypt | Abdel-Hady *et al*. (2008) |
| *Ergasilus fryeri* Paperna, 1964 | *Anguilla anguilla* | Anguillidae | coastal plain streams | Israel | Paperna (1964) |
|  |  |  | Bafa Lake | Turkey | Altunel (1979) |
|  | *Chelon auratus* | Mugilidae | NA | Israel | Paperna (1971) |
|  | *Chelon ramada* | Mugilidae | coastal plain streams | Israel | Paperna (1964) |
|  |  |  | fish ponds | Israel | Lahav & Sarig (1967) |
|  | *Coptodon zillii* | Cichlidae | coastal plain streams | Israel | Paperna (1964) |
|  |  |  | fish ponds | Israel | Paperna & Lahav (1971) |
|  |  |  | Yarkon River | Israel | Paperna & Lahav (1971) |
|  | *Cyprinus carpio* | Cyprinidae | fish ponds | Israel | Lahav & Sarig (1967) |
|  | *Mugil cephalus* | Mugilidae | coastal plain streams | Israel | Paperna (1964) |
|  |  |  | fish ponds | Israel | Lahav & Sarig (1967) |
|  | *Oreochromis aureus* | Cichlidae | coastal plain streams | Israel | Paperna (1964) |
|  |  |  | fish ponds | Israel | Lahav & Sarig (1967) |
|  | *Sarotherodon galilaeus* | Cichlidae | coastal plain streams | Israel | Paperna (1964) |
| *Ergasilus gibbus* von Nordmann, 1832 | *Anguilla anguilla* | Anguillidae | Aegean Sea | Turkey | Altunel (1980) |
|  |  |  | Karacabey Lagoon Lake | Turkey | Altunel (1990) |
|  |  |  | Köyceğiz Lake | Turkey | Soylu *et al*. (2013) |
|  |  |  | Maayan Tzvi fishponds | Israel | Paperna & Lahav (1971) |
|  |  |  | coastal ponds, south France | France | Raibaut & Altunel (1976) |
|  |  |  | Lake Ischkeul | Tunisia | Raibaut & Altunel (1976) |
|  |  |  | Boka Kotorska | Montenegro | Radujkovič & Raibaut (1987) |
|  |  |  | River Este | Portugal | Saraiva & Eiras (1996) |
|  |  |  | Aveiro Lagoon | Portugal | Hermida *et al*. (2008) |
|  |  |  | Biguglia Pond, Corsica | France | Caillot *et al.* (1999) |
|  |  |  | Biguglia Lagoon, Corsica | France | Filippi *et al*. (2013) |
|  |  |  | River Ulla | Spain | Aguilar *et al*. (2005) |
|  |  |  | Lake Oubeïra | Algeria | Tahri *et al*. (2018) |
| *Ergasilus iraquensis* Amado, 2001 | *Planiliza subviridis* | Mugilidae | marine waters, Khor Al-Zubair | Iraq | Amado *et al*. (2001), Al-Daraji (2002c) |
|  |  |  | marine waters, Khor Abdullah | Iraq | Mhaisen & Al-Daraji (2023) |
| ***Ergasilus italicus* n. sp.** | ***Protochondrostoma genei*** | **Leuciscidae** | **Torrente Cerfone, Le Ville** | **Italy** | **present study** |
| *Ergasilus lagunaris* Grandori, 1925 | ***-*** | **-** | **Venetian Lagoon** | **Italy** | Grandori (1925) |
| *Ergasilus lizae* Krøyer, 1863 (=*Ergasilus nanus* Beneden, 1870) | *Anguilla anguilla* | Anguillidae | Köyceğiz Lake | Turkey | Soylu *et al*. (2013) |
|  | *Barbus barbus* | Cyprinidae | Lake Ischkeul | Tunisia | Raibaut *et al.* (1971) |
|  | ***Barbus cyclolepis*** | **Cyprinidae** | **Macropotamos river, Filiouri basin** | **Greece** | **present study** |
|  | ***Barbus sperchiensis*** | **Cyprinidae** | **Sperchios, Ypati** | **Greece** | **present study** |
|  | *Chelon auratus* | Mugilidae | Kızılırmak Delta | Turkey | Özer & Kirca (2013) |
|  |  |  | Sarıkum Lagoon Lake | Turkey | Öztürk (2013) |
|  |  |  | Black Sea, Samsun coast | Turkey | Özer *et al*. (2017) |
|  |  |  | Black Sea, Sinop coast | Turkey | Özer *et al*. (2017) |
|  |  |  | Boka Kotorska | Montenegro | Radujkovič & Raibaut (1982) |
|  | *Chelon labrosus* | Mugilidae | Aegean Sea | Turkey | Altunel (1983) |
|  |  |  | Beymelek Lagoon Lake | Turkey | Yalim *et al*. (2023) |
|  |  |  | Boka Kotorska | Montenegro | Radujkovič & Raibaut (1982) |
|  | *Chelon ramada* | Mugilidae | Aegean Sea | Turkey | Altunel (1983) |
|  |  |  | Boka Kotorska | Montenegro | Radujkovič & Raibaut (1982) |
|  | *Chelon saliens* | Mugilidae | Aegean Sea | Turkey | Altunel (1983) |
|  | ***Luciobarbus graecus*** | **Cyprinidae** | **Sperchios, Ypati** | **Greece** | **present study** |
|  | *Mugil cephalus* | Mugilidae | Aegean Sea | Turkey | Tareen (1982), Altunel (1983) |
|  |  |  | Black Sea, Samsun coast | Turkey | Özer *et al*. (2017) |
|  |  |  | coastal plain streams | Israel | Paperna (1975) |
|  |  |  | Boka Kotorska, | Montenegro | Radujkovič & Raibaut (1982) |
|  |  |  | Biguglia Pond, Corsica | France | Caillot *et al.* (1999) |
|  |  |  | NA | Greece | Ragias *et al.* (2005) |
|  | *Oedalechilus labeo* | Mugilidae | Aegean Sea | Turkey | Altunel (1983) |
|  | *Planiliza haematocheilus* | Mugilidae | Black Sea | Turkey | Öktener & Trilles (2004) |
|  | *Planiliza subviridis* | Mugilidae | Garmat Ali River | Iraq | Adday (2013) |
|  | *Sparus aurata* | Sparidae | Scardovari Lagoon | Italy | Lui *et al*. (2013) |
| *Ergasilus luteusi* Al-Sahlany, Adday & Ali, 2024 | *Carasobarbus luteus* | Cyprinidae | Al-Gharraf River | Iraq | Al-Sahlany *et al.* (2024) |
|  | *Planiliza abu* | Mugilidae | Al-Gharraf River | Iraq | Al-Sahlany *et al.* (2024) |
| *Ergasilus megaceros* Wilson, 1916 | *Carassius carassius* | Cyprinidae | Beni-Haroun dam, Mila | Algeria | Berrouk *et al*. (2018) |
|  | *Luciobarbus callensis* | Cyprinidae | Beni-Haroun dam, Mila | Algeria | Berrouk *et al*. (2018) |
| *Ergasilus mosulensis* Rahemo, 1982 | *Acanthobrama marmid* | Leuciscidae | Tigris River, Mosul | Iraq | Rahemo (2011) |
|  | *Acanthopagrus arabicus* | Sparidae | Shatt Al-Arab River | Iraq | Mhaisen & Al-Daraji (2023) |
|  | *Alburnus sellal* | Leuciscidae | Atatürk Dam Lake | Turkey | Öktener *et al*. (2008) |
|  |  |  | Garmat Ali River | Iraq | Mhaisen & Al-Daraji (2023) |
|  |  |  | Shatt Al-Arab River | Iraq | Mhaisen & Al-Daraji (2023) |
|  | *Carasobarbus luteus* | Cyprinidae | Mehaijeran Creek | Iraq | Mhaisen *et al*. (1986), Khamees & Mhaisen (1988), Mhaisen & Al-Daraji (2023) |
|  |  |  | Al-Hammar Marsh in Basrah Province | Iraq | Mhaisen & Al-Daraji (2023) |
|  |  |  | Tigris River, Salah Al-Din Province | Iraq | Mhaisen & Al-Daraji (2023) |
|  |  |  | Dokan Lake | Iraq | Mhaisen & Al-Daraji (2023) |
|  |  |  | Garmat Ali River | Iraq | Mhaisen & Al-Daraji (2023) |
|  |  |  | Al-Qadisiya Dam Lake | Iraq | Balasem *et al*. (2003) |
|  |  |  | Diyala River | Iraq | Al-Rubaie *et al* (2003) |
|  |  |  | Some drainage networks in Baghdad Province | Iraq | Balasem et al. (2002b) |
|  |  |  | Some drainage networks in Al-Diwaniyah Province | Iraq | Mhaisen & Al-Daraji (2023) |
|  |  |  | Some drainage networks in Karbala Province | Iraq | Al-Hajimi (2021) |
|  |  |  | Euphrates River, Karbala Province | Iraq | Al-Saadi (2007), Al-Saadi *et al.*(2010) |
|  |  |  | Shatt Al-Arab River | Iraq | Mhaisen & Al-Daraji (2023) |
|  |  |  | fish markets, Basrah Province | Iraq | Mhaisen (1986) |
|  | *Carassius auratus* | Cyprinidae | Shatt Al-Arab River | Iraq | Mhaisen & Al-Daraji (2023) |
|  | *Carassius carassius* | Cyprinidae | Garmat Ali River | Iraq | Mhaisen & Al-Daraji (2023) |
|  | *Coptodon zillii* | Cichlidae | Ibn-Najim Marsh in Al-Najaf Al-Ashraf Province | Iraq | Mhaisen & Al-Daraji (2023) |
|  | *Ctenopharyngodon idella* | Xenocyprididae | fish ponds, Babylon Province | Iraq | Al-Zubaidy (1998) |
|  |  |  | Garmat Ali River | Iraq | Mhaisen & Al-Daraji (2023) |
|  |  |  | fish ponds, Baghdad Province | Iraq | Asmar *et al* (2004) |
|  | *Cyprinion kais* | Cyprinidae | Ibn-Najim Marsh in Al-Najaf Al-Ashraf Province | Iraq | Mhaisen & Al-Daraji (2023) |
|  | ***Cyprinion macrostomus*** | **Cyprinidae** | **Tigris River, Mosul** | Iraq | **Rahemo (1982)** |
|  |  |  | Ibn-Najim Marsh in Al-Najaf Al-Ashraf Province | Iraq | Mhaisen & Al-Daraji (2023) |
|  | *Cyprinus carpio* | Cyprinidae | Dokan Lake | Iraq | Mhaisen & Al-Daraji (2023) |
|  |  |  | fish ponds and farms, Babylon Province | Iraq | Al-Zubaidy (1998) |
|  |  |  | Garmat Ali River | Iraq | Mhaisen & Al-Daraji (2023) |
|  |  |  | Fish ponds and farms, Basrah Province | Iraq | Abed (2005), Hussein *et al.* (2011) |
|  |  |  | Ashar Canal | Iraq | Mhaisen & Al-Daraji (2023) |
|  |  |  | Tigris River, Baghdad Province | Iraq | Mhaisen & Al-Daraji (2023) |
|  |  |  | Euphrates River, Al-Anbar Province | Iraq | Al-Salmany (2022) |
|  | *Glyptothorax steindachneri* | Sisoridae | Euphrates River, Babylon Province | Iraq | Al-Sa'adi (2007), Mhaisen *et al*. (2015) |
|  | *Heteropneustes fossilis* | Heteropneustidae | Garmat Ali River | Iraq | Mhaisen & Al-Daraji (2023) |
|  | *Hypophthalmichthys molitrix* | Xenocyprididae | fish ponds and farms, Babylon Province | Iraq | Al-Zubaidy (1998) |
|  | *Leuciscus vorax* | Leuciscidae | Al-Hammar Marsh in Basrah Province | Iraq | Mhaisen & Al-Daraji (2023) |
|  |  |  | Garmat Ali River | Iraq | Mhaisen & Al-Daraji (2023) |
|  |  |  | Euphrates River, Karbala Province | Iraq | Al-Saadi (2007), Al-Saadi *et al* (2010) |
|  |  |  | Tigris River, Baghdad Province | Iraq | Mhaisen & Al-Daraji (2023) |
|  |  |  | Euphrates River, Al-Anbar Province | Iraq | Al-Salmany (2022) |
|  | *Luciobarbus schejch* | Cyprinidae | Tigris River, Baghdad Province | Iraq | Mhaisen & Al-Daraji (2023) |
|  | *Mastacembelus mastacembelus* | Mastacembelidae | Garmat Ali River | Iraq | Mhaisen & Al-Daraji (2023) |
|  | *Mesopotamichthys sharpeyi* | Cyprinidae | Al-Hammar Marsh in Basrah Province | Iraq | Mhaisen & Al-Daraji (2023) |
|  |  |  | Garmat Ali River | Iraq | Mhaisen & Al-Daraji (2023) |
|  | *Mystus pelesius* | Bagridae | Garmat Ali River | Iraq | Mhaisen & Al-Daraji (2023) |
|  | *Planiliza abu* | Mugilidae | Atatürk Dam Lake | Turkey | Öktener *et al*. (2007) |
|  |  |  | Tigris River, Nineveh Province | Iraq | Mhaisen & Al-Daraji (2023) |
|  |  |  | Mehaijeran Creek | Iraq | Mhaisen *et al*. (1986, 1988), Mhaisen & Al-Daraji (2023) |
|  |  |  | Al-Hammar Marsh in Basrah Province | Iraq | Mhaisen & Al-Daraji (2023) |
|  |  |  | fish markets, Basrah Province | Iraq | Mhaisen (1986) |
|  |  |  | Shatt Al-Arab River | Iraq | Ho *et al*. (1996), Khamees & Mhaisen (2001) |
|  |  |  | Garmat Ali River | Iraq | Khamees (1996), Khamees & Mhaisen (1998), Al-Niaeem (2006), Mhaisen & Al-Daraji (2023) |
|  |  |  | Euphrates River, Al-Anbar Province | Iraq | Al-Alusi (1998), Al-Alusi (2010), Al-Salmany (2015), Al-Salmany (2022) |
|  |  |  | Shatt Al-Arab River, Basrah Province | Iraq | Ho *et al*. (1996), Mhaisen & Al-Daraji (2023) |
|  |  |  | fish ponds and farms, Baghdad Province | Iraq | Mohammad-Ali *et al*. (1999) |
|  |  |  | Diyala River | Iraq | Balasem *et al.* (2001), Mhaisen & Al-Daraji (2023) |
|  |  |  | some drainage networks, Baghdad Province | Iraq | Balasem *et al.* (2002b) |
|  |  |  | Al-Qadisiya Dam Lake | Iraq | Balasem *et al.* (2003) |
|  |  |  | Some drainage networks in Babylon Province | Iraq | Mhaisen & Al-Daraji (2023) |
|  |  |  | Fish ponds and farms, Basrah Province | Iraq | Abed (2005), Hussein *et al.* (2011) |
|  |  |  | Fish ponds and farms, Babylon Province | Iraq | Al-Dulaimi *et al*. (2006) |
|  |  |  | Euphrates River, Karbala Province | Iraq | Al-Saadi (2007), Al-Saadi *et al.* (2010, 2011) |
|  |  |  | Euphrates River, Babylon Province | Iraq | Hussain (2007) |
|  |  |  | Ashar Canal | Iraq | Mhaisen & Al-Daraji (2023) |
|  |  |  | Tigris River, Baghdad Province | Iraq | Mhaisen & Al-Daraji (2023) |
|  |  |  | Some drainage networks in Karbala Province | Iraq | Al-Hajimi (2021) |
|  |  |  | Al-Dalmaj Marsh of Al-Diwaniyah Province | Iraq | Al-Khenifsawy & Al-Mayli (2022), Mhaisen & Al-Daraji (2023) |
|  | *Planiliza subviridis* | Mugilidae | Shatt Al-Arab River, Basrah Province | Iraq | Mhaisen & Al-Daraji (2023) |
|  |  |  | Garmat Ali River | Iraq | Mhaisen & Al-Daraji (2023) |
|  |  |  | Ashar Canal | Iraq | Mhaisen & Al-Daraji (2023) |
|  | *Silurus triostegus* | Siluridae | Tigris River, Nineveh Province | Iraq | Mhaisen & Al-Daraji (2023) |
|  |  |  | Tigris River, Mosul | Iraq | Rahemo & Al-Niaeemi (2001) |
|  |  |  | Al-Hammar Marsh in Basrah Province | Iraq | Abbas (2007), Mhaisen & Al-Daraji (2023) |
|  |  |  | Garmat Ali River | Iraq | Jori (2006), Awad *et al*. (2007b, c), Mhaisen & Al-Daraji (2023) |
|  |  |  | Greater Zab River | Iraq | Abdullah & Shwani (2010), Mhaisen & Al-Daraji (2023) |
|  | *Squalius lepidus* | Leuciscidae | Darbandikhan Lake | Iraq | Abdullah & Abdullah (2015a, b, c), Mhaisen & Al-Daraji (2023) |
| *Ergasilus ogawai* Kabata, 1992 | *Acanthobrama marmid* | Leuciscidae | Garmat Ali River | Iraq | Mhaisen & Al-Daraji (2023) |
|  |  |  | Ashar Canal | Iraq | Mhaisen & Al-Daraji (2023) |
|  | *Acanthopagrus arabicus* | Sparidae | Garmat Ali River | Iraq | Adday *et al*. (2006a), Mhaisen & Al-Daraji (2023) |
|  |  |  | Ashar Canal | Iraq | Mhaisen & Al-Daraji (2023) |
|  | *Alburnus sellal* | Leuciscidae | Garmat Ali River | Iraq | Mhaisen & Al-Daraji (2023) |
|  | *Carasobarbus luteus* | Cyprinidae | Garmat Ali River | Iraq | Mhaisen & Al-Daraji (2023) |
|  | *Carassius auratus* | Cyprinidae | Garmat Ali River | Iraq | Mhaisen & Al-Daraji (2023) |
|  | *Coptodon zillii* | Cichlidae | Garmat Ali River | Iraq | Mhaisen & Al-Daraji (2023) |
|  | *Cyprinus carpio* | Cyprinidae | Garmat Ali River | Iraq | Mhaisen & Al-Daraji (2023) |
|  |  |  | Shatt Al-Arab River | Iraq | Eassa *et al*. (2014) |
|  | *Hemiculter leucisculus* | Xenocyprididae | Garmat Ali River | Iraq | Mhaisen & Al-Daraji (2023) |
|  | *Heteropneustes fossilis* | Heteropneustidae | Garmat Ali River | Iraq | Mhaisen & Al-Daraji (2023) |
|  | *Leuciscus vorax* | Leuciscidae | Garmat Ali River | Iraq | Mhaisen & Al-Daraji (2023) |
|  | *Luciobarbus schejch* | Cyprinidae | Garmat Ali River | Iraq | Mhaisen & Al-Daraji (2023) |
|  | *Mastacembelus simack* | Mastacembelidae | Garmat Ali River | Iraq | Adday *et al*. (2006a), Mhaisen & Al-Daraji (2023) |
|  | *Mystus pelusius* | Bagridae | Garmat Ali River | Iraq | Adday *et al*. (2006), Mhaisen & Al-Daraji (2023) |
|  | *Planiliza abu* | Mugilidae | Garmat Ali River | Iraq | Mhaisen & Al-Daraji (2023) |
|  | *Planiliza subviridis* | Mugilidae | Garmat Ali River | Iraq | Mhaisen & Al-Daraji (2023) |
|  |  |  | Ashar Canal | Iraq | Mhaisen & Al-Daraji (2023) |
|  | *Silurus triostegus* | Siluridae | Garmat Ali River | Iraq | Adday *et al*. (2006a, b), Mhaisen & Al-Daraji (2023) |
|  | *Tenualosa ilisha* | Dorosomatidae | Garmat Ali River | Iraq | Mhaisen & Al-Daraji (2023) |
| *Ergasilus pararostralis* Amado, 2001 | *Planiliza subviridis* | Mugilidae | marine waters, Khor Al-Zubair | Iraq | Amado et al. (2001), Al-Daraji (2002b) |
|  |  |  | marine waters, Khor Abdullah | Iraq | Mhaisen & Al-Daraji (2023) |
|  | *Silurus triostegus* | Siluridae | Al-Hammar Marsh in Basrah Province | Iraq | Jori (2006), Awad *et al*. (2007a) |
| *Ergasilus peregrinus* Heller, 1865 | *Acanthobrama* sp. | Leuciscidae | Al-Qadisiya Dam Lake | Iraq | Balasem *et al*. (2003) |
|  | *Acanthobrama urmianus* | Leuciscidae | West Azarbaijan, Zanjan | Iraq | Pazooki & Masoumian (2012) |
|  | *Arabibarbus grypus* | Cyprinidae | Al-Qadisiya Dam Lake | Iraq | Balasem *et al*. (2003) |
|  |  |  | Tigris River, Baghdad Province | Iraq | Bdair & Al-Rudainy (2018) |
|  | *Capoeta gracilis* | Cyprinidae | West Azarbaijan, Zanjan | Iran | Pazooki & Masoumian (2012) |
|  | *Carasobarbus luteus* | Cyprinidae | Euphrates River, Karbala Province | Iraq | Al-Saadi (2007), Al-Saadi *et al*. (2010) |
|  |  |  | Euphrates River, Babylon Province | Iraq | Al-Sa'adi (2007), Mhaisen *et al*. (2015) |
|  |  |  | Tigris River, Salah Al-Din Province | Iraq | Mhaisen & Al-Daraji (2023) |
|  | *Chondrostoma regium* | Leuciscidae | Tigris River, Baghdad Province | Iraq | Bdair & Al-Rudainy (2018) |
|  | *Ctenopharyngodon idella* | Xenocyprididae | Khandaghloo Res | Iran | Pazooki & Masoumian (2012) |
|  | *Cyprinus carpio* | Cyprinidae | Souk-Ahras, Mila | Algeria | Boucenna *et al*. (2015) |
|  |  |  | West Azarbaijan, Zanjan | Iran | Pazooki & Masoumian (2012) |
|  |  |  | Al-Qadisiya Dam Lake | Iraq | Balasem *et al*. (2003) |
|  | *Glyptothorax steindachneri* | Sisoridae | Euphrates River, Babylon Province | Iraq | Al-Sa'adi (2007), Mhaisen *et al*. (2015) |
|  | *Leuciscus vorax* | Leuciscidae | Tigris River, Salah Al-Din Province | Iraq | Mhaisen & Al-Daraji (2023) |
|  |  |  | Euphrates River, Karbala Province | Iraq | Al-Saadi (2007), Al-Saadi *et al*. (2010) |
|  | *Luciobarbus callensis* | Cyprinidae | Foum El Kangha | Algeria | Boucenna *et al*. (2018) |
|  | *Mesopotamichthys sharpeyi* | Cyprinidae | Euphrates River, Karbala Province | Iraq | Al-Saadi (2007), Al-Saadi *et al*. (2010) |
|  | *Planiliza abu* | Mugilidae | Tigris River, Salah Al-Din Province | Iraq | Mhaisen & Al-Daraji (2023) |
|  |  |  | Al-Qadisiya Dam Lake | Iraq | Balasem *et al*. (2003) |
|  |  |  | Euphrates River, Babylon Province | Iraq | Al-Sa'adi (2007), Mhaisen *et al*. (2015) |
|  |  |  | Some drainage networks in Karbala Province | Iraq | Al-Hajimi (2021) |
|  | *Squalius turcicus* | Leuciscidae | Khandaghloo Res | Iran | Pazooki & Masoumian (2012) |
| *Ergasilus rostralis* Ho, Jayarajan & Radhakrishnan, 1992 | *Acanthobrama marmid* | Leuciscidae | Garmat Ali River | Iraq | Mhaisen & Al-Daraji (2023) |
|  | *Acanthopagrus arabicus* | Sparidae | Garmat Ali River | Iraq | Mhaisen & Al-Daraji (2023) |
|  |  |  | Ashar Canal | Iraq | Mhaisen & Al-Daraji (2023) |
|  | *Alburnus sellal* | Leuciscidae | Garmat Ali River | Iraq | Mhaisen & Al-Daraji (2023) |
|  | *Arabibarbus grypus* | Cyprinidae | Euphrates River, Karbala Province | Iraq | Al-Saadi (2007), Al-Saadi *et al*. (2010) |
|  | ***Barbus lacerta*** | **Cyprinidae** | **Kani Shok, tributary of Tabin River** | **Iraq** | **present study** |
|  | ***Capoeta umbla*** | **Cyprinidae** | **wadi Kalat Shirah, tributary of Tabin River** | **Iraq** | **present study** |
|  | *Carasobarbus luteus* | Cyprinidae | Garmat Ali River | Iraq | Mhaisen & Al-Daraji (2023) |
|  |  |  | Euphrates River, Karbala Province | Iraq | Al-Saadi (2007), Al-Saadi *et al*. (2010) |
|  |  |  | Ashar Canal | Iraq | Mhaisen & Al-Daraji (2023) |
|  |  |  | Euphrates River, Al-Muthanna Province | Iraq | Al-Helli (2019) |
|  | *Carassius auratus* | Cyprinidae | Garmat Ali River | Iraq | Mhaisen & Al-Daraji (2023) |
|  |  |  | Ashar Canal | Iraq | Mhaisen & Al-Daraji (2023) |
|  |  |  | Diyala River | Iraq | Mhaisen & Al-Daraji (2023) |
|  | *Coptodon zillii* | Cichlidae | Garmat Ali River | Iraq | Mhaisen & Al-Daraji (2023) |
|  | *Ctenopharyngodon idella* | Xenocyprididae | Garmat Ali River | Iraq | Mhaisen & Al-Daraji (2023) |
|  | *Cyprinus carpio* | Cyprinidae | Garmat Ali River | Iraq | Al-Niaeem (2006), Al-Salim *et al*. (2007), Mhaisen & Al-Daraji (2023) |
|  |  |  | Ashar Canal | Iraq | Mhaisen & Al-Daraji (2023) |
|  | *Hemiculter leucisculus* | Xenocyprididae | Garmat Ali River | Iraq | Mhaisen & Al-Daraji (2023) |
|  | *Heteropneustes fossilis* | Heteropneustidae | Garmat Ali River | Iraq | Mhaisen & Al-Daraji (2023) |
|  | *Leuciscus vorax* | Leuciscidae | Garmat Ali River | Iraq | Mhaisen & Al-Daraji (2023) |
|  |  |  | Euphrates River, Karbala Province | Iraq | Al-Saadi (2007), Al-Saadi *et al*. (2010) |
|  |  |  | Ashar Canal | Iraq | Mhaisen & Al-Daraji (2023) |
|  | *Luciobarbus schejch* | Cyprinidae | Garmat Ali River | Iraq | Mhaisen & Al-Daraji (2023) |
|  | *Mesopotamichthys sharpeyi* | Cyprinidae | Euphrates River, Karbala Province | Iraq | Al-Saadi (2007), Al-Saadi *et al*. (2010) |
|  | *Mystus pelusius* | Bagridae | Garmat Ali River | Iraq | Mhaisen & Al-Daraji (2023) |
|  | *Planiliza abu* | Mugilidae | Shatt Al-Arab River | Iraq | Khamees & Mhaisen (1995), Ho *et al*. (1996), Khamees & Mhaisen (2001) |
|  |  |  | Garmat Ali River | Iraq | Khamees (1996), Khamees & Mhaisen (1998), Al-Salim & Jori (2002), Mhaisen & Al-Daraji (2023) |
|  |  |  | Fish ponds and farms, Basrah Province | Iraq | Abed (2005), Al-Niaeem (2006), Al-Salim *et al*. (2007), Hussein *et al*. (2011) |
|  |  |  | Euphrates River, Al-Anbar Province | Iraq | Al-Alusi (2010) |
|  |  |  | Ashar Canal | Iraq | Mhaisen & Al-Daraji (2023) |
|  |  |  | Euphrates River, Al-Muthanna Province | Iraq | Al-Helli (2019) |
|  | *Planiliza subviridis* | Mugilidae | marine waters, Khor Al-Zubair | Iraq | Al-Daraji (1995) |
|  |  |  | Garmat Ali River | Iraq | Al-Salim & Jori (2002), Mhaisen & Al-Daraji (2023) |
|  |  |  | Ashar Canal | Iraq | Mhaisen & Al-Daraji (2023) |
|  | *Silurus triostegus* | Siluridae | Garmat Ali River | Iraq | Mhaisen & Al-Daraji (2023) |
|  |  |  | Al-Hammar Marsh in Basrah Province | Iraq | Jori (2006) |
|  | *Tenualosa ilisha* | Dorosomatidae | Garmat Ali River | Iraq | Mhaisen & Al-Daraji (2023) |
| *Ergasilus sarsi* Capart, 1944 | *Coptodon zillii* | Cichlidae | Port Said | Egypt | Easa et al. (1989) |
|  | *Lates niloticus* | Latidae | Qena, Upper Egypt | Egypt | El-Seify *et al*. (2013) |
|  | *Mugil cephalus* | Mugilidae | Lake Manzala | Egypt | Marzouk *et al*. (2001) |
|  |  |  | Suez Canal | Egypt | Ezz El-Dien (1994) |
|  | *Oreochromis niloticus* | Cichlidae | Kafr El-Sheikh governorate | Egypt | El-Moghazy (2008) |
| *Ergasilus sieboldi* von Nordmann, 1832 | *Abramis brama* | Leuciscidae | Turkey | Turkey | Geldiay & Balik (1974) |
|  |  |  | Tolisa River | Bosnia and Herzegovina | Nedić *et al*. (2014) |
|  |  |  | Beni-Haroun dam, Mila | Algeria | Berrouk *et al*. (2021) |
|  | *Acanthobrama marmid* | Leuciscidae | Beni-Haroun dam, Mila | Algeria | Berrouk *et al*. (2018) |
|  |  |  | Lesser Zab River | Iraq | Rashid *et al*. (1989) |
|  |  |  | fish ponds and farms, Baghdad Province | Iraq | Mhaisen & Al-Daraji (2023) |
|  |  |  | Tigris River, Neinava Province | Iraq | Al-Jawda *et al*. (2003) |
|  |  |  | Garmat Ali River | Iraq | Mhaisen & Al-Daraji (2023) |
|  | *Acanthobrama* sp. | Leuciscidae | Al-Qadisiya Dam Lake | Iraq | Balasem *et al*. (2003) |
|  | *Acanthopagrus arabicus* | Sparidae | Garmat Ali River | Iraq | Mhaisen & Al-Daraji (2023) |
|  | *Alburnus alburnus* | Leuciscidae | Modrac Lake | Bosnia and Herzegovina | Skenderovic (2015) |
|  | *Alburnus istanbulensis* | Leuciscidae | Ömerli Dam Lake | Turkey | Alas *et al*. (2015) |
|  | *Alburnus sellal* | Leuciscidae | Göynük Stream | Turkey | Koyun *et al*. (2019) |
|  |  |  | NA | Turkey | Geldiay & Balik (1974) |
|  |  |  | Garmat Ali River | Iraq | Mhaisen & Al-Daraji (2023) |
|  |  |  | fish ponds and farms, Baghdad Province | Iraq | Mhaisen & Al-Daraji (2023) |
|  |  |  | some drainage networks, Baghdad Province | Iraq | Balasem *et al*. (2002b) |
|  | *Alburnus orontis* | Leuciscidae | Murat River | Turkey | Tunç & Koyun (2018) |
|  |  |  | Balıklıag Stream | Turkey | Cengizler & Goksu (1994) |
|  | *Ameiurus melas* | Ictaluridae | Sava River | Bosnia and Herzegovina | Skenderovic *et al*. (2015) |
|  | *Ameiurus nebulosus* | Ictaluridae | Sava River | Bosnia and Herzegovina | Nedić *et al*. (2014) |
|  | *Anatolichthys danfordii* | Aphaniidae | Balıklıag Stream | Turkey | Cengizler & Goksu (1994) |
|  | *Aphanius chantrei* | Aphaniidae | Sarıkum Lagoon | Turkey | Öztürk & Özer (2008) |
|  | *Arabibarbus grypus* | Cyprinidae | Euphrates River, Karbala Province | Iraq | Al-Saadi (2007), Al-Saadi *et al*. (2010) |
|  |  |  | Diyala River | Iraq | Ali *et al*. (1987) |
|  |  |  | fish ponds and farms, Baghdad Province | Iraq | Mhaisen & Al-Daraji (2023) |
|  |  |  | Al-Qadisiya Dam Lake | Iraq | Balasem *et al*. (2003) |
|  |  |  | Euphrates River, Babylon Province | Iraq | Al-Zubaidy (2007) |
|  | *Barbus lacerta* | Cyprinidae | Sarıkum Lagoon Lake | Turkey | Öztürk (2005) |
|  | *Boleophthalmus dussumieri* | Oxudercidae | Shatt Al-Arab River | Iraq | Al-Janaibi (2010) |
|  | *Capoeta capoeta* | Cyprinidae | Murat River | Turkey | Koyun *et al*. (2015) |
|  | *Carasobarbus luteus* | Cyprinidae | Mahabad Reservoir | Iran | Mirhashemi Nasab & Pazooki (2003) |
|  |  |  | Diyala River | Iraq | Ali *et al*. (1987), Mhaisen & Al-Daraji (2023) |
|  |  |  | Greater Zab River | Iraq | Rashed & Hussain (1988) |
|  |  |  | Al-Qadisiya Dam Lake | Iraq | Asmar *et al*. (1999), Balasem *et al*. (2003) |
|  |  |  | Tigris River, Salah Al-Din Province | Iraq | Al-Jawda *et al*. (2000) |
|  |  |  | fish ponds and farms, Baghdad Province | Iraq | Mhaisen & Al-Daraji (2023) |
|  |  |  | some drainage networks, Baghdad Province | Iraq | Balasem *et al*. (2002a) |
|  |  |  | Euphrates River, Thi Qar Province | Iraq | Al-Abbaddie (2006) |
|  |  |  | Euphrates River, Karbala Province | Iraq | Al-Saadi (2007), Al-Saadi *et al*. (2010) |
|  |  |  | Euphrates River, Babylon Province | Iraq | Al-Zubaidy (2007) |
|  |  |  | Garmat Ali River | Iraq | Mhaisen & Al-Daraji (2023) |
|  | *Carassius auratus* | Cyprinidae | Garmat Ali River | Iraq | Mhaisen & Al-Daraji (2023) |
|  |  |  | Diyala River | Iraq | Mhaisen & Al-Daraji (2023) |
|  | *Carassius carassius* | Cyprinidae | Balıklıag Stream | Turkey | Cengizler & Goksu (1994) |
|  |  |  | Modrac Lake | Bosnia and Herzegovina | Skenderovic *et al*. (2015) |
|  |  |  | Beni-Haroun dam, Mila | Algeria | Berrouk *et al*. (2022) |
|  |  |  | Foum El- Khonga and Ain El Dalia dams | Algeria | Berrouk *et al*. (2018) |
|  |  |  | Al-Habbaniyah Lake | Iraq | Mhaisen *et al*. (1999) |
|  | *Chondrostoma nasus* | Leuciscidae | Beni-Haroun dam, Mila | Algeria | Berrouk *et al*. (2018) |
|  | *Chondrostoma regium* | Leuciscidae | Keban Dam Lake | Turkey | Sağlam (1992) |
|  |  |  | Diyala River | Iraq | Ali *et al*. (1987) |
|  |  |  | Tigris River, Baghdad Province | Iraq | Mhaisen *et al*. (1995) |
|  |  |  | Bahdinan River in Erbil Province | Iraq | Bilal & Abdullah (2008), Mhaisen & Al-Daraji (2023) |
|  |  |  | Keban Dam Lake | Turkey | Sağlam (1992) |
|  | *Coptodon zillii* | Cichlidae | Garmat Ali River | Iraq | Mhaisen & Al-Daraji (2023) |
|  | *Ctenopharyngodon idella* | Xenocyprididae | Al-Habbaniyah Lake | Iraq | Ali *et al*. (1988a) |
|  |  |  | Fish ponds and farms, Babylon Province | Iraq | Al-Zubaidy (1998), Al-Jadoaa (2002) |
|  | *Cyprinion macrostomus* | Cyprinidae | Murat River | Turkey | Korkut & Koyun (2022) |
|  | *Cyprinus carpio* | Cyprinidae | NA | Turkey | Geldiay & Balik (1974) |
|  |  |  | Karacabey Lagoon Lake | Turkey | Aydoğdu *et al*. (2001) |
|  |  |  | Sapanca Lake | Turkey | Alas *et al*. (2015) |
|  |  |  | Sava River | Bosnia and Herzegovina | Nedić *et al*. (2014) |
|  |  |  | Souk-Ahras Mila | Algeria | Berrouk *et al*. (2019) |
|  |  |  | Gradche Reservoir | North Macedonia | Blazhekovikj-Dimovska.& Stojanovski (2022) |
|  |  |  | Hoorol-azim Lagoon | Iran | Jalali (1998) |
|  |  |  | Hamoon Lagoon | Iran | Sharif Rohani (1994) |
|  |  |  | Al-Habbaniyah Lake | Iraq | Ali *et al*. (1988a) |
|  |  |  | Fish ponds and farms, Babylon Province | Iraq | Ali *et al* (1988b), Mhaisen & Abul-Eis (1991), Al-Zubaidy (1998), Al-Rubaie *et al*. (2007) |
|  |  |  | Al-Qadisiya Dam Lake | Iraq | Asmar *et al*. (1999), Balasem *et al*. (2003) |
|  |  |  | Fish ponds and farms, Baghdad Province | Iraq | Mohammad-Ali *et al*. (1999), Al-Nasiri *et al*. (2002), Asmar *et al*. (2004), Mhaisen & Al-Daraji (2023) |
|  |  |  | Euphrates River, Al-Diwaniah Province | Iraq | Al-Jadoaa (2002) |
|  |  |  | Euphrates River, Karbala Province | Iraq | Al-Saadi (2007), Al-Saadi *et al*. (2010) |
|  |  |  | Euphrates River, Babylon Province | Iraq | Al-Zubaidy (2007) |
|  |  |  | fish markets, Baghdad Province | Iraq | Mansoor & Al-Shaikh (2011) |
|  |  |  | Garmat Ali River | Iraq | Mhaisen & Al-Daraji (2023) |
|  |  |  | Tigris River, Baghdad Province | Iraq | Mhaisen & Al-Daraji (2023) |
|  |  |  | Diyala River | Iraq | Mhaisen & Al-Daraji (2023) |
|  |  |  | Al-Dalmaj Marsh of Al-Diwaniyah Province | Iraq | Al-Khenifsawy & Al-Mayli (2022), Mhaisen & Al-Daraji (2023) |
|  |  |  | Euphrates River, Al-Anbar Province | Iraq | Al-Salmany (2022) |
|  | *Esox lucius* | Esocidae | NA | Turkey | Geldiay & Balik (1974) |
|  | *Garra rufa* | Cyprinidae | Diyala River | Iraq | Ali *et al*. (1987) |
|  |  |  | Euphrates River, Al-Diwaniah Province | Iraq | Al-Jadoaa (2002) |
|  | *Hemiculter leucisculus* | Xenocyprididae | Garmat Ali River | Iraq | Mhaisen & Al-Daraji (2023) |
|  | *Heteropneustes fossilis* | Heteropneustidae | Fish ponds and farms, Baghdad Province | Iraq | Mhaisen & Al-Daraji (2023) |
|  | *Hypophthalmichthys molitrix* | Xenocyprididae | Al-Habbaniyah Lake | Iraq | Ali *et al*. (1988a) |
|  |  |  | Fish ponds and farms, Babylon Province | Iraq | Al-Zubaidy (1998), Asmar *et al*. (2001), Al-Jadoaa (2002) |
|  | *Leuciscus aspius* | Leuciscidae | Karacabey Lagoon Lake | Turkey | Öztürk *et al*. (2002) |
|  |  |  | Modrac Lake | Bosnia and Herzegovina | Skenderovic *et al*. (2015) |
|  |  |  | Hoorol-azim Lagoon | Iran | Jalali (1998) |
|  |  |  | Al-Habbaniyah Lake | Iraq | Herzog (1969) |
|  |  |  | Euphrates River, Thi Qar Province | Iraq | Al-Abbaddie (2006) |
|  |  |  | Euphrates River, Karbala Province | Iraq | Al-Saadi (2007), Al-Saadi *et al*. (2010) |
|  |  |  | Garmat Ali River | Iraq | Mhaisen & Al-Daraji (2023) |
|  |  |  | Tigris River, Baghdad Province | Iraq | Mhaisen & Al-Daraji (2023) |
|  |  |  | Euphrates River, Al-Anbar Province | Iraq | Al-Salmany (2022) |
|  | *Luciobarbus callensis* | Cyprinidae | Beni-Haroun dam, Mila | Algeria | Berrouk *et al*. (2019) |
|  |  |  | Foum El Kangha | Algeria | Boucenna *et al*. (2018) |
|  | *Luciobarbus schejch* | Cyprinidae | Al-Tharthar- Tigris Canal in Salah Al-Din Province | Iraq | Khalifa (1989) |
|  |  |  | Fish ponds and farms, Baghdad Province | Iraq | Salih *et al*. (2000) |
|  |  |  | Garmat Ali River | Iraq | Mhaisen & Al-Daraji (2023) |
|  |  |  | Tigris River, Baghdad Province | Iraq | Bdair & Al-Rudainy (2018) |
|  |  |  | Euphrates River, Al-Anbar Province | Iraq | Al-Salmany (2022) |
|  | *Mesopotamichthys sharpeyi* | Cyprinidae | Hoorol-azim Lagoon | Iraq | Jalali (1998) |
|  |  |  | Al-Qadisiya Dam Lake | Iraq | Balasem *et al*. (2003) |
|  |  |  | Euphrates River, Karbala Province | Iraq | Al-Saadi (2007), Al-Saadi *et al*. (2010) |
|  | *Mystus pelusius* | Bagridae | Al-Habbaniyah Lake | Iraq | Ali *et al*. (1988a) |
|  |  |  | Euphrates River, Al-Diwaniah Province | Iraq | Al-Jadoaa (2002) |
|  | *Mugil cephalus* | Mugilidae | fish ponds, Israel | Israel | Lahav & Sarig (1967) |
|  |  |  | Karacabey Lagoon Lake | Turkey | Öztürk & Aydoğdu (2003) |
|  | *Neogobius melanostomus* | Gobionidae | Sırakaraağaçlar Stream | Turkey | Özer (2007) |
|  | *Paracapoeta trutta* | Cyprinidae | Keban Dam Lake | Turkey | Sağlam (1992) |
|  |  |  | Keban Dam Lake | Turkey | Sarıeyyüboğlu & Sağlam (1991) |
|  | *Planiliza abu* | Mugilidae | Diyala River | Iraq | Ali *et al*. (1987), Al-Shaikh *et al*. (1995), Al-Rubaie *et al*. (2003), Mhaisen & Al-Daraji (2023) |
|  |  |  | Dokan Lake | Iraq | Mhaisen & Al-Daraji (2023) |
|  |  |  | Tigris River, Baghdad Province | Iraq | Balasem *et al.* (1993), Adday *et al*. (1999), Mhaisen & Al-Daraji (2023) |
|  |  |  | Fish hatcheries in Wasit Province | Iraq | Mhaisen & Abul-Eis (1993) |
|  |  |  | Al-Qadisiya Dam Lake | Iraq | Asmar *et al*. (1999), Balasem *et al*. (2003) |
|  |  |  | Al-Habbaniyah Lake | Iraq | Mhaisen *et al*. (1999) |
|  |  |  | Fish ponds and farms, Baghdad Province | Iraq | Al-Nasiri *et al*. (2003), Asmar *et al*. (2004), Mhaisen & Al-Daraji (2023) |
|  |  |  | Euphrates River, Al-Diwaniah Province | Iraq | Al-Jadoaa (2002) |
|  |  |  | some drainage networks, Baghdad Province | Iraq | Balasem *et al*. (2002a, b), Asmar *et al*. (2003), Mhaisen *et al*. (2003) |
|  |  |  | Euphrates River, Thi Qar Province | Iraq | Al-Abbaddie (2006) |
|  |  |  | Euphrates River, Karbala Province | Iraq | Al-Saadi (2007), Al-Saadi *et al*. (2010, 2011) |
|  |  |  | Euphrates River, Babylon Province | Iraq | Al-Sa'adi (2007), Al-Zubaidy (2007), Hussain (2009), Mhaisen *et al*. (2015) |
|  |  |  | some drainage networks, Al-Diwaniyah Province | Iraq | Al-Jadoaa (2008) |
|  |  |  | Garmat Ali River | Iraq | Mhaisen & Al-Daraji (2023) |
|  |  |  | Euphrates River, Al-Anbar Province | Iraq | Mhaisen & Al-Daraji (2023) |
|  |  |  | some drainage networks, Babylon Province | Iraq | Mhaisen & Al-Daraji (2023) |
|  |  |  | some drainage networks, Karbala Province | Iraq | Al-Hajimi (2021) |
|  | *Planiliza subviridis* | Mugilidae | Garmat Ali River | Iraq | Mhaisen & Al-Daraji (2023) |
|  | *Platichthys flesus* | Pleuronectidae | Sarıkum Lagoon Lake | Turkey | Öztürk (2005) |
|  | *Sander lucioperca* | Percidae | Sava River | Bosnia and Herzegovina | Nedić *et al*. (2014) |
|  | *Scardinius erythrophthalmus* | Leuciscidae | NA | Turkey | Geldiay & Balik (1974) |
|  |  |  | Lake Trasimeno | Italy | Aisa *et al*. (1983) |
|  | *Perca fluviatilis* | Percidae | Sava River | Bosnia and Herzegovina | Skenderovic *et al*. (2015) |
|  | *Phoxinus septimaniae* | Leuciscidae | Tordera Stream | Spain | Almeida *et al*. (2024) |
|  | *Pomatoschistus marmoratus* | Gobionidae | Bafra Fish Lakes | Turkey | Alas *et al*. (2015) |
|  | *Proterorhinus marmoratus* | Gobionidae | Bafra Fish Lakes | Turkey | Alas *et al*. (2015) |
|  | *Silurus glanis* | Siluridae | Modrac Lake | Bosnia and Herzegovina | Skenderovic *et al*. (2015) |
|  | *Silurus triostegus* | Siluridae | Greater Zab River | Iraq | Abdullah & Shwani (2010), Mhaisen & Al-Daraji (2023) |
|  |  |  | Garmat Ali River | Iraq | Mhaisen & Al-Daraji (2023) |
|  |  |  | Euphrates River, Al-Anbar Province | Iraq | Al-Salmany (2022) |
|  | *Sparus aurata* | Sparidae | marine fish farm | Egypt | Abdel-Radi *et al*. (2022) |
|  | *Squalius cephalus* | Leuciscidae | Turkey | Turkey | Geldiay & Balik (1974) |
|  |  |  | Murat River | Turkey | Korkut & Koyun (2022) |
|  |  |  | Sava River | Bosnia and Herzegovina | Nedić *et al*. (2014) |
|  | *Tenualosa ilisha* | Dorosomatidae | Garmat Ali River | Iraq | Mhaisen & Al-Daraji (2023) |
|  | *Tinca tinca* | Tincidae | Uluabat Lake | Turkey | Öztürk (2002) |
|  |  |  | Sapanca Lake | Turkey | Alas *et al*. (2015) |
|  | *Vimba vimba* | Leuciscidae | Sapanca Lake | Turkey | Alas *et al*. (2015) |
|  | hybrid *Rutilus rutilus* | Leuciscidae | Modrac Lake | Bosnia and Herzegovina | Nedić *et al*. (2014) |
|  | unknown host | - | Vistonis, Doärani, Volvi | Greece | Zarfdjian & Economidis (1989) |
|  | unknown host | - | Tigris River, Baghdad Province | Iraq | Mansoor *et al*. (2012) |
|  | zooplankton | - | Ebro River | Spain | Muñoz-Colmenares *et al*. (2021) |
| *Ergasilus synanceiensis* Amado in Amado, da Rocha, Piasecki, Al-Daraji & Mhaisen, 2001 | *Pseudosynanceia melanostigma* | Synanceiidae | marine waters, Khor Al-Zubair | Iraq | Amado *et al*. (2001), Al-Daraji (2002c) |
| *Ergasilus* sp. | *Acanthobrama marmid* | Leuciscidae | Ashar Canal | Iraq | Mhaisen & Al-Daraji (2023) |
|  | *Acanthobrama urmianus* | Leuciscidae | Mahabad Reservoir | Iran | Mirhashemi Nasab & Pazooki (2003) |
|  | *Anguilla anguilla* | Anguillidae | El’Taref | Algeria | Bakaria *et al*. (2018) |
|  | *Arabibarbus grypus* | Cyprinidae | Hoorol-azim Lagoon | Iran | Jalali (1998) |
|  | *Barbus lacerta* | Cyprinidae | Vahdat Reservoir | Iran | Barzegar & Jalali (2009) |
|  | *Barbus* sp. | Cyprinidae | Timgad dam (Batna) | Algeria | Berrouk *et al*. (2018) |
|  |  |  | Sefid-rud River | Iran | Barzegar & Jalali (2009) |
|  |  |  | Karoon River | Iran | Barzegar & Jalali (2009) |
|  | *Capoeta capoeta* | Cyprinidae | Mahabad Reservoir | Iran | Mirhashemi Nasab & Pazooki (2003) |
|  | *Capoeta damascina* | Cyprinidae | Tigris Basin, Mesopotamian Region | Iran | Jafari & Miar (2011) |
|  | *Capoeta* sp. | Cyprinidae | NA | Turkey | Burgu *et al*. (1988) |
|  | *Carasobarbus luteus* | Cyprinidae | Karoon River | Iran | Molnar (1990) |
|  |  |  | Al-Hammar Marsh in Basrah Province | Iraq | Mhaisen & Al-Daraji (2023) |
|  |  |  | Greater Zab River | Iraq | Rashed & Hussain (1988) |
|  |  |  | Ashar Canal | Iraq | Mhaisen & Al-Daraji (2023) |
|  | *Carassius gibelio* | Cyprinidae | Büyükçekmece Lake | Turkey | Yardımcı *et al*. (2018) |
|  | *Chelon auratus* | Mugilidae | Latakia | Syria | Hassan *et al*. (2023) |
|  | *Chondrostoma regium* | Leuciscidae | Greater Zab River | Iraq | Al-Marjan (2016) |
|  | *Ctenopharyngodon idella* | Cyprinidae | Mahabad Reservoir | Iran | Mirhashemi Nasab & Pazooki (2003) |
|  |  |  | Hamoon Lagoon | Iran | Sharif Rohani (1994) |
|  | *Cyprinion macrostomus* | Cyprinidae | Euphrates River, Al-Diwaniah Province | Iraq | Al-Jadoaa (2002) |
|  | *Cyprinus carpio* | Cyprinidae | Uluabat, Kocadere Lakes | Turkey | Öktener (2003) |
|  |  |  | fish ponds and farms, Nineveh Province | Iraq | Al-Hamdane & Aziz (2006) |
|  | *Dicologlossa cuneata* | Soleidae | marine coast | Portugal | Marques *et al*. (2006, 2009, 2011) |
|  | *Heteropneustes fossilis* | Heteropneustidae | Al-Hammar Marsh in Basrah Province | Iraq | Al-Salim & Mohamad (1993a, b), Mhaisen & Al-Daraji (2023) |
|  | *Hypophthalmichthys molitrix* | Xenocyprididae | Fish ponds and farms, Baghdad Province | Iraq | Asmar *et al*. (2004) |
|  | *Leuciscus vorax* | Leuciscidae | Karoon River | Iran | Barzegar & Jalali (2009) |
|  |  |  | Al-Hammar Marsh in Basrah Province | Iraq | Mhaisen & Al-Daraji (2023) |
|  | *Luciobarbus schejch* | Cyprinidae | Hoorol-azim Lagoon | Iran | Jalali (1998) |
|  | *Mastacembelus simack* | Mastacembelidae | Zohreh River | Iran | Peyghan *et al*. (2011) |
|  | *Mesopotamichthys sharpeyi* | Cyprinidae | Greater Zab River | Iraq | Rashed & Hussain (1988) |
|  | *Mugil cephalus* | Mugilidae | Latakia, Syria | Syria | Hassan *et al*. (2023) |
|  | *Oreochromis niloticus* | Cichlidae | Sharkia Province | Egypt | Marzouk *et al*. (2010) |
|  |  |  | Lake Manzala | Egypt | Ibrahim & Soliman (2011) |
|  | *Planiliza abu* | Mugilidae | Hoorol-azim Lagoon | Iran | Jalali (1998) |
|  |  |  | Al-Tharthar- Tigris Canal in Salah Al-Din Province | Iraq | Ali & Shaaban (1984) |
|  |  |  | Al-Hammar Marsh in Basrah Province | Iraq | Mhaisen & Al-Daraji (2023) |
|  |  |  | Al-Majidiah Rive | Iraq | Mehdi *et al*. (2009), Mhaisen & Al-Daraji (2023) |
|  | *Planiliza subviridis* | Mugilidae | marine waters, Khor Al-Zubair | Iraq | Piasecki *et al*. (1993) |
|  | *Silurus glanis* | Siluridae | NA | Turkey | Burgu *et al*. (1988) |
|  |  |  | Mahabad Reservoir | Iran | Mirhashemi Nasab & Pazooki (2003) |
|  | *Silurus triostegus* | Siluridae | Al-Hammar Marsh in Basrah Province | Iraq | Mhaisen & Al-Daraji (2023) |
|  | *Sparus aurata* | Sparidae | Kavala lagoon | Greece | Vagianou *et al*. (2006) |
|  |  |  | Pila di Porto Tolle, Rovigo | Italy | Dezfuli *et al*. (2011) |
|  | *Squalius cephalus* | Leuciscidae | Mahabad Reservoir | Iran | Mirhashemi Nasab & Pazooki (2003) |
|  | *Tenualosa ilisha* | Dorosomatidae | Garmat Ali River | Iraq | Adday (2013) |
|  | *Tinca tinca* | Tincidae | NA | Turkey | Burgu *et al*. (1988) |
|  | zooplankton | - | NA | Albania | Mancinelli *et al*. (2019) |
| ***Mugilicola* Tripathi, 1960** |  |  |  |  |  |
| *Mugilicola bulbosa* Tripathi, 1960 | *Planiliza subviridis* | Mugilidae | Garmat Ali River | Iraq | Al-Mosawi & Adday (2024) |
| *Mugilicola kabatai* Piasecki, Khamees & Mhaisen, 1991 | *Planiliza abu* | Mugilidae | Shatt Al-Arab river | Iraq | Piasecki *et al*. (1991), Ho *et al*. (1996), Khamees (1996), Khamees & Mhaisen (1998) |
|  |  |  | marine waters, Khor Al-Zubair | Iraq | Al-Daraji (1995) |
|  |  |  | Garmat Ali River | Iraq | Al-Niaeem (2006), Mhaisen *et al*. (2018) |
|  |  |  | Al-Salihya canal | Iraq | Mhaisen *et al*. (2018) |
| *Mugilicola* sp. | *Planiliza subviridis* | Mugilidae | marine waters, Khor Al-Zubair | Iraq | Piasecki *et al*. (1993) |
| ***Neoergasilus* Yin, 1956** |  |  |  |  |  |
| ***Neoergasilus japonicus* (Harada, 1930) (=*Ergasilus japonicus* Harada, 1930)** | ***Acanthobrama marmid*** | **Leuciscidae** | **Du Choman, Aw-e Shiler River** | **Iraq** | **present study** |
|  | ***Alburnus arborella*** | **Leuciscidae** | **canale maestro de la Chiana, Chuisa dei Capannoi, Arno basis** | **Italy** | **present study** |
|  | ***Alburnus sellal*** | **Leuciscidae** | **Dukan Lake** | **Iraq** | **present study** |
|  |  |  | **Great Zab River (1)** | **Iraq** | **present study** |
|  |  |  | **Zahrzi, Tabin River** | **Iraq** | **present study** |
|  | ***Carasobarbus luteus*** | **Cyprinidae** | **Darbandikhan Lake** | **Iraq** | **present study** |
|  | *Carassius carassius* | Cyprinidae | Beni-Haroun dam, Mila | Algeria | Berrouk *et al*. (2022) |
|  |  |  | Foum El- Khonga and Ain El Dalia dams | Algeria | Berrouk *et al*. (2018) |
|  | ***Chondrostoma soetta*** | **Leuciscidae** | **Carmagnola, Cave Germaire** | **Italy** | **present study** |
|  | ***Chondrostoma regium*** | **Leuciscidae** | **Du Choman, Aw-e Shiler River** | **Iraq** | **present study** |
|  | ***Chondrostoma turnai*** | **Leuciscidae** | **Çine River, near Sitmalik** | **Turkey** | **present study** |
|  | *Cyprinus carpio* | Cyprinidae | Beni-Haroun dam, Mila | Algeria | Berrouk *et al*. (2019) |
|  | ***Cyprinion macrostomus*** | **Cyprinidae** | **Dukan Lake** | **Iraq** | **present study** |
|  |  |  | **Darbandikhan Lake** | **Iraq** | **present study** |
|  | ***Leucos aula*** | **Leuciscidae** | **Grabovač reservoir** | **Croatia** | **present study** |
|  | *Luciobarbus callensis* | Cyprinidae | Foum El Kangha | Algeria | Boucenna *et al*. (2018) |
|  | ***Luciobarbus schejch*** | **Cyprinidae** | **Du Choman, Aw-e Shiler River** | **Iraq** | **present study** |
|  | *Poecilia sphenops* | Poeciliidae | Kerman | Iran | Mirzaei *et al*. (2016) |
|  | ***Scardinius dergle*** | **Leuciscidae** | **Grabovač reservoir** | **Croatia** | **present study** |
|  | *Scardinius erythrophthalmus* | Leuciscidae | Sapanca Lake | Turkey | Soylu & Soylu (2012) |
|  | ***Squalius lepidus*** | **Leuciscidae** | **Du Choman, Aw-e Shiler River** | **Iraq** | **present study** |
|  | ***Squalius squalus*** | **Leuciscidae** | **Pazin, Pazinčica river** | **Croatia** | **present study** |
|  | ***Vimba mirabilis*** | **Leuciscidae** | **Çine River, near Sitmalik** | **Turkey** | **present study** |
|  | zooplankton | - | Lakes Acerenza, Basentello, Camastra, Cecita, Genzano, Lampeggiano, Rendina, Passante, Pertusillo, Pontano, S. Giuliano | Italy | Alfonso & Belmonte (2010) |
|  | zooplankton | - | Ebro River | Spain | Muñoz-Colmenares *et al*. (2021) |
|  | zooplankton | - | Grand Laoucien Lake, Marseille | France | Alekseev *et al*. (2021) |
| *Neoergasilus longispinosus* Yin, 1956 | *Carassius carassius* | Cyprinidae | Beni-Haroun dam, Mila | Algeria | Berrouk *et al*. (2022) |
|  | *Cyprinus carpio* | Cyprinidae | Beni-Haroun dam, Mila | Algeria | Berrouk *et al*. (2019) |
|  | *Luciobarbus callensis* | Cyprinidae | Foum El Kangha | Algeria | Boucenna *et al*. (2018) |
| *Neoergasilus* sp. | *Luciobarbus callensis* | Cyprinidae | Foum El Kangha | Algeria | Boucenna *et al*. (2018) |
| ***Nipergasilus* Yamaguti, 1939** |  |  |  |  |  |
| *Nipergasilus bora* (Yamaguti, 1939) (=*Ergasiloides bora* Yamaguti, 1939; *Yamagutia bora* (Yamaguti, 1939)) | *Chelon auratus* | Mugilidae | Yarkon River, Israel | Israel | Paperna & Lahav (1971) |
|  | *Chelon labrosus* | Mugilidae | Languedoc-Roussillon | France | Braun (1981) |
|  |  |  | Boka Kotorska | Montenegro | Radujkovič (1983) |
|  |  |  | Aegean Sea | Turkey | Ben Hassine (1983) |
|  | *Chelon saliens* | Mugilidae | Lake Burullus | Egypt | El-Rashidy & Boxshall (2000) |
|  | *Mugil cephalus* | Mugilidae | Yarkon River | Israel | Paperna & Lahav (1971), Paperna & Overstreet (1981) |
|  |  |  | Gulf of Eilat | Israel | Paperna & Overstreet (1981) |
|  |  |  | Boka Kotorska | Montenegro | Radujkovič (1983) |
|  |  |  | Aegean Sea | Turkey | Ben Hassine (1983) |
|  |  |  | Languedoc-Roussillon | France | Braun (1981) |
|  |  |  | Biguglia Pond, Corsica | France | Caillot *et al.* (1999) |
| ***Paraergasilus* Markevich, 1937** |  |  |  |  |  |
| *Paraergasilus brevidigitus* Yin, 1954 | *Carassius carassius* | Cyprinidae | Beni-Haroun dam, Mila | Algeria | Berrouk *et al*. (2022) |
| *Paraergasilus inflatus* Ho, Khamees & Mhaisen, 1996 | *Carasobarbus luteus* | Cyprinidae | Garmat Ali River | Iraq | Mhaisen *et al.* (2017) |
|  | *Cyprinus carpio* | Cyprinidae | Garmat Ali River | Iraq | Mhaisen *et al.* (2017) |
|  | *Hypophthalmichthys molitrix* | Xenocyprididae | Fish ponds and farms, Babylon Province | Iraq | Al-Zubaidy (1998) |
|  | *Leuciscus vorax* | Leuciscidae | Garmat Ali River | Iraq | Mhaisen *et al.* (2017) |
|  | *Planiliza abu* | Mugilidae | Shatt Al-Arab river | Iraq | Ho *et al.* (1996), Khamees (1996), Khamees & Mhaisen (1998), Mhaisen & Khamees (2001) |
|  |  |  | Garmat Ali River | Iraq | Mhaisen *et al.* (2017) |
|  | *Planiliza subviridis* | Mugilidae | Garmat Ali River | Iraq | Mhaisen *et al.* (2017) |
|  | *Silurus triostegus* | Siluridae | Garmat Ali River | Iraq | Mhaisen *et al.* (2017) |
|  |  |  | Al-Hammar Marsh in Basrah Province | Iraq | Jori (2006) |
| ***Paraergasilus longidigitus* Yin, 1954** | ***Alburnoides fangfangae*** | **Leuciscidae** | **Osum, Vodice** | **Albania** | **present study** |
|  | *Alburnus alburnus* | Leuciscidae | Enne Dam Lake | Turkey | Koyun *et al*. (2007) |
|  | ***Alburnus scoranza*** | **Leuciscidae** | **Skadar Lake, Shiroke** | **Albania** | **present study** |
| **Lernaeidae Cobbold, 1879** |  |  |  |  |  |
| ***Lamproglena* von Nordmann, 1832** |  |  |  |  |  |
| *Lamproglena chinensis* Yü, 1937 | *Capoeta damascina* | Cyprinidae | Chaharmahal-Bakhtirari | Iran | Pazooki & Masoumian (2012) |
|  | *Leuciscus vorax* | Leuciscidae | A drainage network at Babylon Province | Iraq | Al-Sa'adi (2022) |
|  | *Paracapoeta trutta* | Cyprinidae | Tigris River, Nineveh Province | Iraq | Mhaisen *et al*. (2024) |
| *Lamproglena compacta* Markevich, 1936 | *Capoeta gracilis* | Cyprinidae | Sajasar-rud River | Iran | Pazooki & Masoumian (2012) |
|  | *Capoeta* sp. | Cyprinidae | Mahabad Reservoir | Iran | Abdi *et al* (1995) |
|  | *Leuciscus vorax* | Leuciscidae | Hoorol-azim Lagoon | Iran | Jalali (1998) |
|  | *Luciobarbus xanthopterus* | Cyprinidae | Hoorol-azim Lagoon | Iran | Jalali (1998) |
|  | *Schizocypris altidorsalis* | Cyprinidae | Hamoon Lagoon | Iran | Sharif Rohani (1994) |
|  | *Squalius cephalus* | Leuciscidae | Khandaghloo Res | Iran | Pazooki & Masoumian (2012) |
| *Lamproglena jordani* Paperna, 1964 | ***Capoeta damascina*** | **Cyprinidae** | **Lake Tiberias** | **Israel** | **Paperna (1964)** |
|  | *Carasobarbus canis* | Cyprinidae | Lake Tiberias | Israel | Paperna (1964) |
|  | *Luciobarbus longiceps* | Cyprinidae | Lake Tiberias | Israel | Paperna (1964) |
|  | *Mirogrex terraesanctae* | Cyprinidae | Lake Tiberias | Israel | Paperna (1964) |
| *Lamproglena monodi* Capart, 1944 | *Coptodon zillii* | Cichlidae | Nile River at Assiut province | Egypt | Hassan *et al*. (2013) |
|  |  |  | River Nile (Golden Island), Giza | Egypt | Abdel-Gaber *et al*. (2017) |
|  |  |  | El-Abbassa fish farms | Egypt | Al Malki *et al*. (2021) |
|  | *Oreochromis aureus* | Cichlidae | River Nile (Golden Island), Giza | Egypt | Abdel-Gaber *et al*. (2017) |
|  |  |  | El-Abbassa fish farms | Egypt | Al Malki *et al*. (2021) |
|  | *Oreochromis niloticus* | Cichlidae | Nile River at Assiut province | Egypt | Hassan *et al*. (2013) |
|  |  |  | Qena, Upper Egypt | Egypt | El-Seify *et al*. (2013) |
|  |  |  | El-Abbassa fish farms | Egypt | Al Malki *et al*. (2021) |
|  | *Sarotherodon galilaeus* | Cichlidae | River Nile (Golden Island), Giza | Egypt | Abdel-Gaber *et al*. (2017) |
|  |  |  | El-Abbassa fish farms | Egypt | Al Malki *et al*. (2021) |
| ***Lamproglena pulchella* von Nordmann, 1832** | ***Acanthobrama microlepis*** | **Leuciscidae** | **Ölçek, Ölçeksuyu, Kura basin** | **Turkey** | **present study** |
|  | ***Alburnoides ohridanus*** | **Leuciscidae** | **Aoos** | **Greece** | **present study** |
|  | *Alburnus alborella* | Leuciscidae | Lake Garda | Italy | Boni *et al*. (1992) |
|  | *Arabibarbus grypus* | Cyprinidae | Tigris River, Baghdad Province | Iraq | Mhaisen *et al*. (1995) |
|  | ***Barbus cyri*** | **Cyprinidae** | **Yiğitkonağı closest village, Çakir, Kura basin** | **Turkey** | **present study** |
|  | *Capoeta aculeata* | Cyprinidae | Euphrates River, Al-Anbar Province | Iraq | Mhaisen *et al*. (2024) |
|  | ***Capoeta capoeta*** | **Cyprinidae** | **Ardahan, Kura basin** | **Turkey** | **present study** |
|  | *Capoeta damascina* | Cyprinidae | Greater Zab River | Iraq | Abdullah (2002), Abdullah & Mhaisen (2006) |
|  |  |  | Lesser Zab River | Iraq | Abdullah (2002) |
|  | ***Capoeta sieboldii*** | **Cyprinidae** | **Çifteler** | **Turkey** | **present study** |
|  | ***Capoeta tinca*** | **Cyprinidae** | **Çifteler** | **Turkey** | **present study** |
|  | *Capoeta umbla* | Cyprinidae | Greater Zab River | Iraq | Mhaisen *et al*. (2024) |
|  |  |  | Bahdinan River at Erbil Province | Iraq | Bilal & Abdullah (2008), Mhaisen *et al*. (2024) |
|  | *Carasobarbus luteus* | Cyprinidae | Greater Zab River | Iraq | Abdullah (2002), Abdullah & Mhaisen (2006), Mhaisen *et al*. (2024) |
|  |  |  | Lesser Zab River | Iraq | Abdullah (2002) |
|  |  |  | Darbandikhan Lake | Iraq | Abdullah (2005) |
|  |  |  | Al-Husainia Creek in Karbala Province | Iraq | Al-Saadi (2007), Al-Saadi *et al*. (2010b) |
|  |  |  | Tigris River, Salah Al-Din Province | Iraq | Kamil *et al*. (2022), Esmaeel *et al*. (2023) |
|  | *Chondrostoma regium* | Leuciscidae | Keban Lake | Turkey | Sağlam (1992) |
|  |  |  | Tigris River, Nineveh Province | Iraq | Rahemo (1977) |
|  |  |  | Diyala River | Iraq | Ali *et al*. (1987) |
|  |  |  | Tigris River, Baghdad Province | Iraq | Adday *et al*. (1999) |
|  |  |  | Greater Zab River | Iraq | Abdullah (2002), Abdullah & Mhaisen (2006) |
|  |  |  | Lesser Zab River | Iraq | Abdullah (2002) |
|  |  |  | Bahdinan River at Erbil Province | Iraq | Bilal & Abdullah (2008), Mhaisen *et al*. (2024) |
|  | *Chondrostoma soetta* | Leuciscidae | Ticino River | Italy | Galli *et al*. (1998b) |
|  | *Cyprinion macrostomus* | Cyprinidae | Bahdinan River at Erbil Province | Iraq | Bilal & Abdullah (2008), Mhaisen *et al*. (2024) |
|  | *Cyprinus carpio* | Cyprinidae | Halil-ür Lake | Turkey | Öktener *et al*. (2008) |
|  |  |  | Fish ponds and farms, Babylon Province | Iraq | Al-Zubaidy (1998) |
|  |  |  | Diyala River | Iraq | Mhaisen *et al*. (2024) |
|  |  |  | Tigris River, Salah Al-Din Province | Iraq | Kamil *et al*. (2022), Esmaeel *et al*. (2023) |
|  | *Esox lucius* | Esocidae | Tartaro River | Italy | Garbini (1898) |
|  | ***Garra rezai*** | **Cyprinidae** | **near Meydan village, inflow of Garzan river, Tigris basin** | **Turkey** | **present study** |
|  | *Garra rufa* | Cyprinidae | Diyala River | Iraq | Ali *et al*. (1987) |
|  |  |  | Tigris River, Salah Al-Din Province | Iraq | Mhaisen *et al*. (2024) |
|  |  |  | Tigris River, Baghdad Province | Iraq | Mhaisen *et al*. (1995) |
|  |  |  | Greater Zab River | Iraq | Abdullah (2002), Abdullah & Mhaisen (2006) |
|  |  |  | Lesser Zab River | Iraq | Abdullah (2002) |
|  | ***Gobio sakaryaensis*** | **Gobionidae** | **Seyitgazi** | **Turkey** | **present study** |
|  | *Leuciscus vorax* | Leuciscidae | Shatt Al-Arab River: Mehaijeran Creek | Iraq | Mhaisen *et al*. (1986), Mhaisen *et al*. (2024) |
|  |  |  | Al-Hammar Marsh in Basrah Province | Iraq | Mhaisen *et al*. (2024) |
|  |  |  | Greater Zab River | Iraq | Rashed & Hussain (1988) |
|  |  |  | Tigris River, Baghdad Province | Iraq | Balasem *et al*. (1993), Adday *et al*. (1999), Mhaisen *et al*. (2024) |
|  |  |  | Fish ponds and farms, Baghdad Province | Iraq | Mhaisen *et al*. (2024) |
|  |  |  | Al-Husainia Creek in Karbala Province | Iraq | Al-Saadi (2007), Al-Saadi *et al*. (2010) |
|  |  |  | Euphrates River, Babylon Province | Iraq | Al-Sa'adi (2007), Mhaisen *et al*. (2015) |
|  |  |  | Tigris River, Salah Al-Din Province | Iraq | Fahmy *et al*. (2019), Mhaisen *et al*. (2024) |
|  |  |  | Euphrates River, Al-Anbar Province | Iraq | Al-Salmany (2022) |
|  | *Luciobarbus brachycephalus* | Cyprinidae | Sefid-rud River | Iran | Mohkayer (1985) |
|  | *Luciobarbus esocinus* | Cyprinidae | Greater Zab River | Iraq | Rashed & Hussain (1988), Mhaisen *et al*. (2024) |
|  |  |  | Dokan Lake | Iraq | Abdullah & Rasheed (2004), Mhaisen *et al*. (2024) |
|  |  |  | Tigris River, Baghdad Province | Iraq | Adday *et al*. (1999) |
|  | *Luciobarbus schejch* | Cyprinidae | Bahdinan River at Erbil Province | Iraq | Bilal & Abdullah (2008), Mhaisen *et al*. (2024) |
|  |  |  | Dokan Lake | Iraq | Abdullah & Rasheed (2004), Mhaisen *et al*. (2024) |
|  |  |  | Tigris River, Baghdad Province | Iraq | Mhaisen *et al*. (2024) |
|  |  |  | Greater Zab River | Iraq | Mhaisen *et al*. (2024) |
|  |  |  | Bahdinan River at Erbil Province | Iraq | Bilal & Abdullah (2008), Mhaisen *et al*. (2024) |
|  | *Mesopotamichthys sharpeyi* | Cyprinidae | Al-Hammar Marsh in Basrah Province | Iraq | Mhaisen *et al*. (2024) |
|  | *Paracapoeta trutta* | Cyprinidae | Keban Lake | Turkey | Sağlam (1992) |
|  |  |  | Halil-ür Lake | Turkey | Öktener *et al*. (2008) |
|  |  |  | Tigris River, Nineveh Province | Iraq | Rahemo (1977) |
|  |  |  | Bahdinan River at Erbil Province | Iraq | Bilal & Abdullah (2008), Mhaisen *et al*. (2024) |
|  |  |  | Euphrates River, Al-Anbar Province | Iraq | Al-Salmany (2022) |
|  | *Planiliza abu* | Mugilidae | Fish ponds and farms, Babylon Province | Iraq | Mhaisen *et al*. (2024) |
|  | ***Protochondrostoma genei*** | **Leuciscidae** | **Torrente Cerfone, Le Ville** | **Italy** | **present study** |
|  | ***Sarmarutilus rubilio*** | **Leuciscidae** | **Torrente Cerfone, Intoppo** | **Italy** | **present study** |
|  | ***Scardinius acarnanicus*** | **Leuciscidae** | **Trichonis Lake, Panetolio** | **Greece** | **present study** |
|  | *Scardinius erythrophthalmus* | Leuciscidae | Sapanca Lake | Turkey | Soylu (1990) |
|  |  |  | Lake Garda | Italy | Boni *et al*. (1992) |
|  |  |  | Lake Frassino | Italy | Boni *et al*. (1992) |
|  |  |  | Lake Idro | Italy | Boni *et al*. (1992) |
|  |  |  | Lake Varese | Italy | Galli *et al*. (1998a) |
|  | ***Scardinius plotizza*** | **Leuciscidae** | **Rečina river, near Jelim lake, Hutovo Blato** | **Bosnia and Herzegovina** | **present study** |
|  | *Squalius cephalus* | Leuciscidae | Lake Garda | Italy | Boni *et al*. (1992) |
|  |  |  | Idice River | Italy | Boni *et al*. (1992) |
|  |  |  | Naviglio Grande Canal | Italy | Galli *et al*. (1998a, 2001) |
|  |  |  | Ticino River | Italy | Galli *et al*. (1998b, 2001) |
|  |  |  | Lambro (near Merone) | Italy | Galli *et al*. (2001) |
|  |  |  | Lambro (near Monza) | Italy | Galli *et al*. (2001) |
|  | ***Squalius fellowesii*** | **Leuciscidae** | **Kocaalam Deresi** | **Turkey** | **present study** |
|  | *Squalius lepidus* | Leuciscidae | Greater Zab River | Iraq | Abdullah (2002), Abdullah & Mhaisen (2006), Mhaisen *et al*. (2024) |
|  |  |  | Bahdinan River, Erbil Province | Iraq | Bilal & Abdullah (2008), Mhaisen *et al*. (2024) |
|  | *Squalius spurius* | Leuciscidae | Greater Zab River | Iraq | Mhaisen *et al*. (2024) |
|  | ***Squalius orpheus*** | **Leuciscidae** | **Angitis river, between Alistrati and Drama** | **Greece** | **present study** |
|  | ***Squalius pamvoticus*** | **Leuciscidae** | **Acheron, Gliki** | **Greece** | **present study** |
|  | ***Squalius prespensis*** | **Leuciscidae** | **Aoos, Kalithea** | **Greece** | **present study** |
|  |  |  | **Aoos** | **Greece** | **present study** |
|  | ***Squalius squalus*** | **Leuciscidae** | **Torrente Cerfone, Intoppo** | **Italy** | **present study** |
|  | ***Squalius vardarensis*** | **Leuciscidae** | **Gallikos, Mandres, Gallikos basin** | **Greece** | **present study** |
|  | ***Telestes pleurobipunctatus*** | **Leuciscidae** | **Kokitos, Pagrati** | **Greece** | **present study** |
|  | *Telestes souffia* | Leuciscidae | Lake Garda | Italy | Boni *et al*. (1992) |
|  | ***Telestes* sp.** | **Leuciscidae** | **Zagoritikos River, Baldouma** | **Greece** | **present study** |
| *Lamproglena* sp. | *Leuciscus vorax* | Leuciscidae | Hoorol-azim Lagoon | Iraq | Jalali (1998) |
|  |  |  | Euphrates River, Al-Anbar Province | Iraq | Al-Salmany (2022) |
|  | *Planiliza abu* | Mugilidae | Hoorol-azim Lagoon | Iran | Jalali (1998) |
| ***Lernaea* Linnaeus, 1758** |  |  |  |  |  |
| *Lernaea ctenopharyngodontis* Yin, 1960 | *Acanthalburnus urmianus* | Cyprinidae | West Azerbaijan | Iran | Pazooki & Masoumian (2012) |
| *Lernaea cyprinacea* Linnaeus, 1758 | *Abramis brama* | Leuciscidae | North of Iran | Iran | Jalali (1998) |
|  | *Acanthobrama marmid* | Leuciscidae | Fish ponds and farms, Baghdad Province | Iraq | Mhaisen & Abdul-Ameer (2021) |
|  | *Acanthobrama persidis* | Leuciscidae | Kaftar Lake | Iran | Barzegar & Jalali (2000) |
|  | *Acanthobrama urmianus* | Leuciscidae | Mahabad Reservoir | Iran | Mirhashemi Nasab & Pazooki (2003) |
|  | *Acipenser stellatus* | Acipenseridae | Mazandaran Province | Iran | Bozorgnia *et al*. (2018) |
|  | *Alburnus alburnus* | Leuciscidae | NA | Iran | Pazooki & Masoumian (2012) |
|  |  |  | north-eastern Spain | Spain | Maceda-Veiga *et al*. (2019) |
|  |  |  | NA | Albania | Sadikaj *et al*. (2014) |
|  | *Alburnus baliki* | Leuciscidae | Karpuzçay Creek | Turkey | Innal (2020) |
|  | *Alburnus chalcoides* | Leuciscidae | NA | Iran | Pazooki & Masoumian (2012) |
|  | *Alburnus hohenackeri* | Leuciscidae | Choghakhour Lagoon | Iran | Raissy *et al.* (2013) |
|  | *Alburnus sellal* | Leuciscidae | NA | Iran | Pazooki & Masoumian (2012) |
|  |  |  | Dorudzan | Iran | Sayyadzadeh *et al.* (2016) |
|  |  |  | Kaftar Lake | Iran | Barzegar & Jalali (2000) |
|  |  |  | Fish ponds and farms, Baghdad Province | Iraq | Mhaisen & Abdul-Ameer (2021) |
|  | *Alosa alosa* | Alosidae | Guadiana River, Spain | Spain | Perez-Bote (2005) |
|  | *Ameiurus melas* | Ictaluridae | Massaciuccoli district, Tuscany | Italy | Macchioni *et al*., (2015) |
|  | *Anguilla anguilla* | Anguillidae | north-eastern Spain | Spain | Maceda-Veiga *et al*. (2019) |
|  | *Aphaniops dispar* | Aphaniidae | Mashkid River | Iran | Malekzehi *et al*. (2014) |
|  | *Aphaniops stoliczkanus* | Aphaniidae | Fish ponds and farms, Basrah Province | Iraq | Mhaisen (1986) |
|  |  |  | Garmat Ali river | Iraq | Mhaisen & Abdul-Ameer (2021) |
|  | *Arabibarbus grypus* | Cyprinidae | Fish ponds and farms, Baghdad Province | Iraq | Al-Hamed & Hermiz (1973), Khalifa *et al*. (1978) |
|  |  |  | Mortuka stream at Erbil Province | Iraq | Abdullah (2004) |
|  |  |  | Dokan Lake | Iraq | Abdullah & Ismail (2004) |
|  |  |  | Euphrates River, Babylon Province | Iraq | Hussain (2007) |
|  |  |  | Tigris River, Baghdad Province | Iraq | Mhaisen & Abdul-Ameer (2021) |
|  | *Atherina boyeri* | Atherinidae | Draa and Ghir basins | Morocco | Clavero *et al*. (2015) |
|  | *Bangana dero* | Cyprinidae | Mashkid River | Iran | Malekzehi *et al*. (2014) |
|  | *Bartbatula* sp. | Nemacheilidae | north-eastern Spain | Spain | Maceda-Veiga *et al*. (2019) |
|  | *Barbus haasi* | Cyprinidae | Llobregat River | Spain | Gutiérrez-Galindo & Lacasa-Millán (2005) |
|  |  |  | north-eastern Spain | Spain | Maceda-Veiga *et al*. (2019) |
|  | *Barbus lacerta* | Cyprinidae | Mahabad Reservoir | Iran | Mirhashemi Nasab & Pazooki (2003) |
|  | *Barbus meridionalis* | Cyprinidae | north-eastern Spain | Spain | Maceda-Veiga *et al*. (2019) |
|  | *Barbus* sp. | Cyprinidae | Vahdat Reservoir | Iran | Mohkayer (1985) |
|  |  |  | Doghab River | Iran | Jalali (1998) |
|  | *Barbus xanthos* | Cyprinidae | Dalaman Stream | Turkey | Innal (2020) |
|  | *Blicca bjoerkna* | Leuciscidae | Boojagh Lagoon | Iran | Khara *et al*. (2011) |
|  | *Boleophthalmus dussumieri* | Oxudercidae | Garmat Ali river | Iraq | Khamees (1997) |
|  |  |  | Shatt Al-Arab river | Iraq | Al-Janabi (2010b) |
|  | *Cabdio morar* | Danionidae | Mashkid River | Iran | Malekzehi *et al*. (2014) |
|  | *Capoeta aculeata* | Cyprinidae | Dorudzan and Kor River | Iran | Sayyadzadeh *et al.* (2016) |
|  |  |  | Kaftar Lake | Iran | Barzegar & Jalali (2000) |
|  |  |  | Behesht abad River | Iran | Barzegar *et al*. (2004) |
|  |  |  | Choghakhour Lag | Iran | Raissy *et al.* (2013) |
|  | *Capoeta capoeta* | Cyprinidae | Karasu Creek, Van | Turkey | Koyun & Atici (2018) |
|  |  |  | Mahabad Reservoir | Iran | Mirhashemi Nasab & Pazooki (2003) |
|  |  |  | Doghab River | Iran | Mohkayer (1985) |
|  | *Capoeta damascina* | Cyprinidae | Chaghakhour Lagoon | Iran | Fadaei Fard *et al*. (2001) |
|  |  |  | Gandoman Lagoon | Iran | Raissy *et al*. (2013) |
|  |  |  | Kaftar Lake | Iran | Barzegar & Jalali (2000) |
|  |  |  | Tigris Basin, Mesopotamian Region | Iran | Jafari & Miar (2011) |
|  |  |  | Tigris River, Salah Al-Din Province | Iraq | Al-Jawda *et al*. (2000) |
|  |  |  | **Kayacik, Cuma stream, Euphrates basin** | **Turkey** | **present study** |
|  | *Capoeta gracilis* | Cyprinidae | NA | Iran | Pazooki & Masoumian (2012) |
|  | *Capoeta saadii* | Cyprinidae | Dorudzan | Iran | Sayyadzadeh *et al.* (2016) |
|  | *Carasobarbus luteus* | Cyprinidae | Karun River | Iran | Molnar & Baska (1993) |
|  |  |  | Zarineh-rud River | Iran | Jalali (1998) |
|  |  |  | Fish ponds and farms, Baghdad Province | Iraq | Al-Hamed & Hermiz (1973), Khalifa *et al*. (1978) |
|  |  |  | Dokan Lake | Iraq | Abdullah & Ismail (2004), Mhaisen & Abdul-Ameer (2021) |
|  |  |  | Garmat Ali river | Iraq | Khamees (1997) |
|  |  |  | Fish ponds and farms, Baghdad Province | Iraq | Mhaisen & Abdul-Ameer (2021) |
|  |  |  | Euphrates River, Al-Diwaniah Province | Iraq | Al-Jadoaa (2002), Mhaisen & Abdul-Ameer (2021) |
|  |  |  | Shatt Al-Arab river | Iraq | Mhaisen & Abdul-Ameer (2021) |
|  | *Carassius auratus* | Cyprinidae | aquaculture, Mersin | Turkey | Koyuncu (2002) |
|  |  |  | Esla River | Spain | Simon Vicente *et al*. (1973) |
|  |  |  | canal near Mantova | Italy | Fratello & Sabatini (1972) |
|  |  |  | Massaciuccoli district, Tuscany | Italy | Macchioni *et al*., (2015) |
|  |  |  | El-Wafa table-fish farm | Egypt | Mahmoud *et al*. (2009) |
|  |  |  | Kafr El-Sheikh governorate | Egypt | Noor El-Deen *et al*. (2013) |
|  |  |  | Al-Sharqia governorate | Egypt | Abu-Elala *et al*. (2018) |
|  |  |  | Dorudzan | Iran | Sayyadzadeh *et al.* (2016) |
|  |  |  | all regions | Iran | Sharif Rohani (1994); Jalali (1998) |
|  |  |  | Fish ponds and farms, Baghdad Province | Iraq | Al-Hamed & Hermiz (1973), Khalifa *et al*. (1978), Ali (1986), Asmar *et al*. (2004) |
|  |  |  | Fish ponds and farms, Basrah Province | Iraq | Mhaisen (1982, 1986), Al-Niaeem (2006) |
|  |  |  | Al-Salihiya river | Iraq | Mhaisen & Abdul-Ameer (2021) |
|  |  |  | fish aquaria in Al-Najaf Al-Ashraf Province | Iraq | Naji (2010) |
|  |  |  | Shatt Al-Arab river | Iraq | Mhaisen & Abdul-Ameer (2021) |
|  | *Carassius carassius* | Cyprinidae | Kovada Lake | Turkey | Geldiay & Balik (1974) |
|  |  |  | Karacaören II, Dam Lake | Turkey | Innal (2020) |
|  |  |  | all regions | Iran | Jalali (1998) |
|  |  |  | Beni-Haroun dam, Mila | Algeria | Berrouk *et al*. (2018) |
|  |  |  | Fish ponds and farms, Baghdad Province | Iraq | Salih *et al*. (2000) |
|  | *Carassius gibelio* | Cyprinidae | Karacaören II, Dam Lake | Turkey | Kır & Samancı (2012) |
|  |  |  | Marmara Lake | Turkey | Demir & Karakişi (2016) |
|  |  |  | Onaç Reservoir | Turkey | Innal (2020) |
|  |  |  | Çanaklı Reservoir | Turkey |  |
|  |  |  | Soğanlı Reservoir | Turkey |  |
|  |  |  | Karataş Lake | Turkey |  |
|  |  |  | Anzali Lagoon | Iran | Jalali (1998) |
|  |  |  | Choghakhor Lagoon | Iran | Raissy *et al*. (2013) |
|  | *Carassius* sp. | Cyprinidae | Anzali Lagoon | Iran | Asadzadeh Mangili *et al*. (2000) |
|  | *Channa gachua* | Channidae | Mashkid River | Iran | Malekzehi *et al*. (2014) |
|  | *Chelon ramada* | Mugilidae | Karataş Lake | Turkey | Innal *et al*. (2021) |
|  | *Chondrostoma beysehirense* | Leuciscidae | Karpuzçay Creek | Turkey | Innal *et al*. (2021) |
|  |  |  | Üzümlü pond, Konya | Turkey | Erbatur *et al*. (2018) |
|  | ***Chondrostoma meandrense*** | **Leuciscidae** | **Büyük Menderes, around 1 km east of Işıklı lake** | **Turkey** | **present study** |
|  | *Chondrostoma nasus* | Leuciscidae | Tahtali Dam Lake | Turkey | Demir & Karakişi (2014) |
|  | *Chondrostoma orientale* | Leuciscidae | Kor River Basin | Iran | Sayyadzadeh & Joladeh Roudbar (2014) |
|  | *Chondrostoma regium* | Leuciscidae | Kaftar Lake | Iran | Barzegar & Jalali (2000) |
|  |  |  | Tigris River, Baghdad Province | Iraq | Balasem *et al*. (1993) |
|  |  |  | Greater Zab River | Iraq | Al-Marjan (2016) |
|  | *Cobitis calderoni* | Cobitidae | Tormes River | Spain | Sánchez-Hernández (2017) |
|  | *Ctenopharyngodon idella* | Xenocyprididae | Adana DSI | Turkey | Innal (2020) |
|  |  |  | all regions | Iran | Jalali (1998) |
|  |  |  | North of Iran and Khuzestan | Iran | Jalali (1998) |
|  |  |  | Sefid-rud River | Iran | Naem *et al*. (2000) |
|  |  |  | Zarineh-rud River | Iran | Jalali (1998) |
|  |  |  | Hamoon Lagoon | Iran | Molnar & Baska (1993) |
|  |  |  | Mahabad Reservoir | Iran | Mirhashemi Nasab & Pazooki (2003) |
|  |  |  | Zarivar Lake | Iran | Molnar (1990), Jalali & Barzegar (2006) |
|  |  |  | Shushtar, Khuzestan province | Iran | Manshadi *et al* (2024) |
|  |  |  | import | Egypt | Faisal *et al*. (1988) |
|  |  |  | Fish ponds and farms, Baghdad Province | Iraq | Al-Hamed & Hermiz (1973), Mhaisen & Abdul-Ameer (2021) |
|  |  |  | Fish ponds and farms, Babylon Province | Iraq | Ali *et al*. (1988b), Mhaisen *et al*. (1990), Al-Zubaidy (1998), Muhammed (2000), Al-Jadoaa (2002) |
|  |  |  | Fish ponds and farms, Duhok Province | Iraq | Ali (2002) |
|  |  |  | Fish ponds and farms, Basrah Province | Iraq | Mhaisen & Abdul-Ameer (2021) |
|  | ***Cyprinion kais*** | **Cyprinidae** | **Sinanköy, Akçayır stream (inflow of Batman river), Tigris basin** | **Turkey** | **present study** |
|  | *Cyprinion macrostomus* | Cyprinidae | Beni suif fish hatchery | Egypt | Abd El-Galil *et al*. (2012) |
|  |  |  | Fish ponds and farms, Baghdad Province | Iraq | Mohammad-Ali *et al*. (1999) |
|  |  |  | Dokan Lake | Iraq | Abdullah & Ismail (2004) |
|  |  |  | Euphrates River, Babylon Province | Iraq | Hussain (2007) |
|  | *Cyprinion microphthalmum* | Cyprinidae | Mashkid River | Iraq | Malekzehi *et al*. (2014) |
|  | *Cyprinus carpio* | Cyprinidae | Garip village, Bingöl | Turkey | Koyun *et al*. (2015) |
|  |  |  | Adana DSI | Turkey | Innal (2020) |
|  |  |  | Fish Research Unit, Cukurova | Turkey | Şahan & Duman (2010) |
|  |  |  | Karacaören II, Dam Lake | Turkey | Innal (2020) |
|  |  |  | Tahtalı Dam Lake | Turkey | Karakişi & Demir (2012) |
|  |  |  | Mazandaran fish hatcheries | Iran | Barzegar & Jalali (2009) |
|  |  |  | Dorudzan | Iran | Sayyadzadeh *et al.* (2016) |
|  |  |  | ponds around Mashhad | Iran | Borji *et al.* (2012) |
|  |  |  | Choghakhor Lagoon | Iran | Raissy *et al*. (2013) |
|  |  |  | Mashhad | Iran | Nematollahi *et al*. (2013) |
|  |  |  | Anzali Lagoon | Iran | Asadzadeh Mangili *et al*. (2000) |
|  |  |  | Hamoon Lagoon | Iran | Sharif Rohani (1994) |
|  |  |  | Kaftar Lake | Iran | Barzegar & Jalali (2000) |
|  |  |  | Vahdat Reservoir | Iran | Barzegar & Jalali (2009) |
|  |  |  | Zarivar Lake | Iran | Jalali & Barzegar (2006) |
|  |  |  | Llobregat River | Spain | Gutiérrez-Galindo & Lacasa-Millán (2005) |
|  |  |  | Retiro pond | Spain | Almeida *et al*. (2008) |
|  |  |  | north-eastern Spain | Spain | Maceda-Veiga *et al*. (2019) |
|  |  |  | canal near Mantova | Italy | Fratello & Sabatini (1972) |
|  |  |  | Massaciuccoli district, Tuscany | Italy | Macchioni *et al*., (2015) |
|  |  |  | Souk-Ahras Mila | Algeria | Boucenna *et al*. (2015) |
|  |  |  | Poljana | Croatia | Dolšak (2018), Pofuk (2021) |
|  |  |  | Bardača, Saničani, Prnjavor | Bosnia and Herzegovina | Jažić (1995) |
|  |  |  | NA | Albania | Sadikaj *et al*. (2014) |
|  |  |  | import | Egypt | Faisal *et al*. (1988) |
|  |  |  | Abbassa | Egypt | Saleh & Mohamed (2002) |
|  |  |  | Beni suif fish hatchery | Egypt | Abd El-Galil *et al*. (2012) |
|  |  |  | fish farms at Sharkia, Beni Seuif and Kafr Elshaikh | Egypt | El-Khatib & El-Hady (2012) |
|  |  |  | Fish ponds and farms, Baghdad Province | Iraq | Al-Hamed & Hermiz (1973), Khalifa *et al*. (1978), Khalifa (1982), Ali & Shaaban (1984), Ali (1985, 1986), Al-Shaikh & Al-Sardee (1993), Mhaisen *et al*. (1993a, b), Al-Aubaidi *et al*. (1999), Mohammad-Ali *et al*. (1999), Salih *et al*. (2000), Al-Tamimi (2001), Al-Nasiri *et al*. (2002), Asmar *et al*. (2004), Sadek *et al*. (2006), Mhaisen & Abdul-Ameer (2021) |
|  |  |  | Fish ponds and farms, Basrah Province | Iraq | Mhaisen (1982), Abed (2005), Al-Niaeem (2006), Al-Salim *et al*. (2007), Bannai *et al*. (2007), Jassim (2007), Hussein *et al*. (2011), Mhaisen & Abdul-Ameer (2021) |
|  |  |  | Fish ponds and farms, Babylon Province | Iraq | Mhaisen (1983), Ali *et al*. (1988b), Mhaisen & Abul-Eis (1991), Al-Zubaidy (1998), Muhammed (2000), Al-Jubory (2009), Kadim & Al-Zubaidy (2009), Yaseen *et al*. (2009), Hussain *et al*. (2011), Hussain (2017), Mhaisen & Al-Daraji (2023) |
|  |  |  | Habbaniyah Lake in Al-Anbar province | Iraq | Ali *et al*. (1988a) |
|  |  |  | Fish ponds and farms, Salah Al-Din Province | Iraq | Khalifa (1989) |
|  |  |  | Dokan Lake | Iraq | Abdullah & Ismail (2004), Abdullah & Rasheed (2004), Mhaisen & Al-Daraji (2023) |
|  |  |  | Garmat Ali river | Iraq | Khamees (1997) |
|  |  |  | Fish ponds and farms, Duhok Province | Iraq | Ali (2002), Mhaisen & Al-Daraji (2023) |
|  |  |  | Euphrates River, Al-Diwaniah Province | Iraq | Al-Jadoaa (2002), Yassin (2010) |
|  |  |  | some drainage networks, Baghdad Province | Iraq | Mhaisen *et al*. (2003) |
|  |  |  | Mortuka stream at Erbil Province | Iraq | Abdullah (2004) |
|  |  |  | Darbandikhan Lake | Iraq | Abdullah (2005, 2013), Abdullah &Abdullah (2015a, b) |
|  |  |  | Fish ponds and farms, Nineveh Province | Iraq | Daoud *et al*. (2005), Al-Hamdane & Azziz (2006) |
|  |  |  | Fish ponds and farms, Erbil Province | Iraq | Al-Marjan & Abdullah (2008, 2009), Mhaisen & Abdul-Ameer (2021) |
|  |  |  | Al-Mussayab fish farm | Iraq | Al-Rubaie *et al*. (2007) |
|  |  |  | Euphrates River, Babylon Province | Iraq | Al-Zubaidy (2007) |
|  |  |  | Garmat Ali river | Iraq | Al-Saboonchi *et al*. (2009), Mhaisen & Abdul-Ameer (2021) |
|  |  |  | Tigris River, Nineveh Province | Iraq | Al-Niaeemi (2011) |
|  |  |  | Lesser Zab River | Iraq | Mama & Abdullah (2012, 2013), Mhaisen & Abdul-Ameer (2021) |
|  |  |  | some pools in Basrah Province | Iraq | Al-Ali *et al*. (2013) |
|  |  |  | Shatt Al-Arab river | Iraq | Eassa *et al*. (2014), Mhaisen & Abdul-Ameer (2021) |
|  |  |  | Euphrates River, Al-Muthanna Province | Iraq | Al-Helli (2019) |
|  |  |  | floating cages at Euphrates river at Al-Najaf Al-Ashraf province | Iraq | Al-Salami (2019) |
|  |  |  | fish farms | Syria | Samman (1989) |
|  | *Dicentrarchus labrax* | Moronidae | Kafr El-Sheikh Governorate | Egypt | Khoris & Bileh (2024) |
|  | *Esmaeilius sophiae* | Aphaniidae | Ghadamgah Spring | Iran | Rahimi *et al*. (2013) |
|  | *Esmaeilius vladykovi* | Aphaniidae | Tarska Bay | Croatia | Čož-Rakovac *et al*. (2002) |
|  |  |  | Behesht abad River | Iran | Barzegar *et al*. (2004) |
|  | *Esox lucius* | Esocidae | Anzali Lagoon | Iran | Asadzadeh Mangili *et al*. (2000) |
|  | *Gambusia affinis* | Poeciliidae | Sapanca Lake | Turkey | Soylu (1990) |
|  | *Gambusia holbrooki* | Poeciliidae | pond near Modena, Italy | Italy | Fratello & Sabatini (1972) |
|  |  |  | Kundu River Estuary | Turkey | Innal & Avenant-Oldewage (2012) |
|  |  |  | Fish pond in north of Iran | Iran | Mohkayer (1985) |
|  |  |  | Fish ponds and farms, Basrah Province | Iraq | Mhaisen (1986), Al-Niaeem (2006), Al-Salim *et al*. (2007), Bannai *et al*. (2007) |
|  |  |  | Fish ponds and farms, Baghdad Province | Iraq | Mhaisen & Abdul-Ameer (2021) |
|  |  |  | Al-Hammar marsh in Basrah province | Iraq | Jarallah *et al*. (2005) |
|  |  |  | Euphrates River, Babylon Province | Iraq | Hussain (2008) |
|  |  |  | Garmat Ali river | Iraq | Mhaisen & Abdul-Ameer (2021) |
|  |  |  | some pools in Basrah Province | Iraq | Al-Ali *et al*. (2013) |
|  | *Garra rufa* | Cyprinidae | Euphrates River, Al-Muthanna Province | Iraq | Al-Helli (2019) |
|  | *Glyptothorax silviae* | Sisoridae | Saimareh River | Iran | Sayyadzadeh *et al*. (2014) |
|  | *Gobio lozanoi* | Gobionidae | Düger Creek | Turkey | Innal *et al*. (2017) |
|  | *Gobio* sp. | Gobionidae | Duero River | Spain | llán Aguirre (2012) |
|  |  |  | Hamoon Lagoon | Iran | Molnar (1990) |
|  | *Gonorhynchus diplochilus* | Cyprinidae | Mashkid River | Iran | Malekzehi *et al*. (2014) |
|  | *Hemiculter leucisculus* | Xenocyprididae | Darbandikhan Lake | Iraq | Abdullah & Abdullah (2015a, b), Mhaisen & Abdul-Ameer (2021) |
|  | *Heteropneustes fossilis* | Heteropneustidae | Fish ponds and farms, Baghdad Province | Iraq | Salih *et al*. (2000) |
|  | *Hypophthalmichthys molitrix* | Xenocyprididae | north-eastern Spain | Spain | Maceda-Veiga *et al*. (2019) |
|  |  |  | Mazandaran fish hatcheries | Iran | Barzegar & Jalali (2009) |
|  |  |  | all regions | Iran | Jalali (1998) |
|  |  |  | Anzali Lagoon | Iran | Asadzadeh Mangili *et al*. (2000) |
|  |  |  | Choghakhour Lagoon | Iran | Sharif Rohani (1994) |
|  |  |  | Mashhad | Iran | Raissy *et al*. (2013) |
|  |  |  | Manzala area | Egypt | Nofal *et al*. (2016) |
|  |  |  | Fish ponds and farms, Baghdad Province | Iraq | Al-Hamed & Hermiz (1973), Mhaisen & Abdul-Ameer (2021) |
|  |  |  | Fish ponds and farms, Babylon Province | Iraq | Ali *et al*. (1989), Al-Zubaidy (1998), Muhammed (2000) |
|  |  |  | Fish ponds and farms, Duhok Province | Iraq | Ali (2002) |
|  |  |  | Euphrates River, Al-Diwaniah Province | Iraq | Al-Jadoaa (2002) |
|  |  |  | Fish ponds and farms, Basrah Province | Iraq | Jassim (2007) |
|  | *Hypophthalmichthys nobilis* | Xenocyprididae | Beni suif fish hatchery | Egypt | Abd El-Galil *et al*. (2012) |
|  |  |  | North of Iran and Khuzestan | Iran | Jalali (1998) |
|  | *Knipowitschia panizzae* | Gobionidae | Lake Trasimeno | Italy | Ahnelt *et al*. (2018) |
|  | *Lepomis gibbosus* | Centrarchidae | Gala Lake | Turkey | Soylu (2014) |
|  |  |  | Dipsiz-Çine Stream | Turkey | Kvach *et al*. (2024) |
|  |  |  | Degirmenköy Reservoir | Turkey | Kvach *et al*. (2024) |
|  |  |  | north-eastern Spain | Spain | Maceda-Veiga *et al*. (2019) |
|  |  |  | Lake Trasimeno | Italy | Fratello & Sabatini (1972) |
|  |  |  | canal near Mantova | Italy | Fratello & Sabatini (1972) |
|  |  |  | Massaciuccoli district, Tuscany | Italy | Macchioni *et al*., (2015) |
|  |  |  | Draa and Ghir basins | Morocco | Clavero *et al*. (2015) |
|  | *Leuciscus vorax* | Leuciscidae | Karun River | Iran | Molnar & Baska (1993) |
|  |  |  | Fish ponds and farms, Baghdad Province | Iraq | Khalifa *et al*. (1978), Mhaisen & Abdul-Ameer (2021) |
|  |  |  | Euphrates River, Al-Diwaniah Province | Iraq | Al-Jadoaa (2002) |
|  |  |  | Euphrates River, Al-Muthanna Province | Iraq | Al-Helli (2019) |
|  | *Leucos aula* | Leuciscidae | Massaciuccoli district, Tuscany | Italy | Macchioni *et al*., (2015) |
|  |  |  | **Grabovač reservoir** | **Croatia** | **present study** |
|  | *Luciobarbus bocagei* | Cyprinidae | Tormes River | Spain | Simon Vicente *et al*. (1973), Sánchez-Hernández (2017) |
|  |  |  | Duero River | Spain | llán Aguirre (2012) |
|  | *Luciobarbus callensis* | Cyprinidae | Beni-Haroun dam, Mila | Algeria | Berrouk *et al*. (2022) |
|  |  |  | Lake Oubeïra | Algeria | Berrouk *et al*. (2018) |
|  |  |  | Bounamoussa river | Algeria | Berrouk *et al*. (2018) |
|  | *Luciobarbus comizo* | Cyprinidae | Guadiana River | Spain | Perez-Bote (2010) |
|  | *Luciobarbus esocinus* | Cyprinidae | Fish ponds and farms, Baghdad Province | Iraq | Khalifa *et al*. (1978) |
|  |  |  | Hemrin dam lake in Diyala province | Iraq | Balasem *et al*. (2000) |
|  |  |  | Dokan Lake | Iraq | Abdullah & Ismail (2004) |
|  |  |  | Darbandikhan Lake | Iraq | Abdullah & Abdullah (2015a, b), Mhaisen & Abdul-Ameer (2021) |
|  | *Luciobarbus graellsii* | Cyprinidae | Llobregat River | Spain | Gutiérrez-Galindo & Lacasa-Millán (2005) |
|  |  |  | north-eastern Spain | Spain | Maceda-Veiga *et al*. (2019) |
|  | ***Luciobarbus guiraonis*** | **Cyprinidae** | **Magro river (1)** | **Spain** | **present study** |
|  |  |  | **Magro river (2)** | **Spain** | **present study** |
|  |  |  | **Turia river** | **Spain** | **present study** |
|  | *Luciobarbus sclateri* | Cyprinidae | Guadiana River | Spain | Pérez-Bote (2000) |
|  | *Luciobarbus schejch* | Cyprinidae | Armand River | Iran | Raissy & Ansari (2012) |
|  |  |  | Vahdat Reservoir | Iran | Jalali & Barzegar (2005) |
|  |  |  | Dokan Lake | Iraq | Abdullah & Ismail (2004) |
|  |  |  | Darbandikhan Lake | Iraq | Abdullah & Abdullah (2015a, b), Mhaisen & Abdul-Ameer (2021) |
|  |  |  | Fish ponds and farms, Baghdad Province | Iraq | Al-Hamed & Hermiz (1973), Khalifa (1989), Mhaisen & Abdul-Ameer (2021) |
|  |  |  | Euphrates River, Al-Diwaniah Province | Iraq | Al-Jadoaa (2002) |
|  |  |  | Dokan Lake | Iraq | Abdullah & Ismail (2004) |
|  | *Luciobarbus* sp. | Cyprinidae | Draa and Ghir basins | Morocco | Clavero *et al*. (2015) |
|  | *Mastacembelus simack* | Mastacembelidae | Zarivar Lake | Iran | Jalali & Barzegar (2006); Jalali *et al*. (2008) |
|  |  |  | Tigris River, Baghdad Province | Iraq | Al-Janabi (2010a) |
|  | *Mesopotamichthys sharpeyi* | Cyprinidae | Fish ponds and farms, Baghdad Province | Iraq | Khalifa *et al*. (1978), Khalifa (1989), Mhaisen & Abdul-Ameer (2021) |
|  |  |  | Garmat Ali river | Iraq | Khamees (1997) |
|  |  |  | Euphrates River, Al-Diwaniah Province | Iraq | Al-Jadoaa (2002) |
|  | *Mugil capito* | Mugilidae | Lake Manzala | Egypt | Marzouk *et al.* (2002) |
|  | *Mystus pelusius* | Bagridae | Euphrates River, Al-Diwaniah Province | Iraq | Al-Jadoaa (2002) |
|  | *Ninnigobius canestrinii* | Gobionidae | Lake Trasimeno | Italy | Ahnelt *et al*. (2018) |
|  | *Oncorhynchus mykiss* | Salmonidae | aquaculture | Turkey | Tokşen *et al*. (2015) |
|  |  |  | Sarı Mehmet Dam Lake | Turkey | Urku & Onalan (2018) |
|  |  |  | Sücüllü Dam Lake | Turkey | Akçimen *et al*. (2012) |
|  |  |  | north-eastern Spain | Spain | Maceda-Veiga *et al*. (2019) |
|  |  |  | Sistan Chah nimeh | Iran | Sharif Rohani (1994) |
|  |  |  | Chaharmahal and Bakhtiari province | Iran | Momeni *et al*. (2024) |
|  | *Oreochromis aureus* | Cichlidae | El-Abbassa fish farms | Egypt | Al Malki (2021) |
|  | *Oreochromis niloticus* | Cichlidae | Lake Manzala | Egypt | Ibrahim & Soliman (2011) |
|  |  |  | El-Abbassa fish farms | Egypt | Al Malki (2021) |
|  | *Oxynoemacheilus anatolicus* | Nemacheilidae | Düğer Creek, Burdur | Turkey | Innal *et al*. (2017) |
|  | *Paracapoeta trutta* | Cyprinidae | Vahdat Reservoir | Iran | Jalali & Barzegar (2005) |
|  | ***Parachondrostoma arrigonis*** | **Leuciscidae** | **Magro River (2)** | **Spain** | **present study** |
|  | *Parachondrostoma miegii* | Leuciscidae | River Ebro | Spain | Sterling *et al*. (1995) |
|  |  |  | north-eastern Spain | Spain | Maceda-Veiga *et al*. (2019) |
|  | *Phoxinus* sp. | Leuciscidae | north-eastern Spain | Spain | Maceda-Veiga *et al*. (2019) |
|  | *Planiliza abu* | Mugilidae | Fish ponds and farms, Babylon Province | Iraq | Mhaisen *et al*. (1989) |
|  |  |  | Fish ponds and farms, Baghdad Province | Iraq | Mohammad-Ali *et al*. (1999) |
|  |  |  | Euphrates River, Babylon Province | Iraq | Al-Zubaidy (2007) |
|  |  |  | some drainage networks, Babylon Province | Iraq | Mhaisen & Abdul-Ameer (2021) |
|  | *Planiliza subviridis* | Mugilidae | Shatt Al-Arab river | Iraq | Mhaisen & Abdul-Ameer (2021) |
|  | *Poecilia latipinna* | Poeciliidae | aquaculture, Mersin | Turkey | Koyuncu (2002) |
|  |  |  | Esfahan province | Iran | Rahmati-Holasoo *et al*. (2023) |
|  | *Poecilia reticulata* | Poeciliidae | aquaculture, Mersin | Turkey | Koyuncu (2002) |
|  |  |  | El-Taif | Saudi Arabia | Ghobashy *et al*. (2018) |
|  |  |  | Esfahan province | Iran | Rahmati-Holasoo *et al*. (2023) |
|  | *Poecilia sphenops* | Poeciliidae | some fish aquaria in Al-Najaf Al-Ashraf province | Iraq | Naji (2010) |
|  |  |  | Al-Hammar marsh in Basrah province | Iraq | Abbas *et al*. (2014) |
|  | *Pomatoschistus anatoliae* | Gobionidae | Karpuzçay Creek | Turkey | Innal *et al*. (2021) |
|  | *Pseudochondrostoma duriense* | Leuciscidae | Duero River | Spain | llán Aguirre (2012) |
|  |  |  | Tormes River | Spain | Sánchez-Hernández (2017) |
|  | *Pseudochondrostoma polylepis* | Leuciscidae | Arrocampo reservoir | Spain | Moreno *et al*. (1986) |
|  |  |  | **Colares** | **Portugal** | **present study** |
|  | *Pseudochondrostoma willkommii* | Leuciscidae | Guadiana River | Spain | Perez-Bote (2000) |
|  | *Pseudophoxinus burduricus* | Leuciscidae | Düğer Creek, Burdur | Turkey | Innal *et al*. (2017) |
|  |  |  | Degirmendere Creek | Turkey | Innal (2020) |
|  | *Pseudophoxinus egridiri* | Leuciscidae | Eğirdir Lake, Isparta | Turkey | Akcimen *et al*. (2018) |
|  | *Pseudophoxinus zekayi* | Leuciscidae | Ceyhan River | Turkey | Innal (2020) |
|  | *Pseudorasbora parva* | Gobionidae | Onaç Reservoir | Turkey | Innal (2020) |
|  |  |  | Kaftar Lake | Iran | Barzegar & Jalali (2000) |
|  |  |  | Massaciuccoli district, Tuscany | Italy | Macchioni *et al*., (2015) |
|  | *Pterophyllum scalare* | Cichlidae | aquaculture, Ankara | Turkey | Innal (2020) |
|  | *Rhodeus sericeus* | Acheilognathidae | aquaculture, Ankara | Turkey | Innal (2020) |
|  | *Rutilus rutilus* | Leuciscidae | Boojagh Lagoon | Iran | Khara *et al*. (2005, 2011) |
|  | *Salariopsis fluviatilis* | Blenniidae | north-eastern Spain | Spain | Maceda-Veiga *et al*. (2019) |
|  | *Salmo trutta* | Salmonidae | river Minho catchment | Portugal | Bao *et al*. (2016) |
|  |  |  | Tormes River | Spain | Sánchez-Hernández (2017) |
|  |  |  | north-eastern Spain | Spain | Maceda-Veiga *et al*. (2019) |
|  | *Scardinius erythrophthalmus* | Leuciscidae | north-eastern Spain | Spain | Maceda-Veiga *et al*. (2019) |
|  |  |  | Massaciuccoli district, Tuscany | Italy | Macchioni *et al*., (2015) |
|  | *Schizocypris altidorsalis* | Cyprinidae | Hamoon Lagoon | Iran | Sharif Rohani (1994) |
|  |  |  | Chahnimeh lakes and Hamoun wetland | Iran | Elahi Moghadam *et al.* (2014) |
|  | *Schizothorax* sp. | Cyprinidae | Hamoon Lagoon | Iran | Molnar (1990) |
|  | *Schizothorax zarudnyi* | Cyprinidae | Hamoon Lagoon | Iran | Sharif Rohani (1994) |
|  |  |  | Chah nimeh and Zahak Dam | Iran | Elahi Moghadam (2010) |
|  | *Silurus triostegus* | Siluridae | Sapanca Lake | Turkey | Soylu (1990) |
|  |  |  | Zarineh-rud River | Iran | Jalali (1998) |
|  |  |  | Anzali Lagoon | Iran | Daghigh Roohi *et al.* (2014) |
|  |  |  | Shatt Al-Arab river | Iraq | Mhaisen & Abdul-Ameer (2021) |
|  |  |  | Euphrates River, Al-Muthanna Province | Iraq | Al-Helli (2019) |
|  | *Squalius alburnoides* | Leuciscidae | Guadiana River, Spain | Spain | Pérez-Bote (2000) |
|  | *Squalius carolitertii* | Leuciscidae | Tormes River, Spain | Spain | Sánchez-Hernández (2017) |
|  | *Squalius cephalus* | Leuciscidae | Üzümlü pond, Konya | Turkey | Erbatur *et al*. (2018) |
|  |  |  | Khandaghloo River | Iran | Pazooki *et al* (2005) |
|  |  |  | Mahabad Reservoir | Iran | Mirhashemi Nasab & Pazooki (2003) |
|  |  |  | Frio River | Portugal | Saraiva & Valente (1988) |
|  |  |  | Llobregat River | Spain | Gutiérrez-Galindo & Lacasa-Millán (2005) |
|  | *Squalius laietanus* | Leuciscidae | north-eastern Spain | Spain | Maceda-Veiga *et al*. (2019) |
|  | *Squalius lepidus* | Leuciscidae | Dokan Lake | Iraq | Abdullah & Ismail (2004), Abdullah & Rasheed (2004), Mhaisen & Abdul-Ameer (2021) |
|  | ***Squalius pyrenaicus*** | **Leuciscidae** | **Peraleda de Zancejo, Zujar river** | **Spain** | **present study** |
|  | ***Squalius tenellus*** | **Leuciscidae** | **Šujica, Šujičko Polje** | **Bosnia and Herzegovina** | **present study** |
|  | ***Squalius torgalensis*** | **Leuciscidae** | **Torgal river, Mira basin** | **Portugal** | **present study** |
|  | ***Squalius valentinus*** | **Leuciscidae** | **Magro River (2)** | **Spain** | **present study** |
|  | *Tinca tinca* | Tincidae | Seyhan Dam Lake, Adana | Turkey | Innal (2020) |
|  |  |  | Gölhisar Lake | Turkey | Innal (2020) |
|  |  |  | Anzali Lagoon | Iran | Asadzadeh Mangili *et al*. (2000) |
|  |  |  | Boojagh Wetland | Iran | Khara *et al*. (2011) |
|  | *Xiphophorus helleri* | Poeciliidae | aquaculture, Mersin | Turkey | Koyuncu (2002) |
|  | *Xiphophorus maculatus* | Poeciliidae | aquaculture, Mersin | Turkey | Koyuncu (2002) |
|  | unknown host | - | Volvi | Greece | Zarfdjoam & Economidis (1989) |
|  | unknown host | - | Fish ponds and farms, Baghdad Province | Iraq | Khalifa (1982) |
|  | unknown host | - | Shatt Al-Arab river | Iraq | Al-Niaeem *et al*. (2015) |
| *Lernaea oryzophila* Monod, 1932 | *Cyprinus carpio* | Cyprinidae | Fish ponds and farms, Baghdad Province | Iraq | Al-Nasiri *et al*. (2001) |
| *Lernaea* sp. | *Barbus* sp. | Cyprinidae | El Ghaicha dam (Laghouat) | Algeria | Berrouk *et al*. (2018) |
|  | *Carassius auratus* | Cyprinidae | unknown fish pond | Iraq | Al-Tayyar *et al*. (2011) |
|  | *Ctenopharyngodon idella* | Xenocyprididae | fish ponds and farms, Al-Diwanizah Province | Iraq | Abd & Abdul wahab (2011) |
|  |  |  | fish ponds and farms, Suliemaniya | Iraq | Abdulrahman *et al*. (2020) |
|  | *Cyprinus carpio* | Cyprinidae | Esla River | Spain | Álvarez Pellitero *et al*. (1979) |
|  |  |  | fish ponds and farms, Al-Diwanizah Province | Iraq | Abd & Abdul wahab (2011) |
|  |  |  | fish ponds and farms, Basrah | Iraq | Ahmed & Ali (2013) |
|  |  |  | Euphrates River, Al-Muthanna Province | Iraq | Al-Helli (2019) |
|  |  |  | fish ponds and farms, Suliemaniya | Iraq | Abdulrahman *et al*. (2020) |
|  |  |  | Al-Mashab and Harerr marshes in Basrah province | Iraq | Jarallah *et al*. (2024) |
|  | *Gambusia holbrooki* | Poeciliidae | north-eastern Spain | Spain | Maceda-Veiga *et al*. (2019) |
|  | *Hypophthalmichthys molitrix* | Xenocyprididae | fish ponds and farms, Al-Diwanizah Province | Iraq | Abd & Abdul wahab (2011) |
|  |  |  | fish ponds and farms, Suliemaniya | Iraq | Abdulrahman *et al*. (2020) |
|  | *Mullus surmuletus* | Mullidae | Aegean Sea | Turkey | Oguz & Öktener (2007) |
|  | *Poecilia sphenops* | Poeciliidae | unknown fish pond | Iraq | Al-Tayyar *et al*. (2011) |
|  | *Silurus triostegus* | Siluridae | Euphrates River, Al-Muthanna Province | Itraq | Al-Helli (2019) |
|  | *Squalius cephalus* | Leuciscidae | Esla River | Spain | Álvarez Pellitero *et al*. (1979) |
|  | *Trichopodus trichopterus* | Osphronemidae | unknown fish pond | Iraq | Al-Tayyar *et al*. (2011) |
|  | zooplankton | - | NA | Albania | Mancinelli *et al*. (2019) |
| ***Pseudolamproglena* Boxshall, 1976** |  |  |  |  |  |
| *Pseudolamproglena annulata* Boxshall, 1976 | *Capoeta umbla* | Cyprinidae | Bahdinan River at Erbil Province | Iraq | Bilal & Abdullah (2008), Mhaisen *et al*. (2024) |
|  | *Carasobarbus luteus* | Cyprinidae | Tigris River, Salah Al-Din Province | Iraq | Al-Jawda *et al*. (2000), Esmaeel *et al*. (2023), Mhaisen *et al*. (2024) |
|  |  |  | Greater Zab River | Iraq | Abdullah (2002), Abdullah & Mhaisen (2006), Muhammad *et al*. (2013), Mhaisen *et al*. (2024) |
|  |  |  | Dokan Lake | Iraq | Abdullah & Rasheed (2004), Mhaisen *et al*. (2024) |
|  |  |  | Tigris River, Nineveh Province | Iraq | Rahemo & Al-Kallak (1998) |
|  |  |  | Darbandikhan Lake | Iraq | Abdullah (2005) |
|  |  |  | Euphrates River, Al-Anbar Province | Iraq | Mhaisen *et al*. (2024) |
|  | *Chondrostoma regium* | Leuciscidae | Euphrates River, Al-Anbar Province | Iraq | Mhaisen *et al*. (2024) |
|  | *Cyprinion kais* | Cyprinidae | Euphrates River, Al-Anbar Province | Iraq | Mhaisen *et al*. (2024) |
|  | *Cyprinion macrostomus* | Cyprinidae | Tigris River, Nineveh Province | Iraq | Boxshall (1976), Kasim & Rahemo (1981) |
|  |  |  | Tigris River, Salah Al-Din Province | Iraq | Mhaisen *et al*. (2024) |
|  |  |  | Greater Zab River | Iraq | Abdullah (2002), Abdullah & Mhaisen (2006), Mhaisen *et al*. (2024) |
|  |  |  | Dokan Lake | Iraq | Mhaisen & Al-Daraji (2023) |
|  |  |  | Bahdinan River at Erbil Province | Iraq | Bilal & Abdullah (2008), Mhaisen *et al*. (2024) |
|  |  |  | Darbandikhan Lake | Iraq | Abdullah & Abdullah (2015a, b, c), Mhaisen *et al*. (2024) |
|  |  |  | Euphrates River, Al-Anbar Province | Iraq | Mhaisen *et al*. (2024) |
|  | *Cyprinus carpio* | Cyprinidae | Dokan Lake | Iraq | Abdullah & Rasheed (2004), Mhaisen & Al-Daraji (2023) |
|  |  |  | Tigris River, Salah Al-Din Province | Iraq | Kamil *et al*. (2022), Esmaeel *et al*. (2023) |
|  | *Garra rufa* | Cyprinidae | Tigris River, Nineveh Province | Iraq | Rahemo (1995) |
|  | *Garra variabilis* | Cyprinidae | Euphrates River, Al-Anbar Province | Iraq | Mhaisen *et al*. (2024) |
|  | *Leuciscus vorax* | Leuciscidae | Tigris River, Baghdad Province | Iraq | Adday *et al*. (1999) |
|  | *Luciobarbus esocinus* | Cyprinidae | Mosul Dam Lake | Iraq | Rahemo & Ami (2013), Rahemo *et al*. (2013) |
|  | *Luciobarbus schejch* | Cyprinidae | Bahdinan River at Erbil Province | Iraq | Bilal & Abdullah (2008), Mhaisen *et al*. (2024) |
|  | *Planiliza abu* | Mugilidae | Tigris River, Salah Al-Din Province | Iraq | Esmaeel *et al*. (2023) |
| *Pseudolamproglena boxshalli* Al-Nasiri, Ho & Mhaisen, 2012 | *Carasobarbus luteus* | Cyprinidae | Tigris River, Salah Al-Din Province | Iraq | Mhaisen *et al*. (2024) |
|  |  |  | Euphrates River, Al-Anbar Province | Iraq | Mhaisen *et al*. (2024) |
|  | *Cyprinion macrostomus* | Cyprinidae | Tigris River, Salah Al-Din Province | Iraq | Al-Nasiri *et al*. (2012), Mhaisen *et al*. (2024) |
|  |  |  | Euphrates River, Al-Anbar Province | Iraq | Mhaisen *et al*. (2024) |
|  | *Cyprinus carpio* | Cyprinidae | Tigris River, Salah Al-Din Province | Iraq | Mhaisen *et al*. (2024) |
|  | *Luciobarbus xanthopterus* | Cyprinidae | Tigris River, Salah Al-Din Province | Iraq | Mhaisen *et al*. (2024) |
|  | *Paracapoeta trutta* | Cyprinidae | Tigris River, Salah Al-Din Province | Iraq | Mhaisen *et al*. (2024) |
| ***Pseudolamproglena zahrziensis* n. sp.** | ***Carasobarbus luteus*** | **Cyprinidae** | **Zahrzi, Tabin River** | **Iraq** | **present study** |
|  |  |  | **Du Choman, Aw-e Shiler River** | **Iraq** | **present study** |
|  |  |  | **Grdi Go, Zalm Stream** | **Iraq** | **present study** |

**Supplementary Table S2:** List of all collection localities with coordinates

| **Country** | **ID** | **Locality** | **Coordinates** |
| --- | --- | --- | --- |
| Portugal | P1 | Alcabrichel | 39°08'51.33"N 09°14'29.14"W |
|  | P2 | Alcoa, Fervenca | 39°34'00.94"N 08°59'20.34"W |
|  | P3 | Arunca, Mondego basin (Vermoil) | 39°51'04.61"N 08°39'19.22"W |
|  | P4 | Colares | 38°47'53.37"N 09°26'14.16"W |
|  | P5 | Seixe | 37°25'22.41"N 08°44'56.42"W |
|  | P6 | Torgal river, Mira basin | 37°38'16.76"N 08°37'10.58"W |
|  | P7 | tributary of Seixe | 37°21'47.87"N 08°40'07.45"W |
| Spain | S1 | Chico River, flow of Palancia | 39°54'09.78"N 00°27'19.66"W |
|  | S2 | Magro river (1) | 39°21'25.76"N 00°39'51.76"W |
|  | S3 | Magro river (2) | 39°21'18.85"N 00°40'38.85"W |
|  | S4 | Turia river | 39°34'46.46"N 00°37'09.63"W |
|  | S5 | Benahavis, Guadalmina river | 36°31'03.45"N 05°02'25.07"W |
|  | S6 | Istán, Verde river | 36°36'04.25"N 04°56'15.02"W |
|  | S7 | Peraleda de Zaucejo, Zujar river | 38°27'12.02"N 05°31'59.67"W |
|  | S8 | near Llera, Retin river | 38°27'10.02"N 06°06'24.99"W |
|  | S9 | Valencia de las Torres, Retin river | 38°24'05.60"N 06°02'40.30"W |
|  | S10 | Tera river | 41°54'47.49"N 02°28'44.13"W |
|  | S11 | Ucero river | 41°32'49.11"N 03°04'32.50"W |
|  | S12 | Beceite, Uldemo river | 40°50'25.59"N 00°11'38.12"E |
|  | S13 | upstream Maella, Rio Matarraña | 41°06'41.00"N 00°08'05.00"E |
| Italy | I1 | canale maestro de la Chiana, Chuisa dei Capannoi, Arno basis | 43°29'31.07"N 11°48'39.09"E |
|  | I2 | Pad, Between Verona & Modena | - |
|  | I3 | Torrente Cerfone, Intoppo | 43°26'12.03"N 11°58'33.00"E |
|  | I4 | Torrente Cerfone, Le Ville | 43°28'42.00"N 12°04'25.03"E |
|  | I5 | Isorno River, Montecreteste | 46°09'05.94"N 08°19'38.41"E |
|  | I6 | Melezzo River, Masera | 46°08'00.45"N 08°19'20.51"E |
|  | I7 | Strona River, Gravellona Toce | 45°56'02.12"N 08°26'38.78"E |
|  | I8 | Carmagnola, Cave Germaire | 44°51'42.96"N 07°40'26.33"E |
| Croatia | C1 | Baštica river, below the Baštica reservoir/Grabovač reservoir | 44°11'42.37"N 15°24'32.13"E |
|  | C2 | Bribirske Mostine, Bribišnica | 43°55'28.21"N 15°48'45.07"E |
|  | C3 | Cetina river, Kosore | 43°56'29.78"N 16°26'23.37"E |
|  | C4 | Drežnica, Sušik river | 45°08'44.13"N 15°04'41.56"E |
|  | C5 | Konavočica, Grude | 42°31'33.86"N 18°22'04.16"E |
|  | C6 | Krbavsko polje, Laudonov gaj | 44°38'14.33"N 15°40'05.65"E |
|  | C7 | Lovinac, Ričica river | 44°22'44.72"N 15°40'15.87"E |
|  | C8 | Pazin, Pazinčica river | 45°14'47.92"N 13°58'10.66"E |
|  | C9 | Raša river | 45°06'15.70"N 14°01'21.03"E |
|  | C10 | Sveti Rok, Obsenica river | 44°21'03.64"N 15°40'40.00"E |
|  | C11 | Udbina, Krbava river | 44°32'32.00"N 15°46'13.02"E |
|  | C12 | Gornja Ploča, Jadova | 44°26'59.00"N 15°40'32.00"E |
|  | C13 | Lovinac, Ričica | 44°23'24.00"N 15°41'51.00"E |
|  | C14 | Čikola | 43°48'22.09"N 16°17'24.53"E |
|  | C15 | Bunić | 44°40'45.00"N 15°37'55.00"E |
| Bosnia and Herzegovina | BIH1 | Krenica lake, Drinovci | 43°22'25.00"N 17°19'59.04"E |
|  | BIH2 | Lištica, Polog | 43°20'32.09"N 17°41'37.04"E |
|  | BIH3 | Mušnica, Avtovac | 43°08'42.05"N 18°35'45.00"E |
|  | BIH4 | Nezdravica, Tihaljina | 43°19'00.05"N 17°23'20.01"E |
|  | BIH5 | Šujica, Duvansko Polje | 43°42'05.07"N 17°15'50.05"E |
|  | BIH6 | Šujica, Šujičko Polje | 43°49'41.43"N 17°10'48.20"E |
|  | BIH7 | Tihaljina, Klobuk | 43°16'17.11"N 17°26'38.57"E |
|  | BIH8 | Vrijeka, Dabarsko Polje | 43°03'32.07"N 18°14'39.04"E |
|  | BIH9 | Vrijoštica | 43°13'34.03"N 17°29'42.04"E |
|  | BIH10 | Zalomka, Nevesinjsko polje | 43°12'06.06"N 18°12'21.07"E |
|  | BIH11 | Zalomka, Ribari | 43°15'26.04"N 18°21'41.05"E |
|  | BIH12 | Bosansko Grahovo, Korana river | 44°10'37.00"N 16°23'03.61"E |
|  | BIH13 | Donja Drežnica, Drežnica river | 43°31'31.46"N 17°42'51.66"E |
|  | BIH14 | Mostarsko Blato, channel in the field | 43°19'51.03"N 17°41'34.34"E |
|  | BIH15 | Rečina river, near Jelim lake, Hutovo Blato | 43°03'39.72"N 17°48'29.30"E |
|  | BIH16 | Zagorje, Jabuke | 43°32'18.53"N 17°12'34.28"E |
| Albania | A1 | Devoli, Maliq | 40°42'57.07"N 20°40'54.06"E |
|  | A2 | Fani i Vogel, Reps | 41°52'51.01"N 20°04'44.04"E |
|  | A3 | Kiri | 42°08'56.02"N 19°39'42.01"E |
|  | A4 | Mat, Klos | 41°29'37.01"N 20°05'29.04"E |
|  | A5 | Ohrid lake | 40°59'00.66"N 20°38'23.40"E |
|  | A6 | Ohrid lake, Lin | 41°04'27.08"N 20°37'40.00"E |
|  | A7 | Osum, Vodice | 40°24'13.07"N 20°39'04.04"E |
|  | A8 | Shkumbini, Pajove | 41°03'31.07"N 19°51'47.03"E |
|  | A9 | Shkumbini, Perrenjas | 41°03'50.09"N 20°33'56.06"E |
|  | A10 | Skadar lake, Shegan | 42°16'22.09"N 19°23'39.09"E |
|  | A11 | Skadar lake, Shiroke | 42°03'24.94"N 19°28'07.05"E |
| Greece | G1 | Rihios river, Stavros | 40°40'16.34"N 23°39'50.87"E |
|  | G2 | Angitis river, Koninogia | 41°11'36.41"N 23°54'25.00"E |
|  | G3 | Gallikos, Mandres, Gallikos basin | 40°52'07.33"N 22°53'59.12"E |
|  | G4 | Vardar, Axiopolis | 40°59'28.35"N 22°33'14.49"E |
|  | G5 | Angistis, between Alistrati & Drama | 41°05'42.08"N 24°00'18.29"E |
|  | G6 | flood pools by Struma, Lithopos | 41°07'40.41"N 23°16'24.70"E |
|  | G7 | Sperchios, Ypati | 38°54'14.33"N 22°17'30.22"E |
|  | G8 | stream in Livadia, Kifisos | 38°27'02.12"N 22°53'03.02"E |
|  | G9 | Pinios, Rongia - Valamandrio | 39°33'07.85"N 21°42'08.02"E |
|  | G10 | channel near Sperchios | 38°50'54.60"N 22°25'54.46"E |
|  | G11 | Neda, Gianitsochori | 37°23'04.34"N 21°41'24.15"E |
|  | G12 | Pamisos, Vasiliko | 37°15'17.39"N 21°53'45.15"E |
|  | G13 | Evrotas, Sparti | 37°05'34.70"N 22°25'34.81"E |
|  | G14 | Pinios, Kalivakia | 37°54'31.13"N 21°32'44.26"E |
|  | G15 | Erimantos, Tripotamo | 37°52'37.07"N 21°53'15.05"E |
|  | G16 | Acheron, Gliki | 39°19'00.05"N 20°36'04.03"E |
|  | G17 | Kokitos, Pagrati | 39°26'53.02"N 20°30'03.06"E |
|  | G18 | Rivio, Amvrakia | 38°44'37.68"N 21°11'35.86"E |
|  | G19 | Trichonis lake, Panetolio | 38°35'20.19"N 21°28'02.68"E |
|  | G20 | Aoos, Kalithea | 40°01'16.67"N 20°41'40.19"E |
|  | G21 | Macropotamos river, Filiouri basin | 41°04'13.00"N 25°32'52.00"E |
|  | G22 | Pinios, Amygdalea | 39°39'49.20"N 22°13'15.47"E |
|  | G23 | Yliky Lake | 38°25'47.82"N 23°14'37.09"E |
|  | G24 | Pamisos River | 37°13'26.80"N 21°56'59.49"E |
|  | G25 | Alfios River | 37°28'40.34"N 22°03'31.96"E |
|  | G26 | Zagoritikos River, Baldouma | 39°41'46.28"N 20°59'46.00"E |
|  | G27 | Aoos | 40°01'31.39"N 20°41'48.75"E |
| Iraq | IRQ1 | Dukan Lake | 36°10'12"N 44°57'24"E |
|  | IRQ2 | Great Zab River (1) | 36°16'25"N 43°38'40"E |
|  | IRQ3 | Darbandikhan Lake | 35°07'17"N 45°43'50"E |
|  | IRQ4 | Great Zab River (2) | 36°40'03"N 44°13'45"E |
|  | IRQ5 | Dukan, Little Zab | 35°54'29"N 44°58'26"E |
|  | IRQ6 | By the road Suleymanya-Dukan | 35°52'53"N 45°00'20"E |
|  | IRQ7 | wadi Kalat Shirah, tributary of Tabin River | 35°47'3"N 44°58'43"E |
|  | IRQ8 | Kani Shok, tributary of Tabin River | 35°50'01''N 45°06'16''E |
|  | IRQ9 | Zahrzi, Tabin River | 35°48'32''N 45°01'20''E |
|  | IRQ10 | Grdi Go, Zalm Stream | 35°18'26"N 45°58'18"E |
|  | IRQ11 | Du Choman, Aw-e Shiler River | 35°45'49"N 45°27'12"E |
| Türkiye | TUR1 | Çine River, near Çiftlikköy | 37°45'48"N 27°50'03"E |
|  | TUR2 | Kamişdere stream, near Yatagan | 37°20'30"N 28°06'55"E |
|  | TUR3 | Kocaalam Deresi | 36°57'12"N 28°17'13"E |
|  | TUR4 | Çine River, near Sitmalik | 37°24'36"N 28°06'49"E |
|  | TUR5 | Porsuk Tibet | 39°46'05.7"N 30°28'52.2"E |
|  | TUR6 | Çifteler | 39°20'40.2"N 31°18'44.8"E |
|  | TUR7 | Seyitgazi | 39°21'27.5"N 30°35'37.0"E |
|  | TUR8 | Kütahya | 39°22'48.8"N 30°03'58.9"E |
|  | TUR9 | south of Mahmudyie, inflow of the lake Sapanca | 40°40'40"N 30°13'12"E |
|  | TUR10 | east of Doğançay, inflow of Sakarya | 40°37'27"N 30°21'44"E |
|  | TUR11 | Sapanca, inflow of the Lake Sapanca | 40°41'54"N 30°14'48"E |
|  | TUR12 | East of Barakfatih, Burutma kanali | 40°13'27"N 29°18'56"E |
|  | TUR13 | Büyükdoğanca, Söğütlü creek | 40°46'07"N 26°34'54"E |
|  | TUR14 | South of Yülüce, Kocadere | 40°39'56"N 26°57'48"E |
|  | TUR15 | Ardahan, Kura | 41°06'56.9"N 42°42'02.5"E |
|  | TUR16 | Aralik, Aras (channels in flood area), Kura Basin | 39°54'26"N 44°30'28"E |
|  | TUR17 | Yiğitkonağı closest village, Çakir, Kura Basin | 40°58'00.6"N 42°35'15.9"E |
|  | TUR18 | Ölçek, Ölçeksuyu, Kura Basin | 41°08'01.4"N 42°51'21.7"E |
|  | TUR19 | Gaziler stream near the mouth to Aras, Gaziler, Kura Basin | 40°06'28"N 43°27'07"E |
|  | TUR20 | Aşağıkent, Cuma stream, Euphrates Basin | 39°56'57"N 43°16'15"E |
|  | TUR21 | Kayacik, Cuma stream, Euphrates Basin | 39°53'45"N 43°10'34"E |
|  | TUR22 | Özkavak, marsh with creek in Karasu river, Euphrates Basin | 38°40'24"N 42°02'53"E |
|  | TUR23 | between Otluca and Kasımoğlu, Karasu stream, Van Lake | 38°41'25"N 43°23'53"E |
|  | TUR24 | Haydarbey (Erciş), Deliçay stream, Van Lake | 39°00'59"N 43°26'34"E |
|  | TUR25 | near Meydan village, inflow of Garzan river, Tigris Basin | 38°21'19"N 41°46'48"E |
|  | TUR26 | Sinanköy, Akçayır stream (inflow of Batman river), Tigris Basin | 37°51'56"N 40°59'21"E |
|  | TUR27 | west of Esendere, Urmia Lake | 37°42'57.5"N 44°34'27.6"E |
|  | TUR28 | Darköprü, Çelebyian stream, Tigris Basin | 38°08'01.7"N 40°49'15.3"E |
|  | TUR29 | Taşlıburç, Çağ-Çağ stream, Euphrates Basin | 37°11'56"N 41°18'28"E |
|  | TUR30 | Kelkit, Kelkit stream, Yeşilirmak Basin | 40°08'09"N 39°26'37"E |
|  | TUR31 | inflow of Iyidere, Iyidere Basin | 40°57'39"N 40°22'26"E |
|  | TUR32 | west of Ardanuç, Cehennem/Şuat/Köprüler stream, Çoruh Basin | 41°08'09"N 42°02'00"E |
|  | TUR33 | ca 5 km north of Borçka, inflow of Çoruh, Çoruh Basin | 41°24'13"N 41°41'47"E |
|  | TUR34 | Çoruh Basin | N/A |
|  | TUR35 | inflow of Zab, Tigris Basin | 37°39'59"N 43°53'07"E |
|  | TUR36 | Nilufer river, upstream the reservoir Doganci | 40°4'50.333''N 29°0'3.649''E |
|  | TUR37 | Simav river, Karacabey | 40°11'50.526''N 28°21'12.321''E |
|  | TUR38 | Koca river, Cavus | 40°03'49.997''N 27°54'5.796''E |
|  | TUR39 | Sasal stream, Kuner | 38°11'58.017''N 27°8'9.305''E |
|  | TUR40 | inflow of Gonen river, Kalkim | 39°48'22.877''N 27°13'38.097''E |
|  | TUR41 | Tuzla river, Ayvacik | 39°34'30.672''N 26°24'36.299''E |
|  | TUR42 | Gediz river, Yesilkoy | 38°36'52.175''N 27°36'38.508''E |
|  | TUR43 | between Ortabag and Diskaya | 38°37'10.125''N 28°50'22.186''E |
|  | TUR44 | Karamusa, upper Dalaman | 37°11' 3.935''N 29°45'14.272''E |
|  | TUR45 | Karamanli | 37°25'12.424''N 29°49'17.654''E |
|  | TUR46 | Kirkpinar | 37°8'35.156''N 29°55'7.362''E |
|  | TUR47 | Kovada river, Asagi Gokdere, Aksu drainage | 37°35'49.134''N 30°49'32.101''E |
|  | TUR48 | Stream in Bagilli, Kopru river drainage | 37°45'48.993''N 31°1'59.746''E |
|  | TUR49 | Kovada river, Aksu drainage | 37°32'45.847''N 30°46'50.207''E |
|  | TUR50 | Çenger stream, Çavuşköy, Kapruz drainage | 36°43'44.571''N 31°36'14.414''E |
|  | TUR51 | Ilica stream, Ilica | 36°49'6.367''N 31°21'12.061''E |
|  | TUR52 | Kirgkoz wetland | 37°6'34.565''N 30°34'47.348''E |
|  | TUR53 | Büyük Menderes, around 1 km east of Işıklı lake | 38°12'49.74"N 29°49'16.83"E |

**Supplementary Table S3:** List of parasitic copepods used for phylogenetic analyses, including their host species, collection locality, and accession numbers for partial 28S rDNA sequences from database GenBank

| **Parasite species** | **Host species** | **Host family** | **Locality** | **GB Acc. No.** | **Reference** |
| --- | --- | --- | --- | --- | --- |
| **Ergasilidae** |  |  |  |  |  |
| *Acusicola margulisae* | *Amphilophus citrinellus* | Cichlidae | Nicaragua | MN852850 | Santacruz *et al.* (2020) |
| *Dermoergasilus madagascarensis* | *Paretroplus polyactis* | Cichlidae | Canal des Pangalanes, Madagascar | PP115569 | Míč *et al*. (2024) |
| *Ergasilus anchoratus* | *Tachysurus fulvidraco* | Bagridae | Baoan Lake, China | DQ107528 | Song *et al.* (2008) |
| *Ergasilus arenalbus* | *Amblyrhynchotes honckenii* | Tetraodontidae | Breede River Estuary, Witsand, South Africa | PQ451957 | van der Spuy *et al*. (2024) |
| *Ergasilus briani* | *Misgurnus anguillicaudatus* | Cobitidae | Dangjiangkou, China | DQ107532 | Song *et al.* (2008) |
| *Ergasilus caparti* | *Spathodus erythrodon* | Cichlidae | Lake Tanganyika, Burundi | OQ407474 | Míč *et al*. (2023) |
| *Ergasilus chintensis* | *Amblyrhynchotes honckenii* | Tetraodontidae | Chintsa East, South Africa | PQ451959 | van der Spuy *et al*. (2024) |
| *Ergasilus hypomesi* | *Acanthogobius hasta* | Gobiidae | Dangjiangkou, China | DQ107539 | Song *et al.* (2008) |
| *Ergasilus kandti* | *Tylochromis polylepis* | Cichlidae | Zambia | PQ249843 | Jansen *et al*. (2024) |
| *Ergasilus macrodactylus* | *Gnathochromis permaxillaris* | Cichlidae | Lake Tanganyika, Burundi | OQ407470 | Míč *et al*. (2023) |
| *Ergasilus megacheir* | *Simochromis diagramma* | Cichlidae | Lake Tanganyika, Burundi | OQ407471 | Míč *et al*. (2023) |
| *Ergasilus mirabilis* | *Clarias gariepinus* | Clariidae | The Barotse floodplain, Zambezi, Zambia | OR449756 | Fikiye *et al*. (2023) |
| *Ergasilus parasarsi* | *Ophthalmotilapia nasuta* | Cichlidae | Lake Tanganyika, Burundi | OQ407473 | Míč *et al*. (2023) |
| *Ergasilus parasiluri* | *Silurus asotus* | Siluridae | Dangjiangkou, China | DQ107537 | Song *et al.* (2008) |
| *Ergasilus parvus* | *Spathodus erythrodon* | Cichlidae | Lake Tanganyika, Burundi | OQ407472 | Míč *et al*. (2023) |
| *Ergasilus peregrinus* | *Siniperca chuatsi* | Sinipercidae | Dangjiangkou, China | DQ107531 | Song *et al.* (2008) |
| *Ergasilus scalaris* | *Tachysurus dumerili* | Bagridae | Poyang Lake, China | DQ107538 | Song *et al.* (2008) |
| *Ergasilus sieboldi* | *Ameiurus nebulosus* | Ictaluridae | Skrecon Pond, Czech Republic | PQ100628 | Ondračková *et al*. (2025) |
|  | *Sparus aurata* | Sparidae | Egypt | ON706996 | Abou-Okada *et al*. (2023) |
| *Ergasilus* sp. (Mexico) | *Floridichthys polyommus* | Cyprinodontidae | La Carbonera lagoon, Yucatán, Mexico | OR537907 | Espínola-Novelo *et al*. (2023) |
| *Ergasilus tumidus* | *Acheilognathus taenianalis* | Acheilognathidae | Niushan Lake, China | DQ107533, DQ107535 | Song *et al.* (2008) |
| *Ergasilus wilsoni* | *-* | - | South Korea | KR048842 | Baek *et al*. (2016) |
| *Ergasilus yaluzangbus* | *Oxygymnocypris stewartii* | Cyprinidae | Lasa River, Tibet, China | DQ107540 | Song *et al.* (2008) |
| *Gamispatulus schizodontis* | *Serrasalmus maculatus* | Serrasalmidae | Pardo River, Brazil | PQ452579 | Narciso *et al*. (2024) |
| *Neoergasilus africanus* | *Clarias gariepinus* | Clariidae | Great Fish River, Eastern Cape, South Africa | PP864461 | Fikiye *et al*. (2024) |
| *Neoergasilus japonicus* | *Ameiurus nebulosus* | Ictaluridae | Elbe, Za Pilou Pond, Czech republic | PQ100638 | Ondračková *et al*. (2025) |
|  | *Lepomis gibbosus* | Centrarchidae | Babice, Moravia, Czech republic | MW810240 | Kvach *et al*. (2021) |
| *Paraergasilus brevidigitus* | *Cyprinus carpio* | Cyprinidae | Tangxun Lake, China | DQ107530 | Song *et al.* (2008) |
| *Paraergasilus longidigitus* | *Abramis brama* | Leuciscinae | Pahrbek, U Jezu, Czech Republic | MW810243 | Kvach *et al.* (2021) |
| *Paraergasilus medius* | *Ctenopharyngodon idella* | Xenocyprididae | Tangxun Lake, China | DQ107529 | Song *et al.* (2008) |
| *Rhinergasilus piranhus* | *Psalidodon bockmanni* | Characidae | Pardo River, Brazil | PQ452581 | Narciso *et al*. (2024) |
| *Sinergasilus major* | *Ctenopharyngodon idella* | Xenocyprididae | Tangxun Lake, China | DQ107524 | Song *et al.* (2008) |
|  | *Silurus glanis* | Siluridae | Danube River, Hungary | MZ047815 | Dos Santos *et al*. (2021) |
| *Sinergasilus polycolpus* | *Hypophthalmichthys molitrix* | Xenocyprididae | Tangxun Lake, China; Jingzhou, China | DQ107525 | Song *et al.* (2008) |
| *Sinergasilus undulatus* | *Carassius auratus* | Cyprinidae | Tangxun Lake, China | DQ107527 | Song *et al.* (2008) |
| **Lernaeidae** |  |  |  |  |  |
| *Lamproglena chinensis* | *Channa argus* | Channidae | Dangjiangkou, China | DQ107545 | Song *et al.* (2008) |
| *Lamproglena clariae* | *Clarias gariepinus* | Clariidae | Vaal Dam, South Africa | OR048803 | Dos Santos *et al*. (2023) |
|  | *Clarias gariepinus* | Clariidae | Nyando River near Ahero town, Kenya | OR338195 | Rindoria *et al*. (2023) |
| *Lamproglena cleopatra* | *Labeo victorianus* | Cyprinidae | Nyando River near Ahero town, Kenya | OR338170 | Rindoria *et al*. (2023) |
| *Lamproglena hemprichii* | *Hydrocynus vittatus* | Alestidae | Lake Kariba, Zimbabwe | OP277527 | Dos Santos *et al*. (2023) |
| *Lamproglena hoi* | *Labeobarbus polylepis* | Cyprinidae | Komati River, South Africa | OR048808 | Dos Santos *et al*. (2023) |
| *Lamproglena monodi* | *Oreochromis niloticus* | Cichlidae | Kibos Fish Farm, Kenya | ON419422 | Rindoria *et al*. (2022) |
|  |  |  | Egypt | PQ872721 | Hanna *et al*. (2025) unpublished |
| *Lamproglena orientalis* | *Chanodichthys erythropterus* | Xenocyprididae | Tangxun Lake, China | DQ107541 | Song *et al.* (2008) |
|  | *Chanodichthys dabryi* | Xenocyprididae | Tangxun Lake, China | DQ107542 | Song *et al.* (2008) |
|  | *Chanodichthys mongolicus* | Xenocyprididae | E-zhou farm, China | DQ107543 | Song *et al.* (2008) |
|  | *Squaliobarbus curriculus* | Xenocyprididae | Dangjiangkou, China | DQ107544 | Song *et al.* (2008) |
| *Lernaea cruciata* | *Gambusia holbrooki* | Poeciliidaeo | Guangdong and Hunan, China | MH982211 | Hua *et al*. (2019) |
| *Lernaea ctenopharyngodontis* | *Siniperca chuatsi* | Sinipercidae | Zhangzhou farm, Fujian, China | MK742748 | Hua (2019) unpublished |
| *Lernaea cyprinacea* | *Carassius auratus* | Cyprinidae | Australia | KY346866 | McCredden (2016) |
|  | *Carassius auratus* | Cyprinidae | Al-Sharqia governorate, Egypt | KX258626 | Abu-Elala *et al*. (2018) |
|  | *Carassius gibelio* | Cyprinidae | Lake Chany, Russia | MW423693 | Kashinskaya *et al*. (2021) |
|  | *Cyprinus carpio* | Cyprinidae | Sleman and Bantul, Yogyakarta, Indonesia | OP902215 | Prastowo *et al.* (2023) |
|  | *Labeo rohita* | Cyprinidae | College of Fisheries, Mangaluru, India | OM835790 | Nirosha *et al*. (2022) unpublished |
|  | *Odontesthes bonariensis* | Atherinopsidae | Lagoon La Helvecia, Argentina | KX908211 | Soares *et al*. (2018) |
|  | *Plecoglossus altivelis* | Plecoglossidae | Shonai River, Japan | KP235364 | Yoshimine *et al*. (2015) unpublished |
|  | NA | Cyprinidae | Iran | KM281817 | Tabaripour & Youseffi (2014) unpublished |
| *Lernaea polymorpha* | *Hypophthalmichthys molitrix* | Xenocyprididae | Taibai Lake, Hubei, China | MK742757 | Hua (2019) unpublished |

**Supplementary Table S4:** List of all fish species including localities of their collection in the Mediterranean and list of collected parasitic copepods from respective hosts (N – number of fish hosts; NP – number of fish hosts positive for parasitic copepods; S – stage; L – localization on the host; A – abundance; IN – intensity of infection (min – max); P – prevalence).

| **Host species** | **Host family** | **N** | **NP** | **ID** | **Year** | **Locality** | **Parasitic copepod** | **A** | **IN** | **P** |
| --- | --- | --- | --- | --- | --- | --- | --- | --- | --- | --- |
| *Alburnoides economoui* Barbieri, Vukić, Šanda & Zogaris, 2017 | Cyprinidae | 11 | 0 | G7 | 2014 | Sperchios, Ypati | - | - | - | - |
| *Alburnoides fangfangae* Bogutskaya, Zupančič & Naseka, 2010 | Cyprinidae | 7 | 1 | A7 | 2015 | Osum, Vodice | *Paraergasilus longidigitus* | 1 | 1 | 14% |
| *Alburnoides ohridanus* (Karaman, 1928) | Cyprinidae | 10 | 1 | G27 | 2017 | Aoos | *Lamproglena pulchella* | 1 | 1 | 10% |
| *Alburnoides prespensis* (Karaman, 1924) | Cyprinidae | 2 | 0 | G20 | 2014 | Aoos, Kalithea | - | - | - | - |
| *Alburnus arborella* (Bonaparte, 1841) | Cyprinidae | 10 | 1 | I1 | 2015 | canale maestro de la Chiana, Chuisa dei Capannoi, Arno basis | *Neoergasilus japonicus* | 1 | 1 | 10% |
| *Alburnus neretvae* Buj, Šanda & Perea, 2010 | Cyprinidae | 7  10 | 1  9 | B3  B16 | 2015  2016 | Mušnica, Artovac  Zagorje, Jabuke | *Ergasilus briani*  *Ergasilus briani* | 2  52 | 2  1-14 | 14%  90% |
| *Alburnus scoranza* Heckel & Kner, 1857 | Cyprinidae | 5 | 3 | A11 | 2015 | Skadar lake, Shiroke | *Ergasilus briani*  *Paraergasilus longidigitus* | 4  2 | 1  1 | 80%  40% |
| *Barbus balcanicus* Kotlík, Tsigenopoulos, Ráb & Berrebi, 2002 | Cyprinidae | 5 | 0 | G4 | 2014 | Vardar, Axiopolis | - | - | - | - |
| *Barbus cyclolepis* Heckel, 1837 | Cyprinidae | 3 | 1 | G21 | 2014 | Macropotamos river, Filiouri basin | *Ergasilus lizae* | 20 | 20 | 33% |
| *Barbus peloponnesius* Valenciennes, 1842 | Cyprinidae | 8  8 | 0  2 | G11  G24 | 2014  2017 | Neda, Gianitsochori  Pamisos River | -  copepodid *Lamproglena* | -  3 | -  1-2 | -  25% |
| *Barbus prespensis* Karaman, 1924 | Cyprinidae | 5  8 | 0  0 | G17  G20 | 2014  2014 | Kokitos, Pagrati  Aoos, Kalithea | -  - | -  - | -  - | -  - |
| *Barbus sperchiensis* Stephanidis, 1950 | Cyprinidae | 4  4 | 1  4 | G7  G7 | 2014  2017 | Sperchios, Ypati  Sperchios, Ypati | *Ergasilus lizae*  *Ergasilus lizae* | 64  23 | 64  1-9 | 25%  100% |
| *Barbus strumicae* Karaman, 1955 | Cyprinidae | 5 | 0 | G1 | 2014 | Rihios river, Stavros | - | - | - | - |
| *Chondrostoma nasus* (Linnaeus, 1758) | Cyprinidae | 4 | 0 | G20 | 2014 | Aoos, Kalithea | - | - | - | - |
| *Chondrostoma soetta* Bonaparte, 1840 | Cyprinidae | 5 | 2 | I8 | 2018 | Carmagnola, Cave Germaire | *Neoergasilus japonicus* | 2 | 1 | 40% |
| *Chondrostoma vardarense* Karaman, 1928 | Cyprinidae | 2 | 0 | G2 | 2014 | Angitis river, Koninogia | - | - | - | - |
| *Iberochondrostoma almacai* (Coelho, Mesquita & Collares-Pereira, 2005) | Cyprinidae | 19 | 1 | P6 | 2016 | Torgal river, Mira basin | copepodid *Lernaea* | 1 | 1 | 5% |
| *Iberochondrostoma lusitanicum* (Collares-Pereira, 1980) | Cyprinidae | 15 | 1 | P4 | 2016 | Colares | copepodid *Lernaea* | 1 | 1 | 7% |
| *Leucos aula* (Bonaparte, 1841) | Cyprinidae | 10 | 8 | C1 | 2016 | Grabovač reservoir | *Ergasilus briani*  *Neoergasilus japonicus*  *Lernaea cyprinacea*  copepodid *Lernaea* | 16  3  1  1 | 1-5  1-2  1  1 | 50%  20%  10%  10% |
| *Leucos basak* Heckel, 1843 | Cyprinidae | 13 | 12 | B1 | 2015 | Krenica lake, Drinovci | *Ergasilus briani* | 205 | 5-27 | 92% |
| *Leucos panosi* (Bogutskaya & Iliadou, 2006) | Cyprinidae | 5 | 1 | G18 | 2014 | Rivio, Amvrakia | copepodid *Lamproglena* | 1 | 1 | 20% |
| *Leucos ylikiensis* (Economidis, 1991) | Cyprinidae | 9 | 8 | G23 | 2017 | Yliky Lake | *Ergasilus briani* | 84 | 1-25 | 89% |
| *Luciobarbus albanicus* (Steindachner, 1870) | Cyprinidae | 2  9 | 0  0 | G14  G19 | 2014  2014 | Pinios, Kalivakia  Trichonis lake, Panetolio | -  - | -  - | -  - | -  - |
| *Luciobarbus comizo* (Steindachner, 1864) | Cyprinidae | 5 | 5 | S7 | 2017 | Peraleda de Zancejo, Rio Zujar | copepodid *Lernaea* | 9 | 1-3 | 100% |
| *Luciobarbus graecus* (Steindachner, 1895) | Cyprinidae | 3 | 3 | G7 | 2017 | Sperchios, Ypati | *Ergasilus lizae* | 5 | 1-2 | 100% |
| *Luciobarbus guiraonis* (Steindachner, 1866) | Cyprinidae | 1  10  1 | 1  4  1 | S2  S3  S4 | 2016  2016  2016 | Magro river (1)  Magro river (2)  Turia river | copepodid *Lernaea*  *Lernaea cyprinacea*  copepodid *Lernaea*  copepodid *Lernaea* | 13  3  6  2 | 13  1  1-3  2 | 100%  33%  40%  100% |
| *Luciobarbus microcephalus* (Almaça, 1967) | Cyprinidae | 5 | 5 | S7 | 2017 | Peraleda de Zancejo, Zujar river | copepodid *Lernaea* | 6 | 1-2 | 100% |
| *Luciobarbus sclateri* (Günther, 1868) | Cyprinidae | 5 | 2 | P6 | 2016 | Torgal river, Mira basin | copepodid *Lernaea* | 4 | 2 | 40% |
| *Pachychilon macedonicum* (Steindachner, 1892) | Leuciscidae | 8 | 1 | G9 | 2014 | Pinios, Rongia – Valamandrio | - | - | - | - |
| *Pachychilon pictum* (Heckel & Kner, 1858) | Leuciscidae | 5 | 0 | G20 | 2014 | Aoos, Kalithea | - | - | - | - |
| *Parachondrostoma arrigonis* (Steindachner, 1866) | Leuciscidae | 3 | 2 | S3 | 2016 | Magro River (2) | *Lernaea cyprinacea*  copepodid *Lernaea* | 1  1 | 1  1 | 33%  33% |
| *Pelasgus laconicus* (Kottelat & Barbieri, 2004) | Leuciscidae | 10 | 0 | G13 | 2014 | Evrotas, Sparti | - | - | - | - |
| *Pelasgus stymphalicus* (Valenciennes, 1844) | Leuciscidae | 5  10 | 0  2 | G12  G24 | 2014  2017 | Pamisos, Vasiliko  Pamisos River | -  copepodid *Lamproglena* | -  2 | -  1 | -  20% |
| *Pelasgus thesproticus* (Stephanidis, 1939) | Leuciscidae | 1  5 | 1  0 | G16  G17 | 2014  2014 | Acheron, Gliki  Kokitos, Pagrati | copepodid *Lamproglena*  - | 1  - | 1  - | 100%  - |
| *Protochondrostoma genei* (Bonaparte, 1839) | Leuciscidae | 9 | 4 | I4 | 2015 | Torrente Cerfone, Le Ville | *Ergasilus italicus* n. sp.  *Lamproglena pulchella*  copepodid *Lamproglena* | 4  4  1 | 1  1-3  1 | 44%  22%  11% |
| *Pseudochondrostoma polylepis* (Steindachner, 1864) | Leuciscidae | 15  10 | 1  0 | P4  P2 | 2016  2016 | Colares  Alcoa, Fervenca | copepodid *Lernaea*  - | 1  - | 1  - | 7%  - |
| *Rutilus heckelii* (Nordmann, 1840) | Leuciscidae | 3 | 0 | G6 | 2014 | flood pools by Struma, Lithopos | - | - | - | - |
| *Rutilus* sp. Sperchios | Leuciscidae | 4 | 0 | G10 | 2014 | channel near Sperchios | - | - | - | - |
| *Sarmarutilus rubilio* (Bonaparte, 1837) | Leuciscidae | 10 | 1 | I3 | 2015 | Torrente Cerfone, Intoppo | *Lamproglena pulchella* | 1 | 1 | 10% |
| *Scardinius acarnanicus* Economidis, 1991 | Leuciscidae | 4 | 2 | G19 | 2014 | Trichonis lake, Panetolio | *Lamproglena pulchella*  copepodid *Lamproglena* | 5  2 | 2-3  1 | 50%  50% |
| *Scardinius dergle* Heckel & Kner, 1858 | Leuciscidae | 10 | 2 | C1 | 2016 | Grabovač rerervoir | *Neoergasilus japonicus*  copepodid *Lamproglena* | 1  1 | 1  1 | 10%  10% |
| *Scardinius plotizza* Heckel & Kner, 1858 | Leuciscidae | 7 | 6 | B15 | 2016 | Rečina river, near Jelim lake, Hutovo Blato | *Lamproglena pulchella* | 20 | 1-6 | 86% |
| *Squalius keadicus* (Stephanidis, 1971) | Leuciscidae | 5 | 0 | G13 | 2014 | Evrotas, Sparti | - | - | - | - |
| *Squalius orpheus* Kottelat & Economidis, 2006 | Leuciscidae | 4  1 | 0  1 | G1  G5 | 2014  2017 | Rihios river, Stavros  Angitis river, between Alistrati and Drama | -  *Lamproglena pulchella* | -  4 | -  4 | -  100% |
| *Squalius pamvoticus* (Stephanidis, 1939) | Leuciscidae | 6 | 1 | G16 | 2014 | Acheron, Gliki | *Lamproglena pulchella* | 1 | 1 | 17% |
| *Squalius peloponensis* (Valenciennes, 1844) | Leuciscidae | 5  5  5 | 0  2  2 | G12  G24  G25 | 2014  2017  2017 | Pamisos, Vasiliko  Pamisos River  Alfeios River | -  copepodid *Lamproglena*  copepodid *Lamproglena* | -  3  2 | -  1-2  1 | -  40%  40% |
| *Squalius prespensis* (Fowler, 1977) | Leuciscidae | 6  1 | 2  1 | G20  G27 | 2014  2017 | Aoos, Kalithea  Aoos | *Lamproglena pulchella*  copepodid *Lamproglena*  *Lamproglena pulchella* | 1  1  3 | 1  1  3 | 17%  17%  100% |
| *Squalius pyrenaicus* (Günther, 1868) | Leuciscidae | 5 | 5 | S7 | 2017 | Peraleda de Zancejo, Zujar river | *Lernaea cyprinacea*  copepodid *Lernaea* | 1  3 | 1  1 | 20%  60% |
| *Squalius squalus* (Bonaparte, 1837) | Leuciscidae | 11  10 | 1  3 | I3  C8 | 2015  2016 | Torrente Cerfone, Intoppo  Pazin, Pazinčica river | *Lamproglena pulchella*  *Neoergasilus japonicus* | 4  4 | 4  2 | 9%  20% |
| *Squalius* sp. Evinos | Leuciscidae | 2 | 1 | G19 | 2014 | Trichonis lake, Panetolio | copepodid *Lamproglena* | 1 | 1 | 50% |
| *Squalius tenellus* Heckel, 1843 | Leuciscidae | 3  11 | 1  1 | B5  B6 | 2015  2016 | Šujica, Duvansko Polje  Šujica, Šujičko Polje | *Ergasilus briani*  *Lernaea cyprinacea* | 1  2 | 1  2 | 33%  9% |
| *Squalius torgalensis* (Coelho, Bogutskaya, Rodrigues & Collares-Pereira, 1998) | Leuciscidae | 10 | 3 | P6 | 2016 | Torgal river, Mira basin | *Lernaea cyprinacea* | 3 | 1 | 30% |
| *Squalius valentinus* Doadrio & Carmona, 2006 | Leuciscidae | 5  5 | 2  5 | S2  S3 | 2016  2016 | Magro River (1)  Magro River (2) | copepodid *Lernaea*  *Lernaea cyprinacea*  copepodid *Lernaea* | 2  7  2 | 1  1-3  1 | 40%  100%  20% |
| *Squalius vardarensis* Karaman, 1928 | Leuciscidae | 7  1 | 4  1 | G3  G22 | 2014  2017 | Gallikos, Mandres, Gallikos basin  Pinios, Amygdalea | *Lamproglena pulchella*  copepodid *Lamproglena*  copepodid *Lamproglena* | 1  3  1 | 1  1  1 | 14%  43%  100% |
| *Telestes beoticus* (Stephanidis, 1939) | Leuciscidae | 8 | 0 | G8 | 2014 | stream in Livadia, Kifisos | - | - | - | - |
| *Telestes montenigrinus* (Vukovic, 1963) | Leuciscidae | 10 | 3 | A11 | 2015 | Skadar lake, Shiroke | *Paraergasilus longidigitus* | 3 | 1-2 | 30% |
| *Telestes pleurobipunctatus* (Stephanidis, 1939) | Leuciscidae | 5  4  6 | 0  0  3 | G15  G16  G17 | 2014  2014  2014 | Erimantos, Tripotamo  Acheron, Gliki  Kokitos, Pagrati | -  -  *Lamproglena pulchella* | -  -  3 | -  -  1-2 | -  -  50% |
| *Telestes* sp. | Leuciscidae | 11 | 3 | G26 | 2017 | Zagoritikos River, Baldouma | *Lamproglena pulchella*  copepodid *Lamproglena* | 2  2 | 1  1 | 18%  18% |
| *Tropidophoxinellus hellenicus* (Stephanidis, 1971) | Leuciscidae | 5  5 | 0  0 | G18  G19 | 2014  2014 | Rivio, Amvrakia  Trichonis lake, Panetolio | -  - | -  - | -  - | -  - |
| *Tropidophoxinellus spartiaticus* (Schmidt-Ries, 1943) | Leuciscidae | 5  5 | 0  0 | G11  G13 | 2014  2014 | Neda, Gianitsochori  Evrotas, Sparti | -  - | -  - | -  - | -  - |

**Supplementary Table S5:** List of all fish species including localities of their collection in the Middle East and list of collected parasitic copepods from respective hosts (N – number of fish hosts; NP – number of fish hosts positive for parasitic copepods; S – stage; L – localization on the host; A – abundance; IN – intensity of infection (min – max); P – prevalence).

| **Host species** | **Host family** | **N** | **NP** | **ID** | **Year** | **Locality** | **Parasitic copepod** | **A** | **IN** | **P** |
| --- | --- | --- | --- | --- | --- | --- | --- | --- | --- | --- |
| *Acanthobrama marmid* Heckel, 1843 | Cyprinidae | 1  10 | 0  1 | IRQ1  IRQ11 | 2019  2021 | Dukan Lake  Du Choman, Aw-e Shiler River | -  *Neoergasilus japonicus* | -  1 | -  1 | -  10% |
| *Acanthobrama microlepis* (De Filippi, 1863) | Cyprinidae | 1  5 | 0  1 | TUR16  TUR18 | 2022  2022 | Aralik, Aras, Kura basin  Ölçek, Ölçeksuyu, Kura basin | -  copepodid *Lamproglena* | -  1 | -  1 | -  20% |
| *Alburnoides diclensis* Turan, Bektaş, Kaya & Bayçelebi, 2016 | Cyprinidae | 7 | 0 | TUR35 | 2022 | inflow of Zab | - | - | - | - |
| *Alburnoides eichwaldii* (De Filippi, 1863) | Cyprinidae | 4  6 | 0  0 | TUR15  TUR17 | 2022  2022 | Ardahan, Kura basin  Yiğitkonağı closest village, Çakir, Kura basin | -  - | -  - | -  - | -  - |
| *Alburnoides emineae* Turan, Kaya, Ekmekçi & Doğan, 2014 | Cyprinidae | 10 | 0 | TUR29 | 2022 | Taşlıburç, Çağ-Çağ stream, Euphrates basin | - | - | - | - |
| *Alburnoides fasciatus* (Nordmann, 1840) | Cyprinidae | 6  5 | 0  3 | TUR31  TUR33 | 2022  2022 | inflow of Iyidere, Iyidere basin  ca 5 km north of Borçka, inflow of Çoruh, Çoruh basin | *-*  *Ergasilus briani* | -  14 | -  2-9 | -  60% |
| *Alburnoides kosswigi* Turan, Kaya, Bayçelebi, Bektaş & Ekmekçi, 2017 | Cyprinidae | 10  5 | 0  0 | TUR5  TUR7 | 2021  2021 | Porsuk Tibet  Seyitgazi | -  - | -  - | -  - | -  - |
| *Alburnoides manyasensis* Turan, Ekmekçi, Kaya & Güçlü, 2013 | Cyprinidae | 13 | 0 | TUR36 | 2023 | Nilufer river, upstream the reservoir Doganci | - | - | - | - |
| *Alburnoides petrubanarescui* Bogutskaya & Coad, 2009 | Cyprinidae | 7 | 0 | TUR27 | 2022 | west of Esendere, Urmia Lake | - | - | - | - |
| *Alburnoides velioglui* Turan, Kaya, Ekmekçi & Doğan, 2014 | Cyprinidae | 10 | 0 | TUR20 | 2022 | Aşağıkent, Cuma stream, Euphrates basin | - | - | - | - |
| *Alburnus alburnus* (Linnaeus, 1758) | Cyprinidae | 5 | 0 | TUR13 | 2021 | Büyükdoğanca, Söğütlü creek, Evros | - | - | - | - |
| *Alburnus baliki* Bogutskaya, Küçük & Ünlü, 2000 | Cyprinidae | 15 | 0 | TUR50 | 2023 | Çenger stream, Çavuşköy, Kapruz drainage | - | - | - | - |
| *Alburnus attalus* Özuluğ & Freyhof, 2007 | Cyprinidae | 2 | 0 | TUR43 | 2023 | between Ortabag and Diskaya | - | - | - | - |
| *Alburnus caeruleus* Heckel, 1843 | Cyprinidae | 10 | 0 | TUR29 | 2022 | Taşlıburç, Çağ-Çağ stream, Euphrates basin | - | - | - | - |
| *Alburnus carianorum* Freyhof, Kaya, Bayçelebi, Geiger & Turan, 2019 | Cyprinidae | 3 | 1 | TUR4 | 2020 | Çine River, near Sitmalik | copepodid *Lernaea* | 1 | 1 | 33% |
| *Alburnus demiri* Özuluğ & Freyhof, 2008 | Cyprinidae | 7 | 0 | TUR39 | 2023 | Sasal stream, Kuner | - | - | - | - |
| *Alburnus derjugini* Berg, 1923 | Cyprinidae | 5 | 0 | TUR32 | 2022 | west of Ardanuç, Cehennem/Şuat/Köprüler stream, Çoruh basin | - | - | - | - |
| *Alburnus escherichii* Steindachner, 1897 | Cyprinidae | 10  10 | 0  0 | TUR5  TUR8 | 2021  2021 | Porsuk Tibet  Kütahya | -  - | -  - | -  - | -  - |
| *Alburnus filippii* Kessler, 1877 | Cyprinidae | 6 | 0 | TUR15 | 2022 | Ardahan, Kura basin | - | - | - | - |
| *Alburnus hohenackeri* Kessler, 1877 | Cyprinidae | 15 | 0 | TUR16 | 2022 | Aralik, Aras, Kura basin | - | - | - | - |
| *Alburnus sellal* Heckel, 1843 | Cyprinidae | 2  1  12  7  5 | 1  1  0  1  0 | IRQ1  IRQ2  IRQ7  IRQ9  IRQ10 | 2019  2019  2021  2021  2021 | Dukan Lake  Great Zab River (1)  wadi Kalat Shirah, tributary of Tabin River  Zahrzi, Tabin River  Grdi Go, Zalm Stream | *Neoergasilus japonicus*  *Neoergasilus japonicus*  -  *Neoergasilus japonicus*  - | 2  1  -  1  - | 2  1  -  1  - | 50%  100%  -  14%  - |
| *Alburnus* sp*. (*from *Alburnus alburnus* complex*)* | Cyprinidae | 10 | 1 | TUR37 | 2023 | Simav river, Karacabey | *Ergasilus briani* | 1 | 1 | 10% |
| *Alburnus* sp. | Cyprinidae | 20 | 0 | IRQ10 | 2021 | Grdi Go, Zalm Stream | - | - | - | - |
| *Alburnus tarichi* (Güldenstädt, 1814) | Cyprinidae | 5 | 0 | TUR24 | 2022 | Haydarbey (Erciş), Deliçay stream, Van Lake | - | - | - | - |
| *Alburnus timarensis* Kuru, 1980 | Cyprinidae | 5 | 0 | TUR23 | 2022 | between Otluca and Kasımoğlu, Karasu stream, Van Lake | - | - | - | - |
| *Arabibarbus grypus* (Heckel, 1843) | Cyprinidae | 1 | 0 | IRQ | 2021 | fish market | - | - | - | - |
| *Barbus anatolicus* Turan, Kaya, Geiger & Freyhof, 2018 | Cyprinidae | 5 | 0 | TUR30 | 2022 | Kelkit, Kelkit stream, Yeşilirmak basin | - | - | - | - |
| *Barbus cyclolepis* Heckel, 1837 | Cyprinidae | 6 | 0 | TUR14 | 2021 | South of Yülüce, Kocadere | - | - | - | - |
| *Barbus cyri* De Filippi, 1865 | Cyprinidae | 3  4  1  4 | 0  2  0  0 | TUR15  TUR17  TUR18  TUR27 | 2022  2022  2022  2022 | Ardahan, Kura basin  Yiğitkonağı closest village, Çakir, Kura basin  Ölçek, Ölçeksuyu, Kura basin  west of Esendere, Urmia Lake | -  *Lamproglena pulchella*  -  - | -  5  -  - | -  2-3  -  - | -  50%  -  - |
| *Barbus escherichii* (Steindachner, 1897) | Cyprinidae | 5  5  10  9  1 | 1  5  0  0  0 | TUR6  TUR8  TUR10  TUR32  TUR34 | 2021  2021  2021  2022  2022 | Çifteler  Kütahya  east of Doğançay, inflow of the Sakarya  west of Ardanuç, Cehennem/Şuat/Köprüler stream, Çoruh basin  Çoruh basin | copepodid *Lamproglena*  *Ergasilus briani*  -  -  - | 1  165  -  -  - | 1  3-72  -  -  - | 20%  100%  -  -  - |
| *Barbus ida* Güçlü, Kalayci, Ozulug & Turan, 2021 | Cyprinidae | 5 | 0 | TUR40 | 2023 | inflow of Gonen river, Kalkim | - | - | - | - |
| *Barbus lacerta* Heckel, 1843 | Cyprinidae | 10  3 | 3  0 | IRQ8  TUR24 | 2021  2022 | Kani Shok, tributary of Tabin River  Haydarbey (Erciş), Deliçay stream, Van Lake | *Ergasilus rostralis*  - | 4  - | 4  - | 30%  - |
| *Barbus niluferensis* Turan, Kottelat & Ekmekçi, 2009 | Cyprinidae | 5 | 0 | TUR36 | 2023 | Nilufer river, upstream the reservoir Doganci | - | - | - | - |
| *Barbus oligolepis* Battalgil, 1941 | Cyprinidae | 12  3 | 0  0 | TUR12  TUR38 | 2021  2023 | East of Barakfaith, Burutma kanali, Nilufer  Koca river, Cavus | -  - | -  - | -  - | -  - |
| *Barbus* sp. Tahtali | Cyprinidae | 5 | 1 | TUR39 | 2023 | Sasal stream, Kuner | *Ergasilus barbi* | 5 | 5 | 20% |
| *Barbus tauricus* Kessler, 1877 | Cyprinidae | 10 | 0 | TUR31 | 2022 | inflow of Iyidere, Iyidere basin | - | - | - | - |
| *Barbus xanthos* Güçlü, Kalayci, Küçük & Turan, 2020 | Cyprinidae | 7 | 0 | TUR1 | 2020 | Çine River, near Çiftlikköy | - | - | - | - |
| *Capoeta antalyensis* (Battalgil, 1943) | Cyprinidae | 2  1 | 0  0 | TUR47  TUR51 | 2023  2023 | Kovada river, Asagi Gokdere, Aksu drainage  Ilica stream, Ilica | -  - | -  - | -  - | -  - |
| *Capoeta aydinensis* Turan, Küçük, Kaya, Güçlü & Bektaş, 2017 | Cyprinidae | 10 | 4 | TUR1 | 2020 | Çine River, near Çiftlikköy | *Ergasilus barbi* | 8 | 1-3 | 40% |
| *Capoeta banarescui* Turan, Kottelat, Ekmekçi & Imamoglu, 2006 | Cyprinidae | 5 | 0 | TUR30 | 2022 | Kelkit, Kelkit stream, Yeşilirmak basin | - | - | - | - |
| *Capoeta caelestis* Schöter, Özuluğ & Freyhof, 2009 | Cyprinidae | 5  5 | 0  0 | TUR50  TUR51 | 2023  2023 | Çenger stream, Çavuşköy, Kapruz drainage  Ilica stream, Ilica | -  - | -  - | -  - | -  - |
| *Capoeta capoeta* (Güldenstädt, 1773) | Cyprinidae | 7  4  8 | 1  0  0 | TUR15  TUR16  TUR32 | 2022  2022  2022 | Ardahan, Kura basin  Aralik, Aras, Kura basin  west of Ardanuç, Cehennem/Şuat/Köprüler stream, Çoruh basin | *Lamproglena pulchella*  -  - | 1  -  - | 1  -  - | 14%  -  - |
| *Capoeta damascina* (Valenciennes, 1842) | Cyprinidae | 4  3 | 1  0 | TUR21  TUR23 | 2022  2022 | Kayacik, Cuma stream, Euphrates basin  between Otluca and Kasımoğlu, Karasu stream, Van Lake | *Lernaea cyprinacea*  - | 1  - | 1  - | 25%  - |
| *Capoeta sieboldii* (Steindachner, 1864) | Cyprinidae | 7  1 | 1  1 | TUR6  TUR32 | 2021  2022 | Çifteler  west of Ardanuç, Cehennem/Şuat/Köprüler stream, Çoruh basin | *Lamproglena pulchella*  *Lamproglena pulchella* | 1  2 | 1  2 | 14%  100% |
| *Capoeta tinca* (Heckel, 1843) | Cyprinidae | 1  5  8 | 0  1  0 | TUR5  TUR6  TUR7 | 2021  2021  2021 | Porsuk Tibet  Çifteler  Seyitgazi | -  *Lamproglena pulchella*  - | -  4  - | -  4  - | -  20%  - |
| *Capoeta umbla* (Heckel, 1843) | Cyprinidae | 10 | 1 | IRQ7 | 2021 | wadi Kalat Shirah, tributary of Tabin River | *Ergasilus rostralis* | 1 | 1 | 10% |
| *Carassius auratus* (Linnaeus, 1758) | Cyprinidae | 4 | 0 | IRQ3 | 2019 | Darbandikhan Lake | - | - | - | - |
| *Carasobarbus luteus* (Heckel, 1843) | Cyprinidae | 3  8  2  3 | 1  5  2  2 | IRQ3  IRQ9  IRQ10  IRQ11 | 2019  2021  2021  2021 | Darbandikhan Lake  Zahrzi, Tabin River  Grdi Go, Zalm Stream  Du Choman, Aw-e Shiler River | *Neoergasilus japonicus*  *Pseudolamproglena zahrziensis* n. sp.  copepodid *Pseudolamproglena*  *Pseudolamproglena zahrziensis* n. sp.  copepodid *Pseudolamproglena*  *Pseudolamproglena zahrziensis* n. sp. | 1  6  5  7  13  3 | 1  1-4  1-2  7  1-12  1-2 | 33%  63%  50%  50%  100%  67% |
| *Chondrostoma colchicum* Derjugin, 1899 | Cyprinidae | 5  5  5  3 | 2  1  0  0 | TUR6  TUR7  TUR8  TUR34 | 2021  2021  2021  2022 | Çifteler  Seyitgazi  Kütahya  Çoruh basin | *Ergasilus barbi*  copepodid *Lamproglena*  *Ergasilus barbi*  -  - | 1  1  3  -  - | 1  1  3  -  - | 20%  20%  20%  -  - |
| *Chondrostoma cyri* Kessler, 1877 | Cyprinidae | 5 | 0 | TUR16 | 2022 | Aralik, Aras, Kura basin | - | - | - | - |
| *Chondrostoma holmwoodii* (Boulenger, 1896) | Cyprinidae | 10 | 0 | TUR43 | 2023 | between Ortabag and Diskaya | - | - | - | - |
| *Chondrostoma kinzelbachi* Krupp, 1985 | Cyprinidae | 3 | 0 | IRQ2 | 2019 | Great Zab River (1) | - | - | - | - |
| *Chondrostoma meandrense* Elvira, 1987 | Cyprinidae | 9 | 1 | TUR | 2023 | Büyük Menderes, around 1 km east of Işıklı lake | *Lernaea cyprinacea* | 1 | 1 | 11% |
| *Chondrostoma regium* (Heckel, 1843) | Cyprinidae | 6 | 6 | IRQ11 | 2021 | Du Choman, Aw-e Shiler River | *Neoergasilus japonicus* | 18 | 1-5 | 100% |
| *Chondrostoma smyrnae* Küçük, Çiftçi, Güçlü & Turan, 2021 | Cyprinidae | 3 | 0 | TUR39 | 2023 | Sasal stream, Kuner | - | - | - | - |
| *Chondrostoma turnai* Güçlü, Küçük, Turan, Çiftçi & Mutlu, 2018 | Cyprinidae | 5 | 3 | TUR4 | 2020 | Çine River, near Sitmalik | *Neoergasilus japonicus* | 22 | 1-18 | 60% |
| *Cobitis simplicispina* Hankó, 1925 | Cobitidae | 5 | 0 | TUR7 | 2021 | Seyitgazi | - | - | - | - |
| *Cyprinion kais* Heckel, 1843 | Cyprinidae | 8 | 1 | TUR26 | 2022 | Sinanköy, Akçayır stream (inflow of Batman river), Tigris basin | *Lernaea cyprinacea* | 2 | 2 | 13% |
| *Cyprinion macrostomus* Heckel, 1843 | Cyprinidae | 5  9  1  10 | 2  3  0  0 | IRQ1  IRQ3  IRQ7  IRQ8 | 2019  2019  2021  2021 | Dukan Lake  Darbandikhan Lake  wadi Kalat Shirah, tributary of Tabin River  Kani Shok, tributary of Tabin River | *Neoergasilus japonicus*  *Neoergasilus japonicus*  -  - | 2  3  -  - | 1  3  -  - | 40%  33%  -  - |
| *Garra rufa* (Heckel, 1843) | Cyprinidae | 10  3 | 0  0 | IRQ6  TUR26 | 2021  2022 | by the road Suleymania-Dukan, Little Zab  Sinanköy, Akçayır stream (inflow of Batman river), Tigris basin | -  - | -  - | -  - | -  - |
| *Garra rezai* Mousavi-Sabet, Eagderi, Saemi-Komsari, Kaya & Freyhof, 2022 | Cyprinidae | 5 | 3 | TUR25 | 2022 | near Meydan village, inflow of Garzan river, Tigris basin | *Lamproglena pulchella* | 3 | 1 | 60% |
| *Garra turcica* Karaman, 1971 | Cyprinidae | 10 | 0 | TUR51 | 2023 | Ilica stream, Ilica | - | - | - | - |
| *Garra variabilis* (Heckel, 1843) | Cyprinidae | 15 | 0 | TUR28 | 2022 | Darköprü, Çelebyian stream, Tigris basin | - | - | - | - |
| *Gobio maeandricus*  Naseka, Erk'akan & Küçük, 2006 | Gobionidae | 2 | 0 | TUR | 2023 | Büyük Menderes, around 1 km east of Işıklı lake | - | - | - | - |
| *Gobio sakaryaensis* Turan, Ekmekçi, Luskova & Mendel, 2012 | Gobionidae | 3  10 | 0  4 | TUR5  TUR7 | 2021  2021 | Porsuk Tibet  Seyitgazi | -  *Lamproglena pulchella*  copepodid *Lamproglena* | -  22  4 | -  1-12  1-3 | -  40%  20% |
| *Gobio artvinicus* Turan, Japoshvili, Aksu & Bektaş, 2016 | Gobionidae | 5 | 1 | TUR32 | 2022 | west of Ardanuç, Cehennem/Şuat/Köprüler stream, Çoruh basin | *Ergasilus briani* | 41 | 41 | 20% |
| *Leucalburnus satunini* (Berg, 1910) | Leuciscidae | 3  3 | 0  0 | TUR15  TUR17 | 2022  2022 | Ardahan, Kura basin  Yiğitkonağı closest village, Çakir, Kura basin | -  - | -  - | -  - | -  - |
| *Luciobarbus barbulus* (Heckel, 1847) | Cyprinidae | 7 | 2 | IRQ11 | 2021 | Du Choman, Aw-e Shiler River | *Neoergasilus japonicus* | 2 | 1 | 29% |
| *Luciobarbus capito* (Güldenstädt, 1773) | Cyprinidae | 3 | 0 | TUR19 | 2022 | Gaziler stream near the mouth to Aras, Gaziler, Kura Basin | - | - | - | - |
| *Luciobarbus graecus* (Steindachner, 1895) | Cyprinidae | 10  10 | 0  0 | TUR1  TUR43 | 2020  2023 | Çine River, near Çiftlikköy  between Ortabag and Diskaya | -  - | -  - | -  - | -  - |
| *Luciobarbus mursa* (Güldenstädt, 1773) | Cyprinidae | 1 | 0 | TUR19 | 2022 | Gaziler stream near the mouth to Aras, Gaziler, Kura Basin | - | - | - | - |
| *Luciobarbus schejch* (Steindachner, 1897) | Cyprinidae | 1  2 | 0  0 | IRQ4  IRQ5 | 2019  2021 | Great Zab River (2)  Dukan, Litle Zab | - | - | - | - |
| *Oxynoemacheilus angorae* (Steindachner, 1897) | Nemacheilidae | 3 | 0 | TUR5 | 2021 | Porsuk Tibet | - | - | - | - |
| *Oxynoemacheilus* sp. | Nemacheilidae | 6  3 | 0  0 | TUR1  IRQ5 | 2020  2021 | Çine River, near Çiftlikköy  Dukan, Litle Zab | -  - | -  - | -  - | -  - |
| *Paracapoeta trutta* (Heckel, 1843) | Cyprinidae | 3  10 | 0  0 | IRQ2  IRQ8 | 2019  2021 | Great Zab River (1)  East of Barakfaith, Burutma kanali, Nilufer | -  - | -  - | -  - | -  - |
| *Petroleuciscus borysthenicus* (Kessler, 1859) | Leuciscidae | 5 | 0 | TUR12 | 2021 | East of Barakfaith, Burutma kanali, Nilufer | - | - | - | - |
| *Petroleuciscus ninae* Turan, Kalayci, Kaya, Bektaş & Küçük, 2018 | Leuciscidae | 15  15 | 0  0 | TUR1  TUR2 | 2020  2020 | Çine River, near Çiftlikköy  Kamişdere stream, near Yatagan | -  - | -  - | -  - | -  - |
| *Petroleuciscus smyrnaeus* (Boulenger, 1896) | Leuciscidae | 10 | 0 | TUR42 | 2023 | Gediz river, Yesilkoy | - | - | - | - |
| *Phoxinus* sp. | Leuciscidae | 10 | 0 | TUR9 | 2021 | south of Mahmudyie, inflow of the Sapanca Lake | - | - | - | - |
| *Phoxinus* sp. Gönen | Leuciscidae | 10 | 0 | TUR40 | 2023 | inflow of Gonen river, Kalkim | - | - | - | - |
| *Planiliza abu* (Heckel, 1843) | Mugilidae | 1  2 | 0  0 | IRQ2  IRQ4 | 2019  2019 | Great Zab River (1)  Great Zab River (2) | -  - | -  - | -  - | -  - |
| *Pseudophoxinus antalyae* Bogutskaya, 1992 | Leuciscidae | 10 | 0 | TUR52 | 2023 | Kirgkoz wetland | - | - | - | - |
| *Pseudophoxinus burduricus* Küçük, Gülle, Güçlü, Çiftçi & Erdoğan, 2013 | Leuciscidae | 10 | 0 | TUR45 | 2023 | Karamanli | - | - | - | - |
| *Pseudophoxinus evliyae* Freyhof & Özuluğ, 2010 | Leuciscidae | 10 | 0 | TUR46 | 2023 | Kirkpinar | - | - | - | - |
| *Pseudophoxinus fahrettini*  Freyhof & Özuluğ, 2010 | Leuciscidae | 10 | 0 | TUR48 | 2023 | Stream in Bagilli, Kopru river drainage | - | - | - | - |
| *Pseudophoxinus firati* Bogutskaya, Küçük & Atalay, 2006 | Leuciscidae | 5 | 0 | TUR22 | 2022 | Özkavak, marsh with creek in Karasu River, Euphrates basin | - | - | - | - |
| *Rutilus rutilus* (Linnaeus, 1758) | Leuciscidae | 3 | 0 | TUR13 | 2021 | Büyükdoğanca, Söğütlü creek, Evros | - | - | - | - |
| *Squalius agdamicus* Kamensky, 1901 | Leuciscidae | 6 | 0 | TUR15 | 2022 | Ardahan, Kura basin | - | - | - | - |
| *Squalius aristotelis* Özuluğ & Freyhof, 2011 | Leuciscidae | 5 | 0 | TUR41 | 2023 | Tuzla river, Ayvacik | - | - | - | - |
| *Squalius berak* Heckel, 1843 | Leuciscidae | 9  1 | 0  0 | IRQ8  IRQ11 | 2021  2021 | Kani Shok, tributary of Tabin River  Du Choman, Aw-e Shiler River | -  - | -  - | -  - | -  - |
| *Squalius fellowesii* (Günther, 1868) | Leuciscidae | 11  10 | 0  10 | TUR1  TUR2 | 2020  2020 | Çine River, near Çiftlikköy  Kocaalam Deresi | -  *Lamproglena pulchella*  copepodid *Lamproglena* | -  58  8 | -  1-13  1-3 | -  100%  50% |
| *Squalius carinus* Özuluğ & Freyhof, 2011 | Leuciscidae | 1 | 0 | TUR | 2023 | Büyük Menderes, around 1 km east of Işıklı lake | - | - | - | - |
| *Squalius cii* (Richardson, 1857) | Leuciscidae | 5  8  3 | 0  0  0 | TUR11  TUR12  TUR38 | 2021  2021  2023 | Sapanca, inflow to the Sapanca Lake  East of Barakfaith, Burutma kanali, Nilufer  Koca river, Cavus | -  -  - | -  -  - | -  -  - | -  -  - |
| *Squalius ghigii* (Gianferrari, 1927) | Leuciscidae | 10 | 4 | TUR3 | 2020 | Kocaalam Deresi | copepodid *Lamproglena* | 11 | 1-4 | 40% |
| *Squalius kosswigi* (Karaman, 1972) | Leuciscidae | 5 | 0 | TUR39 | 2023 | Sasal stream, Kuner | - | - | - | - |
| *Squalius lepidus* Heckel, 1843 | Leuciscidae | 9 | 4 | IRQ11 | 2021 | Du Choman, Aw-e Shiler River | *Neoergasilus japonicus* | 7 | 1-3 | 44% |
| *Squalius orientalis* Heckel, 1847 | Leuciscidae | 5  2 | 0  0 | TUR30  TUR33 | 2022  2022 | Kelkit, Kelkit stream, Yeşilirmak basin  ca 5 km north of Borçka, inflow of Çoruh, Çoruh basin | -  - | -  - | -  - | -  - |
| *Squalius orpheus* Kottelat & Economidis, 2006 | Leuciscidae | 3  1 | 0  0 | TUR13  TUR14 | 2021  2021 | Büyükdoğanca, Söğütlü creek, Evros  South of Yülüce, Kocadere | -  - | -  - | -  - | -  - |
| *Squalius pursakensis* (Hankó, 1925) | Leuciscidae | 7  7  1 | 0  0  0 | TUR5  TUR7  TUR8 | 2021  2021  2021 | Porsuk Tibet  Seyitgazi  Kütahya | -  -  - | -  -  - | -  -  - | -  -  - |
| *Squalius semae* Turan, Kottelat & Bayçelebi, 2017 | Leuciscidae | 3 | 0 | TUR21 | 2022 | Kayacik, Cuma stream, Euphrates basin | - | - | - | - |
| *Squalius* sp. | Leuciscidae | 3 | 0 | TUR49 | 2023 | Kovada river, Aksu drainage | - | - | - | - |
| *Squalius turcicus* De Filippi, 1865 | Leuciscidae | 5 | 0 | TUR16 | 2022 | Aralik, Aras, Kura basin | - | - | - | - |
| *Turcichondrostoma fahirae* (Ladiges, 1960) | Leuciscidae | 10 | 0 | TUR44 | 2023 | Karamusa, upper Dalaman | - | - | - | - |
| *Vimba mirabilis* (Ladiges, 1960) | Leuciscidae | 10 | 3 | TUR4 | 2020 | Çine River, near Sitmalik | *Neoergasilus japonicus* | 5 | 1-3 | 30% |
| *Vimba vimba* (Linnaeus, 1758) | Leuciscidae | 2 | 0 | TUR6 | 2021 | Çifteler | - | - | - | - |

REFERENCES

**Abbas, A. A. K.** (2007). Histopathological studies of some parasites of the Asian catfish, *Silurus triostegus* (Heckel, 1843) and potassium permanganate on the black molly, *Poecilia sphenops* (Valenciennes, 1846) (Ph.D. thesis). College of Education, University of Basrah, Iraq. (In Arabic).

**Abbas, F., Ashraf, M., Hafeez-ur-Rehman, M., Iqbal, K. J., Abbas, S., & Javid, A.** (2014). *Lernaea* susceptibility, infestation, and its treatment in indigenous major and exotic Chinese carps under a polyculture system. *Pakistan Journal of Zoology, 46*(5), 1215–1222.

**Abd, A. A.-A., & Abdul Wahab, H. M.** (2011). Investigation of some diseases of carp at Al-Shamiya city/Iraq. *Kufa Journal of Veterinary Medicine Sciences*, *2*(2), 51-59.

**Abd El-Galil, M. A., Essa, M. A. A., & Korni, F. M. M.** (2012). Studies on lernaeosis and the efficacy of Dipterex as treatment in the hatchery reared fingerlings of cyprinids. *Journal of American Science, 8*(8).

**Abdel-Gaber, R., El Deeb, N., Maher, S., & Kamel, R.** (2017). Diversity and host distribution of the external gill parasite *Lamproglena monodi* (Copepoda: Lernaeidae) among Tilapia species in Egypt: Light and scanning electron microscopic studies. *Egyptian Journal of Experimental Biology (Zoology), 13*(1).

**Abdel-Hady, O. K., Bayoumy, E. M., & Osman, H. A. M.** (2008). New copepodal ergasilid parasitic on *Tilapia zilli* from Lake Temsah with special reference to its pathological effect. *Global Veterinaria*, *2*, 123-129.

**Abdel-Radi, S., Rashad, M. M., Ali, G. E., Eissa, A. E., Abdelsalam, M., & Abou-Okada, M.** (2022). Molecular characterization and phylogenetic analysis of parasitic copepoda; *Ergasilus sieboldi* isolated from cultured gilthead sea bream (*Sparus aurata*) in Egypt, associated with analysis of oxidative stress biomarkers. *Journal of Parasitic Diseases, 46*(4), 1080–1089.

**Abdi, K., Jalali, B., Moobedi, I., & Naem, S.** (1995). Identification of crustacean parasites in Mahabad Reservoir. *Pazhohesh and Sazandegi, 36*, 128–132. (In Persian).

**Abdullah, S. M. A.** (2002). *Ecology, taxonomy and biology of some parasites of fishes from Lesser Zab and Greater Zab rivers in north of Iraq* (Ph.D. thesis). College of Education (Ibn Al-Haitham), University of Baghdad, Iraq. (In Arabic).

**Abdullah, S. M. A.** (2004). Comparison between the parasitic infections of fishes caught in two of each of small natural habitats and fish farms in Erbil City. *Zanco*, *16*(4), 43–50. (In Arabic).

**Abdullah, S. M. A.** (2005). Parasitic fauna of some freshwater fishes from Darbandikhan Lake, north of Iraq. *Journal of Dohuk University, 8*(1), 29–35.

**Abdullah, S. M. A., & Mhaisen, F. T**. (2003). The ecology of *Ergasilus barbi* (Copepoda: Crustacea) parasitizing gills of *Barbus luteus* from Greater Zab River in north of Iraq. *Iraqi Journal of Agriculture* (Special Issue), *8*(1), 141–147.

**Abdullah, S. M. A., & Ismail, T. F.** (2004). Observations on *Lernaea cyprinacea* L. parasite of freshwater fishes in Kurdistan of Iraq. *Zanco*, *16*(2), 25–34. (In Arabic).

**Abdullah, S. M. A., & Rasheed, A. A. M.** (2004). Parasitic fauna of some freshwater fishes from Dokan Lake, north of Iraq. I: Ectoparasites. *Ibn Al-Haitham Journal of Pure and Applied Science*, *17*(1), 34–46.

**Abdullah, S. M. A., & Mhaisen, F. T.** (2006). Parasitic infections with Protozoa and Crustacea on fishes of Lesser Zab and Greater Zab rivers, north of Iraq. In *Proceedings of the 4th Scientific Conference, College of Veterinary Medicine, University of Mosul, Mosul* (Vol. 1, pp. 51–58.)

**Abdullah, S. M. A., & Shwani, A. A. A.** (2010). Ectoparasites of the Asian catfish *Silurus triostegus* (Heckel, 1843) from Greater Zab River—Kurdistan Region—Iraq. *Journal of Duhok University, 13*(1), 164-171.

**Abdullah, S. M. A., & Mhaisen, F. T.** (2011). Some ecological aspects of the crustacean *Ergasilus barbi* parasitizing gills of *Liza abu* from Greater Zab and Lesser Zab rivers in north of Iraq. In *Proceedings of the Second Scientific Conference of Biological Sciences, College of Science, University of Mosul* (pp. 145–152), Mosul, Iraq.

**Abdullah, Y. S., & Abdullah, S. M. A.** (2015a). Observations on fishes and their parasites of Darbandikhan Lake, Kurdistan Region in north Iraq. *American Journal of Biological and Life Sciences, 3*(5), 176-180.

**Abdullah, Y. S., & Abdullah, S. M. A.** (2015b). The parasitic infections of some freshwater fishes from Darbandikhan Lake, Kurdistan Region, Iraq. *Journal of Garmian University, Special Issue 2*, 874-884.

**Abdullah, Y. S., & Abdullah, S. M. A.** (2015c). Some observations on fishes and their parasites of Darbandikhan Lake, Kurdistan Region in north Iraq. *European Scientific Journal, Special Edition*, 409-417.

**Abdulrahman, N. M., Hussein, A. J., Sulaiman, S. S., & Rasul, B. A.** (2020). Survey of fish diseases in Ranya (Raparin administration)/ Sulaimaniya governorate: Case study. *Basrah Journal of Veterinary Research*, *19*(3), 32-47.

**Abed, J. M.** (2005). *Biological and ecological survey of some enemies of fishes in three fish farms in Iraq* (Ph.D. thesis). College of Agriculture, University of Basrah, Iraq. 111 pp. (In Arabic).

**Abou-Okada, M., Rashad, M. M., Ali, G. E., Abdel-Radi, S., & Hassan, A.** (2023). Oxidative stress, gene expression and histopathology of cultured gilthead sea bream (*Sparus aurata*) naturally co-infected with *Ergasilus sieboldi* and *Vibrio alginolyticus*. *BMC Veterinary Research, 19*(1), 277.

**Abu-Elala, N. M., Attia, M. M., & Abd-Elsalam, R. M.** (2018). Chitosan-silver nanocomposites in goldfish aquaria: A new perspective in *Lernaea cyprinacea* control. *International Journal of Biological Macromolecules, 111*, 614-622.

**Adday, T. K., Balasem, A. N., Mhaisen, F. T., & Al-Khateeb, G. H.** (1999). A second survey of fish parasites from Tigris River at Al-Zaafaraniya, south of Baghdad. *Ibn Al-Haitham Journal for Pure and Applied Science, 12*(1), 22–31.

**Adday, T. K., Balasem, A. N., & Khamees, N. R.** (2006a). First occurrence of the crustacean *Ergasilus ogawai* from gills of four species of fishes in Iraq. *Ibn Al-Haitham Journal for Pure and Applied Science, 19*(2), 18–31. (In Arabic).

**Adday, T. K., Khamees, N. R., & Balasem, A. N.** (2006b). Biology of the copepod *Ergasilus ogawai* Kabata, 1992 parasitic on gills of *Silurus triostegus* at Garmat Ali River, north of Basrah City. *Ibn Al-Haitham Journal for Pure and Applied Science, 19*(1), 18–27. (In Arabic).

**Adday, T. K., & Ali, A. H.** (2011). *Ergasilus boleophthalmi* sp. n. (Copepoda: Ergasilidae) parasitic on gobiid fishes from Shatt Al-Basrah Canal, south of Iraq. *Wiadomości Parazytologiczne*, *57*(3), 137–142.

**Adday, T. K.** (2013). *Parasitic crustaceans of some marine fishes of Basrah Province, Iraq* (Ph.D. thesis). University of Basrah, College of Agriculture, Iraq. 302 pp.

**Aguilar, A., Alvarez, M. F., Leiro, J. M., & Sanmartin, M. L.** (2005). Parasite populations of the European eel (*Anguilla anguilla* L.) in the Rivers Ulla and Tea (Galicia, northwest Spain). *Aquaculture*, *249*(1-4), 85-94.

**Ahmed, S. M., & Ali, A. H.** (2013). Serum proteins and leucocytes differential count in the common carp (*Cyprinus carpio* L.) infested with ectoparasites. *Mesopotamian Journal of Marine Sciences*, *28*(2), 151-162.

**Ahnelt, H., Konecny, R., Gabriel, A., Bauer, A., Pompei, L., Lorenzoni, M., & Sattmann, H.** (2018). First report of the parasitic copepod *Lernaea cyprinacea* (Copepoda: Lernaeidae) on gobioid fishes (Teleostei: Gobonellidae) in southern Europe. *Knowledge & Management of Aquatic Ecosystems*, *(419)*, 34.

**Aisa, E., Desideri, L., Guerrieri, P., & Bonelli, P.** (1983). Further information on fish parasites from Lake Trasimeno. II. Observations on the population of *Scardinius erythrophthalmus* L. *Bollettino Della Societa Italiana di Biologia Sperimentale*, *59*(9), 1383-1389.

**Akçimen, U., Ceylan, M., Bulut, C., & Meke, T.** (2012). *Sücüllü Baraj Göletindeki (Yalvaç, Isparta) bir gökkuşağı alabalığı işletmesinde görülen Lernaea cyprinacea enfestasyonu*. In 5th Limnology Symposium, 27–29 August 2012, Isparta. (In Turkish).

**Al-Abbadie, F. A. M.** (2006). Preliminary study on the parasites of some freshwater fishes in Al-Gharraf River, southern Iraq. *Journal of Thi Qar University, 2*(3), 17–27. (In Arabic).

**Al-Ali, M. F. M., Atte, R. S., & Banay, M. A.** (2013). Activity of the pesticide Sumcidin on the copepods *Lernaea cyprinacea* and *Tetrahymena corlissi* infecting *Cyprinus carpio* and *Gambusia affinis* fishes. *Diyala Agricultural Sciences Journal*, *5*(1), 24–31. (In Arabic).

**Al-Alusi, M. A.-S.** (1998). *A study of some biological aspects and parasites of the mugilid fish Liza abu (Heckel) in Alus Region, upper Euphrates River, Anbar Province* (Ph.D. thesis). College of Science, Al-Mustansirya University, Iraq, 121 pp. (In Arabic).

**Al-Alusi, M. A.-S.** (2010). Use of some chemicals in treating the copepod crustaceans *Ergasilus mosulensis* and *E. rostralis* parasitized on gills of *Liza abu*. *Journal of Kerbala University*, 8(2), 61-71. (In Arabic).

**Al-Aubaidi, I.K., Mhaisen, F.T., & Balasem, A.N.** (1999). The external parasites of the common carp (*Cyprinus carpio*) in Al-Zaafaraniya fish farm, Baghdad. *Ibn Al-Haitham Journal of Pure and Applied Science, 12*(1), 32-40.

**Al-Daraji, S. A. M.** (1995). *Taxonomical and ecological studies on the metazoan parasites of some marine fishes of Khor Al-Zubair Estuary, north-west of the Arabian Gulf* (Ph.D. thesis). College of Agriculture, University of Basrah, Iraq.

**Al-Daraji, S. A. M.** (2002b). Description of *Ergasilus pararostralis* new species (Copepoda: Poecilostomatoida) infesting *Liza subviridis* (Valenciennes, 1836) in Khor Al-Zubair Lagoon, Iraq. *Marshes Mesopotamica, 17*(1), 155–162.

**Al-Daraji, S. A. M.** (2002c). *Ergasilus irakiensis* new species (Copepoda: Poecilostomatoida) from *Liza subviridis* (Valenciennes, 1836) in Iraq. *Marshes of Mesopotamia (Mar. Mesopot.)*, *17*(2), 341–346.

**Al-Dulaimi, F. H. A., Al-Hamiary, A. K. A., & Al-Khafaji, A. H. A.** (2006). Infection of *Liza abu* with parasitic crustaceans in waters of a fish farm in Al-Eskandaria District, Babylon Province. *Journal of Babylon University, Pure Sciences*, *12*(3), 749–758. (In Arabic).

**Al-Hajimi, Y. M. M.** (2021). Survey study on the parasitic on gills and intestine of some fishes of Karbala Main Drainage. (Ph.D. thesis). Al-Mussaib Technical College, Al-Furat Al-Awsat Technical University, 90 pp. (In Arabic).

**Al-Hamdane, A. H., & Azziz, A. H.** (2006). Study of some parasitic diseases of pond fish in Mosul area. *Iraqi Journal of Veterinary Sciences, 20*(2), 225–228. (In Arabic).

**Al-Hamed, M. I., & Hermiz, L.** (1973). Experiments on the control of anchor worm (*Lernaea cyprinacea*). *Aquaculture*, *2*, 45–51.

**Al-Helli, A. M. S**. (2019). *Fish assemblage structure and some of its environmental and health aspects in Euphrates River near Samawa City* (Ph.D. thesis). College of Agriculture, University of Basrah, Iraq. (In Arabic).

**Al-Jadoaa, N. A. A.** (2002). *The parasitic infections and pathological changes of some local and cultured fishes from Al-Qadisiya and Babylon provinces* (Ph. D. Thesis). College of Education, Al-Qadisiya University, Iraq. (In Arabic)

**Al-Janabi, M. I. G.** (2010a). The relationship between feeding type and exposure to infection with some ectoparasites of *Marmarij Mastacembelus mastacebelus* in Baghdad. *Iraqi Journal of Veterinary Medicine, 34*(2), 170–175. (In Arabic).

**Al-Janabi, M. I. G.** (2010b). The relation between sex and ectoparasites infestation of *Albushlumbo Pseudapocrypte dentatus* in Iraq. *Al-Anbar Journal of Veterinary Sciences, 3*(2), 89–96. (In Arabic).

**Al-Jawda, J. M., Balasem, A. N., Mhaisen, F. T., & Al-Khateeb, G. H.** (2000). Parasitic fauna of fishes from Tigris River at Salah Al-Deen Province, Iraq. *Iraqi Journal of Biological Sciences, 19–20*, 16–24.

**Al-Jawda, J. M., Balasem, A. N., Mhaisen, F. T., Al-Shaikh, S. M. J., Asmar, K. R., & Adday, T. K.** (2003). Some fish parasites from Tigris River at Nineveh Province, north of Iraq. *Basrah Journal of Agricultural Sciences, 16*(2), 19–29.

**Al-Jubory, A.A.E.** (2009). Influence of fertilizing the ponds of *Cyprinus carpio* on the infection with *Lernaea cyprinacea*. Higher Diploma Research Project, Al-Musayab Technical College, Foundation of Technical Education, 41 pp. (In Arabic).

**Al-Khenifsawy, R. H. A., & Al-Mayli, H. M. H.** (2022). Detection of internal and external parasites of some common fish species from Al-Dalmaj Marsh/Iraq. *International Journal of Entomological Research, 7*(2), 37-47.

**Al Malki, S.** (2021). Drastic parasitic infestations among cultured tilapias at El-Abbassa fish farms, Egypt, with respect to stressors of abiotic factors. *Egyptian Journal of Aquatic Biology and Fisheries, 25*(3), 281–295.

**Al-Marjan, K. S. N.** (2016). Seasonal variations and prevalence of infections of some species of ectoparasites affecting freshwater fish, *Chondrostoma regium* from Greater Zab River, Kurdistan Region, Iraq. *PolyTechnic, 6*(1), 310–315.

**Al-Marjan, K. S. N., & Abdullah, S. M. A.** (2008). Experimental study of the life cycle of the anchor worm *Lernaea cyprinacea* Linnaeus, 1758. *Journal of Duhok University*, 11(2), 110–116.

**Al-Marjan, K. S. N., & Abdullah, S. M. A.** (2009). Some ectoparasites of the common carp (*Cyprinus carpio*) in Ainkawa fish hatchery, Erbil province. *Journal of Duhok University*, 12(1), 102–107.

**Al-Mosawi, M. H., & Adday, T. K.** (2024). First record of *Mugilicola bulbosa* Tripathi, 1960 (Ergasilidae: Cyclopoida) on the gills of greenback mullet *Planiliza subviridis* (Valenciennes) from Shatt Al-Arab River, southern Iraq. *Basrah Journal of Agricultural Sciences, 37*(2), 67–77.

**Al-Nasiri, F. S., Mhaisen, F. T., & Al-Nasiri, S. K.** (2001). First record of the ectoparasitic crustacean *Lernaea oryzophila* Monod, 1932 (Copepoda: Lernaeidae) in Iraq on the common carp *Cyprinus carpio*. *Al-Mustansiriya Journal of Science*, *12*(7), 643-648.

**Al-Nasiri, F. S., Mhaisen, F. T., & Al-Nasiri, S. K.** (2002). Parasitic infections of the common carp, *Cyprinus carpio*, in a man-made lake at Baghdad Region. *Iraqi Journal of Agriculture (Special Issue), 7*(1), 175–181.

**Al-Nasiri, F. S., Mhaisen, F. T., & Al-Nasiri, S. K.** (2003). Parasites of the grey mullet *Liza abu* in a man-made lake at Baghdad Region. *Iraqi Journal of Agriculture* (Special Issue), *8*(1), 133–140.

**Al-Nasiri, F. S., Ho, J.-S., & Mhaisen, F. T.** (2012). *Pseudolamproglena boxshalli* sp. n. (Lernaeidae: Lamprogleninae) parasitic on gills of *Cyprinion macrostomum* (Teleostei: Cyprinidae) from Tigris River, Iraq. *Folia Parasitologica*, 59(4), 308-310.

**Al-Niaeem, K. S. K.** (2006). *Infection distribution of fish parasites in Basrah Province and pathological effects of Saprolegnia sp. and its susceptibility to some plant extracts* (Ph.D. thesis), College of Agriculture, University of Basrah, Iraq, 172 pp. (In Arabic).

**Al-Niaeem, K. S., Al-Saboonchi, A. A., & Ahmed, R. A.** (2015). Effect of water quality on fishes infected with copepods from three stations in Basrah province, Iraq. *Journal of International Academic Research for Multidisciplinary*, *3*(4), 428-436.

**Al-Rubaie, I. A., Mohammad-Ali, N. R., Balasem, A. N., & Al-Jawda, J. M.** (2003). Study on fish diseases in Diyala River. *Iraqi Journal of Agriculture (Special Issue)*, 8(6), 40-47. (In Arabic).

**Al-Rubaie, A. L., Hussain, H. T., & Abdul-Ameer, K. N.** (2007). The external parasites of the common carp (*Cyprinus carpio*) in Technical Institute of Al-Mussayab Fish Farm. *Journal of Babylon University, Science, 14*(3), 46–50.

**Al-Sa'adi, B. A. H.** (2022). *Leuciscus vorax* Heckel, 1848 fish as a new host of two parasites, crustacean parasite *Lamproglena chinensis* and the excysted metacercaria *Centrocestus formosanus* phase for the first time in Iraq. *Journal of University of Babylon for Pure and Applied Sciences*, 63–66.

**Al-Saadi, A. A. J.** (2007). *Ecology and taxonomy of parasites of some fishes and biology of Liza abu from Al-Husainia Creek in Karbala Province, Iraq* (Ph.D. thesis). College of Education (Ibn Al-Haitham), University of Baghdad, Iraq. (In Arabic).

**Al-Saadi, A. A. J., Mhaisen, F. T., & Hasan, H. R.** (2010). Ectoparasites of seven fish species from Al-Husainia Creek, Karbala Province, Mid Iraq. *Journal of Kerbala University*, *8*(4), 1–7.

**Al-Saadi, A. A. J., Mhaisen, F. T., & Hasan, H. R.** (2011). Some aspects of the reproductive biology of the mugilid fish *Liza abu* (Heckel) in Al-Husainia Creek, Karbala Province, Iraq and effect of parasitism on its gonadosomatic index. *Iraqi Journal of Agricultural Research* (Special Issue), *16*(3), 166–173. (In Arabic).

**Al-Saboonchi, A. A., Al-Niaeem, K. S., & Al-Janae'e, A. M.** (2009). Effect of organically polluted water on common carp *Cyprinus carpio* infected with the anchor worm *Lernaea cyprinacea*. In *Proceedings of the 3rd Scientific Conference, College of Science, University of Baghdad* (pp. 252–259). Baghdad, Iraq, March 24–26, 2009.

**Al-Sahlany, B. A., Ali, A. H., & Adday, T. K.** (2024). *Ergasilus luteusi* Al-Sahlany, Adday et Ali, 2024 (Cyclopoida: Ergasilidae) parasite of two fishes and effect of length groups, gender, and season in Al-Gharraf River, Southern Iraq. *University of Thi-Qar Journal of Agricultural Research, 13*(2), 176-188.

**Al-Salim, N. K., & Mohamad, E. T.** (1993a). Infection of *Heteropneustes fossilis* with some parasite species and their pathological effects on the infected fishes. *First Scientific Symposium, College of Agriculture, University of Basrah*, 12–13 April 1993, 17. (Abstract, in Arabic).

**Al-Salim, N. K., & Mohamad, E. T.** (1993b). Seasonal variation of infection of *Heteropneustes fossilis* with some parasite species. *First Scientific Symposium, College of Agriculture, University of Basrah*, 12–13 April 1993, 18. (Abstract, in Arabic).

**Al-Salim, N. K., & Jori, M. M.** (2002). Study of the parasites of two mugilid fish species and the effect of some of them on the blood parameters. II: Effect of the crustacean *Ergasilus rostralis* on blood parameters of *Liza abu* and *L. subviridis*. *Marshes Mesopotamica, 17*(1), 209–221. (In Arabic).

**Al-Salim, N. K., Khamees, N. R., & Al-Niaeem, K. S.** (2007). Seasonality of some parasites found in three species of fishes in two different localities. *Journal of Basrah Research, Special Issue, Third Scientific Conference on Microbiology*, 32–46. (In Arabic).

**Al-Salmany, S. O. K.** (2022). *Isolation and diagnosis of helminths and crustacea parasitizing in some fish species caught from determinant areas of Upper Euphrates River/ Al-Anbar Province, Iraq* (Ph.D. thesis). College of Science, University of Tikrit, Iraq. (In Arabic).

**Al-Shaikh, S. M. J., & Al-Sardee, H. M. J.** (1993). Gross pathological and histological changes in infection with the anchor worm and its treatment with quicklime. *Al-Tharwa Al-Samakia*, 13, 95-97. (In Arabic).

**Al-Shaikh, S. M., Mhaisen, F. T., Al-Khateeb, G. H., Balasem, A. N., & Mansoor, N. T.** (1995). Collection of some fish parasites from the lower reaches of Diyala River, Mid Iraq. *Journal of Environmental Science and Health, A, 30*(8), 1707–1715.

**Al-Tamimi, S. S. J.** (2001). *Efficacity of formalin, chemogos insecticide and some plant extract in treating the common carp, Cyprinus carpio, infested with monogenetic trematodes* (Ph. D. thesis). College of Education (Ibn Al-Haitham), University of Baghdad, Iraq. 99 pp. (In Arabic).

**Al-Tayyar, O. B. A., Abdul Jabaar, W., Raof, S. A., & Shather, M. M.** (2011). Isolation of *Ichthyophthirius multifiliis*, *Gyrodactylus*, and Copepoda: *Lernaeidae* species from six ornamental fish species for the first time in Iraq. *Al-Anbar Journal of Veterinary Sciences*, *4*(2), 82-87.

**Al-Zubaidy, A. B.** (1998). Studies on the parasitic fauna of carps in Al-Furat Fish Farm, Babylon Province, Iraq. (Ph.D. thesis). College of Science, University of Babylon, Iraq, 141 pp. (In Arabic).

**Al-Zubaidy, A. B.** (2007). A study on some crustacean fish parasites from fresh and marine water. *Journal of the Egyptian Academy of Society for Environmental Development, 8*(2), 127–137.

**Alas, A., Öktener, A., & Türker, D. Ç.** (2015). Review of parasitic copepods recorded in fish from Turkey/Übersicht parasitärer in Fischen festgestellter Copepoden/Recenzia copepodelor parazite pe pesti înregistrate în Turcia. *Transylvanian Review of Systematical and Ecological Research, 17*(1), 39–62.

**Alekseev, V., Cuoc, C., Jamet, D., Jamet, J. L., & Chappaz, R.** (2021). Biological invasion of fish parasite *Neoergasilus japonicus* (Harada, 1930) (Copepoda: Ergasilidae) in Lake Grand Laoucien, France: A field study on life cycle parameters and reasons for unusual high population density. *Life, 11*(10), 1100.

**Alfonso, G., & Belmonte, G.** (2010). *Neoergasilus japonicus* (Harada, 1930): A new non-indigenous copepod for the Italian fauna. *Italian Journal of Zoology, 77*(2), 172–178.

**Ali, M. D.** (1985). Observations on lernaeosis and gyrodactylosis in carp fingerlings raised in ponds. *Journal of Biological Sciences Research, 16*(1), 125-132.

**Ali, M. D.** (1986). Anchor worm *Lernaea cyprinacea* and its control. *Journal of Biological Sciences Research, 17*(2), 131-139. (In Arabic).

**Ali, M. D., & Shaaban, F.** (1984). Some species of parasites of freshwater fish raised in ponds and in Tigris–Al-Tharthar Canal Region. *Proceedings of the Seventh Scientific Conference of the Iraqi Veterinary Medical Association*, Mosul, 23–25 October 1984, 44–46. (Abstract).

**Ali, M. D.** (2002). *A survey on health and diseases of carp fish raised in fish culture projects/Erbil, Duhok and Suliemanyia region & other activities* (Report). FAO Representation in Iraq, FAO Coordination Office for Northern Iraq – Animal Production Unit.

**Ali, N. M., Al-Jafery, A. R., & Abdul-Ameer, K. N.** (1987). Parasitic fauna of freshwater fishes in Diyala River, Iraq. *Journal of Biological Science Research, 18*(1), 163–181.

**Ali, N. M., Abul-Eis, E. S., & Abdul-Ameer, K. N**. (1988a). Study on the parasites of common carp *Cyprinus carpio* and other freshwater fishes in Habbaniyah Lake, Iraq. *Journal of Biological Science Research, 19*(2), 395–407.

**Ali, N. M., Salih, N. E., & Abdul-Ameer, K. N.** (1988b). Protozoa and crustacea infesting three species of carp raised in ponds in Iraq. *Journal of Biological Science Research, 19*(2), 387–394.

**Ali, A. H., & Adday, T. K.** (2019). Description of a new species of *Dermoergasilus* Ho & Do, 1982 (Copepoda: Ergasilidae) from the redbelly tilapia *Coptodon zillii* (Gervais) (Perciformes: Cichlidae) in Basrah, southern Iraq. *Systematic Parasitology*, *96*, 715-722.

**Almeida, D., Almodóvar, A., Nicola, G. G., & Elvira, B.** (2008). Fluctuating asymmetry, abnormalities and parasitism as indicators of environmental stress in cultured stocks of goldfish and carp. *Aquaculture, 279*(1-4), 120-125.

**Almeida, D., Alcaraz-Hernández, J. D., Cruz, A., Lantero, E., Fletcher, D. H., & García-Berthou, E.** (2024). Seasonal effects on health status and parasitological traits of an invasive minnow in Iberian waters. *Animals, 14*(10), 1502.

**Altunel, F. N.** (1979). Parasitisme chez quelques anguilles (*Anguilla anguilla* L.) du lac de Bafa. *Rapports et procès-verbaux des réunions, Commission internationale pour l'Exploration scientifique de la mer Méditerranée*, *10*, 25–26. (In French).

**Altunel, F. N.** (1980). Examinations on parasites of eel (*Anguilla anguilla* L., 1758). In *Proceedings of the Science Congress (Biology Section)*, 6–10 October, Aydın, 11.

**Altunel, F. N.** (1983). Parasitism on the mullets. In *Proceedings of the 1st National Congress on Marine and Freshwater Research*, *Ege University, Journal of Science Faculty, Series B*, *1*, 364–378. (In Turkish).

**Altunel, F. N.** (1990). Karacabey Ekinli Lagünü yılan balıklarında rastlanılan metazoon parazitler. In *X. Ulusal Biyoloji Kongresi, Zooloji Bildiriler Kitabı* (pp. 27–35). (In Turkish).

**Álvarez Pellitero, M. P., Pereira Bueno, J. M., & González Lanza, M. C.** (1979). Myxosporidios de *Carassius carassius* y *Cyprinus carpio* en el río Esla (León). *Revista Ibérica de Parasitología*, *39*(1-4), 611–623. (In Spanish).

**Amado, M. A. P. M., Rocha, C. E. F., Piasecki, W., Al-Daraji, S. A. M., & Mhaisen, F. T.** (2001). Copepods of the family Ergasilidae (Poecilostomatoida) parasitic on fishes from Khor Al-Zubair Lagoon, Iraq. *Hydrobiologia*, *459*, 213–221.

**Asadzadeh Mangili, A., Mokhayyer, B., & Jalali, B.** (2000). Health assessment of external parasites of cultured Cyprinidae in pen culture of Anzali Lagoon. *Pajohesh and Sazandegi, 47*, 96-101. (In Persian).

**Asmar, K. R., Balasem, A. N., Mhaisen, F. T., Al-Khateeb, G. H., & Al-Jawda, J. M.** (1999). Survey of the parasites of some fish species from Al-Qadisiya Dam Lake, Iraq. *Ibn Al-Haitham Journal for Pure and Applied Science, 12*(1), 52–61.

**Asmar, K. R., Balasem, A. N., Adday, T. K., & Al-Jawda, J. M.** (2003). Parasitic infections in some lotic water systems in Mid Iraq. *Iraqi Journal of Agriculture (Special Issue), 8*(6), 59–65. (In Arabic).

**Asmar, K. R., Balasem, A. N., Al-Jawda, J. M., & Adday, T. K.** (2004). Recording of parasitic and fungal infections in three fish farms, south of Baghdad. *Iraqi Journal of Aquaculture*, 2, 117-132. (In Arabic).

**Awad, A. H., Abdullah, F. A., & Jori, M. M.** (2007a). Taxonomic study of parasites of the Asian catfish *Silurus triostegus* (Heckel, 1843) from Al-Hammar Marsh, Basrah, Iraq. In *Proceedings of the Second Scientific Conference on the Rehabilitation of the Southern Iraqi Marshes* (p. 32, Abstract). Basrah, Iraq, April 2–4, 2007. (In Arabic).

**Awad, A. H., Abdullah, F. A., & Jori, M. M.** (2007b). Immunological study on the Asian catfish *Silurus triostegus* (Heckel, 1843) from Al-Hammar Marsh, Basrah, Iraq. In *Second Scientific Conference on the Rehabilitation of the Southern Iraqi Marshes* (pp. 63). Basrah, Iraq. (Abstract). (In Arabic).

**Awad, A. H., Abdullah, F. A., & Jori, M. M.** (2007c). Effect of two parasites on blood parameters of the Asian catfish *Silurus triostegus* (Heckel, 1843). In *Second Scientific Conference on the Rehabilitation of the Southern Iraqi Marshes* (pp. 67). Basrah, Iraq. (Abstract). (In Arabic).

**Aydogdu, A., Ozturk, M. O., Oğuz, M. C., & Altunel, F. N. (**2001). Investigations on metazoan parasites of common carp (*Cyprinus carpio* L. 1758) in Dalyan Lagoon, Karacabey, Turkey. *Acta Veterinaria (Beograd), 51*(5/6), 351–358.

**Baek, S. Y., Jang, K. H., Choi, E. H., Ryu, S. H., Kim, S. K., Lee, J. H., Lim, Y. J., Lee, J., Jun, J., Kwak, M., Lee, Y.-S., Hwang, J.-S., Maran, B. A. V., Chang, C. Y., Kim, I.-H., & Hwang, U. W.** (2016). DNA barcoding of metazoan zooplankton copepods from South Korea. *PLoS ONE, 11*(12), e0157307.

**Bakaria, F., Belhaoues, S., Djebbari, N., Tahri, M., Ladjama, I., & Bensaad, L.** (2018). Metazoan parasites and health state of European eel, *Anguilla anguilla* (Anguilliformes, Anguillidae), from Tonga Lake and El Mellah lagoon in the northeast of Algeria. *Vestnik Zoologii, 52*(4), 279–288.

**Balasem, A. N., Mhaisen, F. T., Al-Shaikh, S. M. J., Al-Khateeb, G. H., Asmar, K. R., & Adday, T. K.** (1993). Survey of fish parasites from Tigris River at Al-Zaafaraniya, south of Baghdad, Iraq. *Marine Mesopotamia, 8*(3), 226–235.

**Balasem, A. N., Mustafa, S. R., Salih, A. M., Al-Jawda, J. M., & Mohammad-Ali, N. R.** (2001). A second study for fish parasites of Diyala River. *Al-Fatah Journal*, *10*, 457-470. (In Arabic).

**Balasem, A. N., Mhaisen, F. T., Al-Jawda, J. M., & Asmar, K. R.** (2002a). Collection of some fish parasites from the northern sector of Saddam’s River, mid Iraq. *Scientific Journal of the Iraqi Atomic Energy Commission, 4*(2), 186–191.

**Balasem, A. N., Mhaisen, F. T., Al-Jawda, J. M., Asmar, K. R., & Adday, T. K.** (2002b). Parasitic fauna of some fishes in northern sector of Saddam’s River at Al-Mahmoodiya City, Iraq. *Al-Tharwa Al-Samakia*, *21*, 43–48. (In Arabic).

**Balasem, A. N., Mhaisen, F. T., Adday, T. K., Al-Jawda, J. M., & Asmar, K. R.** (2003). A second survey of parasitic infections in freshwater fishes from Al-Qadisiya Dam Lake, Euphrates River, Iraq. *Marine Mesopotamia*, *18*(2), 123–140. (In Arabic).

**Bannai, M.A.A., Al-Ali, M.F.M., & Hwait, A.R.** (2007). Therapeutic activity of sumcidin against the parasitic crustacean *Lernaea cyprinacea* and the parasitic protozoan *Tetrahymena corlissi* which infects eggs and larvae of cultured *Cyprinus carpio* and *Gambusia affinis* with notification of histopathological effects caused by the insecticide. *2nd Scientific Conference Rehabiitation of Southern Iraqi Marshes*, Basrah, 2-4 April 2007, 50. (In Arabic).

**Bao, M., Costal, D., Garci, M. E., Pascual, S., & Hastie, L. C.** (2016). Sea lice (*Lepeophtheirus salmonis*) and anchor worms (*Lernaea cyprinacea*) found on sea trout (*Salmo trutta*) in the River Minho catchment, an important area for conservation in NW Spain. *Aquatic Conservation: Marine and Freshwater Ecosystems*, *26*(2), 386–391.

**Barzegar, M., & Jalali, B.** (2000). Parasites of Kaftar Lake fishes, their geographical distribution and economical importance (In Persian). *Scientific Journal of the School of Veterinary Medicine, Shahid Chamran University, 3*(5), 52–62.

**Barzegar, M., Asadollah, S., Hemmat-zadeh, A., Rahnama, R., & Jalali, B.** (2004). Parasites of Behesht Abad River (Chaharmahal O Bakhtiary). *Iranian Journal of Veterinary Sciences, 1*(1), 67–74. (In Persian).

**Barzegar, M., & Jalali, B.** (2009). Crustacean parasites of fresh and brackish (Caspian Sea) water fishes of Iran. *Journal of Agricultural Science and Technology, 11*(2), 161–171.

**Bdair, A. T., & Al-Rudainy, A. A. J.** (2018). Digenetic trematodes and crustacean parasitising some fishes from the Tigris River, Al-Zaafaraniya Region, Baghdad City. *Journal of Entomology and Zoology Studies, 6*(1), 681–685.

**Ben Hassine, O. K.** (1983). *Les copépodes parasites de poissons Mugilidae en Méditerranée occidentale (côtes françaises et tunisiennes). Morphologie, bio-écologie, cycles évolutifs* (Ph.D. thesis). Université des Sciences et Techniques du Languedoc, Montpellier, France, 471 pp.

**Berrouk, H., Tolba, M., Boucenna, I., Touarfia, M., Bensouilah, M., Kaouachi, N., & Boualleg, C.** (2018). Copepod parasites of the gills of *Luciobarbus callensis* (Valencienne, 1842) and *Carassius carassius* (Linnaeus, 1758) (Cyprinid fish) collected from Beni Haroun Dam (Mila, Algeria). *World Journal of Environmental Biosciences*, *7*(4).

**Berrouk, H.** (2019). *Étude des crustacés ectoparasites branchiaux de l’ichtyofaune dulçaquicole du barrage Béni-Haroun-Mila* (Ph.D. thesis). University of Souk Ahras, Algeria.

**Berrouk, H., Khelifi, N., Tourafia, M., & Boualleg, C.** (2019). Effect of abiotic factors on copepod parasites from Beni-Haroun Dam (Mila City), north-east of Algeria. *Journal of Harmonized Research in Applied Sciences, 7*(4), 112–122.

**Berrouk, H., Sahtout, F., Kaouachi, N., & Boualleg, C.** (2021). Effect of parasitic copepods on the growth of *Abramis brama* fish from Beni-Haroun dam of Mila city (Northeast Algeria). *Ukrainian Journal of Ecology, 11*(8), 79–88.

**Berrouk, H., Sid, A., Lahoual, A., Sahtout, F., Kaouachi, N., & Boualleg, C.** (2022). Effect of parasitic copepods on the length–weight relationship and the condition factor of crucian carp (*Carassius carassius*) in the Beni-Haroun Dam, Mila City, Northeast Algeria. *Animal Research International, 19*(3), 4625–4633.

**Bilal, S. J., & Abdullah, S. M. A.** (2008). Protozoa and Crustacea infesting some cyprinid fishes from Bahdinan River in Kurdistan Region–Iraq. *Journal of Duhok University*, *12*(1, Special Issue), 108–112.

**Blazhekovikj-Dimovska, D., & Stojanovski, S.** (2022). Parasitic copepods on common carp (*Cyprinus carpio*, L. 1758) from Gradche Reservoir (Macedonia). *Journal of Fisheries Science*, *4*(1), 61-67.

**Boni, P., Alborali, G. L., Zanardi, G., Cappellaro, H., & Fioravanti, M. L.** (1992). Recovery of *Lamproglena pulchella* (Nordmann, 1832) in Cyprinidae fishes from some lakes of Brescia (Italy). *Bollettino della Società Italiana di Patologia Animale, 8*, 27–32.

**Borji, H., Naghibi, A., Nasiri, M. R., & Ahmadi, A.** (2012). Identification of *Dactylogyrus* spp. and other parasites of common carp in northeast of Iran. *Journal of Parasitic Diseases, 36*, 234-238.

**Boucenna, I., Boualleg, C., Kaouachi, N., Allalgua, A., Menasria, A., Maazi, M. C., Barour, C., & Bensouilah, M.** (2015). L’infestation de la population de *Cyprinus carpio* (Linnaeus, 1758) par les copépodes parasites dans le barrage Foum El Khanga (Souk Ahras, Algérie). *Bulletin de la Société Zoologique de France, 140*(3), 163–179.

**Boucenna, I., Khelifi, N., Boualleg, C., Allalgua, A., & Bensouilah, M.** (2018). L'infestation de *Luciobarbus callensis* (Cyprinidés) par les copépodes parasites dans le barrage Foum El Khanga (Souk-Ahras, Algérie). *Bulletin de la Société zoologique de France*, *143*(4).

**Boxshall, G. A.** (1976). Copepod parasitic on freshwater fishes. *Bulletin of the British Museum (Natural History) Zoology*, *30*(6).

**Bozorgnia, A., Sharifi, N., Youssefi, M., & Barzegar, M.** (2018). *Acipenser stellatus* as a new host record for *Lernaea cyprinacea* Linnaeus, 1758 (Crustacea; Copepoda), a parasite of freshwater fishes in Iran. *Journal of Aquaculture & Marine Biology, 7*(3), 123–125.

**Braun, M.** (1981). *Contribution à l’étude biologique des zones à salinité variable du littoral Méditerranéen français: Copépodes parasites de Mugilidés* (PhD. thesis). Université des Sciences et Techniques du Languedoc, Montpellier, France, 88 pp.

**Burgu, A., Oğuz, T., Körtıng, W., & Güralp, N.** (1988). İç Anadolu'nun bazı yörelerinde tatlısu balıklarının parazitleri. *Etlik Veteriner Mikrobiyoloji Dergisi, 6*(3), 143–166. (In Turkish).

**Caillot, C., Morand, S., Mullergraf, C. M., Faliex, E., & Marchand, B.** (1999). Parasites of *Dicentrarchus labrax*, *Anguilla anguilla*, and *Mugil cephalus* from a pond in Corsica, France. *Journal of the Helminthological Society of Washington*, *66*, 95–98.

**Cengizler, I., & Goksu, M. Z. L.** (1994). Balıklıağ Çayı'nda (Adana) yaşayan iki Cyprinid türünde rastlanan bazı metazoan parazitler. *XII. National Biology Conference*, IV, 362–365. (In Turkish).

**Clavero, M., Esquivias, J., Qninba, A., Riesco, M., Calzada, J., Ribeiro, F., Fernández, N., & Delibes, M.** (2015). Fish invading deserts: Non-native species in arid Moroccan rivers. *Aquatic Conservation: Marine and Freshwater Ecosystems, 25*(1), 49–60.

**Čož-Rakovac, R., Strunjak-Perović, I., Popović, N. T., Hacmanjek, M., Šimpraga, B., & Teskeredžić, E.** (2002). Health status of wild and cultured sea bass in the northern Adriatic Sea. *Veterinary Medicine-Czech*, 47, 222–226.

**Daghigh Roohi, J., Sattari, M., Asgharnia, M., & Rufchaei, R.** (2014). Occurrence and intensity of parasites in European catfish, *Silurus glanis* L., 1758 from the Anzali Wetland, southwest of the Caspian Sea, Iran. *Croatian Journal of Fisheries: Ribarstvo*, *72*(1), 25–31.

**Daoud, M. S., Al-Hamdani, A. H., & Al-Bakry, H. S.** (2005). Occurrence of the anchor worm *Lernaea cyprinacea* on *Cyprinus carpio* in Mosul, Iraq. *Iraqi Journal of Veterinary Sciences, 19*(2), 155–156. (In Arabic).

**Demir, S., & Karakişi, H.** (2014). Metazoan parasites of nase (*Chondrostoma nasus* L., 1758) from Tahtalı Dam Lake (İzmir Province, Turkey). *Biharean Biologist*, *8*(2), 95-97.

**Demir, S., & Karakişi, H.** (2016). Metazoan parasite fauna of the prussian carp, *Carassius gibelio* (Bloch, 1782) (Cyprinidae), from Marmara Lake, Turkey. *Acta Zoologica Bulgarica, 68*(2), 265-268.

**Dezfuli, B. S., Giari, L., Lui, A., Lorenzoni, M., & Noga, E. J.** (2011). Mast cell responses to *Ergasilus* (Copepoda), a gill ectoparasite of sea bream. *Fish & Shellfish Immunology, 30*(4-5), 1087-1094.

**Dolšak, I.** (2018). *Health status of common carp (i.e. carp fry, one-year old and two-year old carp) on fish farm Poljana*. University of Zagreb: Faculty of Veterinary Medicine, Department for Biology and Pathology of Fish and Bees. 33 p. (In Croatian with English abstract).

**Dos Santos, Q. M., Avenant-Oldewage, A., Piasecki, W., Molnár, K., Sellyei, B., & Székely, C.** (2021). An alien parasite affects local fauna—Confirmation of *Sinergasilus major* (Copepoda: Ergasilidae) switching hosts and infecting native *Silurus glanis* (Actinopterygii: Siluridae) in Hungary. *International Journal for Parasitology: Parasites and Wildlife, 15*, 127–131.

**Dos Santos, Q. M., Rindoria, N. M., & Avenant-Oldewage, A.** (2023). Genetic characterisation of four *Lamproglena* spp. (Copepoda, Lernaeidae) from Africa and the first mitochondrial data. *Folia Parasitologica, 70*, 1–11.

**Easa, M., El-Fasial, M., & Imam, E. A.** (1989). A recurrent epizootic of ergasiliosis in Tilapia (*Oreochromis niloticus* and *Tilapia zilli*) and the grey mullet (*Liza ramada*) in a brackish water fish farm at Port Said, Egypt. *Veterinary Medicine Journal, Giza, 34*(3), 361–372.

**Eassa, A. M., Al-Jenaei, A. M., Abdul-Nabi, Z. A., Abood, M. A., Kzaal, R. S., & Aliwy, Y. J.** (2014). Comparative ecological study of pathogens structure between wild and cultured common carp *Cyprinus carpio* L. in Basrah. *Marsh Bulletin, 9*(2), 107–123.

**Elahi, M. E., Soltani, M., Nokhbe, Z. D., Ghafari, M., & Naderi, M.** (2014). Study of parasites in skin and gill of *Schizothorax zarudnyi* in Sistan region. *Journal of Aquatic Animal and Fish*, *18*, 1–11.

**El-Khatib, N. R., & El-Hady, M. A.** (2012). The relationship between *Lernaea cyprinacea* infestations in *Cyprinus carpio* and its concurrent bacterial diseases in Egypt. *Abbassa International Journal of Aquaculture, 5*(1), 398-414.

**El-Moghazy, D. F.** (2008). *Studies on some parasitic diseases caused by harmful crustacean in fish* (Ph.D. thesis). Faculty of Veterinary Medicine, Suez Canal University, Egypt.

**El-Rashidy, H. H., & Boxshall, G. A.** (2000). Description of a new *Nipergasilus* species (Family: Ergasilidae) from the gills of grey mullet. *Hydrobiologia, 428*, 61–66.

**El-Seify, M. A., Elshahawy, I. S., Metwally, A. M., & Fwaz, M. M**. (2013). A survey on ectoparasites of some freshwater fish at Qena governorate, upper Egypt. *Kafrelsheikh Veterinary Medical Journal*, *11*(2), 85-106.

**Erbatur, İ., Yağcı, A., Öktener, A., & Akcimen, U.** (2018). New locality for *Lernaea cyprinacea* (Copepoda; Lernaeidae) in Turkey. *13th International Symposium on Fisheries and Aquatic Sciences Proceedings*.

**Esmaeel, H. M., Owaied, Y. H., & Mahmoud, A. J.** (2023). Influence of health-related factors on water quality and prevalence of ecto- and endoparasites in fish within the Tigris River spanning Tikrit City. *Journal of Survey in Fisheries Sciences, 10*(3S), 5135–5143.

**Espínola-Novelo, J. F., Solórzano-García, B., Guillén-Hernández, S., Badillo-Alemán, M., Chiappa-Carrara, X., & de León, G. P. P.** (2023). Metazoan parasites of the Ocellated killifish, *Floridichthys polyommus* (Cyprinodontidae) in La Carbonera coastal lagoon, Yucatán, Mexico. *Regional Studies in Marine Science, 67*, 103223.

**Ezz El-Dien, N. M.** (1994). *Studies on ectoparasites of some marine water fish in Suez Canal area* (Ph.D. thesis). Faculty of Veterinary Medicine, Cairo University, Egypt.

**Fadaei Fard, F., Mokhayer, B., & Ghorbani, H.** (2001). Study of fish parasites in Chaghakhour Lagoon (Chaharmahal-o-Bakhtiary, Iran). *Journal of Veterinary Medicine, University of Tehran*, 109-114. (In Persian).

**Fahmy, S. A., Arafa, S. Z., & Hamdan, Z. K.** (2019). Ultrastructure of *Lamproglena pulchella* (Copepoda: Lernaeidae), a gill parasite of the freshwater fish, *Leuciscus vorax* from Tigris River, Iraq. *Egyptian Journal of Aquatic Biology and Fisheries, 23*(4), 385–390.

**Faisal, M., Easa, M. E. S., Shalaby, S. I., & Ibrahim, M. M.** (1988). Epizootics of *Lernaea cyprinacea* (Copepoda: Lernaeidae) in imported cyprinids to Egypt. *Der Tropenlandwirt – Journal of Agriculture in the Tropics and Subtropics*, *89*(2), 131–141.

**Fikiye, P. P., Smit, N. J., Van As, L. L., Truter, M., & Hadfield, K. A.** (2023). Integrative morphological and genetic characterisation of the fish parasitic copepod *Ergasilus mirabilis* Oldewage & Van As, 1987: Insights into host specificity and distribution in Southern Africa. *Diversity, 15*(9), 965.

**Fikiye, P. P., Van As, L. L., Truter, M., Smit, N. J., & Hadfield, K. A.** (2024). A new species of *Neoergasilus* Yin 1956 (Copepoda: Cyclopoida: Ergasilidae) parasitic on the catfish *Clarias gariepinus* (Burchell, 1822) (Siluriformes: Clariidae) from South Africa. *Systematic Parasitology, 101*(5), 64.

**Filippi, J. J., Quilichini, Y., Foata, J., & Marchand, B.** (2013). Influence of site, season, silvering stage, and length on the parasites of the European eel *Anguilla anguilla* in two Mediterranean coastal lagoons of the island of Corsica, France using indicator species method. *Parasitology research*, *112*(8), 2959-2969.

**Fratello, B., & Sabatini, M. A.** (1972). Cariologia e sistematica di *Lernaea cyprinacea* L. (Crustacea, Copepoda). *Atti della Accademia Nazionale dei Lincei. Classe di Scienze Fisiche, Matematiche e Naturali. Rendiconti*, *53*(1-2), 209-213.

**Galli, P., Crosa, G., Albricci, O., Tieghi, K., Cotta Ramusino, M., & Garibaldi, L.** (1998a). Influenza dell'eutrofizzazione sulle popolazioni di parassiti di scardole (*Scardinius erythrophthalmus*). In *II Convegno Nazionale Ecopatologia della Fauna Selvatica* (pp. 60–62). Bormio, Italy.

**Galli, P., Mariniello, L., Crosa, G., Ortis, M., Occhipinti Ambrogi, A., & D'Amelio, S.** (1998b). Populations of *Acanthocephalus anguillae* and *Pomphorhynchus laevis* in different conditions of pollution. *Journal of Helminthology, 72*, 331–335.

**Galli, P., Crosa, G., Bertoglio, D., Mariniello, L., & Ortis, M.** (2001). Populations of *Lamproglena pulchella* von Nordmann 1832 (Copepoda: Eudactylinidae) in cyprinid fish in rivers with different pollution levels. *Journal of Applied Ichthyology, 17*(2), 93–96.

**Garbini, A.** (1898). Fauna. In S. Sormani & G. Moretti (Eds.), *Monografia della Provincia di Verona* (p. 33).

**Geldiay, R., & Balık, S.** (1974). *Ecto and endoparasites found in the freshwater fish of Turkey* (Monograph No. 14). Ege University, The Science Faculty Monographies, Ege University Press. (In Turkish).

**Ghobashy, M., Abou Shafeey, H., & Taeleb, A.** (2018). Guppy (*Poecilia*, Poeciliidae) fish naturally infected with *Lernaea cyprinacea* parasites (Linnaeus, 1758) in KSA. *Parasitologists United Journal, 11*(3), 141–148.

**Grandori, R.** (1925). Nuove specie di Copepodi della Laguna Veneta [New species of Copepods from the Venetian Lagoon]. *Bollettino di Istituto di Zoologia, Reale Università di Roma*, *3*, 38–70, pls. 1–2.

**Gutiérrez-Galindo, J. F., & Lacasa-Millán, M. I.** (2005). Population dynamics of *Lernaea cyprinacea* (Crustacea: Copepoda) on four cyprinid species. *Diseases of Aquatic Organisms, 67*(1–2), 111–114.

**Hassan, E. S., Mahmoud, M. M., Metwally, A. M., & Mokhtar, D. M.** (2013). *Lamproglena monodi* (Copepoda: Lernaeidae), infesting gills of *Oreochromis niloticus* and *Tilapia zillii*. *Global Journal of Fisheries and Aquaculture Research, 6*, 1–16.

**Hassan, M., Nisafi, A., Dayoub, A., Gnede, S., Fadel, M., & Sasal, P.** (2023). First checklist triggering the inventory of marine fish ectoparasites in the Syrian coast (Eastern Mediterranean). *Experimental Results*, 1-17.

**Hermida, M., Saraiva, A., & Cruz, C.** (2008). Metazoan parasite community of a European eel (*Anguilla anguilla*) population from an estuary in Portugal. *Bulletin-European Association of Fish Pathologists*, *28*(1), 35.

**Herzog, P. H.** (1969). Untersuchungen über die Parasiten der Süßwasserfische des Irak. *Archiv für Fischereiwissenschaft, 20*(2/3), 132–147.

**Ho, J.-S., Khamees, N. R., & Mhaisen, F. T.** (1996). Ergasilid copepods (Poecilostomatoida) parasitic on the mullet *Liza abu* in Iraq, with the description of a new species of *Paraergasilus* Markevich, 1973. *Systematic Parasitology*, *33*, 79–87.

**Hua, C. J., Zhang, D., Zou, H., Li, M., Jakovlić, I., Wu, S. G., Wang, G. T., & Li, W. X.** (2019). Morphology is not a reliable taxonomic tool for the genus *Lernaea*: Molecular data and experimental infection reveal that *L. cyprinacea* and *L. cruciata* are conspecific. *Parasites & Vectors, 12*, 1–13.

**Hussain, H. T.** (2007). Survey of ectoparasites of some fishes of Al-Hilla River in Babylon Province. *Journal of Babylon University, Science*, 14(3), 228-232. (In Arabic).

**Hussain, H. T.** (2008). Study on the external ectoparasites of mosquito fish (*Gambusia affinis*) in Al-Hilla river, Babylon province. *Journal of Babylon University, Pure and Applied Sciences, 15*(1), 245-248.

**Hussain, H. T.** (2009). Effect of temperature on the external parasites recorded on the mugilid fish *Liza abu* (Heckel, 1843) in Al-Hilla River, Babylon Province. *Scientific Journal of Kerbala University, 7*(3), 5–11. (In Arabic).

**Hussain, H.T.** (2017). Distribution of infection with ectoparasites of fishes of one private farm in Al-Eskandriya district, Babylon province, Iraq. *Journal of Babylon University, Pure and Applied Sciences*, 25(4), 1329-1335. (In Arabic).

**Hussain, H.T., Hwaidi, E.H., Elewi, H.H., & AbidAli, H.M.** (2011). Survey of ectoparasitic infections on the common carp *Cyprinus carpio* in three fish farms at Al-Eskandriya, Babylon province. *Scientific Journal of Kerbala University*, 9(1), 126-131. (In Arabic).

**Hussein, S. A., Al-Salem, N. K., & Abed, J. M.** (2011). Survey of parasites and fish enemies in three selected fish farms in Iraq. IV: Survey of fish parasites in Basrah University Fish Farm/Marine Science Center. *Iraqi Journal of Agriculture*, *16*(1), 82-89. (In Arabic).

**Ibrahim, M. M., & Soliman, M. F.** (2011). Parasite community of wild and cultured *Oreochromis niloticus* from Lake Manzalah, Egypt. *Journal of the Egyptian Society of Parasitology*, *41*(3), 685–697.

**Illán Aguirre, G.** (2012). *Descripción y caracterización epidemiológica de la parasitofauna de peces ciprínidos de la cuenca alta y media del río Duero* (Ph.D. thesis). Universidad de Zaragoza, Spain.

**Innal, D.** (2020). Detection of ectoparasite *Lernaea cyprinacea* (Copepoda: Lernaeidae) on some cypriniformes fish from the Mediterranean region of Turkey. *Commagene Journal of Biology, 4*(2), 121–125.

**Innal, D., & Avenant-Oldewage, A.** (2012). Occurrence of *Lernaea cyprinacea* on mosquito fish (*Gambusia* spp.) from Kundu Estuary (Antalya-Turkey). *Bulletin of the European Association of Fish Pathologists*, 32(4), 141.

**Innal, D., Avenant-Oldewage, A., Dogangil, B., Stavrescu-Bedivan, M. M., Ozmen, O., & Mavruk, S. İ. N. A. N.** (2017). Susceptibility of endemic and non-indigenous fish to *Lernaea cyprinacea* (Copepoda: Lernaeidae): A case study from Düger Spring Creek (Burdur-Turkey). *Bulletin of the European Association of Fish Pathologists, 37*(3), 100-109.

**Innal, D., Stavrescu-Bedivan, M. M., & Özmen, O.** (2021). Prevalence and histopathological effects of parasitic copepod *Lernaea cyprinacea* in estuarine fishes from the Mediterranean region of Turkey, with a new host record. *Agriculture and Forestry, 67*(4), 165-174.

**Jalali, B**. (1998). *Parasites and parasitic diseases of freshwater fishes of Iran*. Iranian Fisheries Co, 564pp. (In Persian).

**Jalali, B., & Barzegar, M.** (2005). A survey on parasites of gills of fishes of Vahdat Reservoir. *Iranian Journal of Veterinary Sciences, 3*, 41-50. (In Persian).

**Jalali, B., & Barzegar, M.** (2006). Fish parasites in Zarivar Lake. *Journal of Agricultural Science and Technology, 8*, 47-59.

**Jalali, B., Barzegar, M., & Nezamabadi, H.** (2008). Parasitic fauna of the spiny eel, *Mastacembelus mastacembelus* Banks et Solander (Teleostei: Mastacembelidae) in Iran. *Iranian Journal of Veterinary Research, Shiraz University, 9*(2), 158.

**Jalali Jafari, B., & Miar, A.** (2011). Metazoan parasite community of *Capoeta damascina* (Valenciennes in Cuvier and Valenciennes, 1842), Tigris Basin, Mesopotamian region—a checklist. *Iranian Journal of Veterinary Research, 12*(3), 265–270.

**Jansen, D., Vanhove, M. P., Makasa, L., Vorel, J., Kmentová, N., & Cruz-Laufer, A. J.** (2024). Mitogenomics, phylogenetic position, and updated distribution of *Ergasilus kandti*, an ergasilid copepod parasitizing African cichlid fishes. *Hydrobiologia*, 1–26.

**Jarallah, H. M., Al-Daraji, S. A. M., Bannai, M. A. A., Mohamad, E. T., & Wanis, A. K.** (2005). New description of disease agents affecting mosquito fish (*Gambusia affinis*) in the Basrah marshes. *1st Scientific Conference on the Rehabilitation of the Southern Iraq Marshes*. Basrah, Iraq: 11-12 April 2005. (Abstract).

**Jarallah, H. M., Ail, A. I., Hassouni, Z. A., Farhan, F. J., Mohamed, B. A., & Jawher, N. K.** (2024, November). Infection with crustacean parasites *Lernaea* sp. in culturing fishes in Basrah marshes. In *AIP Conference Proceedings* (Vol. 3219, No. 1). AIP Publishing.

**Jažić, A**. (1995). *Parazitofauna šarana i njen epizootiološki značaj na ribnjačarstvima u Bosni i Hercegovini* [Carp parasitofauna and its epizootiological significance in fish farms in Bosnia and Herzegovina] (PhD thesis). University of Sarajevo, Bosnia and Herzegovina.

**Jori, M. M.** (2006). *Parasitic study on the Asian catfish Silurus triostegus (Heckel, 1843) from Al-Hammar marshes, Basrah, Iraq* (Ph.D. thesis). College of Education, University of Basrah, Iraq.

**Kadim, R.A., & Al-Zubaidy, A.B.** (2009). The pathological effects of the anchor worm *Lernaea cyprinacea* L. on the common carp *Cyprinus carpio* L. *Journal of Babylon University, Pure Science*, 17(3), 929-935. (In Arabic).

**Kamil, M. A., Erdeni, A. A., & Mahmoud Zangana, A. J.** (2022). Diagnosis of external parasites of species of fish of Tigris River passing through Al-Dour district/Iraq. *International Journal of Entomological Research*, *7*(9), 26-33.

**Kashinskaya, E. N., Simonov, E. P., Andree, K. B., Vlasenko, P. G., Polenogova, O. V., Kiriukhin, B. A., & Solovyev, M. M.** (2021). Microbial community structure in a host–parasite system: The case of Prussian carp and its parasitic crustaceans. *Journal of Applied Microbiology, 131*(4), 1722–1741.

**Kasim, M. H., & Rahemo, Z. I. F.** (1981). Influence of seasons and sex on the intensity of *Pseudolamproglena annulata* Boxshall, 1976 (Lernaeidae) infection in *Cyprinion macrostomum*, a freshwater teleost from the River Tigris. *Rivista di Parassitologia*, *42*(3), 455-460.

**Karakisi, H., & Demir, S.** (2012). Metazoan parasites of the common carp (*Cyprinus carpio* L., 1758) from Tahtali Dam Lake (Izmir). *Türkiye Parazitoloji Dergisi, 36*(3), 174.

**Khalifa, K. A., Hassan, F. K., Atiah, H. H., & Latif, B. M. A.** (1978). Parasitic infestation of fishes in Iraqi waters. *Iraqi Journal of Biological Sciences*, *6*(1), 58–63.

**Kamil, M. A., Erdeni, A. A., & Mahmoud Zangana, A. J.** (2022). Diagnosis of external parasites of species of fish of Tigris River passing through Al-Dour district, Iraq. *International Journal of Entomological Research, 7*(9), 26–33.

**Khalifa, K. A.** (1982). Occurrence of parasitic infections in Iraqi fish ponds. *2nd Scientific Conference, Arab Biological Union*, Fés, 17-20 March 1982, 333. (Abstract).

**Khalifa, K. A.** (1989). Incidence of parasitic infestation of fishes in Iraq. *Pakistan Veterinary Journal, 9*(2), 66–69.

**Khalifa, K. A., Hassan, F. K., Atiah, H. H., & Latif, B. M. A.** (1978). Parasitic infestation of fishes in Iraqi waters. *Iraqi Journal of Biological Sciences, 6*(1), 58-63.

**Khamees, N. R.** (1996). *Ecological and biological studies of some copepods (Family Ergasilidae) infesting gills of the mugilid fish, Liza abu from Basrah* (Ph.D. thesis). College of Agriculture, University of Basrah, Iraq, 92 pp.

**Khamees, N. R.** (1997). First occurrence of the anchor worm *Lernaea cyprinacea* L. in fishes of Shatt Al-Arab river, Basrah, Iraq. *Al-Mustansiriya Journal of Science, 8*(3), 1–4.

**Khamees, N. R., & Mhaisen, F. T.** (1988). Ecology of parasites of the cyprinid fish *Carasobarbus luteus* from Mehaijeran Creek, Basrah. *Journal of Biological Science Research, 19*(2), 409-419.

**Khamees, N. R., & Mhaisen, F. T.** (1995). Two copepod crustaceans as additional species to the parasitic fauna of fishes of Iraq. *Basrah Journal of Science*, *13*(1), 49–56.

**Khamees, N. R., & Mhaisen, F. T.** (1998, November 3–4). *Attachment methods for species of Ergasilidae (Copepoda) parasitic on gills of Liza abu* (Abstract, in Arabic). Scientific Conference on Fisheries and Marine Resources, Basrah, Iraq.

**Khamees, N. R., & Mhaisen, F. T.** (2001). Some ecological aspects of *Ergasilus rostralis* (Copepoda: Crustacea) from the mullet, *Liza abu*, from Shatt Al-Arab River. *Al-Mustansiriya Journal of Science, 12*(5), 21–28.

**Khara, H., Sattari, M., Nezami, S., Mirhasheminasab, S. F., & Mousavi, S. A.** (2005, September 11–16). Parasites of some bony fishes in Amirkelayeh wetland from the southwest of the Caspian Sea [Conference presentation abstract]. *12th EAFP International Conference on Diseases of Fish and Shellfish*, Copenhagen, Denmark, p. 135.

**Khara, H., Sattari, M., Nezami, S., Mirhasheminasab, S. F., Mousavi, S. A., & Ahmadnezhad, M.** (2011). Parasites of some bonyfish species from the Boojagh wetland in the southwest shores of the Caspian Sea. *Caspian Journal of Environmental Sciences, 9*(1), 47–53.

**Khoris, E. A., & Bileh, S. S.** (2024). Effect of Artemisia extract on *Argulus coregoni* and *Lernaea cyprinacea* infestation in carp fish. *Journal of Advanced Veterinary Research, 14*(6), 969-974.

**Kır, İ., & Samancı, İ.** (2012). Karacaören II Baraj Gölü (Burdur, Isparta)’nde yaşayan havuz balığı (Carassius gibelio Bloch., 1782)’nın parazit faunası. *Süleyman Demirel Üniversitesi Eğirdir Su Ürünleri Fakültesi Dergisi, 8*(2), 38-42.

**Korkut, N., & Koyun, M.** (2022). Crustacean and protozoan parasites of some cyprinid fish living in the Murat River (Bingöl-Türkiye), with new host records. *Aquatic Research, 5*(2), 154–164.

**Koyun, M., Altunel, F. N., & Öktener, A.** (2007). *Paraergasilus longidigitus* Yin, 1954 (Copepoda: Poecilostomatoida) infestations in the bleak, *Alburnus alburnus* Lin., 1758 from Enne Dam Lake. *Türkiye Parazitoloji Dergisi, 31*(2), 158–161.

**Koyun, M., Ulupınar, M., & Gül, A**. (2015). Seasonal distribution of metazoan parasites on Kura barbell (*Barbus lacerta*) in Eastern Anatolia, Turkey. *Pakistan Journal of Zoology, 47*(5), 1253–1261.

**Koyun, M., & Atıcı, A.** (2018). First record of *Lernaea cyprinacea* (Crustacea: Copepoda) on *Capoeta capoeta* (Actinopterygii: Cyprinidae) from Karasu Creek (Van) Eastern Anatolia. *Biharean Biologist, 12*(2), 70-72.

**Koyun, M., Korkut, N., & Gül, A.** (2019). Occurrence of endo and ectoparasites on *Capoeta trutta* (Heckel, 1843) and *Acanthobrama marmid* Heckel, 1843 (Cypriniformes: Cyprininae) inhabiting in Göynük Stream Eastern Anatolia. *Biharean Biologist*, *13*(2).

**Koyuncu, C. E.** (2002). *Histopathology, effects, incidence and determination of ectoparasites on aquarium (Cyprinidae and Poecilidae) fish and their control* (Ph.D. thesis). Çukurova University, Institute of Science. Turkey. (In Turkish).

**Kvach, Y., Tkachenko, M. Y., Seifertová, M., & Ondračková, M.** (2021). Insights into the diversity, distribution and phylogeny of three ergasilid copepods (Hexanauplia: Ergasilidae) in lentic water bodies of the Morava river basin, Czech Republic. *Limnologica, 91*, 125922.

**Kvach, Y., Tkachenko, M. Y., Giannetto, D., Míč, R., Bartáková, V., Ağdamar, S., Saç, G., Özuluğ, M., Tarkan, A. S., & Ondráčková, M.** (2024). Low genetic and parasite diversity of invasive pumpkinseed *Lepomis gibbosus* (Centrarchidae) expanding in Türkiye. *Diversity*, *16*, 272.

**Lahav, M., & Sarig, S.** (1967). *Ergasilus sieboldi* Nordman infestation of grey mullet in Israel fish ponds. *Bamidgeh*, *19*(4), 69-80.

**Lui, A., Manera, M., Giari, L., Mulero, V., & Dezfuli, B. S.** (2013). Acidophilic granulocytes in the gills of gilthead seabream *Sparus aurata*: Evidence for their responses to a natural infection by a copepod ectoparasite. *Cell and Tissue Research, 353*, 465-472.

**Macchioni, F., Chelucci, L., Torracca, B., Prati, M. C., & Magi, M.** (2015). Fishes and their parasites in the water district of Massaciuccoli (Tuscany, Central Italy). *Veterinaria Italiana, 51*(3), 199–203.

**Maceda-Veiga, A., Mac Nally, R., Green, A. J., Poulin, R., & de Sostoa, A.** (2019). Major determinants of the occurrence of a globally invasive parasite in riverine fish over large-scale environmental gradients. *International Journal for Parasitology, 49*(8), 625–634.

**Mahmoud, M. A., Aly, S. M., Diab, A. S., & John, G.** (2009). The role of ornamental goldfish *Carassius auratus* in transfer of some viruses and ectoparasites to cultured fish in Egypt: Comparative ultra-pathological studies. *African Journal of Aquatic Science, 34*(2), 111-121.

**Malekzehi, M. H., Esmaeili, H. R., Zareian, H., Farahani, Z., & Pazira, A.** (2014). Incidence of *Lernaea* (Crustacea: Copepoda) parasitic in the Mashkid River Basin, Southeast of Iran. *International Journal of Aquatic Biology, 2*(1), 9–13.

**Mama, K. S., & Abdullah, S. M. A.** (2012). A comparative study on the parasitic fauna of the common carp *Cyprinus carpio* from Ainkawa fish hatchery (Erbil) and Lesser Zab river in Kurdistan region, Iraq. *Mesopotamia Journal of Agriculture*, 42(2), 19–26.

**Mama, K. S., & Abdullah, S. M. A.** (2013). Parasitic infections of the common carp *Cyprinus carpio* from Lesser Zab River in Kurdistan region, Iraq. In *Proceedings of the 1st Annual International Interdisciplinary Conference (AIIC)* (pp. 895–900). Azores, Portugal, April 24–26, 2013.

**Mancinelli, G., Mali, S., & Belmonte, G.** (2019). Species richness and taxonomic distinctness of zooplankton in ponds and small lakes from Albania and North Macedonia: The role of bioclimatic factors. *Water, 11*(11), 2384.

**Manshadi, A. G., Beladi, S. M. R., Tarahomi, M., & Kazerun, I.** (2024). *Lernaea cyprinacea* Linnaeus, 1758 (Cyclopoida: Lernaeidae) in grass carp (Ctenopharyngodon idella) from a commercial fish farm in the rearing ponds of Shushtar, Khuzestan province. *Journal of Alternative Veterinary Medicine*, *7*(20).

**Mansoor, N. T., & Al-Shaikh, S. M. J.** (2011). Isolate two crustaceans which infect *Cyprinus carpio* L. from Bab Al-Muatham fish markets, Baghdad City. *Iraqi Journal of Veterinary Medicine, 35*(1), 54–60.

**Mansoor, N. T., Falah, A. B., Al-Jawda, J. M., & Asmar, K. R.** (2012). Histo-pathological study of some Tigris River fish which infected by parasites. *Iraqi Journal of Veterinary Medicine, 36*(1), 33–42. (In Arabic).

**Marques, J. F., Santos, M. J., & Cabral, H. N.** (2006). Soleidae macroparasites along the Portuguese coast: Latitudinal variation and host–parasite associations. *Marine Biology, 150*(2), 285–298.

**Marques, J. F., Santos, M. J., & Cabral, H. N.** (2009). Zoogeographical patterns of flatfish (Pleuronectiformes) parasites in the Northeast Atlantic and the importance of the Portuguese coast as a transitional area. *Scientia Marina, 73*(3), 461–471.

**Marques, J. F., Santos, M. J., Teixeira, C. M., Batista, M. I., & Cabral, H. N.** (2011). Host–parasite relationships in flatfish (Pleuronectiformes) – The relative importance of host biology, ecology and phylogeny. *Parasitology, 138*(1), 107–121.

**Marzouk, M. S., Ezz El-Din, N. M., Mohamed, M. E., Mahmoud, M. A., & Mohamed, R. S.** (2002). Epizootiological and pathological investigations on fishes of Lake Manzala infested with some gill and intestinal parasites. *Egyptian Journal of Agricultural Research, 80*(2), 901–913.

**Marzouk, M. S., Abdelaziz, M. A., Moustafa, M., & Abotorkia, S.** (2010). Monitoring of seasonal parasitic and mycotic problems affecting cultured *Oreochromis niloticus* under semi-intensive system. *Assiut Veterinary Medical Journal*, *56*(125), 1–25.

**McCredden, M.** (2016). *Anchors away: The susceptibility and response to infection between native and co-introduced fishes to the alien anchor worm Lernaea cyprinacea* (Ph.D. thesis). Murdoch University, USA.

**Mehdi, D. S., Berghash, A. J., & Abid-Ali, J. M.** (2009). Study of seasonal variation of infected fish (*Liza abu*) with three types of parasites. *Al-Taqani*, 22(2), 53-57. (In Arabic).

**Mhaisen, F. T.** (1982). The anchor worm, *Lernaea cyprinacea*, in Basrah University fish farm. *Iraqi Journal of Marine Science, 1*(1), 3-11.

**Mhaisen, F.T.** (1983). *Diseases and parasites of fishes*. Basrah University Press. (In Arabic).

**Mhaisen, F.T.** (1986). Records of some fish parasites from Shatt-Al-Arab River and the northwest of the Arab Gulf. *Bulletin of Basrah Natural History Museum*, *6*(1), 111-124.

**Mhaisen, F. T., Al-Salim, N. K., & Khamees, N. R.** (1986). The parasitic fauna of two cyprinids and a mugilid fish from Mehaijeran Creek, Basrah. *Journal of Biological Science Research, 17*(3), 63-73.

**Mhaisen, F. T., Al-Salim, N. K., & Khamees, N. R.** (1988). Occurrence of parasites of the freshwater mugilid fish *Liza abu* (Heckel) from Basrah, southern Iraq. *Journal of Fish Biology, 32*(4), 525–532.

**Mhaisen, F. T., Ali, N. M., Abul-Eis, E. S., & Kadim, L. S.** (1989). Protozoan and crustacean parasites of the mugilid fish *Liza abu* (Heckel) inhabiting Babylon fish farm, Hilla, Iraq. *Journal of Biological Sciences Research*, *20*(3), 517–525.

**Mhaisen, F. T., Khamees, N. R., & Al-Sayab, A. A.** (1990). Flat worms (Platyhelminthes) of two species of gulls (*Larus ichthyaetus* and *L. canus*) from Basrah, Iraq. *Zoology in the Middle East*, *4*, 113–116.

**Mhaisen, F. T., & Abul-Eis, E. S**. (1991). Parasites of the common carp *Cyprinus carpio* in the Babylon Fish Farm, Hilla, Iraq. *Thalassographica, 14*, 27–33.

**Mhaisen, F. T., & Abul-Eis, E. S.** (1993). External parasites of Al-Wahda Fish Hatchery at Suwaira, south of Baghdad. *Marine Mesopotamia, 8*(2), 202–206.

**Mhaisen, F. T., Balasem, A. N., Al-Khateeb, G. H., Al-Shaikh, S. M. J., & Al-Jawda, J. M.** (1993a). Survey of parasites of farm fishes in three provinces in mid Iraq. *Al-Tharwa Al-Samakia*, 13, 84-87. (In Arabic).

**Mhaisen, F. T., Balasem, A. N., Al-Khateeb, G. H., Al-Shaikh, S. M. J., Al-Jawda, J. M., & Haiawi, S. M.** (1993b). Survey of parasites of three fish farms at Al-Latifiya, south Baghdad. *Marine Mesopotamia*, 8(2), 218-224.

**Mhaisen, F. T., Al-Yamour, K. Y., & Allouse, S. B.** (1995). Parasites of some freshwater fishes from Tigris River at Al-Rashidia, north of Baghdad, Iraq. *Arqivos do Museu Bocage, Nova Série, 2*(32), 547–554.

**Mhaisen, F. T., Al-Saadi, A. A. J., & Al-Shamma’a, A. A.** (1999). Some observations on fish parasites of Habbaniya Lake. *Ibn Al-Haitham Journal for Pure and Applied Science, 12*(1), 62–67.

**Mhaisen, F. T., & Khamees, N. R.** (2001). Control of some copepod crustaceans parasitic on gills of the mugilid fish *Liza abu*. *Ibn Al-Haitham Journal of Pure and Applied Sciences, 14*(3), 24–32.

**Mhaisen, F. T., Al-Khateeb, G. H., Balasem, A. N., Al-Shaikh, S. M. J., Al-Jawda, J. M., & Mohammad-Ali, N. R.** (2003). Occurrence of some fish parasites in Al-Madaen drainage network, south of Baghdad. *Bulletin of the Iraq Natural History Museum, 10*(1), 39–47.

**Mhaisen, F. T., Al-Rubaie, A. L., & Al-Sa’adi, B. A.** (2015). Crustaceans and glochidians of fishes from the Euphrates River at Al-Musaib City, Babylon Province, Mid Iraq. *American Journal of Biological and Life Sciences*, *3*(4), 116–121.

**Mhaisen, F. T., & Abdul-Ameer, K. N.** (2021). Checklist of fish hosts of species of *Lernaea* Linnaeus, 1758 (Hexanauplia: Cyclopoida: Lernaeidae) in Iraq. *Biological and Applied Environmental Research*, *5*(1), 53-73.

**Mhaisen, F. T., & Al-Daraji, S. A.** (2023). Checklists of species of *Ergasilus* von Nordmann, 1832 (Copepoda: Ergasilidae) parasitic on fishes of Iraq. *Iraqi Journal of Aquaculture*, *20*(2), 211-244.

**Mhaisen, F., Ali, A., & Adday, T.** (2024). Checklists of Fish Species Infected with Parasites of the Genera *Lamproglena* and *Pseudolamproglena* (Copepoda: Cyclopoida: Lernaeidae) in Iraq. *Iraqi Journal of Aquaculture*, *21*(2), 1-16.

**Míč, R., Řehulková, E., & Seifertová, M.** (2023). Species of *Ergasilus* von Nordmann, 1832 (Copepoda: Ergasilidae) from cichlid fishes in Lake Tanganyika. *Parasitology, 150*(7), 579–598.

**Míč, R., Řehulková, E., Šimková, A., Razanabolana, J. R., & Seifertová, M.** (2024). New species of *Dermoergasilus* Ho & Do, 1982 (Copepoda: Cyclopoida: Ergasilidae) parasitizing endemic cichlid *Paretroplus polyactis* (Bleeker) in Madagascar. *Parasitology, 151*(3), 319–336.

**Mir Hashemi Nasab, F., & Pazoki, J.** (2003). Identification of crustacean parasites in some fishes of Mahabad Reservoir. *Iranian Scientific Fisheries Journal, 11*(4), 133–148.

**Mirzaei, M., Khovand, H., & Kheirandish, R.** (2016). The prevalence of non-indigenous parasitic copepod (*Neoergasilus japonicus*) spreads with fishes of pet trade in Kerman, Iran. *Journal of Parasitic Diseases, 40*, 1283–1288.

**Mohammad-Ali, N. R., Balasem, A. N., Mhaisen, F. T., Salih, A. M., & Waheed, I. K.** (1999). Observations on the parasitic fauna in Al-Zaafaraniya Fish Farm, south of Baghdad. *Veterinary*, 9(2), 79-88.

**Mokhayer, B.** (1985). *Diseases of cultured fishes*. University of Tehran Publication. 318 p. (In Persian).

**Molnar, K.** (1990). Report of the fish pathology course held in Iran from 24 Nov to 21 Dec. *Fisheries Co of Iran*. 11 p.

**Molnar, K., & Baska, F.** (1993). *Scientific report on intensive training course on parasites and parasitic diseases of freshwater fishes of Iran* (15-25 Nov.). Fisheries Co. of Iran. 15 p.

**Momeni, H., Peyghan, R., Bashiri, M., & Bahrami, S.** (2024). Molecular and histopathological study and first report of *Lernaea cyprinacea* infestation in *Oncorhynchus mykiss* in the Chaharmahal and Bakhtiari province. *Research Square*.

**Moreno, O., Granado, C., & Garcia-Novo, F.** (1986). Variabilidad morfológica de *Lernaea cyprinacea* (Crustacea: Copepoda) en el embalse de Arrocampo (Cuenca de Tajo: Cáceres). *Limnetica, 2*, 275–280.

**Muhammad, I. M., Dhahir, S. F., Bilal, S. J., & Abdullah, S. M. A.** (2013). Parasitic fauna of some freshwater fishes from Greater Zab River, Kurdistan Region, Iraq. *Journal of University of Zakho*, *1*(A), 2, 620-627.

**Muñoz-Colmenares, M. E., Sendra, M. D., Sòria-Perpinyà, X., Soria, J. M., & Vicente, E.** (2021). The use of zooplankton metrics to determine the trophic status and ecological potential: An approach in a large Mediterranean watershed. *Water, 13*(17), 2382.

**Naem, S., Moobedi, I., Khomirani, R., & Abolghasemi, S. G.** (2000). A survey on parasites of gills of cultured and wild fishes of western branch of Sefid-Rud River (Gilian Province) and introducing a new species (in Persian). *Iranian Journal of Veterinary Research, University of Shiraz, 3*(2), 118-130.

**Naji, H. S.** (2010). Prevalence of parasitic fauna infection in aquarium fish with investigation of treatment methods. *Al-Kufa University Journal for Biology., 2*(1), 21-24. (In Arabic).

**Narciso, R. B., Smit, N. J., Perbiche-Neves, G., & da Silva, R. J.** (2024). Integrative taxonomy approach to the study of parasitic ergasilids (Cyclopoida: Ergasilidae) of fishes from the Pardo River, Brazil with a redescription of *Rhinergasilus piranhus* Boeger and Thatcher, 1988 and a molecular phylogeny for Ergasilidae. *Parasitology*, 1–24.

**Nedić, Z., Skenderović, I., & Riđanović, S.** (2014). Skin ectoparasites of fish from the lower flow of the Sava River. *Veterinaria*, *63*(1-4), 45-53.

**Nematollahi, A., Ahmadi, A., Mohammadpour, H., & Ebrahimi, M.** (2013). External parasite infection of common carp (*Cyprinus carpio*) and big head (*Hypophthalmichthys nobilis*) in fish farms of Mashhad, northeast of Iran. *Journal of Parasitic Diseases, 37*, 131-133.

**Nofal, M. E., Zaki, V. H., & El-Shebly, A. A.** (2016). Implications of heavy infestation of *Lernaea cyprinacea* (Crustacea: Copepoda) of silver carp, *Hypophthalmichthys molitrix* at Manzala area, with trial for control using Trichlorfon. *International Journal of Fisheries and Aquatic Studies*, *4*(6), 314-318.

**Noor El-Deen, A. I. E., Azza, H. M. H., & Abeer, E. M.** (2013). Studies on lernaeosis affecting cultured golden fish (*Carassius auratus*) and trial for its treatment in earthen ponds at Kafr El-Sheikh governorate, Egypt. *Global Veterinaria, 11*, 521-527.

**Oguz, M. C., & Öktener, A.** (2007). Four parasitic crustacean species from marine fishes of Turkey. *Türkiye Parazitoloji Dergisi*, *31*(1), 79-83.

**Öktener, A., & Trilles, J. P.** (2004). Three new parasitic copepod species for the parasite fauna of marine fish of Turkey. *Journal of Black Sea/Mediterranean Environment, 10*(1).

**Öktener, A., Ali, H., & Alaş, A.** (2008). *Ergasilus mosulensis* Rahemo, 1982 (Ergasilidae) on *Chalcalburnus mossulensis* Heckel, 1843 (Cyprinidae) from Turkey. *Bulletin of the European Association of Fish Pathologists, 28*(5), 193-197.

**Ondračková, M., Kvach, Y., Tkachenko, M. Y., Pravdová, M., Seifertová, M., Bartáková, V., & Jurajda, P.** (2025). The role of North American bullhead catfish as parasite reservoirs in Central European fishing grounds. *Aquaculture, 599*, 742100.

**Özer, A.** (2007). Metazoan parasite fauna of the round goby *Neogobius elanostomus* Pallas, 1811 (Perciformes: Gobiidae) collected from the Black Sea coast at Sinop, Turkey. *Journal of Natural History, 41*(9-12), 483–492.

**Özer, A., & Kirca, D. Y.** (2013). Parasite fauna of golden grey mullet *Liza aurata* (Risso, 1810) collected from Lower Kızılırmak Delta in Samsun, Turkey. *Helminthologia, 50*(4), 269-280.

**Özer, A., Öztürk, T., Sezgin, M., Bat, L., Ürkmez, D., Arıcı, E., & Öztürk, B.** (2017). Parasite diversity of the Black Sea fishes in Turkish coastal areas. In B. Öztürk (Ed.), *Black Sea marine environment: The Turkish shelf* (pp. 289–310). Turkish Marine Research Foundation (TUDAV).

**Öztürk, M. O.** (2002). Metazoan parasites of the tench (*Tinca tinca* L.) from Lake Uluabat, Turkey. *Israel Journal of Zoology, 48*(4), 285–293.

**Öztürk, T.** (2005). *Determination of parasite fauna of flounder, Platichthys flesus L., 1758 and toothcarp Aphanius chantrei Gaillard 1895 present in Sarkum Lagoon Lake, Sinop, Turkey* (Doctoral dissertation). Ondokuz Mayıs University, Institute of Science. (In Turkish).

**Öztürk, T.** (2013). Parasites of juvenile golden grey mullet *Liza aurata* Risso, 1810 in Sarıkum Lagoon Lake at Sinop, Turkey. *Acta Parasitologica, 58*(4), 531-540.

**Öztürk, M. O., Oğuz, M. C., & Aydogdu, A.** (2002). An investigation of metazoan parasitic fauna of pike (*Esox lucius* L.) and rudd (*Scardinius erythrophthalmus* L.) from the Karacabey Lagoon. *The Turkish Journal of Parasitology, 26*(3), 325–328.

**Öztürk, M. O., & Aydoğdu, A.** (2003). Metazoan parasites of grey mullet from Karacabey Bayramdere Lagoon. *Journal of Veterinary Faculty, Ankara University, 50*(1), 53–58.

**Öztürk, T., & Özer, A.** (2008). Parasitic fauna of the toothcarp *Aphanius danfordii* (Boulenger, 1890) (Osteichthyes: Cyprinodontidae), an endemic fish from Sarıkum Lagoon Lake in Sinop (Turkey). *Journal of Fisheries Sciences.com, 2*(3), 388–402.

**Paperna, I.** (1964). Parasitic Crustacea (Copepoda and Branchiura) from inland water fishes of Israel. *Israel Journal of Ecology and Evolution*, *13*(2), 58–68.

**Paperna, I.** (1975). Parasites and diseases of the grey mullet (Mugilidae) with special reference to the seas of the Near East. *Aquaculture, 5*(1), 65–80.

**Paperna, I., & Lahav, M.** (1971). New records and further data on fish parasites in Israel. *Bulletin of Fish Culture Israel (Bamidgeh)*, *16*, 77–86.

**Paperna, I., & Overstreet, R. M.** (1981). Parasites and diseases of mullets (Mugilidae). In O. H. Oren (Ed.), *Aquaculture of grey mullets* (IBP 26, pp. 1–19). Cambridge University Press.

**Pazooki, J., Masoumian, M., & Ghobadian, M.** (2005). Identification of parasites of some fishes in water resources of Zanjan Province. *Iranian Scientific Fisheries Journal*, *1*, 23–35.

**Pazooki, J., & Masoumian, M**. (2012). Synopsis of the parasites in Iranian freshwater fishes. *Iranian Journal of Fisheries Sciences*, *11*(3), 570-589.

**Pérez-Bote, J. L.** (2000). Occurrence of *Lernaea cyprinacea* (Copepoda) on three native cyprinids in the river Guadiana (SW Iberian Peninsula). *Research and Reviews in Parasitology, 60*(3/4), 135-136.

**Pérez-Bote, J. L.** (2005). First record of *Lernaea cyprinacea* (Copepoda: Cyclopoida) on the allis shad. *Folia Biologica (Kraków), 53*(3–4), 197–198.

**Peyghan, R., Dadar, M., & Razijalaly, M. H.** (2011). The diversity of the freshwater fish fauna and metazoan parasites of fishes in the Zohreh River, Khuzestan Province, South-West of Iran. In *Water biodiversity assessment and protection* (p. 197).

**Piasecki, W., Khamees, N. R., & Mhaisen, F. T.** (1991). A new species of *Mugilicola* Tripathi, 1960 (Crustacea, Copepoda, Therodamasidae) parasitic on Iraqi fish. *Acta Ichthyologica et Piscatoria, 21*(2), 143-151.

**Piasecki, W., Al-Daraji, S. A. M., & Mhaisen, F. T.** (1993). Preliminary survey on copepod parasites of four fish species from Khor-Al-Zubair Lagoon, Iraq. *Aquaculture Association of Canada's 10th Annual Meeting Conference*, Charlottetown, 24-27 Aug. 1993. (Abstract).

**Pofuk, M.** (2021). Non-indigenous parasites of fish in inland waters of Croatia. *Croatian Journal of Fisheries: Ribarstvo, 79*(4), 187-204.

**Prastowo, J., Priyowidodo, D., Nugraheni, Y. R., Sahara, A., Nurcahyo, W., & Ninditya, V. I.** (2023). Molecular and morphological identification of *Lernaea* spp. in cyprinid fishes from two districts in Yogyakarta, Indonesia. *Veterinary World, 16*(4), 851–858.

**Radujković, B. M.** (1983). Parasitofaune de muges de l'Adriatique (*Chelon labrosus* Risso, *Liza aurata* Risso et *Liza saliens* Risso) et son influence sur la condition des hôtes. *Rapports et Procès-Verbaux des Réunions de la Commission Internationale pour l'Exploration Scientifique de la Mer Méditerranée, 28*(6), 271–272. (In French).

**Radujkovic, B. M., & Raibaut, A.** (1987). Copépodes parasites des poissons des côtes du Monténégro (Adriatique sud). Première série. [Copepod parasites of fish from the coasts of Montenegro (southern Adriatic). First series.]. *Acta Adriatica*, *28*(1-2), 121–142. (Serbo-Croatian summary).

**Ragias, V., Athanassopoulou, F., & Sinis, A.** (2005). Parasites of *Mugilidae* spp. reared under semi-intensive and intensive conditions in Greece. *Bulletin of the European Association of Fish Pathologists, 25*(3), 107–113.

**Rahemo, Z. I. F.** (1977). Recording of two new hosts of *Lamproglena pulchella* Nordmann, 1832 (Crustacea: Decapoda) in Iraq. *Iraqi Journal of Biological Sciences, 5*(1), 82–83.

**Rahemo, Z. I. F.** (1982). Two new species of *Ergasilus* (Copepoda: Cyclopoida) from the gills of two Iraqi freshwater fishes. *Bulletin of Basrah Natural History Museum*, *5*, 39–59.

**Rahemo, Z. I. F.** (1995). Studies on the parasites of Garra rufa Heckel, 1843 (Pisces: Cyprinidae). *Rivista Parassitologica*, *12*(56), 273-278.

**Rahemo, Z. I. F.** (2011). Parasitic fauna of the freshwater fish (Arath) *Acanthobrama marmid* caught from River Tigris passing through Mosul City, Iraq. *VIII International Symposium of Fish Parasites*, Vina del Mar, Chile: 26-30 Sept. 2011 (Abstract).

**Rahemo, Z. I. F., & Al-Kallak, S. N. H.** (1998). Parasitic fauna of the freshwater fish, *Barbus luteus*, from Tigris River passing through Hammam Al-Alil, Mosul, Iraq. *Türk Parazitoloji Dergisi*, *22*(3), 330-333.

**Rahemo, Z. I. F., & Al-Niaeemi, B. H. S.** (1999). Observations on the histopathology caused by some parasites of the freshwater fish, *Silurus glanis* L. *Rivista di Parassitologia*, 16(60), No. 3, 227-235.

**Rahemo, Z. I. F., & Ami, S. N**. (2013). Studies on the freshwater fish (bizz), *Barbus esocinus* caught from Mosul Dam Lake, Iraq. *Journal of University of Zakho*, *1*(A), 692-698.

**Rahemo, Z. I. F., Ami, S. N., & Taha, K.** (2013). Studies on the freshwater fish (bizz), *Barbus esocinus* caught from Mosul Dam Lake, Iraq. In *1st International Scientific Conference, Zakho University* (pp. 1-13). Zakho University, 23-25 April 2013.

**Rahimi, M. T., Gholami, Z., Esmaeili, H. R., & Mobedi, I.** (2013). Survey on ectoparasites of *Aphanius sophiae* (Cyprinodontidae) from Southern Iran. *Journal of Coastal Life Medicine, 1*(2), 85-87.

**Rahmati-Holasoo, H., Marandi, A., Shokrpoor, S., Goodarzi, T., Ziafati Kafi, Z., Ashrafi Tamai, I., & Ebrahimzadeh Mousavi, H.** (2023). Clinico-histopathological and phylogenetic analysis of protozoan epibiont *Epistylis wuhanensis* associated with crustacean parasite *Lernaea cyprinacea* from ornamental fish in Iran. *Scientific Reports, 13*(1), 14065.

**Rahnama, M., Khedrı, J., Ahmadzadeh, N., Jamshıdıan, A., Sattarı, A., & Bamorovat, M.** (2016). A survey on the prevalence and histopathological findings of *Lernaea* spp. in *Schizocypris altidorsalis* fish from Chahnimeh lakes and Hamoun wetland in southeast Iran. *İstanbul Üniversitesi Veteriner Fakültesi Dergisi*, *43*(1), 19–22.

**Raibaut, A., Hassine, O. K. B., & Maamouri, K.** (1971). Copépodes parasites des poissons de Tunisie (première série). *INSTM Bulletin: Marine and Freshwater Sciences, 2*(2), 169-197.

**Raibaut, A., & Altunel, F. N.** (1976). Redescription de *Ergasilus gibbus* Nordmann, 1832, copepode parasite branchial de l'anguille et remarques sur sa répartition géographique. *Bulletin de la Société de Sciences Naturelles de Tunisie*, *11*, 75–80.

**Raissy, M., & Ansari, M.** (2012). Parasites of some freshwater fish from Armand river, Chaharmahal va Bakhtyari province, Iran. *Iranian Journal of Parasitology, 7*(1), 73.

**Raissy, M., Sohrabi, H. R., Rashedi, M., & Ansari, M.** (2013). Investigation of a parasitic outbreak of *Lernaea cyprinacea* Linnaeus (Crustacea: Copepoda) in Cyprinid fish from Choghakhor lagoon. *Iranian Journal of Fisheries Sciences, 12*(3), 680–688.

**Rashed, A.-R. A.-M., & Hussain, M. M. S.** (1988). Preliminary study on the parasites of some freshwater fishes from Greater Zab River, northeast of Iraq. *Zanco, 2*(2), 7–16.

**Rashed, A.-R. A.-M., Othman, H., & Nsayf, Z. M.** (1989). Preliminary study on some freshwater fish parasites from Little Zab, northeast of Iraq. *Journal of Biological Science Research, 20*(3), 107–114. (In Arabic).

**Rindoria, N. M., Dos Santos, Q. M., Ali, S. E., Ibraheem, M. H., & Avenant-Oldewage, A.** (2022). *Lamproglena monodi* Capart, 1944 infecting *Oreochromis niloticus* (Linnaeus, 1758): Additional information on infection, morphology and genetic data. *African Zoology, 57*(2), 98–110.

**Rindoria, N. M., Gichana, Z., Morara, G. N., van Wyk, C., Smit, W. J., Smit, N. J., & Luus-Powell, W. J.** (2023). Scanning electron microscopy and first molecular data of two species of *Lamproglena* (Copepoda: Lernaeidae) from *Labeo victorianus* (Cyprinidae) and *Clarias gariepinus* (Clariidae) in Kenya. *Pathogens, 12*(8), 980.

**Sadek, A.A., Mhaisen, F.T., & Balasem, A.N.** (2006). Ectoparasites of the common carp (*Cyprinus carpio* L.) fingerlings intensively stocked during autumn and winter. *Ibn Al-Haitham Journal of Pure and Applied Science*, 19(4A), 32-40.

**Sadikaj, R., Libohova, R., Arapi, D., & Sadikaj, F.** (2014). The ecological aspects of parasitic fauna in the natural and cultivated populations of fishes. *Journal of Hygienic Engineering and Design, 6*(13), 192–195.

**Şahan, A., & Duman, S.** (2010). Effect of β-glucan on haematology of common carp (*Cyprinus carpio*) infected by ectoparasites. *Mediterranean Aquaculture Journal, 3*(1), 1–7.

**Saleh, O. A., & Mohamed, R. A.** (2002). A contribution on biology of *Lernaea cyprinacea* (Copepoda) parasitizing cultured common carp (*Cyprinus carpio*). *Egyptian Journal of Agricultural Research, 80*(4), 1937-1948.

**Salih, A. M., Balasem, A. N., Al-Jawda, J. M., Asmar, K. R., & Mustafa, S. R.** (2000). On a second survey of fish parasites in Al-Zaafaranya Fish Farm, Baghdad. *Journal of Diyala, 1*(8 Part 1), 220–238. (In Arabic).

**Samman, A.** (1989). Incidence of monogenean species on the gills of common carp (*Cyprinus carpio*) collected from Hungarian and Syrian fish farms. *Parasitology Hungary, 22*, 45-50.

**Sánchez-Hernández, J.** (2017). *Lernaea cyprinacea* (Crustacea: Copepoda) in the Iberian Peninsula: Climate implications on host–parasite interactions. *Knowledge & Management of Aquatic Ecosystems*, *(418)*, 11.

**Santacruz, A., Morales-Serna, F. N., Leal-Cardín, M., Barluenga, M., & Pérez-Ponce de León, G.** (2020). *Acusicola margulisae* n. sp. (Copepoda: Ergasilidae) from freshwater fishes in a Nicaraguan crater lake based on morphological and molecular evidence. *Systematic Parasitology, 97*, 165–177.

**Saraiva, A., & Valente, A. C. N.** (1988). Black spot disease and *Lernaea* sp. infestation on *Leuciscus cephalus* L. (Pisces: Cyprinidae) in Portugal. *Bulletin of the European Association of Fish Pathologists*, *8*, 7–8.

**Saraiva, A., & Eiras, J. C.** (1996). Parasite community of European eel, *Anguilla anguilla* (L.) in the river Este, northern Portugal. *Research and Reviews in Parasitology*, *56*(4), 179-183.

**Sarıeyyüpoğlu, M., & Sağlam, N.** (1991). *Ergasilus sieboldi* and *Argulus foliaceus* in *Capoeta trutta* caught from a polluted region of Keban Dam Lake. *Ege University Journal of Fisheries and Aquatic Sciences, 8*(31), 143–154.

**Sayyadzadeh, G., & Roudbar, A. J.** (2014). Occurrence of *Lernaea cyprinacea* (Crustacea: Copepoda) in an endemic cyprinid fish, *Chondrostoma orientale* Bianco & Banarescu, 1982 from the Kor River Basin, southwestern Iran. *Iranian Journal of Ichthyology*, *1*(3), 214-217.

**Sayyadzadeh, G., Esmaeili, H. R., Ghasemian, S., Mirghiyasi, S., Parsi, B., Zamanpoore, M., & Akhlaghi, M.** (2016). Co-invasion of anchor worms *Lernaea cyprinacea* (Copepoda: Lernaeidae) in some freshwater fishes of the Kor River Basin, Southwest of Iran with some remarks on the ecological aspects of lernaeosis in the country. *Iranian Journal of Fisheries Sciences, 15*(1), 369–389.

**Sharif Rohani, M.** (1994). Survey on parasites and parasitic diseases in Sistan region. In *Proceedings of the 2nd Symposium of Iranian Veterinary Clinics* (pp. 109). Tehran, Iran.

**Simón Vicente, F., Ramajo, V., & Encinas, A.** (1973). Fauna parasitaria de peces españoles de agua dulce: *Allocreadium isoporum* (Trematoda: Allocreadidae); *Lernaea esocina*, *L. cyprinacea* y *Ergasilus* sp. (Crustacea: Copepoda). *Revista Ibérica de Parasitología*, *33*, 633-647.

**Skenderović, I., Adrović, A., Hajdarević, E., Hadžiahmetović Jurida, E., Čekmić, M., & Bajrić, A.** (2015) Parazitski rakovi (Crustacea) ciprinidnih riba iz hidroakumilacije Modrac. Zbornik radova, Naučna konferencija, Lukavac. [Parasitic crustaceans (Crustacea) of cyprinid fish from the Modrac hydroaccumulation. Proceedings, Scientific Conference, Lukavac], pp 669–676. (In Bosnian).

**Soares, I. A., Salinas, V., Ponti, O. D., Mancini, M. A., & Luque, J. L.** (2018). First molecular data for *Lernaea cyprinacea* (Copepoda: Cyclopoida) infesting *Odontesthes bonariensis*, a commercially important freshwater fish in Argentina. *Revista Brasileira de Parasitologia Veterinária, 27*(1), 105–108.

**Song, Y., Wang, G. T., Yao, W. J., Gao, Q., & Nie, P.** (2008). Phylogeny of freshwater parasitic copepods in the Ergasilidae (Copepoda: Poecilostomatoida) based on 18S and 28S rDNA sequences. *Parasitology Research, 102*, 299–306.

**Soylu, E.** (1990). *Sapanca Gölü'ndeki bazı balık türlerinde rastlanan parazit faunası üzerine araştırmalar* (Ph.D. thesis). İstanbul Üniversitesi Deniz Bilimleri ve Coğrafya Enstitüsü, İstanbul, Turkey, 87 p.

**Soylu, E.** (2014). Metazoan parasites of fish species from Lake Gala (Edirne, Turkey). *Ege Journal of Fisheries and Aquatic Sciences*, *31*(4), 187-193.

**Soylu, E., & Soylu, M. P.** (2012). First record of the nonindigenous parasitic copepod *Neoergasilus japonicus* (Harada, 1930) in Turkey. *Turkish Journal of Zoology, 36*(5), 662–667.

**Soylu, E., Çolak, S. Ö., Erdoğan, F., Erdoğan, M., & Tektas, N.** (2013). Microhabitat distribution of *Pseudodactylogyrus anguillae* (Monogenea), *Ergasilus gibbus* and *Ergasilus lizae* (Copepoda) on the gills of European eels (*Anguilla anguilla*, L.), *Acta Zoologica Bulgarica*, *65*(2), 251-257.

**Sterling, J. E., Carbonell, E., Estellés-Zanón, E. J., & Chirivella, J.** (1995). Estudio estacional del parasitismo por *Lernaea cyprinacea* en la madrilla *Chondrostoma toxostoma miegii* (Pisces: Cyprinidae) en un afluente del río Ebro. In *Proceedings of the 4th Iberian Congress of Parasitology*, Santiago de Compostela, Spain (pp. 90–91).

**Tahri, M., Bensaad-Bendjedid, L., Dahel, A., Djebbari, N., Nouara, N., & Bensouilah, M.** (2018). Site specificity—not everything is everywhere—case of gill ectoparasites of European eel *Anguilla anguilla* (Linnaeus, 1758) (Park National of El Kala, Algeria). *Cahiers de Biologie Marine*, *59*, 71–78.

**Tareen, I. U.** (1982). Parasitic infections of commercially important fish in Turkish water and microhabitat utilization. In *Proceedings of the II Conferencia Mediterranean de Parasitologia* (29 September–2 October, Granada, Spain), p. 175.

**Tokşen, E.** (2015). *Argulus foliaceus* (Crustacea: Branchiura) infestation on Oscar, *Astronotus ocellatus* (Cuvier, 1829) and its treatment. *Ege Journal of Fisheries and Aquatic Sciences, 23*(3), 1177-1179.

**Tunç, A. Ö., & Koyun, M.** (2018). Seasonal infection of metazoan parasites on mosul bleak (*Alburnus mossulensis*) inhabiting Murat River and its tributaries in Eastern Anatolia, Turkey. *Türk Tarım ve Doğa Bilimleri Dergisi*, *5*(2), 153-162.

**Ürkü, Ç., & Önalan, Ş.** (2018). First report of *Lernaea cyprinacea* (Copepoda: Lernaeidae) on rainbow trout (*Oncorhynchus mykiss*) from the Sarı Mehmet Dam Lake in Van-Turkey. *European Journal of Biology, 77*(1), 42-45.

**Vagianou, S., Athanassopoulou, F., Ragias, V., Di Cave, D., Leontides, L., & Golomazou, E.** (2006). Prevalence and pathology of ectoparasites of Mediterranean sea bream and sea bass reared under different environmental and aquaculture conditions. *Israeli Journal of Aquaculture*, 58, 78–88.

**van der Spuy, L., Narciso, R. B., Hadfield, K. A., Wepener, V., & Smit, N. J.** (2024). Exploring South Africa's hidden marine parasite diversity: Two new marine *Ergasilus* species (Copepoda: Cyclopoida: Ergasilidae) from the Evileye blaasop, *Amblyrhynchotes honckenii* (Bloch). *Parasitology*, 1–21.

**Yalım, F. B., Emre, N., Emre, Y., & Kaymak, N.** (2023). Influence of the host sex, size, and season on *Ergasilus lizae* infestation of Thicklip Grey Mullet (*Chelon labrosus*, L., 1758) in Beymelek Lagoon Lake (Antalya, Türkiye). *Journal of Limnology and Freshwater Fisheries Research, 9*(3), 147–153.

**Yardımcı, R. E., Ürkü, Ç., & Yardımcı, C. H.** (2018). Parasite fauna of fish in Büyükçekmece Dam Lake. *Erzincan University Journal of Science and Technology, 11*(2), 158–167.

**Yaseen, A.N., Mhaisen, F.T., & Al-Kaisey, M.T.** (2009). Effect of aqueous and alcoholic extracts of leaves of henna *Lawsonia inermis* in treating the common carp *Cyprinus carpio* L. infected with the anchor worm, *Lernaea cyprinacea*. *Iraqi Journal of Agriculture* (Special Issue), 14(5), 150-156. (In Arabic)

**Yassin, A.M.** (2010). Isolation and identification of the parasites of *Liza abu* and *Cyprinus carpio* in Al-Shenafya river. *Journal of Wasit University for Science and Medicine, 3*(1), 34-43. (In Arabic).

**Zarfdjian, M. H., & Economidis, P. S.** (1989). Listes provisoires des rotifères, cladocères et copépodes des eaux continentales grecques. *Biologia Gallo-Hellenica, 15*, 129–146.
